# Health Effects of Overweight and Obesity in 195 Countries over 25 Years.

**DOI:** 10.1056/NEJMoa1614362

**Published:** 2017-06-12

**Authors:** GBD 2015 Obesity Collaborators, Ashkan Afshin, Mohammad H. Forouzanfar, Marissa Reitsma, Patrick Sur, Kara Estep, Alex Lee, Laurie Marczak, Ali H. Mokdad, Maziar Moradi-Lakeh, Mohsen Naghavi, Joseph S. Salama, Theo Vos, Kalkidan Hassen Abate, Cristiana Abbafati, Muktar Beshir Ahmed, Ziyad Al-Aly, Ala'a Alkerwi, Rajaa Al-Raddadi, Azmeraw T. Amare, Adeladza Kofi Amegah, Erfan Amini, Alemayehu Amberbir, Stephen M. Amrock, Ranjit Mohan Anjana, Johan Ärnlöv, Hamid Asayesh, Amitava Banerjee, Aleksandra Barac, Estifanos Baye, Derrick Bennett, Masako Horino Berger, Addisu Shunu Beyene, Sibhatu Biadgilign, Stan Biryukov, Espen Bjertness, Ismael Campos-Nonato, Juan Jesus Carrero, Pedro Cecilio, Kelly Cercy, Liliana G. Ciobanu, Leslie Cornaby, Solomon Abrha Damtew, Lalit Dandona, Rakhi Dandona, Samath D. Dharmaratne, Bruce Bartholow Duncan, Babak Eshrati, Alireza Esteghamati, Valery Feigin, João C. Fernandes, Thomas Fürst, Tsegaye Tewelde Gebrehiwot, Audra Gold, Philimon Gona, Atsushi Goto, Tesfa Dejenie Habtewold, Kokeb Tesfamariam Hadush, Nima Hafezi-Nejad, Simon I. Hay, Maria Inês Schmidt, Farhad Islami, Dube Jara Boneya, Ritul Kamal, Ami Kasaeian, Srinivasa Vittal Katikireddi, Andre Pascal Kengne, Chandrasekharan Nair Kesavachandran, Yousef Khader, Young-Ho Khang, Jagdish Khubchandani, Daniel Kim, Yun Jin Kim, Yohannes Kinfu, Soewarta Kosen, Tiffany Ku, Barthelemy Kuate Defo, G. Anil Kumar, Heidi J. Larson, Mall Leinsalu, Xiaofeng Liang, Stephen S. Lim, Patrick Liu, Alan D. Lopez, Rafael Lozano, Azeem Majeed, Reza Malekzadeh, Deborah Carvalho Malta, Mohsen Mazidi, Colm McAlinden, Steve McGarvey, Desalegn Tadese Mengiste, Zerihun Menlkalew Zenebe, George A. Mensah, Gert Mensink, Haftay Berhane Mezgebe, Erkin Mirrakhimov, Ulrich O. Mueller, Jean Jacques N. Noubian, Carla Makhlouf Obermeyer, Felix Ogbo, Mayowa O. Owolabi, George C. Patton, Farshad Pourmalek, Mostafa Qorbani, Anwar Rafay, Rajesh Kumar Rai, Chhabi Lal Ranabhat, Nikolas Reinig, Saeid Safiri, Joshua A. Salomon, Juan R. Sanabria, Itamar S. Santos, Benn Sartorius, Monika Sawhney, Josef Schmidhuber, Aletta E. Schutte, Sadaf G. Sepanlou, Moretza Shamsizadeh, Sara Sheikhbahaei, Min-Jeong Shin, Rahman Shiri, Ivy Shiue, Hirbo Shore, Diego Augusto Santos Silva, Jonathan Silverberg, Jasvinder Singh, Saverio Stranges, Soumya Swaminathan, Rafael Tabarés-Seisdedos, Fentaw Tadese, Bemnet Amare Tedla, Balewgizie Sileshi Tegegne, Abdullah Sulieman Terkawi, J.S. Thakur, Marcello Tonelli, Roman Topor-Madry, Stefanos Tyrovolas, Kingsley N. Ukwaja, Olalekan A. Uthman, Masoud Vaezghasemi, Tommi Vasankari, Vasiliy V. Vlassov, Stein Emil Vollset, Elisabete Weiderpass, Andrea Werdecker, Joshua Wesana, Ronny Westerman, Yuichiro Yano, Naohiro Yonemoto, Gerald Yonga, Zoubida Zaidi, Ben Zipkin, Christopher J.L. Murray

**Affiliations:** University of Washington, Institute for Health Metrics and Evaluation, Seattle, Washington, 98121, United States; University of Washington, Institute for Health Metrics and Evaluation, Seattle, Washington, 98121, United States; University of Washington, Institute for Health Metrics and Evaluation, Seattle, Washington, 98121, United States; University of Washington, Institute for Health Metrics and Evaluation, Seattle, Washington, 98121, United States; University of Washington, Institute for Health Metrics and Evaluation, Seattle, Washington, 98121, United States; University of Washington, Institute for Health Metrics and Evaluation, Seattle, Washington, 98121, United States; University of Washington, Institute for Health Metrics and Evaluation, Seattle, Washington, 98121, United States; University of Washington, Institute for Health Metrics and Evaluation, Seattle, Washington, 98121, United States; University of Washington, Institute for Health Metrics and Evaluation, Seattle, Washington, 98121, United States; Iran University of Medical Sciences, Department of Community Medicine, Gastrointestinal and Liver Disease Research Center (GILDRC), Preventative Medicine and Public Health Research Center, Tehran, Iran; University of Washington, Institute for Health Metrics and Evaluation, Seattle, Washington, 98121, United States; University of Washington, Institute for Health Metrics and Evaluation, Seattle, Washington, 98121, United States; University of Washington, Institute for Health Metrics and Evaluation, Seattle, Washington, 98121, United States; Jimma University, Jimma, Ethiopia; Sapienza University of Rome, Rome, 185, Italy; Jimma University, Jimma, Ethiopia; Washington University School of Medicine, Saint Louis, Missouri, 63110-1093, United States; Luxembourg Institute of Health, Department of Population Health, Strassen, Luxembourg; Joint Program of Family and Community Medicine, Jeddah, 21454, Saudi Arabia; The University of Adelaide, School of Medicine, Adelaide, South Australia, 5005, Australia; Bahir Dar University, College of Medicine and Health Sciences, Bahir Dar, Ethiopia; University of Cape Coast, Cape Coast, Ghana; Uro-Oncology Research Center, Department of Urology, Tehran, 1419733141, Iran; Dignitas International, Zomba, Malawi; Oregon Health & Science University, Portland, Oregon, 97239, United States; Madras Diabetes Research Foundation, Chennai, 600086, India; Uppsala University, Department of Medical Sciences, Uppsala, 751 85, Sweden; Dalarna University, School of Health and Social Sciences, Falun, 79188, Sweden; Qom University of Medical Sciences, Department of Medical Emergency, Qom, 3713649373, Iran; University College London, Farr Institute of Health Informatics Research, London, NW1 2DA , United Kingdom; University of Belgrade, Faculty of Medicine, Belgrade, 11000, Serbia; Monash University School of Public Health and Preventive Medicine, Kanooka, Victoria, 3168 VIC, Australia; Wollo University, Department of Public Health, Dessie, 1145, Ethiopia; University of Oxford, Nuffield Department of Population Health, Oxford, OX3 7LF, United Kingdom; Nevada Division of Public and Behavioral Health, Bureau of Child, Family & Community Wellness, Carson City, Nevada, 89706, United States; Haramaya University, College of Health and Medical Sciences, Harar, 235, Ethiopia; Independent Public Health Consultants, Addis Ababa, Ethiopia; University of Washington, Institute for Health Metrics and Evaluation, Seattle, Washington, 98121, United States; University of Oslo, Department of Community Medicine and Global Health, Oslo, 318, Norway; National Institute of Public Health, Cuernavaca, 62100, Mexico; Karolinska Institutet, Department of Clinical Science, Intervention and Technology, Stockholm, 14186, Sweden; Universidade do Porto, i3S - Instituto de Investigação e Inovação em Saúde, Porto, 4200-135, Portugal; Universidade do Porto, Departamento de Ciências Biológicas, Faculdade de Farmácia, Porto, 4050-313, Portugal; University of Washington, Institute for Health Metrics and Evaluation, Seattle, Washington, 98121, United States; The University of Adelaide, School of Medicine, Adelaide, South Australia, 5005, Australia; University of Washington, Institute for Health Metrics and Evaluation, Seattle, Washington, 98121, United States; Wolayta Sodo University, College of Health Sciences and Medicine, Wolaita, Ethiopia; Public Health Foundation of India, Gurgaon, 1222002, India; University of Washington, Institute for Health Metrics and Evaluation, Seattle, Washington, 98121, United States; Public Health Foundation of India, Gurgaon, 1222002, India; University of Washington, Institute for Health Metrics and Evaluation, Seattle, Washington, 98121, United States; University of Peradeniya, Department of Community Medicine, Peradeniya, 20400, Sri Lanka; Universidade Federal do Rio Grande do Sul, Porto Alegre, 90035-003, Brazil; Arak University of Medical Sciences, Arak, 3819693345, Iran; Tehran University of Medical Sciences, Endrocrinology and Metabolism Population Sciences Institute, Tehran, Iran; National Institute for Stroke & Applied Neurosciences, Faculty of Health and Environmental Sciences, Auckland, 627, New Zealand; University of Washington, Institute for Health Metrics and Evaluation, Seattle, Washington, 98121, United States; Catholic University of Portugal, Center for Biotechnology and Fine Chemistry, Porto, P-4202-401, Portugal; Imperial College London, Department of Primary Care & Public Health, London, W6 8RP, United Kingdom; Swiss Tropical and Public Health Institute, Department of Epidemiology and Public Health, Basel, Switzerland; Jimma University, Jimma, Ethiopia; University of Washington, Institute for Health Metrics and Evaluation, Seattle, Washington, 98121, United States; University of Massachusetts Boston, Boston, Massachusetts, 02125, United States; Center for Public Health Sciences, National Cancer Center, Division of Epidemiology, Tokyo, 104-0045, Japan; University of Groningen, Groningen, 9700 RB, The Netherlands; Ambo University, Ambo, 11, Ethiopia; Tehran University of Medical Sciences, Endrocrinology and Metabolism Population Sciences Institute, Tehran, Iran; University of Washington, Institute for Health Metrics and Evaluation, Seattle, Washington, 98121, United States; University of Oxford, Oxford Big Data Institute, Li Ka Shing Centre for Health Information and Discovery, Oxford, OX3 7BN, United Kingdom; Universidade Federal do Rio Grande do Sul, Porto Alegre, 90035-003, Brazil; American Cancer Society, Surveillance and Health Services Research, Atlanta, Georgia, 30303, United States; Debre Markos University, Department of Public Health, Debre Markos, 251269, Ethiopia; CSIR-Indian Institute of Toxicology Research, Epidemiology Division, Lucknow, 226001, India; Tehran University of Medical Sciences, Tehran, 14117-13135, Iran; University of Glasgow, MRC/CSO Social & Public Health Sciences Unit, Glasgow G2 3QB, United Kingdom; South African Medical Research Council, Cape Town, 7505, South Africa; CSIR-Indian Institute of Toxicology Research, Epidemiology Division, Lucknow, 226001, India; Jordan University of Science & Technology, Department of Community Medicine, Public Health and Family Medicine, Irbid, 22110, Jordan; Seoul National University College of Medicine, Seoul, 03080, South Korea; Ball State University, Department of Nutrition and Health Science, Muncie, Indiana, 47306, United States; Northeastern University, Department of Health Sciences, Boston, Massachusetts, 02115, United States; Southern University College, Faculty of Chinese Medicine, Johor, 81300, Malaysia; University of Canberra, Bruce, Canberra, Australian Capital Territory, 2617, Australia; National Institute of Health Research & Development, Jakarta, 10560, Indonesia; University of Washington, Institute for Health Metrics and Evaluation, Seattle, Washington, 98121, United States; University of Montreal, Department of Social and Preventive Medicine & Department of Demography & Public Health Research Institute, School of Public Health, Montreal, Quebec, H3C 3J7, Canada; Public Health Foundation of India, Gurgaon, 1222002, India; Södertörn University, Stockholm Centre for Health and Social Change, Huddinge, 14189, Sweden; National Institute for Health Development, Tallinn, 11619, Estonia; University of Washington, Institute for Health Metrics and Evaluation, Seattle, Washington, 98121, United States; University of Washington, Institute for Health Metrics and Evaluation, Seattle, Washington, 98121, United States; University of Washington, Institute for Health Metrics and Evaluation, Seattle, Washington, 98121, United States; University of Melbourne, Melbourne, Victoria, 3051, Australia; National Institute of Public Health, Cuernavaca, 62100, Mexico; University of Washington, Institute for Health Metrics and Evaluation, Seattle, Washington, 98121, United States; Imperial College London, Department of Primary Care & Public Health, London, W6 8RP, United Kingdom; Shiraz University of Medical Sciences, Non-Communicable Diseases Research Center, Shiraz, 71345, Iran; Tehran University of Medical Sciences, Tehran, 14117-13135, Iran; Universidade Federal de Minas Gerais, Gerais, 30130-100, Brazil,; Institute of Genetics and Developmental Biology, Chinese Academy of Sciences, Key State Laboratory of Molecular Developmental Biology, Beijing, 100101, China; Brown University School of Public Health, Providence, Rhode Island, 02912, United States; Mekelle University, Mekelle, 1871, Ethiopia; Robert Koch Institute, Department of Epidemiology and Health Monitoring, Berlin, 650261, Germany; Mekelle University, Mekelle, 1871, Ethiopia; National Center of Cardiology and Internal Disease, Bishkek, Kyrgyzstan; Federal Institute for Population Research Friedrich-Ebert-Allee 5, Wiesbaden, D-65186, Germany; Groote Schuur Hospital and University of Cape Town, Cape Town, South Africa; American University of Beirut, Center for Research on Population and Health, Faculty of Health Sciences, Beirut, Lebanon; Western Sydney University, Centre for Health Research - School of Medicine, Penrith, New South Wales, 2751, Australia; University of Ibadan, Department of Medicine, Ibadan, 200001, Nigeria; University of Melbourne, Melbourne, Victoria, 3051, Australia; University of British Columbia, Vancouver, British Columbia, V5Z 1M9, Canada; Alborz University of Medical Sciences, Department of Community Medicine, Karaj, 3187148455, Iran; Contech School of Public Health, Lahore, 55141, Pakistan; Society for Health and Demographic Surveillance, West Bengal, 731101, India; Yonsei University, Department of Preventative Medicine, Wonju, 220-701, South Korea; Health Science Foundation and Study Center, Kathmandu, Nepal; University of Washington, Institute for Health Metrics and Evaluation, Seattle, Washington, 98121, United States; Maragheh University of Medical Sciences, Department of Public Health, School of Nursing and Midwifery, Maragheh, 5513855731, Iran; Marshall University, University J Edwards School of Medicine, Huntington, West Virginia, 25701, United States; Case Western Reserve University School of Medicine, Comprehensive Cancer Center, Cleveland, Ohio, 44106, United States; University of São Paulo, São Paulo, 05508-000, Brazil; University of KwaZulu-Natal, Durban, South Africa; Marshall University, Department of Public Health, Huntington, West Virginia, 25755, United States; Food and Agriculture Organization, Global Perspective Studies Unit, Rome, Italy; North-West University, South African Medical Research Council; Hypertension in Africa Research Team, Potchefstroom, 2520, South Africa; Tehran University of Medical Sciences, Tehran, 14117-13135, Iran; Shahroud University of Medical Sciences, School of Nursing and Midwifery, Shahroud, 67187187655, Iran; Tehran University of Medical Sciences, Endrocrinology and Metabolism Population Sciences Institute, Tehran, Iran; Korea University, Department of Public Health Sciences, Seoul, 2841, South Korea; Finnish Institute of Occupational Health, Helsinki, FL-00251, Finland; Northumbria University, Faculty of Health and Life Sciences, Newcastle upon Tyne, United Kingdom; Haramaya University, College of Health and Medical Sciences, Harar, 235, Ethiopia; Federal University of Santa Catarina, Florianopolis, 880400-900, Brazil; Northwestern University, Feinberg School of Medicine, Chicago, Illinois, 60611, United States; University of Alabama at Birmingham, Birmingham, Alabama, 35294, United States; Schulich School of Medicine & Dentistry Western University, Department of Epidemiology & Biostatistics, London, Ontario, N6A 5C1, Canada; Public Health Foundation of India, Gurgaon, 1222002, India; University of Valencia, Department of Medicine, Valencia, 46010, Spain; Wollo University, Department of Public Health, Dessie, 1145, Ethiopia; James Cook University, Cairns, Queensland, 4878, Australia; University of Groningen, Groningen, 9700 RB, The Netherlands; Department of Anesthesiology, University of Virginia, Charlottesville, Virginia, 22903, United States; King Fahad Medical City, Department of Anesthesiology, Riyadh, Saudi Arabia; Cleveland Clinic, Outcomes Research Consortium, Cleveland, OH, 44195, United States; Post Graduate Institute of Medical Education and Research, School of Public Health, Chandigarh, 160012, India; University of Calgary, Calgary, Alberta, T2N 1N4, Canada; Fundació Sant Joan de Déu, Univeristat de Barcelona, Barcelona, 8830, Spain; Federal Teaching Hospital, Abakaliki, 23433, Nigeria; University of Warwick, Warwick-Centre for Applied Health Research and Delivery (WCAHRD), Coventry, CV4 7AL, United Kingdom; Umeå University, Dept Public Health and Clinical Medicine, Umea, SE-901 87, Sweden; The UKK Institute for Health Promotion Research, Tampere, 33500, Finland; National Research University Higher School of Economics, Moscow, 109451, Russia; Norwegian Institute of Public Health and University of Bergen, Bergen, 31-5020, Norway; Institute of Population-Based Cancer Research, Department of Research, Cancer Registry of Norway, Oslo, 304, Norway; Karolinska Institutet, Department of Medical Epidemiology and Biostatistics, Sweden, 171 77, Stockholm; University of Tromsø, Department of Community Medicine, Tromsø, 9037, Norway; Folkhälsan Research Center, Genetic Epidemiology Group, Helsinki, 250, Finland; Federal Institute for Population Research Friedrich-Ebert-Allee 5, Wiesbaden, D-65186, Germany; Ghent University, Faculty of Bioscience Engineering, Ghent, 9000, Belgium; Federal Institute for Population Research Friedrich-Ebert-Allee 5, Wiesbaden, D-65186, Germany; Northwestern University, Department of Preventive Medicine, Chicago, Illinois, 60611, United States; Kyoto University School of Public Health, Department of Biostatistics, Kyoto, 606-8501, Japan; Aga Khan University, NCD Research to Policy Unit, Nairobi, 623, Kenya; University Hospital, Setif, 19000, Algeria; University of Washington, Institute for Health Metrics and Evaluation, Seattle, Washington, 98121, United States; University of Washington, Institute for Health Metrics and Evaluation, Seattle, Washington, 98121, United States

## Abstract

**Background:**

While the rising pandemic of obesity has received significant attention in many countries, the effect of this attention on trends and the disease burden of obesity remains uncertain.

**Methods:**

We analyzed data from 67.8 million individuals to assess the trends in obesity and overweight prevalence among children and adults between 1980 and 2015. Using the Global Burden of Disease study data and methods, we also quantified the burden of disease related to high body mass index (BMI), by age, sex, cause, and BMI level in 195 countries between 1990 and 2015.

**Results:**

In 2015, obesity affected 107.7 million (98.7-118.4) children and 603.7 million (588.2- 619.8) adults worldwide. Obesity prevalence has doubled since 1980 in more than 70 countries and continuously increased in most other countries. Although the prevalence of obesity among children has been lower than adults, the rate of increase in childhood obesity in many countries was greater than the rate of increase in adult obesity. High BMI accounted for 4.0 million (2.7- 5.3) deaths globally, nearly 40% of which occurred among non-obese. More than two-thirds of deaths related to high BMI were due to cardiovascular disease. The disease burden of high BMI has increased since 1990; however, the rate of this increase has been attenuated due to decreases in underlying cardiovascular disease death rates.

**Conclusions:**

The rapid increase in prevalence and disease burden of elevated BMI highlights the need for continued focus on surveillance of BMI and identification, implementation, and evaluation of evidence-based interventions to address this problem.

## Background

The prevalence of overweight and obesity is increasing worldwide, amplifying concerns over the health risks associated with this worsening problem.^1^ Epidemiological studies have identified high body mass index (BMI) as a risk factor for an expanding set of chronic diseases including cardiovascular disease,^2,3^ diabetes mellitus, chronic kidney disease,^2^ many cancers,^4^ and an array of musculoskeletal disorders.^5,6^ As the global health community works to develop treatments and prevention policies to address obesity, timely information about levels of high BMI and health impacts at the population level is needed.

In recent years, increasing efforts have been made to assess the trends of BMI within and across nations.^7,8^ Other studies have quantified the potential effects of high BMI on a variety of health outcomes.^9,10^ These efforts, while useful, have not considered the relationship of high BMI with broader socio-economic development; excluded many data sources; focused exclusively on adults; inadequately captured the skewed distribution of BMI; have not captured emerging evidence on additional outcomes; and have not assessed the effect of epidemiologic and demographic transition on disease burden. The optimal level of BMI for minimum mortality risk has also been questioned.^11,12^

To address these gaps in knowledge, we systematically evaluated the trends in the prevalence of overweight and obesity as well as the patterns of deaths and disability-adjusted life years (DALYs) related to high BMI by age and sex for 195 countries. This analysis supersedes all previous Global Burden of Disease study (GBD) results for high BMI by comprehensively reanalyzing all data from 1990 through 2015 using consistent methods and definitions.

## Methods

We systematically estimated the prevalence of overweight and obesity among children (<20 years of age) and adults between 1980 and 2015. Using the GBD comparative risk assessment approach, we also quantified the burden of disease related to high BMI between 1990 and 2015. The main inputs to our analysis included the distribution of BMI by age, sex, country, and year; the effect size of the change in BMI on disease endpoints; the BMI level associated with the lowest risk from all causes; and disease-specific mortality and morbidity by country, age, sex, and year.

### Assessment of the Global Distribution of Body Mass Index

We systematically searched Medline for studies providing nationally or sub-nationally representative estimates of BMI, overweight, or obesity among children or adults. Studies were included if using standard cutoff points of BMI to define overweight (BMI: 25-29 kg/m^2^) and obesity (BMI≥30 kg/m^2^) among adults or International Obesity Task Force (IOTF) standard to define overweight and obesity among children. The search terms, selection criteria, and flow diagrams of screening are provided in the Appendix. In addition, we searched the Global Health Data Exchange (http://ghdx.healthdata.org) for multi-country survey programs, national surveys, and longitudinal studies providing self-report or measured data on height and weight for children or adults. We identified 1276 unique data sources (855 measured, 421 self-report) from 176 countries providing data on BMI; 1333 unique sources (802 measured, 531 selfreport) from 176 countries for overweight; and 1514 unique sources (713 measured, 801 self-report) from 174 countries for obesity among adults. We also identified 1211 unique sources (800 measured, 411 self-report) from 173 countries for BMI, 1236 unique sources (832 measured, 404 self-report) from 174 countries for overweight, and 1437 unique sources (928 measured, 509 self-report) from 175 countries for obesity among children. Using mixed effects linear regression models, we separately estimated and corrected for self-report bias among men and women in each GBD region and age group (Appendix). We characterized the age and sex patterns for BMI, overweight, and obesity and applied these patterns to split aggregated report data into five-year age groups by sex (Appendix).

We used spatiotemporal Gaussian process regression (ST-GPR) to estimate the mean prevalence of overweight and obesity.^13^ To improve our estimates in data-sparse countries, we tested a wide range of covariates with plausible relationships to obesity and overweight. We selected three country-level covariates with best fit and coefficients in the expected direction – as used in other studies.^8^ These included 10-year lag distributed energy intake per capita, the absolute latitude of the country, and the proportion of people living in urban areas. To estimate mean BMI, we first used a mixed effects linear regression to characterize the relationship between BMI, overweight, and obesity in sources containing information on all three measures. We applied the coefficients of this regression to the prevalence of overweight and of obesity generated through ST-GPR to estimate the mean BMI for each country, age, sex, and year. Of 195 countries and territories included in the present study, only 8 had no data for any age or sex group: Antigua and Barbuda, Bermuda, Brunei, Northern Mariana Islands, Saint Vincent and the Grenadines, The Bahamas, Turkmenistan, and Venezuela. The estimates in these countries were constructed purely from the covariates used in estimation of the linear model and the weighted and smoothed residuals from data of neighboring countries.

We used a novel method to characterize the distribution of BMI at the population level. Prior studies have shown that the distribution of BMI becomes skewed as the mean BMI increases, indicating the need for a flexible distribution that captures both symmetric and asymmetric patterns of BMI.^14^ To identify the appropriate distribution, we examined how various distributions (i.e., lognormal, gamma, inverse Gaussian and beta) approximated the distribution of actual data from national surveys in six countries; the best fit was provided by the beta distribution.^14^ To compute the parameters of a beta distribution for BMI, we used mean BMI, overweight prevalence, and obesity prevalence in each country, age, sex, and year. Details of this approach have been described elsewhere.^14^

### Effect of High Body Mass Index on Health Outcomes

We used Bradford Hill's criteria for causation and the World Cancer Research Fund evidence grading criteria to systematically evaluate epidemiologic evidence supporting the causal relationship between high BMI and various disease endpoints among adults (>25 years of age).^15^ We found convincing or probable evidence for 20 health outcomes including ischemic heart disease, ischemic stroke, hemorrhagic stroke, hypertensive heart disease, diabetes mellitus, chronic kidney disease, esophageal cancer, colon and rectum cancer, liver cancer, gallbladder and biliary tract cancer, pancreatic cancer, breast cancer, uterine cancer, ovarian cancer, kidney cancer, thyroid cancer, leukemia, knee osteoarthritis, hip osteoarthritis, and low back pain (Table S1). For each outcome, we obtained the relative risk from a dose-response meta-analysis of prospective observational studies (Table S2). In the case of ischemic heart disease, ischemic stroke, hemorrhagic stroke, hypertensive heart disease, and diabetes mellitus, we estimated the relative risk for change in five units of BMI in five-year age groups from pooled analyses of prospective cohort studies. For breast cancer, we calculated GBD region-specific relative risk for pre-menopausal and postmenopausal women because of evidence that overweight and obesity has a protective effect for breast cancer in premenopausal women in all countries except for the Asia-Pacific regions (High income Asia Pacific, East Asia, South East Asia and Oceania)^16,17^ while a positive association between high BMI and postmenopausal breast cancer has been observed worldwide.^17^

### Optimal Level of Body Mass Index

We determined the level of BMI associated with the lowest overall level of risk based on the findings of the most recent pooled analysis of prospective observational studies.^11^ To address the limitations of previous publications on this topic, including residual confounding among smokers and reverse causation due to pre-existing chronic diseases,^12^ the analysis was restricted to never-smokers without chronic diseases who survived five years after recruitment. The lowest rate of all-cause mortality was observed for a BMI level of 20-25 kg/m^2^

### Statistical Analysis

To quantify the burden of disease related to high BMI for each endpoint, we calculated the population attributable fraction (PAF) by country, age, sex, and year (Appendix). We computed deaths and DALYs related to high BMI for each country, age, sex, year, and cause by multiplying the PAF by the total deaths or DALYs estimated in GBD 2015 for that country, age, sex, year, and cause. The total disease burden of high BMI was calculated as the sum of disease-specific burden. To understand where in the distribution of BMI most burden occurs, we estimated PAFs for different levels of BMI (20-24 kg/m^2^; 25-29 kg/m^2^; and ≥ 30 kg/m^2^) and different groups of disease endpoints (cardiovascular disease, diabetes mellitus, chronic kidney disease, neoplasms, and musculoskeletal disorders).

We decomposed the change in death and DALY rates attributed to high BMI between population growth, population age structure, risk exposure to high BMI, and risk-deleted death and DALY rates using methods developed by Das Gupta^18^. Risk-deleted rates are the burden of disease in the absence of the risk factor.

We computed 95% uncertainty intervals for all results using Monte Carlo simulations, keeping 1000 draws of each quantity of interest to propagate uncertainty into final estimates. The model included uncertainty from examination surveys; the relative risks for each outcome from the pooled analysis or meta-analysis of cohorts; the optimal level of BMI; and the deaths and DALYs estimated for each country, age, sex, year, and outcome from GBD 2015. Following methods outlined in the GBD 2015 study, we used a Socio-demographic Index (SDI) – a summary measure of lag-distributed income per capita, average educational attainment over the age of 15 years, and total fertility rate – to position countries on the development continuum .^15^ A list of countries with their SDI level in 2015 is provided in Table S3.

## Results

### Prevalence of obesity (1980-2015)

#### Global level

**Figure 1:**
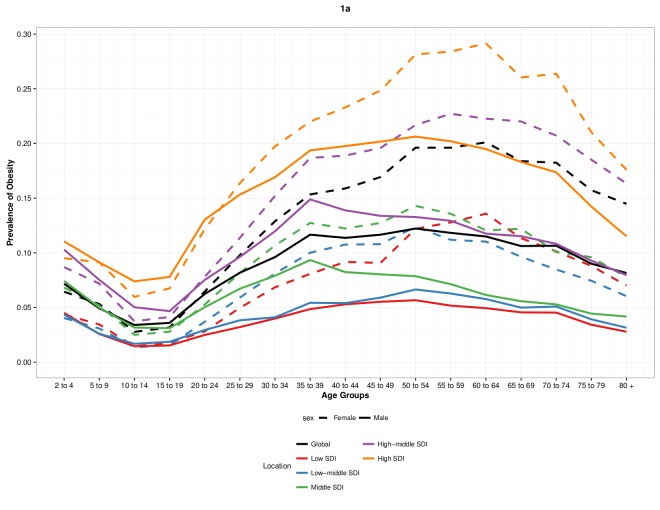

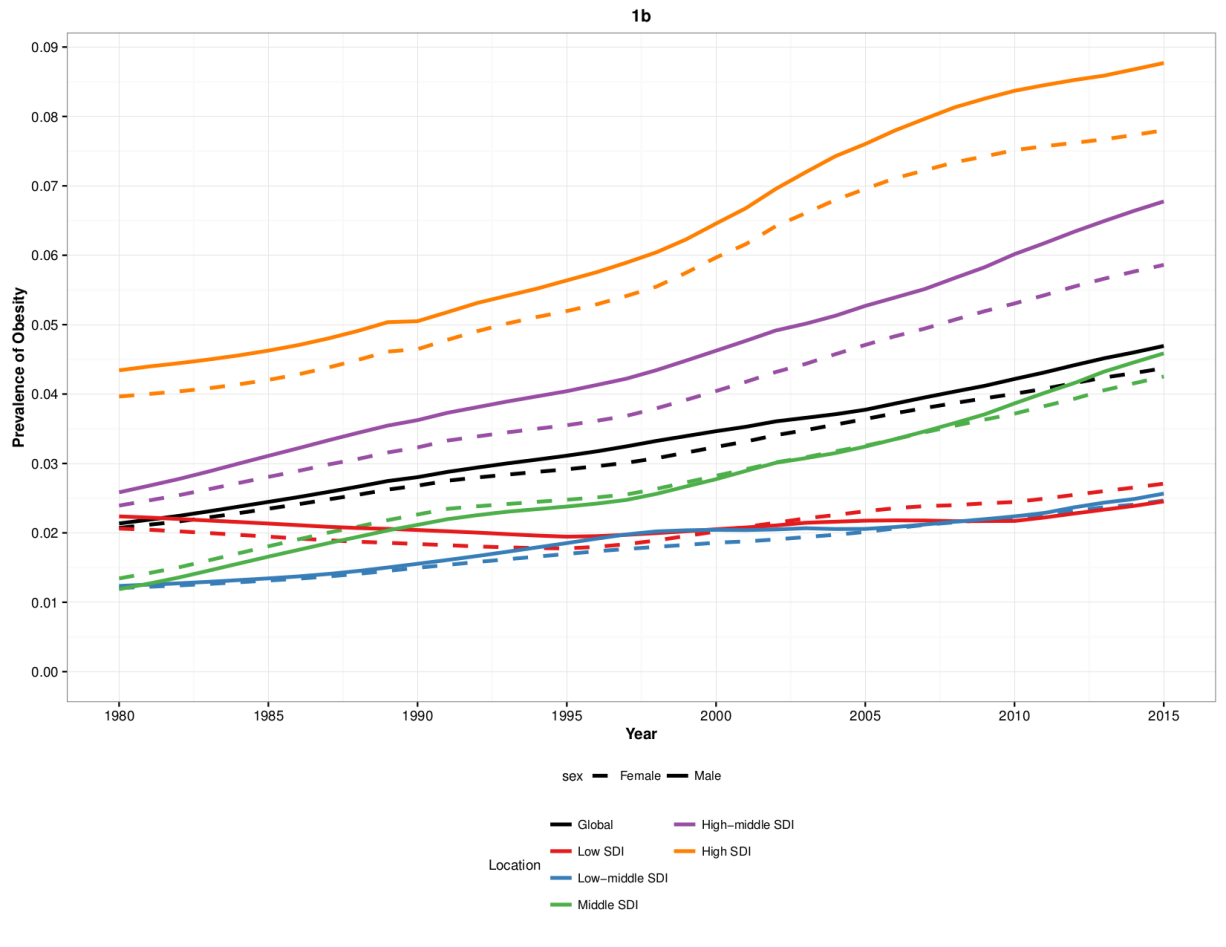

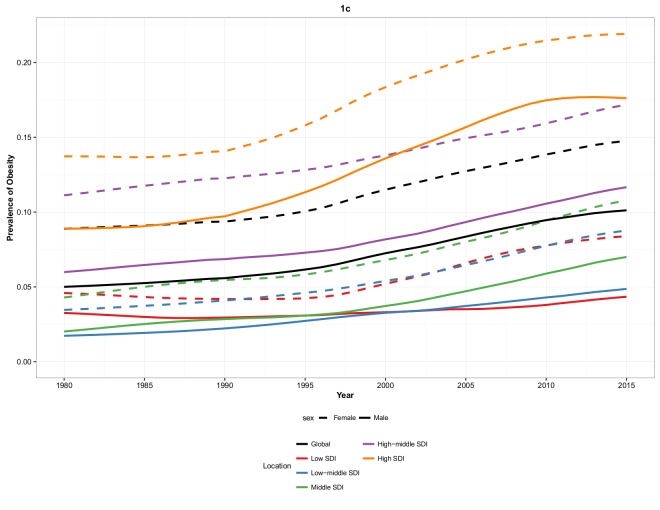
Global prevalence of obesity in 2015 by age, sex, and quintile of Socio−demographic Index (a) and trends in age−standardized prevalence of obesity among children (b) adults (c).

In 2015, 107.7 million (98.7-118.4 million) children and 603.7 million (588.2-619.8 million) adults were obese worldwide. The overall prevalence of obesity for children and adults was 5.0% and 12.0% respectively. Among adults, the prevalence of obesity was generally higher for women than for men in all age brackets (Figure 1). The peak in prevalence of obesity was observed at age 60 to 64 for women and at age 50 to 54 for men. Rates of increase between 1980 and 2015 were not significantly different between women and men in any age bracket; for both, rates of increase were highest in early adulthood. Among children, the prevalence of obesity in 2015 decreased with age bracket until age 14 and then increased; no sex differences were observed in obesity prevalence before age 20. Between 1980 and 2015, rates of increase in global childhood obesity were equal for boys and girls in all age brackets.

#### By level of Socio-demographic Index

At all levels of SDI and for all age groups, the prevalence of obesity was generally higher for women than for men in 2015 (Figure 1); the prevalence of obesity in adults was highest for women aged 60 to 64 in high SDI countries. In general, the prevalence of obesity for both women and men increased with SDI across all age groups. An exception was the prevalence of obesity in women in low SDI geographies—after age 55 to 59, prevalence was higher for women in low SDI geographies than for women of equivalent age in low-middle SDI (Figure 1). Obesity prevalence increased fastest over the period 1980 to 2015 for men age 25 to 29 in lowmiddle SDI countries, from 11.1% (8.5-14.7%) in 1980 to 38.3% (30.7-48.1%) in 2015. Obesity prevalence increased by 2.4 fold in both men and women of all ages in low-middle and middle SDI countries between 1980 and 2015.

Overall, prevalence of obesity for children was greater at higher SDI (Figure 1). At most levels of SDI, prevalence of obesity for children was lowest for both boys and girls between ages 10 to 14. In high and high middle SDI geographies alone, prevalence was generally greater for boys than girls, although this difference reversed beginning with late adolescence (Figure 1). A significant increase was observed in the prevalence of obesity between 1980 and 2015 at low SDI for both girls and boys 20.0% (5.5-35.3%). The highest rates of increase between 1980 and 2015 were observed in middle SDI geographies for both girls and boys.

#### National level

**Figure 2:**
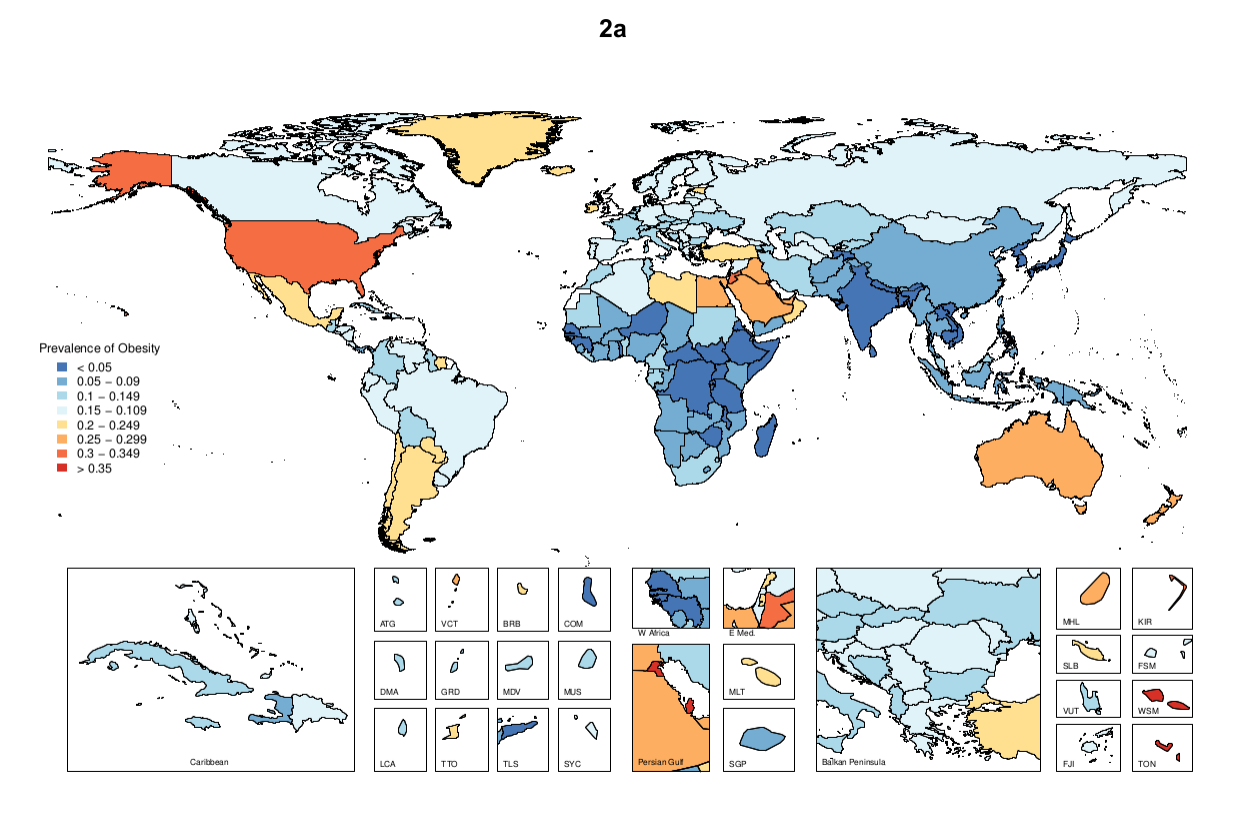

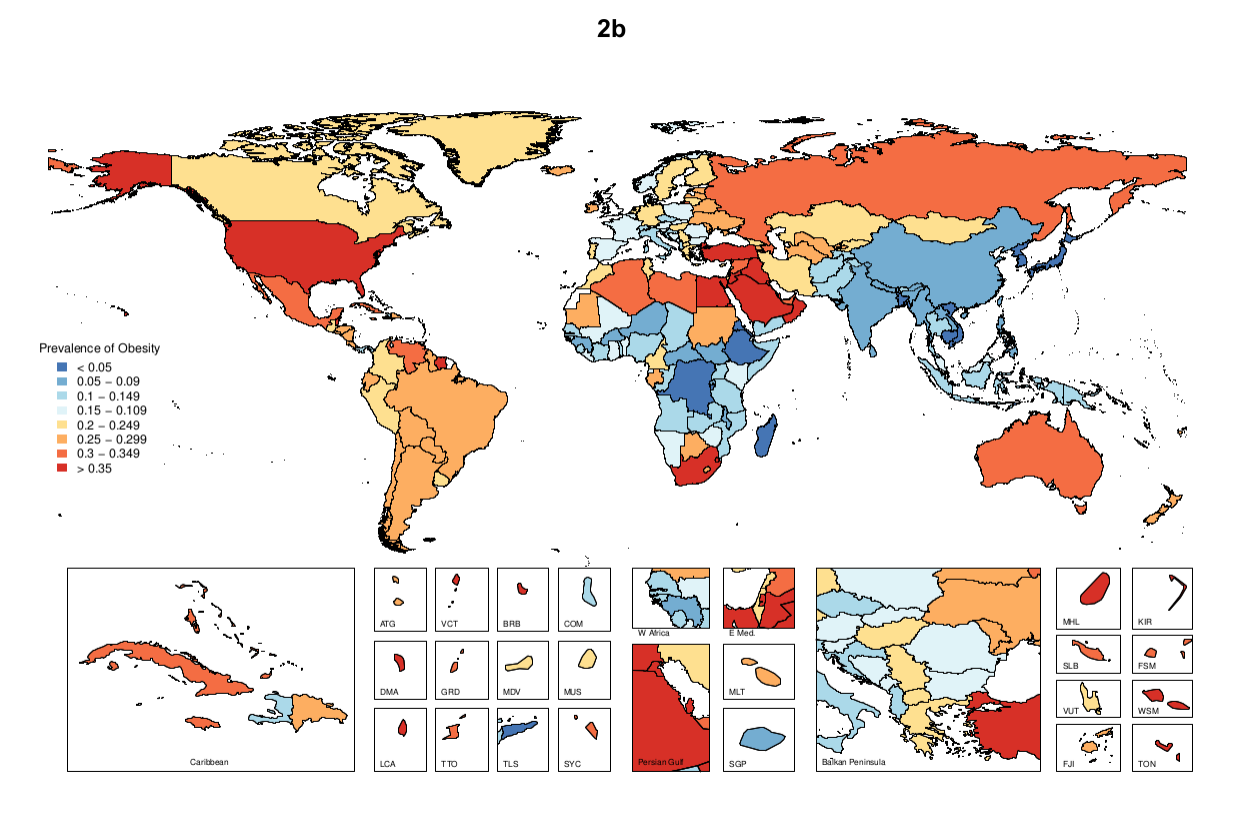

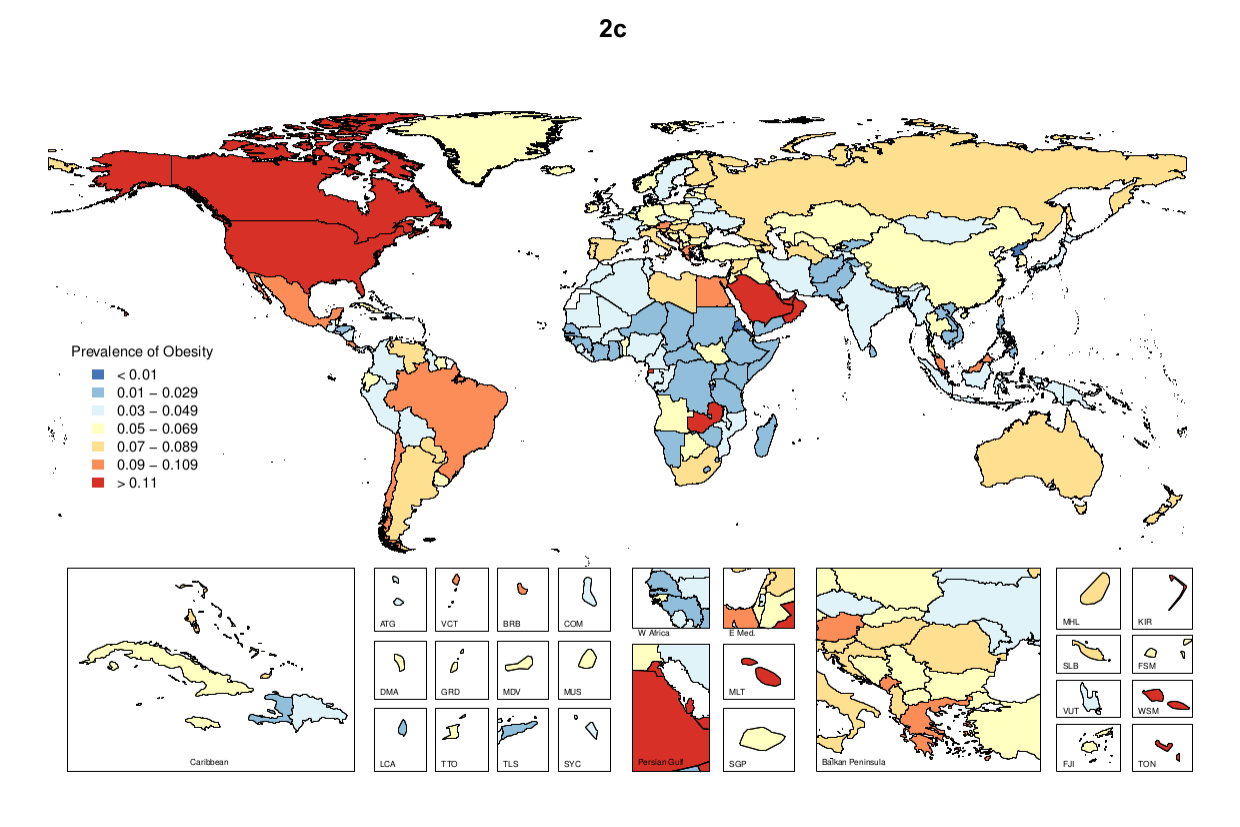

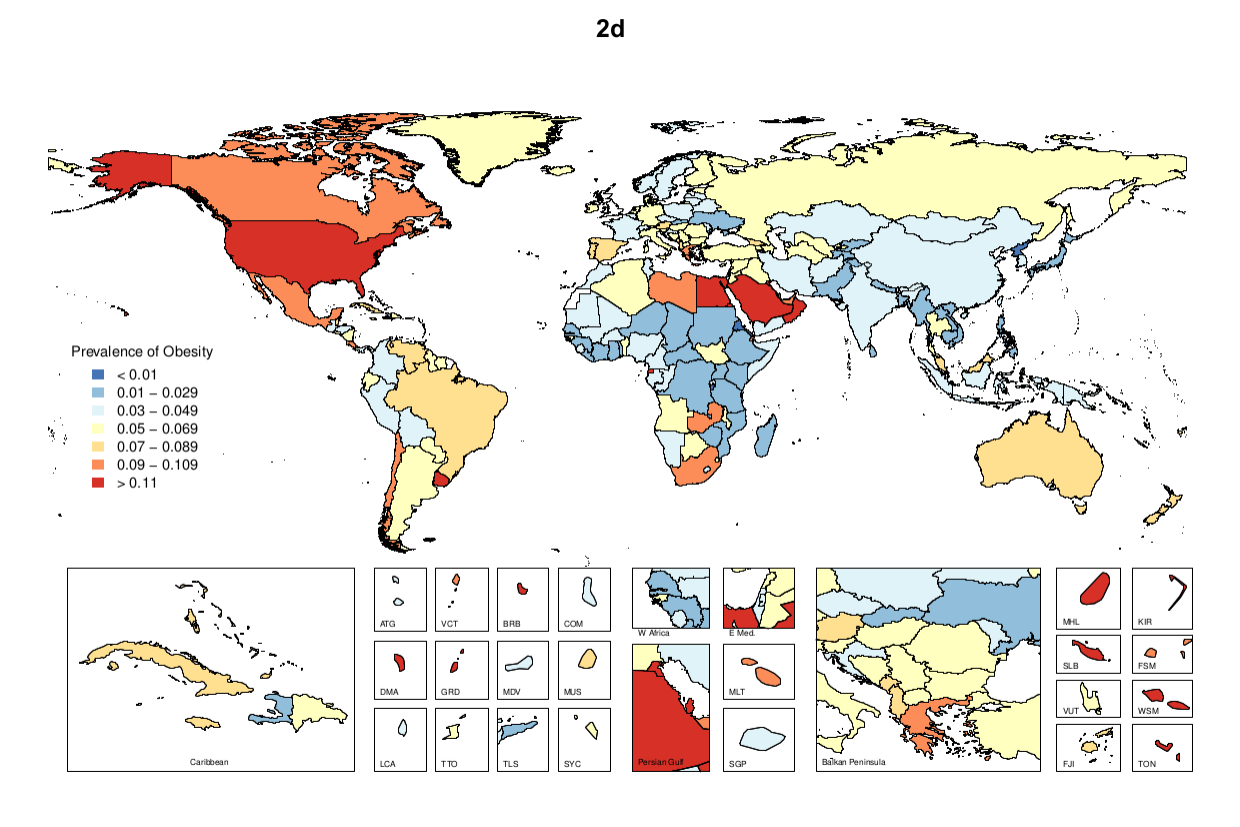
Age−standardized national prevalence of obesity among adult (males [a], females [b]) and children (males [c], females [d]) in 2015.

The estimated age-standardized prevalence of obesity and overweight among children and adults for all 195 countries and territories are provided in the Table S3 here we highlight the findings related to obesity in the most populous countries (Figure 2). Amongst the 20 most populous countries, the highest level of adult obesity in 2015 was observed in Egypt at 34.9% (32.4-37.3%) and the highest level of childhood obesity was in United States at 12.7% (12.0-13.4%); prevalence was lowest for adults in Vietnam at 1.6% (1.3-2.1%) and for children in Bangladesh at 1.2% (0.8-1.9%). The prevalence of obesity doubled or increased more than 2- fold in 13 of these countries between 1980 and 2015; only the Democratic Republic of the Congo showed no increase (Figure S1 and Figure S2). China and India had the highest number of obese children while the United States and the Philippines had the highest number of obese adults in 2015.

### Burden of disease related to high BMI (1990-2015)

#### Global level

**Figure 3:**
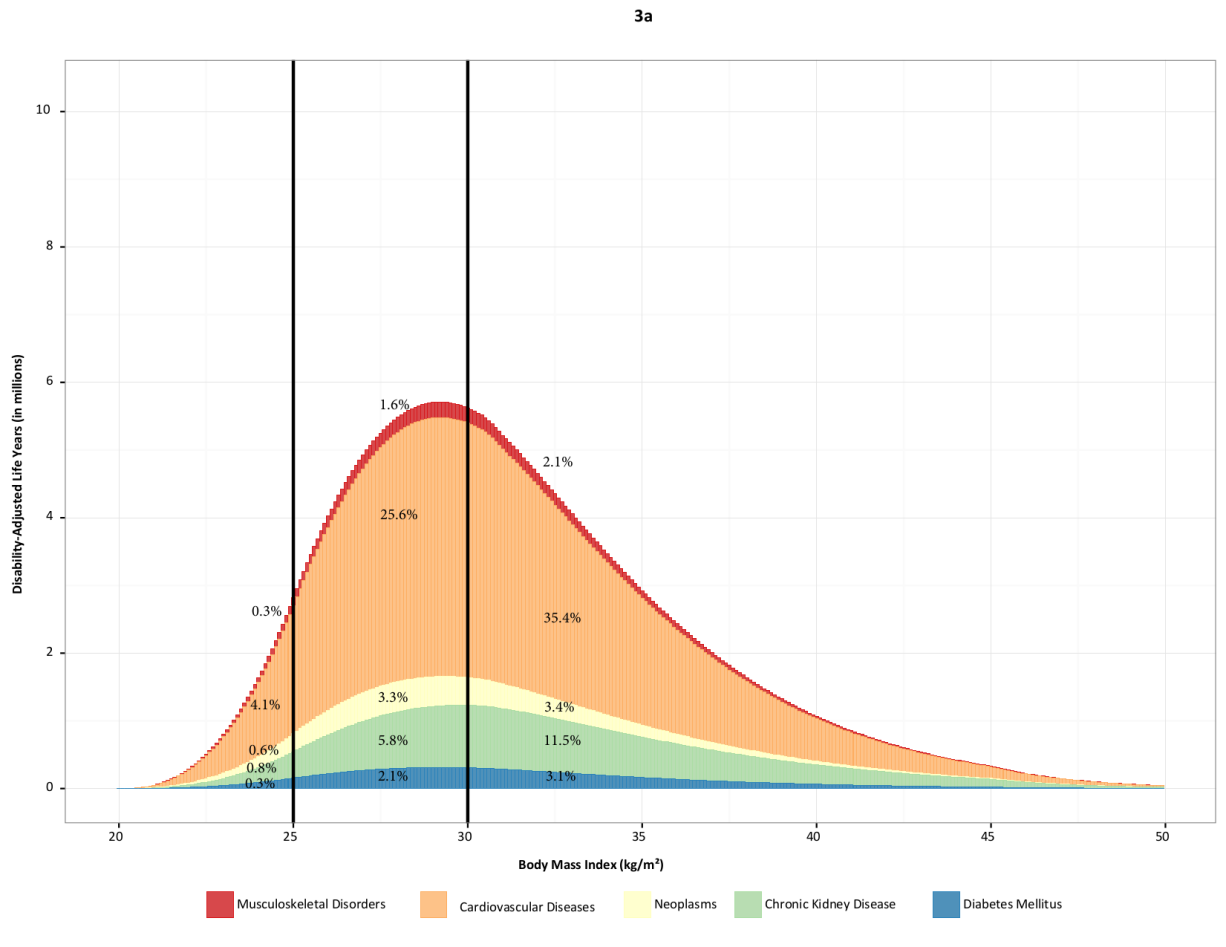

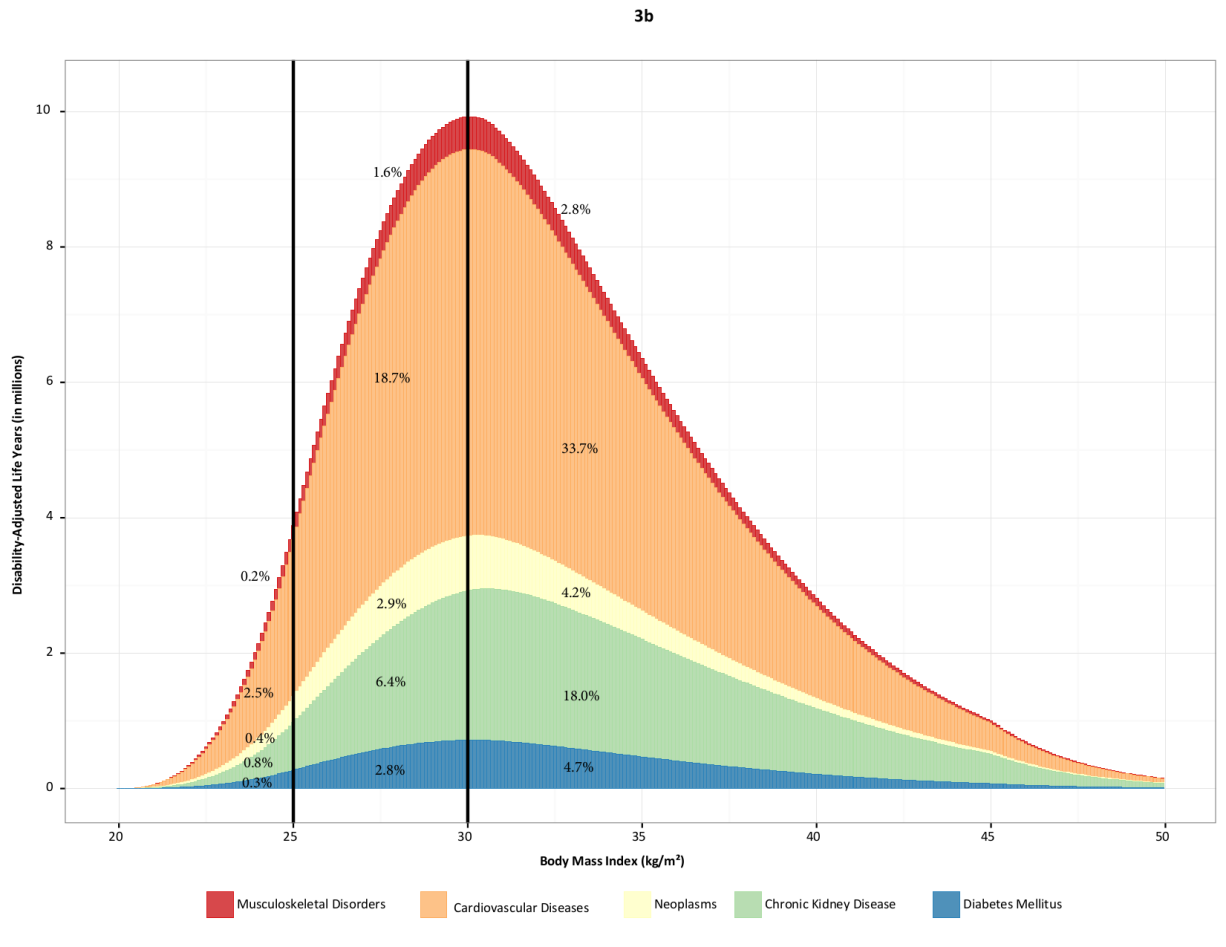

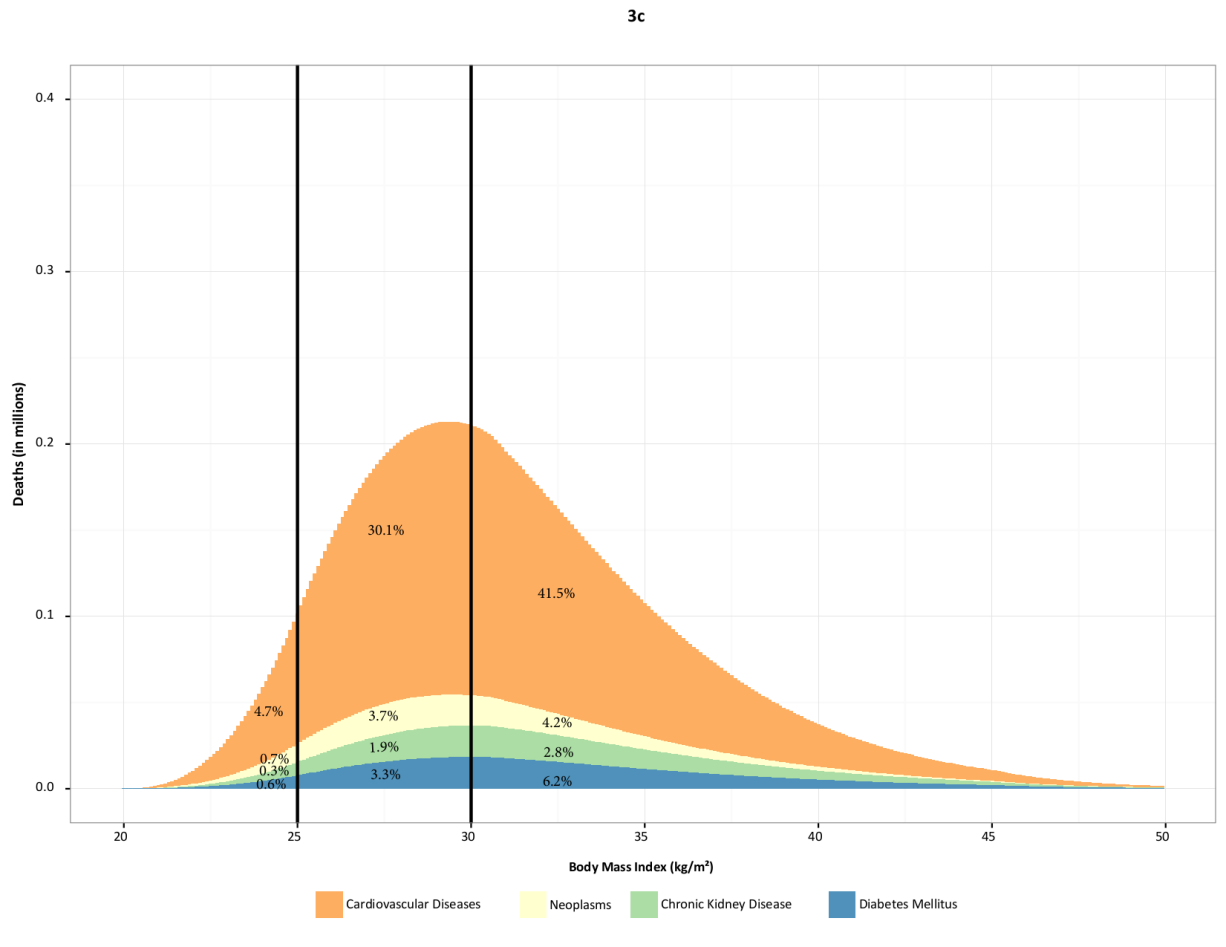

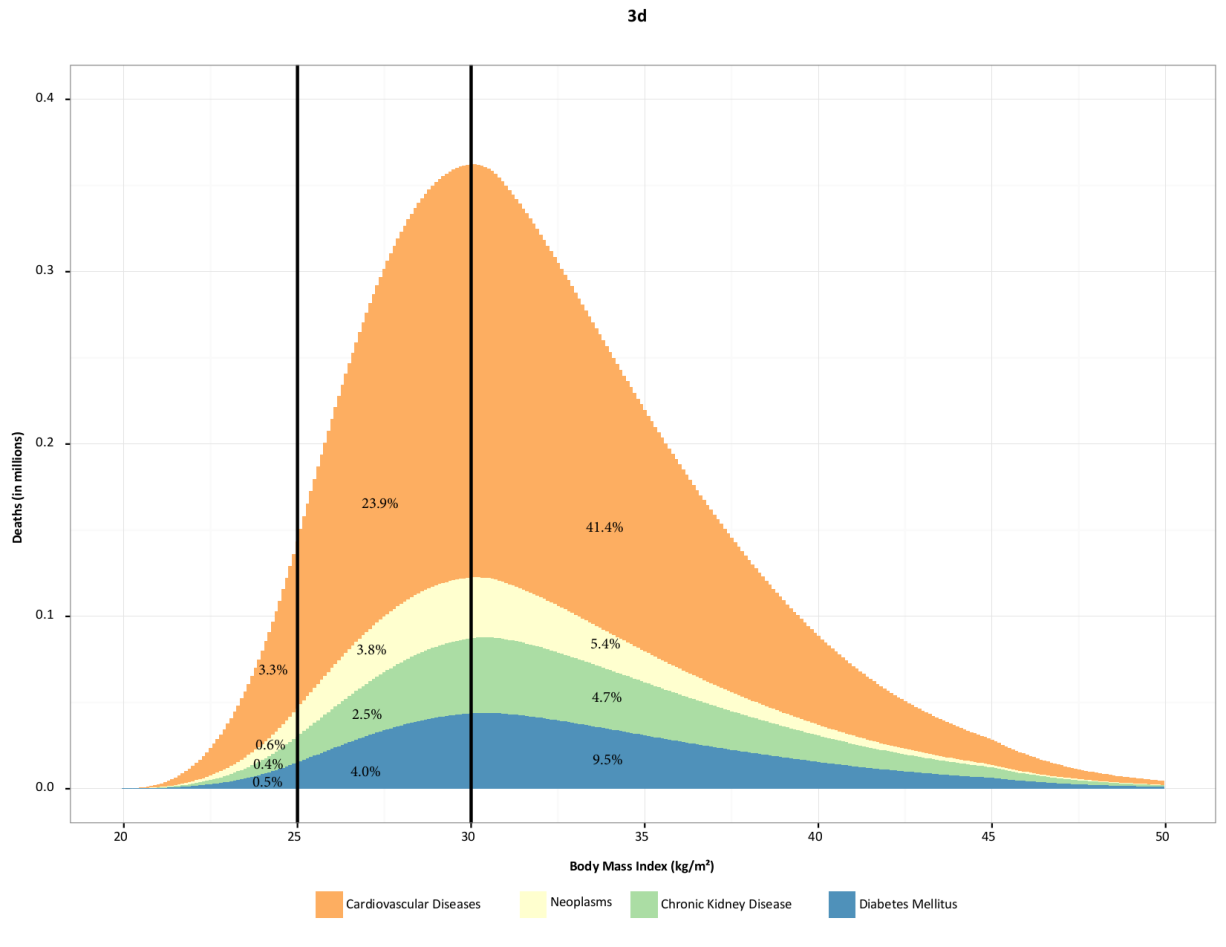
Global disability-adjusted life years (in millions) related to high body mass index (BMI) among adults by cause and the level of BMI in 1990 (a) and 2015 (b) and global deaths (in millions) related to high BMI in 1990 (c) and 2015 (d).

In 2015, excess weight contributed to 4.0 million (2.7-5.3 million) deaths (7.2% [4.9-9.4%] of allcause deaths) and 120 million (84-158 million) DALYs (4.9% [3.5-6.4%] of all-cause DALYs) among adults globally. Nearly 39% of deaths and 36% of DALYs related to high BMI occurred in those with a BMI <30 kg/m2 (Figure 3). Cardiovascular disease was the leading cause of deaths and DALYs related to high BMI, accounting for 2.7 million (1.8-3.7 million) deaths and 66.3 million (45.3-88.5 million) DALYs (Table S4). Globally, 41% of BMI-related deaths and 34% of BMI-related DALYs were due to cardiovascular disease among obese people (BMI>30 kg/m2). Diabetes was the second leading cause of BMI-related deaths in 2015, contributing to 0.9 million (0.6-1.1 million) deaths and 39.1 million (28.1-51.1 million) DALYs; 9.5% and 4.5% of all BMI-related deaths were due to diabetes at BMI >30 and <30 respectively. Chronic kidney disease was the second leading cause of BMI-related DALYs in 2015; 18.0% of DALYs occurred at BMI >30 and 7.3% at BMI <30. Chronic kidney disease and neoplasms each accounted for less than 10% of all BMI-related deaths in 2015, while neoplasms, diabetes, and musculoskeletal disorders each contributed less than 10% of BMI-related DALYs (Figure 3). High BMI also accounted for 28.6 million (17.8-41.4 million) years lived with disability (YLD) (3.6% [2.7-4.6%] of all-cause YLDs) globally. Diabetes was the leading cause of YLDs related to BMI (19.3 million [12.2-27.4 million]) followed by musculoskeletal disorders (5.7 million [3.4-8.8 million]) and cardiovascular disease (3.3 million [2.0-4.9 million]).

**Figure 4:**
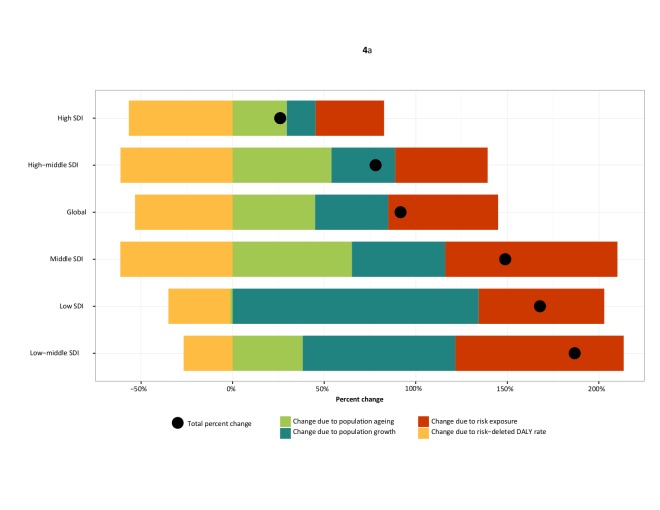

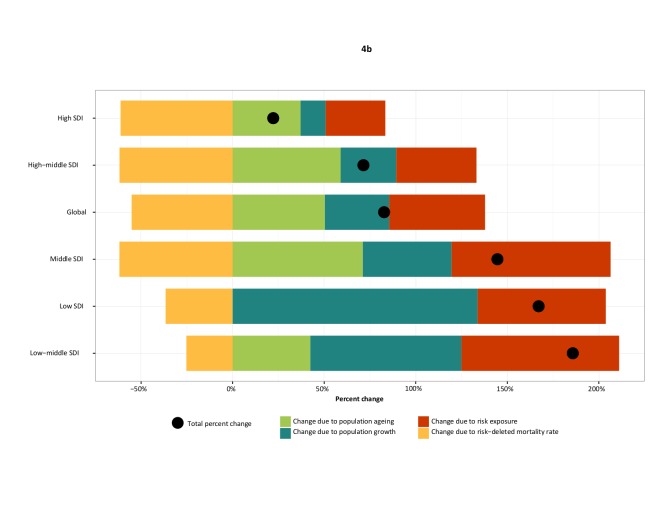
Decomposition of percent changes in all−cause disability-adjusted life years (DALYs) (a) and deaths(b) related to high body mass index from 1990 to 2015 due to population growth, population ageing, risk exposure and the underlying rates of DALYs and deaths by quintiles of Socio−demographic Index. *Locations are reported in order of percent change in the number of related DALYs from 1990 to 2015. DALYs=disability−adjusted life−years.*

The global mortality related to high BMI increased by 28.3% from 41.9 per 100,000 in 1990 to 53.7 per 100,000 in 2015, although age-standardized mortality rates did not significantly change in this period (64.0 [41.7-89.7] per 100,000 in 1990 and 60.2 [43.1-81.5]) per 100,000 in 2015). Similarly, BMI-related DALYs increased by 35.8% between 1990 and 2015, from 1200 per 100,000 to 1630 per 100,000 while no significant change was observed in age-standardized rates. Figure 4 illustrates that, globally, percent change in BMI-related deaths and DALYs due to risk-deleted mortality rates were matched by increases from other factors. Of the disease endpoints considered in this study, decreases in the risk-deleted mortality rate for cardiovascular disease contributed the most to this pattern. Change due to risk exposure and population aging were roughly equal in terms of their contribution to both percent change of related deaths and DALYs globally between from 1990 to 2015.

#### By level of Socio-demographic Index

Age-standardized rates of both BMI-related deaths and DALYs were greatest in high-middle SDI (deaths, 60.1 [47.1-91.6] and DALYs, 1890 [1330-2460] per 100,000) and lowest at high SDI (deaths, 52.6 [38.73-67.9] and DALYs 1530 [1160-1920] per 100,000) in 2015. The all-ages rate of BMI-related deaths increased between 1990 and 2015 at all SDI levels, with a peak for high SDI in the year 2005 at 2359 (1749-2997) per 100,000. Age-standardized rates of death at high and high-middle SDI decreased between 1990 and 2015; in the lowest quintiles, agestandardized BMI-related deaths increased. With increasing levels of SDI, the contribution of risk-deleted mortality rate to the percent change in all-cause related deaths increased while the contribution of population growth to percent change in mortality decreased (Figure 4). The contribution of risk exposure to percent change in BMI-related deaths was also generally inversely related to SDI. Patterns in the decomposition of sources of change for BMI-related DALYs were parallel to those observed for mortality. In disease-specific decomposition, riskdeleted mortality and DALY rates showed a declining trend for most causes across all levels of SDI (Table S5). The largest decrease in risk-deleted deaths and DALYs were observed for cardiovascular disease while cancers and musculoskeletal disorders showed the least decline respectively.

#### National level

Among the 20 most populous countries, the highest burden of related deaths and DALYs was observed in Russia; the lowest rate of related deaths and DALYs occurred in the Democratic Republic of the Congo (Figure S3). Between 1990 and 2015, the greatest percent change in related deaths and DALYs occurred in Russia at 42.2% (31.9-55.9%) and 26.6% (19.6-36.1%) respectively while rates of change were lowest in Japan (deaths, 20.5% [12.7-28.2%]; DALYs, 1.0% [-3.8-5.6%]) (Table S6).

## Discussion

Our systematic evaluation demonstrates that excess body weight is a major risk factor for mortality and morbidity, accounting for 4.0 million deaths and 120 million DALYs worldwide. Nearly 70 percent of deaths related to high BMI are due to cardiovascular disease and over 60 percent of those deaths occurs among the obese. The prevalence of obesity has increased over the past three decades, and at a faster pace than the related burden. Both the trend and magnitude of the BMI-related disease burden, however, vary widely across countries and at different levels of socio-demographic status.

Among leading risks for health assessed in the GBD 2015, high BMI continues to have one of the highest rates of increase. Across levels of development, the prevalence of obesity has increased over recent decades indicating the problem is not simply a function of income or wealth.^15^ Changes in the food environment and food systems are likely to be major drivers.^19^ Increased availability, accessibility, and affordability of energy-dense foods, along with intense marketing of such foods could sufficiently explain excess energy intake and weight gain in different populations.^19^ The reduced opportunities for physical activity that have followed urbanization and other changes in the built environment have also been considered as potential drivers; however, these changes generally preceded the global increase in obesity and are less likely to be major contributors.^19^

Over the past decade, a range of interventions in the food environment and the food system have been proposed in order to reduce obesity.^20^ These include restricting the advertisement of unhealthy foods to children, improving school meals, using taxation to reduce consumption of unhealthy foods and subsidies to increase intake of healthy foods, and using supply-chain incentives to increase production of healthy foods.^20^ However, the effectiveness, feasibility of widespread implementation, and sustainability of these interventions need to be evaluated in various settings. In recent years, some countries have started to implement some of these policies^1^ but no major population success has yet been demonstrated. Many of the countries that have experienced the highest increase in the prevalence of obesity are low or middle SDI countries that simultaneously suffer from high rates of other forms of malnutrition, creating additional challenges. These countries generally have limited financial resources for nutrition programs and mostly rely on external donors whose programs often preferentially target undernutrition; consequently, food security frequently takes precedence over obesity in these countries. In a review of the nutrition policies in countries with a double burden of undernutrition and obesity, only one country reported funding partners available to address both aspects of malnutrition.^21^ In 2013, the World Health Organization (WHO) called for zero increase in the prevalence of obesity among adults and zero increase in the prevalence of overweight among children.^22^ However, given the current pace of increase and the existing challenges in implementing food policies, achieving this goal appears unlikely in the near future. While policy interventions targeting behavioral change, the food environment, and food systems might be successful in prevention of further weight gain or even achieving modest weight loss over the long term, limited improvement can be expected at the individual level in the short term. Given the rising burden of obesity and extreme obesity, health care professionals need to play a more active role in both the promotion of weight loss and controlling the complications of obesity.^1,23^ Training for health care professionals on evidencebased options for treatment of obese adults and children (e.g., behavioral change techniques, medications, and bariatric surgery) is necessary – although limited options are available for treatment of childhood obesity. Such treatment needs to be selected based on the intensity of obesity and their cost-effectives and will only be sustained if accompanied by supporting policies targeting the food environment and food system.

Our study found a greater rate of increase in exposure to high BMI than for the related disease burden. This difference is mainly driven by the decline in risk-deleted mortality rates, particularly for cardiovascular disease; factors such as improved treatment or changes in other risks have resulted in cardiovascular disease declines despite increases in BMI. This observation has important implications for attempts to reduce the disease burden of high BMI at the population level. Existing evidence-based policies, even if fully implemented, are unlikely to rapidly reduce the prevalence of obesity. Clinical interventions, however, have proved effective in controlling high systolic blood pressure, cholesterol, and high fasting plasma glucose (the major risk factors for cardiovascular disease) and are thus implicated in the decrease in underlying disease burden.^24^ Expanded use of such interventions among obese people could effectively reduce the disease burden of high BMI. This approach will also mitigate the effect of high BMI on cardiovascular disease by removing the effect of BMI mediated through these risk factors. A recent pooled cohort analysis including 1.8 million participants found that nearly half of excess risk for ischemic heart disease related to high BMI and more than 75% of the excess risk for stroke was mediated through the combination of raised blood pressure, total serum cholesterol and fasting plasma glucose.^25^ Together, these findings suggest that clinical interventions to reduce the underlying rate of cardiovascular disease could substantively reduce the burden of disease related to high BMI, although maintaining a normal body weight remains necessary to achieve full benefit.^23^

Globally, 40% of deaths and 38% of the DALYs related to high BMI occurred among non-obese individuals. While some studies have argued that overweight is associated with lower risk of allcause mortality compared to a normal range of 18-25 kg/m^2^,^12^ recent evidence from metaanalysis16 and pooled analysis11 of prospective observational studies found a continuous increase in the risk of death for BMI above 25 kg/m^2^. These recent publications are particularly notable because they addressed major sources of bias in prior studies (i.e. residual confounding by smoking and reverse causation due to pre-existing chronic disease) by restricting the analysis to never smokers without chronic diseases. Additionally, the pooled cohort analysis controlled for the same set of covariates, provided cause-specific relative risks, and evaluated the relationship between BMI and mortality across different regions. The balance of evidence thus supports our minimum risk level BMI of 20-25 kg/m^2^. Given this, our study suggests that nonobese individuals carry a large proportion of the total burden that would be missed by focusing solely on the obese individuals. At the same time, to date, there remains insufficient evidence to support the argument that the optimal level of BMI should vary geographically or by ethnicity^11^ because of differences in the relationship between BMI and body fat distribution. We found that 4% of the DALYs related to high BMI were from musculoskeletal disorders. Although high BMI is a major risk factor contributing to years lived with disability globally, and the economic costs associated with treatment are substantial,^26^ these non-fatal but debilitating health outcomes have received comparatively little policy attention. Similar to cardiovascular outcomes, weight loss is beneficial in prevention and treatment of musculoskeletal pain. In the Framingham Study, a decrease in BMI of 2 or more units in the decade prior to evaluation was found to decrease the odds of developing knee osteoarthritis by more than 50%.^27^ A combination of modest weight loss and moderate exercise provides better overall improvement in musculoskeletal pain than either intervention alone;^28^ however, surgical interventions may be most effective for the morbidly obese.^29^

Our systematic evaluation of prospective observational studies found sufficient evidence supporting a causal relationship between high BMI and cancers of the esophagus, colon and rectum, liver, gallbladder and biliary tract, pancreas, breast, uterus, ovary, kidney, thyroid, and leukemia. A recent review by the International Agency for Research on Cancer (IARC) comes to largely similar conclusions, but with some notable differences.^4^ For example, although the IARC report did not include leukemia, we included this outcome based on a systematic review and meta-analysis of 21 prospective cohort studies30 which found a significant association between obesity (BMI>30) and incidence and mortality from leukemia. Additionally, while the IARC report acknowledged consistent inverse associations between BMI and the risk of premenopausal breast cancer, inconsistent findings from studies evaluating the effect of waist circumference or body-weight gain resulted in its exclusion. However, since high BMI was the exposure of interest in our analysis, we included the protective effect of high BMI on breast cancer in pre-menopausal women. We did not evaluate the effect of high BMI on gastric cancer (cardia) and meningioma due to lack of sufficient data to separately estimate the incidence and mortality of these cancers at the population level.

Our study has several important strengths. We have addressed the major limitations of prior studies by including more data sources and quantifying the prevalence of obesity among children. We also systematically evaluated the strength of evidence for the causal relationship between high BMI and health outcomes and included all BMI-outcome pairs for which sufficient evidence on causal relationship was available. We used a beta distribution to characterize the distribution of BMI at the population level, which captures the fraction of the population with high BMI more accurately than other distributions.^14^ We used the best available evidence to determine the optimal level of BMI. We quantified the trends and disease burden of high BMI across levels of development and estimated the contribution of demographic transition and epidemiologic transition to changes in BMI-related burden.

Potential limitations that may result in over- or underestimation of the prevalence of obesity or disease burden from high BMI should also be considered. We used both self-report and measured height and weight data and corrected the self-reported data based on measured data at each age, sex, and country unit. To apply a consistent definition for childhood overweight and obesity across sources, we used the International Obesity Task Force definition and excluded studies using the World Health Organization definition. We did not propagate the uncertainty in the age pattern and sex pattern used to split the data as they seemed to have small effect. We did not incorporate the uncertainty of the regression coefficients in our analysis. Early data were particularly sparse for many locations and estimates were based on country level covariates and regional data. We did not identify a consistent pattern in the relationship of nationally representative data with data representing only urban or rural areas across geographies and were not able to correct those data for potential bias. We did not evaluate the trend and disease burden of other measures of adiposity that may better relate to specific health outcomes including waist circumference or waist to hip ratio. We identified few data points for some countries and the trends in these countries are mostly driven by the covariates included the models. We did not evaluate the health losses due to low BMI in this study and our results might be a conservative estimate of the overall disease burden of suboptimal BMI. We obtained the effect size of BMI on health outcomes from prospective observational studies and the possibility of confounding by lifestyle habits cannot be excluded. Our estimation of relative risks did not capture possible differences due to ethnicity and did not account for the possibility of geographic variation for relative risk curves or optimal level for BMI. The relative risks of BMI on disease endpoints are mostly obtained from meta-analyses or pooled analyses of prospective observational studies. These studies, generally excluded people with prevalent chronic diseases from the analysis of relative risk estimation. Thus, our estimates represent the effect of BMI in people without underlying diseases. This issue might be particularly important for older age groups were the prevalence of chronic disease increases. Finally, other probable complications or forms of BMI-related burden (e.g., disease burden in children) were not included.

In summary, our study provides one of the most comprehensive assessments of the trends and disease burden of high BMI to date. Our results show that both the rate and the disease burden of high BMI is increasing globally. This highlights the need for implementation of multicomponent interventions to reduce the prevalence and disease burden of high BMI.

## Supplementary Material

Supplementary Appendix
