# Supplementary material for: Health Effects of Overweight and Obesity in 195 Countries over 25 Years
Source: N Engl J Med. Author manuscript. (PMC5477817; doi:10.1056/NEJMoa1614362)
Supplement: Supplementary Appendix [file GBD2015-appendix.pdf]

Supplementary Appendix

This appendix has been provided by the authors to give readers additional information about their work.

Supplement to: The GBD 2015 Obesity Collaborators. Health effects of overweight and obesity in 195 countries over 25 years. N Engl J Med. DOI: 10.1056/NEJMoa1614362

Health Effects of Overweight and Obesity in 195 Countries over 25 Years

Supplemental Materials

TABLE OF CONTENTS

	Page #
Data Sources	4
Eligibility Criteria	5
Data collection process	5
Self-report bias adjustment	7
Age and sex splitting	8
Prevalence estimation for overweight and obesity	8
Estimating mean BMI	13
Assessment of risk-outcome pairs	14
Computing attributable burden	14
Flowchart	14
Exposure definition	14
Relative risk	14
Theoretical minimum risk exposure level	15
Population attributable fraction	15
Attributable burden	15
Socio-Demographic Index	16
Decomposition	16
References	138

TABLE OF FIGURES & TABLES

	Page #
**Figure S1:** Age−standardized percent relative change in obesity prevalence between 1980 and 2015 among adults (males [a], females[b]) and children (males [c], females[d]).	17
**Figure S2:** Age−standardized absolute change in obesity prevalence between 1980 and 2015 among adults (males [a], females[b]) and children (males [c], females[d]).	21
**Figure S3:** Age−standardized rates of mortality (males [a], females[b]) and disability-adjusted life years (males [c], females[d]) attributable to high body mass index.	25
**Figure S4:** Selection process of scientific literature and survey data (PRISMA)	29
**Figure S5:** Process diagram of estimating the disease burden of high BMI.	30
**Table S1:** Epidemiological evidence supporting causality between high body mass index (BMI) and disease endpoints.	31
**Table S2:** Relative risks of high body mass index (BMI), by age and sex, for each disease endpoint.	32
**Table S3:** The age-standardized prevalence of overweight and obesity among children and adults by sex in 1980 and 2015 among 195 countries.	33
**Table S4:** Disability adjusted life years (DALYs), deaths, and years lived with disability (YLDs) attributable to high body mass index (BMI) caused by the below causes in males and females in 1990 and 2015. Rates presented as number per 100,000 at the Global level for all adults over the age of 25, age-standardized.	39
**Table S5:** Decomposition of percent changes in cause-specific deaths and disability-adjusted life years (DALYs) related to high body mass index from 1990 to 2015 due to population growth, population ageing, risk exposure and the underlying rates of deaths and DALYs by quintiles of Socio−demographic Index.	40
**Table S6**: Disability-adjusted life years (DALYs) and mortality rates attributable to high body mass index BMI in males and females in 1990 and 2015 among 195 countries. Rates presented as number of deaths and DALYs per 100,000.	42

Table S7: Comparison of the root-mean-square error from models using only measured data with the models using both self-reported and measured data. 47

Table S8: Sources of epidemiological evidence used for assessment of causality between high body mass index and disease endpoints. 48

Table S9: Citations for all sources used for estimating body mass index, overweight, and obesity. 50

Data Sources

We systematically searched Medline to identify studies providing nationally or subnationally representative estimates of overweight prevalence, obesity prevalence, or mean body-mass index (BMI) there were published between 1 January 2014 and 31 December 2015 to update the systematic literature search previously performed as part of GBD 2013.[1]

The search for adults was conducted on 26 January 2016 using the following terms:

(("Body Mass Index"[Mesh] OR "Overweight"[Mesh] OR "Obesity"[Mesh]) AND "Geographic Locations"[Mesh] NOT "United States"[Mesh]) AND ("humans"[Mesh] AND "adult"[MeSH]) AND ("Data Collection"[Mesh] OR "Health Services Research"[Mesh] OR "Population Surveillance"[Mesh] OR "Vital statistics"[Mesh] OR "Population"[Mesh] OR "Epidemiology"[Mesh] OR "surve*"[TiAb]) NOT Comment[ptyp] NOT Case Reports[ptyp] NOT "hospital"[TiAb] AND ("2014/01/01"[Date - Publication] : "2015/12/31"[Date - Publication])

The search for children was conducted on 11 August 2016 using the following terms:

(((("Body Mass Index"[Mesh] OR "Overweight"[Mesh] OR "Obesity"[Mesh]) AND "Geographic Locations"[Mesh] NOT "United States"[Mesh]) AND ("humans"[Mesh] AND "child"[MeSH]) AND ("Data Collection"[Mesh] OR "Health Services Research"[Mesh] OR "Population Surveillance"[Mesh] OR "Vital statistics"[Mesh] OR "Population"[Mesh] OR "Epidemiology"[Mesh] OR "surve*"[TiAb]) NOT Comment[ptyp] NOT Case Reports[ptyp] NOT "hospital"[TiAb])) AND ("2014/01/01"[Date - Publication] : "2015/12/31"[Date - Publication])

Our search for adult estimates identified 2,036 abstracts, of which 127 met inclusion criteria and were extracted. The search for children estimates identified 971 articles, of which 146 were extracted. Including sources from the GBD 2013 search, a total of 10,627 articles were identified, of which 816 were included. Additionally, we searched the Global Health Data Exchange (GHDx) database for

individual-level data from major multinational survey series or country-specific surveys and identified 1,026 unique sources meeting the inclusion criteria. Figure S4 describes the study selection process.

Eligibility Criteria

We included nationally or subnationally representative studies providing data on mean BMI or prevalence of overweight or obesity among adults or children. For adults, studies were included if they defined overweight as BMI≥25 kg/m^2 and obesity as BMI≥30 kg/m^2, or if estimates using those cutoffs could be back-calculated from reported categories. For children (children ages 2-18), studies were included if they used International Obesity Task Force (IOTF) standards to define overweight and obesity thresholds. We only included studies reporting data collected between 1 January 1980 and 31 December 2015 in one of the 195 countries included in this analysis. Studies were excluded if using non-random samples (e.g., case-control studies or convenience samples); conducted among specific subpopulations (e.g., pregnant women, racial or ethnic minorities, immigrants, or individuals with specific diseases); using alternative methods to assess adiposity (e.g., waist-circumference, skin-fold thickness, or hydrodensitometry); having sample sizes of less than 20 per 5-year age-sex group; or providing inadequate information on any of the inclusion criteria. We also excluded review articles and non-English articles.

Data collection process

Where individual-level survey data were available, we computed mean BMI using weight and height and then used BMI to determine the prevalence of overweight and obesity. For individuals aged over 18 years, we considered them to be overweight if their BMI was greater than or equal to 25 kg/m^2, and obese if their BMI was greater than or equal to 30 kg/m^2. For individuals aged 2-18 years, we used monthly IOTF cutoffs[2] to determine overweight and obese status when age in months was available.

When only age in years was available, we used the cutoff for the 6 month of that year. We excluded studies using the World Health Organization (WHO) standards or country-specific cutoffs to define childhood overweight and obesity. At the individual-level, we considered BMI<10 kg/m^2 and BMI>70 kg/m^2 to be biologically implausible and excluded those observations.

The rationale for choosing to use the IOTF cutoffs over the WHO standards has been described elsewhere. [1] Briefly, the IOTF cutoffs provide consistent child-specific standards for ages 2-18 derived surveys covering multiple countries. On the other hand, the WHO growth standards apply to children under 5 and the WHO growth reference applies to children ages 5-19. The WHO growth reference for children ages 5-19 was derived from United States data which is less representative than the multinational data used by IOTF. Additionally, the switch between references at age 5 can produce artificial discontinuities.[1] Given that we estimate global childhood overweight and obesity for ages 2-19 (with ages 19 using standard adult cutoffs), the IOTF cutoffs were preferable. Additionally, we found that IOTF cutoffs were more commonly used in scientific literature covering childhood obesity.

From report and literature data, we extracted data on mean BMI, prevalence of overweight, and prevalence of obesity, measures of uncertainty for each, and sample size, by the most granular age and sex groups available.

In addition to the primary indicators described above, we extracted relevant survey-design variables, including primary sampling unit, strata, and survey weights, which were used to tabulate individual-level microdata and produce accurate measures of uncertainty. We extracted three study-level covariates: 1) whether height and weight data were measured or self-reported, 2) whether the study was predominantly conducted in an urban area, rural area, or both, and 3) the level of representativeness of the study (national or subnational). Finally, we extracted relevant demographic indicators, including location, year, age, and sex. We estimated the standard error of the mean from individual-level data where available and used the reported standard error of the mean for published data. When multiple

data sources were available for the same country, we included all of them in our analysis. If data from the same data source were available in multiple formats such individual-level data and tabulated data, we used individual-level data.

Self-report bias adjustment

We included both measured and self-reported data. Of 68,539,856 person-years of data, 16,944,472 (24.7%) were self-reported. We tested for bias in self-report data compared to measured data, which is considered to be the gold-standard. We found that there was no significant difference between measured and self-reported data for children ages 2-19. For adults, we adjusted self-reported data for overweight prevalence, obesity prevalence, and mean BMI using the following nested hierarchical mixed-effects regression models, fit using restricted maximum likelihood separately by sex:

$$\mathrm{logit(overweight)}_{c,a,t} = \beta_0 + \beta_1 m + \sum_{k=2}^{15} \beta_k I_{A[a]} + \sum_{l=16}^{41} \beta_l\, I_{A[a]} I_{M[m]} + \alpha_s + \alpha_s m + \alpha_r + \alpha_r m + \alpha_c + \alpha_c m + \alpha_t + \alpha_t m + \epsilon_{c,a,t}$$

$$\mathrm{logit(obesity)}_{c,a,t} = \beta_0 + \beta_1 m + \sum_{k=2}^{15} \beta_k I_{A[a]} + \sum_{l=16}^{41} \beta_l\, I_{A[a]} I_{M[m]} + \alpha_s + \alpha_s m + \alpha_r + \alpha_r m + \alpha_c + \alpha_c m + \alpha_t + \alpha_t m + \epsilon_{c,a,t}$$

$$\log(\mathrm{BMI})_{c,a,t} = \beta_0 + \beta_1 m + \sum_{k=2}^{15} \beta_k I_{A[a]} + \sum_{l=16}^{41} \beta_l\, I_{A[a]} I_{M[m]} + \alpha_s + \alpha_s m + \alpha_r + \alpha_r m + \alpha_c + \alpha_c m + \alpha_t + \alpha_t m + \epsilon_{c,a,t}$$

Where m is a fixed effect on measurement (binary, either measured (1) or self-report (0)), $I_{A[a]}$ is an indicator variable for specific age group A, $I_{A[a]} I_{M[m]}$ is an interaction term between age and measurement, α_s, α_r, and α_c are random effects at the super region, region, and country, respectively, and α_t is a random effect by time-period (1980-1991, 1992-2003, 2004-2015). The models were run in Stata 13. Random effects at the country level and time-period level were used to fit the models, but were taken as noise and were not used in adjustment of self-reported data. We propagated the uncertainty in the self-report adjustment model by adding the variance of each of the regression coefficients used in adjustment to the data variance in delta-transformed space. After adjustment, regressions confirmed that self-

reported data was no longer significantly different from measured data. As an additional sensitivity analysis we ran a set of models for overweight and obesity using only measured data and compared the root-mean squared error (RMSE) of these model to the RMSE of the full models. RMSE was calculated comparing measured data to the predicted estimates. Differences in RMSE were minimal and are shown in Table S7.

Age and sex splitting

Any report or literature data provided in age groups wider than the standard 5-year age groups or as both sexes combined were split using the approach used by Ng et al.[4] Briefly, age-sex patterns were identified using sources with data on multiple age-sex groups and these patterns were applied to split aggregated report and literature data. Uncertainty in the age-sex split was propagated by multiplying the standard error of the data by the square root of the number of splits performed. We did not propagate the uncertainty in the age pattern and sex pattern used to split the data as they seemed to have small effect.

Prevalence estimation for overweight and obesity

After adjusting for self-report bias and splitting aggregated data into 5-year age-sex groups, we used spatiotemporal Gaussian process regression (ST-GPR) to estimate the prevalence of overweight and obesity. Prevalence models for adults were restricted to using only adult data (ages 20+). Due to data scarcity in child data for some geographies and time periods, prevalence models for children included data for both children and adults, but only the childhood estimates were retained. This modeling approach has been described in detail elsewhere.[3,4]

Briefly, we estimated the prevalence of overweight and obesity in country c, for age group a, and sex s at time t ($p_{c,a,s,t}$) using the equation below:

$$logit(p_{c,a,s,t}) = g_{c,a,s}(t) + \epsilon_{c,a,s,t}$$

where

$$g_{c,a,s}(t) \sim GP\left(m_{c,a,s}(t), Cov\left(g_{c,a,s}(t)\right)\right).$$

$$\epsilon_{c,a,s,t} \sim Normal(0, \sigma_p^2\ ),$$

σ_p^2 represents the error variance, which is composed of the squared standard error of the observed data point as well as the prediction errors from the self-report adjustment, if applicable, ($m_{c,a,s}(t)$) is the mean prior input to GPR, and $Cov\left(g_{c,a,s}(t)\right)$ is the covariance function input to GPR.

We used an empirical mean prior ($m_{c,a,s}(t)$), which was the output of the first-stage linear model plus the smoothed residuals:

$$m_{c,a,s}(t) = X_{c,a,s}\beta + h(r_{c,a,s,t})$$

where $X\beta$ is the summation of the components from the following linear regressions, fit separately by sex, and $h(r_{c,a,s,t})$, is a smoothing function for the residuals, $r_{c,a,s,t}$, derived from the following linear models for adults only:

$$\mathrm{logit}(\mathrm{ow_{c,a,t}}) = \beta_0 + \beta_1 \mathrm{energy_{c,t}} + \beta_2\ \mathrm{lat_c} + \beta_3 \mathrm{urbanicity_{c,t}} + \sum_{k=4}^{16} \beta_k \mathrm{I_{A[a]}} + \epsilon_{c,a,t}$$

$$\mathrm{logit}\left((\frac{\mathrm{ob}}{\mathrm{ow}})_{\mathrm{c,a,t}}\right) = \beta_0 + \beta_1 \mathrm{energy_{c,t}} + \beta_2\ \mathrm{lat_c} + \beta_3 \mathrm{urbanicity_{c,t}} + \sum_{k=4}^{16} \beta_k \mathrm{I_{A[a]}} + \epsilon_{c,a,t}$$

and adults including children:

$$\mathrm{logit}(\mathrm{ow_{c,a,t}}) = \beta_0 + \beta_1 \mathrm{energy_{c,t}} + \beta_2\ \mathrm{lat_c} + \beta_3 \mathrm{urbanicity_{c,t}} + \sum_{k=4}^{20} \beta_k \mathrm{I_{A[a]}} + \epsilon_{c,a,t}$$

$$\mathrm{logit}\left((\frac{\mathrm{ob}}{\mathrm{ow}})_{\mathrm{c,a,t}}\right) = \beta_0 + \beta_1 \mathrm{energy_{c,t}} + \beta_2\ \mathrm{lat_c} + \beta_3 \mathrm{urbanicity_{c,t}} + \sum_{k=4}^{20} \beta_k \mathrm{I_{A[a]}} + \epsilon_{c,a,t}$$

where $\mathrm{energy}_{c,t}$ is a 10-year lag distributed energy intake per capita in country *c* at year *t*, lat_c is the absolute latitude of country c, $\mathrm{urbanicity}_{c,t}$ is the proportion of people living in urban areas in country c in time t, and $I_{A[a]}$ is an indicator variable for specific age group A that the overweight prevalence point ($\mathrm{ow}_{c,a,t}$) or obese as a proportion of overweight point ($(\frac{\mathrm{ob}}{\mathrm{ow}})_{c,a,t}$) is capturing.

While the linear model component of the mean function captures the general trend in exposures over time, much of the data varaibility may still not be adequately accounted for. To address this, we fit a locally weighted polynomial regression (LOESS) function $h(r_{c,a,s,t})$ to systematically estimate this residual variability by borrowing strength across time, age, and space patterns. The time adjustment parameter(λ) borrows strength from neighboring time points. The age adjustment parameter (ω) borrows strength from data in neighboring age groups. The space adjustment parameter (ξ) borrows strength across the hierarchy of geographical locations.

To estimate the $w_{c,a,s,t}$ (the final weight assigned to observation $r_{c,a,s,t}$ with reference to a focal observation r_{c_0,a_0,s_0,t_0}), we first generated a preliminary weight $w'_{c,a,s,t}$ for smoothing over time, which was based on the scaled distance along the time dimension of the two observations:

$$w'_{c,a,s,t} = \left(1 - \left(\frac{|t - t_0|}{1 + \max|t - t_0|}\right)^{\lambda}\right)^3$$

Next, we calculated the weight $w''_{c,a,s,t}$ to smooth over age, which is based on a distance along the age dimension of two observations. For a point between the age a of the observation $r_{c,a,s,t}$ and a focal observation r_{c_0,a_0,s_0,t_0}, the weight, given an age adjustment parameter ω, is defined as follows:

$$w''_{c,a,s,t} = \frac{1}{e^{\omega|a-a_0|}}$$

Finally, these combined weights were multiplied and further adjusted to account for geographic patterns.

Specifically, we defined a geospatial relationship by categorizing data based on the GBD location hierarchy. We adapted the weighting strategy used in previous studies estimating time series of global indicators to be more flexible with respect to estimating subnational locations and to borrow strength from all levels. [4,5] A vector of spatial weights corresponding to each level of the location hierarchy was derived as $[\xi, \xi * (1-\xi)^{n_1-1}, \ldots, \xi * (1-\xi)^{n_i-1}, (1-\xi)^{n_i}]$, where the vector is expanded to include the number, n_i, levels in the location hierarchy between the location being estimated and global, which recieves a pre-rescaling weight of $(1-\xi)^{n_i}$. For example, estimating a country would use the following weighting scheme:

- Country data: ξ
- Regional data not from the country being estimated: $\xi * (1- \xi)$
- Data from other regions in the same super region: $\xi * (1-\xi)^2$
- Global data from other super regions: $(1-\xi)^3$

A full derivation of weights for each category, assuming the location being estimated was a country, follows:

1) If the observation $r_{c,t}$ belongs to the same country c_0 of the focal observation r_{c_0,t_0}:

$$w_{c,a,s,t} = \frac{\xi(w'_{c,a,s,t}w''_{c,a,s,t})}{\sum_{c=c_0}(w'_{c,a,s,t}w''_{c,a,s,t})} \qquad \forall c = c_0$$

2) If the observation $r_{c,t}$ belongs to a different country than the focal observation r_{c_0,t_0}, but both belong to the same region R:

$$w_{c,a,s,t} = \frac{\xi * (1- \xi)\,(w'_{c,a,s,t}w''_{c,a,s,t})}{\sum_{c\neq c_0}(w'_{c,a,s,t}w''_{c,a,s,t})} \qquad \forall c \neq c_0 \cap\ R[c] = R[c_0]$$

3) If the observation $r_{c,t}$ belongs to the same super region SR but to a both different country c_0 and region $R[c_0]$ than the focal observation r_{c_0,t_0}:

$$w_{c,a,s,t} = \frac{\xi * (1 - \xi)^2 \, (w'_{c,a,s,t} w''_{c,a,s,t})}{\sum_{c \neq c_0} (w'_{c,a,s,t} w''_{c,a,s,t})} \qquad \forall c \neq c_0 \cap R[c] \neq R[c_0] \cap SR[c] = SR[c_0]$$

4) If the observation $r_{c,t}$ is from a different super region than the focal observation r_{c_0,t_0}(ie. all other data currently not receiving a weight):

$$w_{c,a,s,t} = \frac{(1 - \xi)^3 \, (w'_{c,a,s,t} w''_{c,a,s,t})}{\sum_{c \neq c_0} (w'_{c,a,s,t} w''_{c,a,s,t})} \qquad \forall c \neq c_0 \cap R[c] \neq R[c_0] \cap SR[c] \neq SR[c_0]$$

Selection of the parameters was determined based on the results of out-of-sample cross-validation performed by Ng et al. We used three different values for ξ depending on data-coverage. The highest ξ (0.99) was used in countries with high data coverage (≥15 years covered), whereas a mid-range ξ (0.8) and a low ξ (0.7) was used in countries with 5-14 and 1-4 years of data covered. To control the rate of decay in weight for points further away across the time and age dimensions, we used a single λ (0.9) and a single ω (1.5).

For the covariance function, we used the Matern covariance function:

$$M(t,t') = \sigma^2 \frac{2^{1-v}}{\Gamma(\mathrm{v})} \left(\frac{d(t,t')\sqrt{2v}}{l} \right)^v K_v \left(\frac{d(t,t')\sqrt{2v}}{l} \right)$$

where $d(\cdot)$is a distance function; σ^2, v, l, and K_v are hyperparameters of the covariance function—specifically σ^2 is the marginal variance, v is the smoothness parameter that defines the differentiability of the function, l is the length scale, which roughly defines the distance between which two points become uncorrelated, and K_v is the Bessel function. Based on previous analyses, we approximated σ^2 by $MADN(r'_{c,t})$, which is the normalized absolute deviation of the residuals from the smoothing step by region and used the parameter specifications $v = 2$ and $l = 15$.

Based on the specification above, to predict the time series of overweight prevalence p_{c,a,s,t_*}, for country *c*, age *a*, and sex *s* for time t_*, we integrated over $g_{c,a,s}(t_*)$ to obtain the following:

$$logit(p_{c,a,s,t_*}) \sim N\left(m_{c,a,s}(t_*), \sigma_p^2 I + Cov\left(g_{c,a,s}(t_*)\right)\right)$$

From this derivation, we generated the prevalence estimates as well as the uncertainty intervals. The analysis was implemented though PyMC package in Python. Random draws of 1,000 samples were obtained from the distributions above for every country, age, and sex group. The final estimated prevalence for each country, age, and sex group was the mean of the draws. In addition, uncertainty intervals were obtained by taking the 2.5 and 97.5 percentiles of the samples.

Estimating mean BMI

To estimate the mean BMI for adults in each country, age, sex, and time period 1980-2015, we first used the following nested hierarchical mixed-effects model, fit using restricted maximum likelihood on data from sources containing estimates of all three indicators (prevalence of overweight, prevalence of obesity, and mean BMI), in order to characterize the relationship between overweight, obesity, and mean BMI:

$$\log(\mathrm{BMI}_{c,a,s,t}) = \beta_0 + \beta_1 \mathrm{ow}_{c,a,s,t} + \beta_2 \mathrm{ob}_{c,a,s,t} + \beta_3 \mathrm{sex} + \sum_{k=4}^{17} \beta_k \mathrm{I}_{A[a]} + \alpha_s(1 + \mathrm{ow}_{c,a,s,t} + \mathrm{ob}_{c,a,s,t}) + \alpha_r(1 + \mathrm{ow}_{c,a,s,t} + \mathrm{ob}_{c,a,s,t}) + \alpha_c(1 + \mathrm{ow}_{c,a,s,t} + \mathrm{ob}_{c,a,s,t}) + \epsilon_{c,a,s,t}$$

where $\mathrm{ow}_{c,a,s,t}$ is the prevalence of overweight in country *c*, age *a*, sex *s*, and year *t*, $\mathrm{ob}_{c,a,s,t}$ is the prevalence of obesity in country *c*, age *a*, sex *s*, and year *t*, sex is a fixed effect on sex, $I_{A[a]}$ is an indicator variable for age, and α_s, α_r, and α_c are random effects at the super region, region, and country, respectively. The model was run in Stata 13.

We applied the regression coefficients to the 1,000 draws of overweight prevalence and obesity prevalence produced through ST-GPR to estimate 1,000 draws of mean BMI for each country, year, age,

and sex. This approach ensured that overweight prevalence, obesity prevalence, and mean BMI were correlated at the draw level.

Assessment of risk-outcome pairs

Risk-outcome pairs were defined based on strength of available evidence supporting a causal effect. Table S1 reports the results of our assessment for included risk-outcome pairs and Table S8 reports the supporting scientific literature.

Computing attributable burden

Flowchart

The process of estimating the disease burden of high BMI has been shown in Figure S5.

Exposure definition

Exposure to high BMI is defined using metrics related to national and subnational estimates of BMI. If a person has a BMI of 22.5 kg/m^2 or greater (midpoint of the optimal range of 20-25 kg/m^2), he/she is considered at risk for a range of diseases including cardiovascular diseases, musculoskeletal disorders, and cancers.

Relative risk

The relative risk per 5-unit change in BMI for each disease endpoint was obtained from meta-analyses, and where available, pooled analyses of prospective observational studies. Relative risks for all 20 outcomes, by age and sex, are reported in Table S2.

Theoretical minimum risk exposure level

The theoretical minimum risk exposure level (TMREL) of BMI was determined based on the BMI level that was associated with the lowest risk of all-cause mortality in prospective cohort studies. In GBD 2015, based on the findings of the most recent pooled analysis of prospective cohorts,[4] we changed the TMREL of BMI from 21-23 to 20-25 kg/m2.

Population attributable fraction

Population attributable fractions (PAFs) are computed using the following equation:

$$PAF_{oasgt} = \frac{\int_{x=l}^{u} RR_{oasg}(x) P_{asgt}(x) dx - RR_{oasg}(TMREL)}{\int_{x=l}^{u} RR_{oasg}(x) P_{asgt}(x) dx}$$

Where PAF_{oasgt} is the population attributable fraction for cause o, age group a, sex s, geography g, and year t. $RR_{oasg}(x)$ is the relative risk as a function of exposure level x for cause o, age group a, sex s, and geography g with the lowest level of observed exposure as l and the highest as u; $P_{asgt}(x)$ is the distribution of exposure at x for age group a, sex s, geography g, and year t.; $TMREL$ is the theoretical minimum risk exposure level.[6]

Attributable burden

Burden attributable to high BMI is computed using the following equation:

$$AB_{asgt} = \sum_{o=1}^{w} DALY_{oasgt} PAF_{oasgt}$$

where AB_{asgt} is the attributable burden for age group a, sex s, geography g, and year t; $DALY_{oasgt}$ is total DALYs for cause o (of w relevant outcomes) for age group a, sex s, geography g, and year t; PAF_{oasgt} is the population attributable fraction (PAF) for cause o, age group a, sex s, geography g, and year t. The proportion of attributable deaths can analogously computed by sequentially substituting each metric in place of DALYs in the equation above.

Uncertainty of PAF estimates was calculated from 1,000 draws resulting from 1,000 PAF calculations using the 1,000 draws of exposure estimates, 1,000 draws of relative risk estimates, and 1,000 draws of the theoretical minimum risk exposure level. The 1,000 draws of PAF estimates were multiplied by 1,000 draws of deaths and DALYs to produce 1,000 draws of attributable burden. All components of the PAF calculation analysis were assumed to be independent of each other.

Socio-Demographic Index

Details on construction of the Socio-demographic index (SDI) are previously published.[3] Briefly, SDI is a composite indicator (range 0-1), calculated using the Human Development Index methodology, that includes average educational attainment in the population over age 15, lag-distributed income per capita, and total fertility rate. The list of countries with their SDI level is provided in Table S3.

Decomposition

We applied the decomposition methods developed by Das Gupta to decompose changes in all-cause and cause-specific DALYs due to changes in population growth, population structure, exposure to risk, and background death and DALY rates. Details of the decomposition analysis are reported in elsewhere.[3]

Figure S1a. Age−standardized Relative Percent Change in Obesity Prevalence between 1980 and 2015 for Males (ages >= 20)

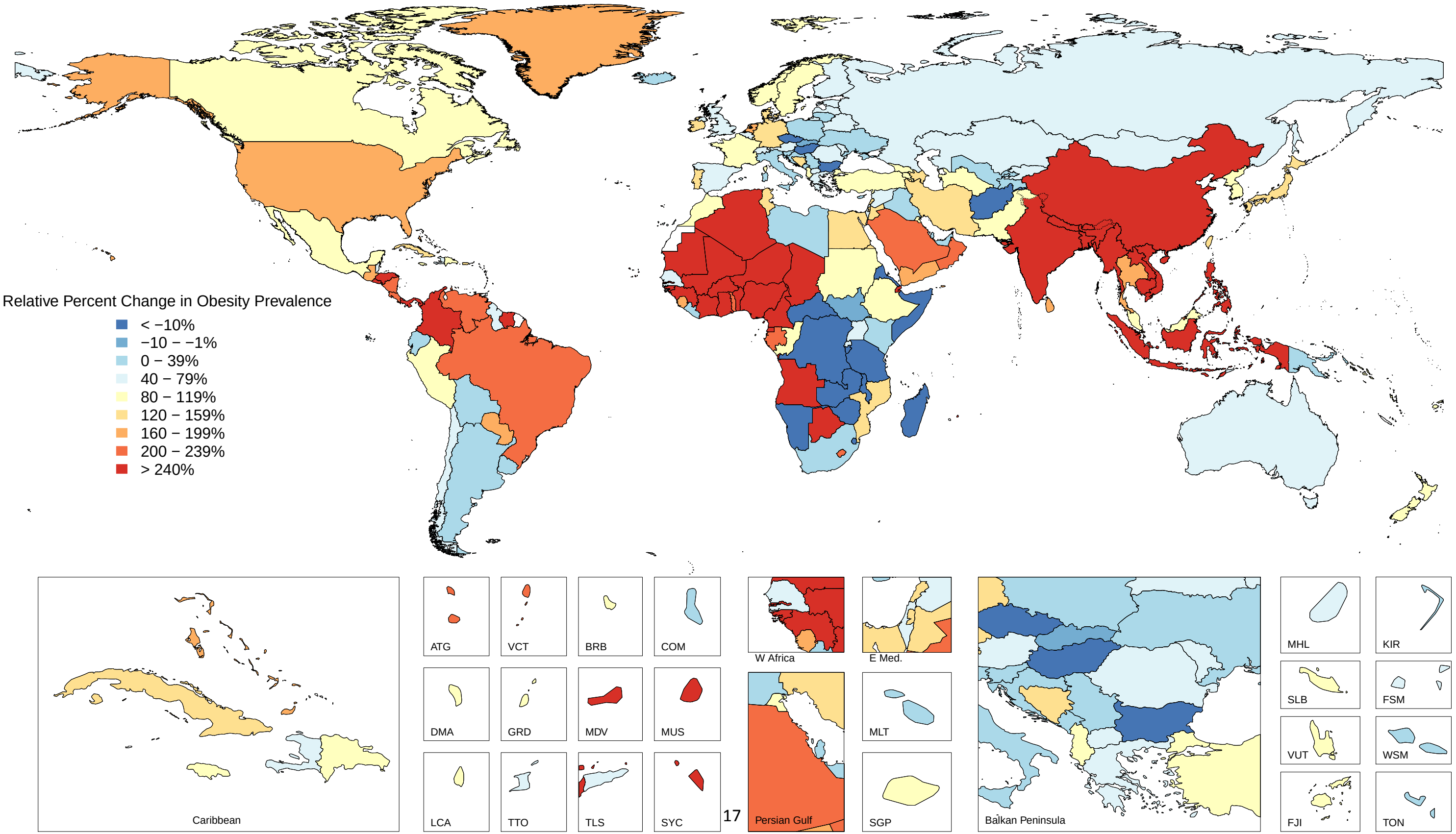

Figure S1b. Age−standardized Relative Percent Change in Obesity Prevalence between 1980 and 2015 for Females (ages >= 20)

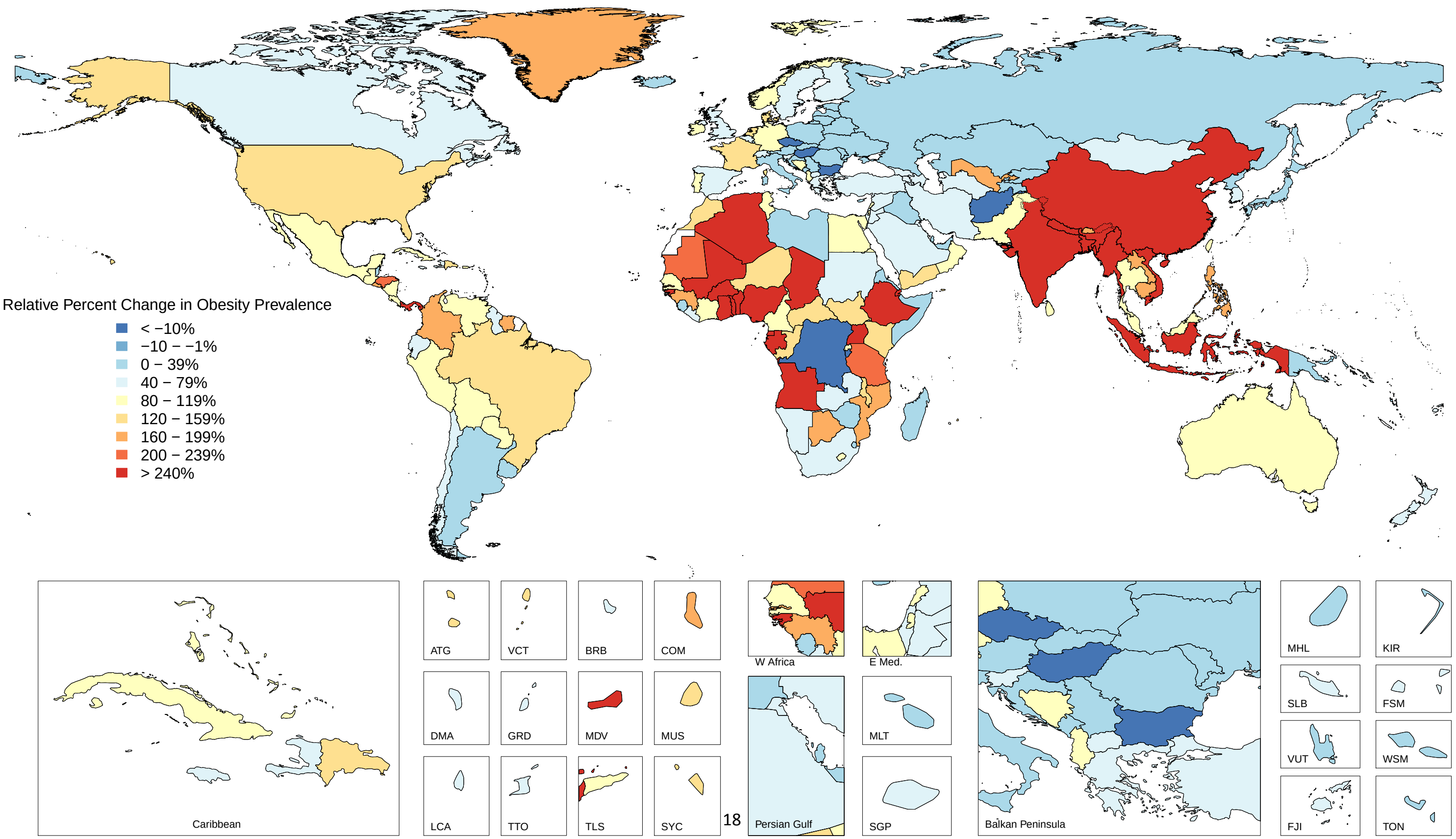

Figure S1c. Age−standardized Relative Percent Change in Obesity Prevalence between 1980 and 2015 for Males (ages 2 − 19)

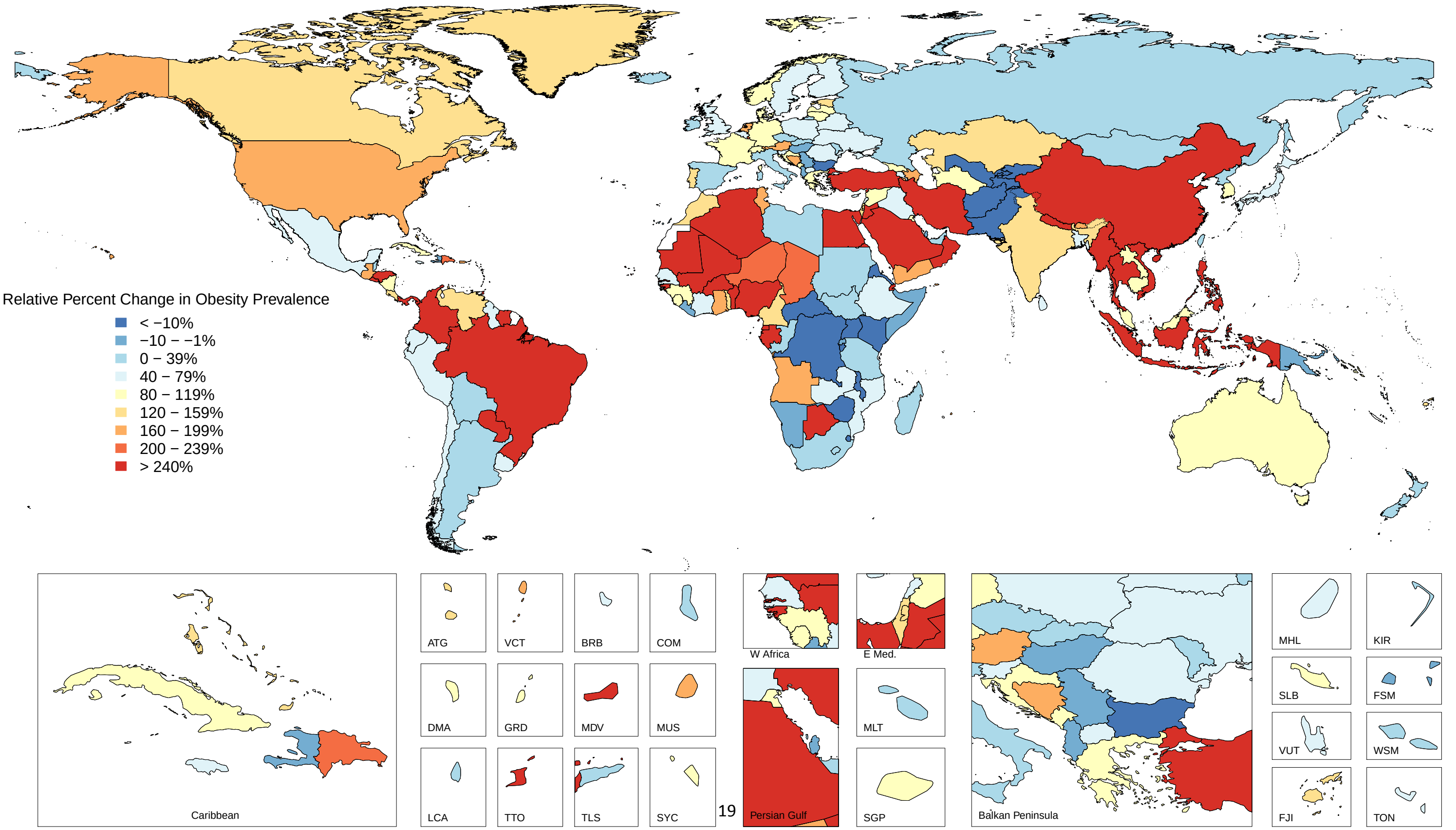

Figure S1d. Age−standardized Relative Percent Change in Obesity Prevalence between 1980 and 2015 for Females (ages 2 − 19)

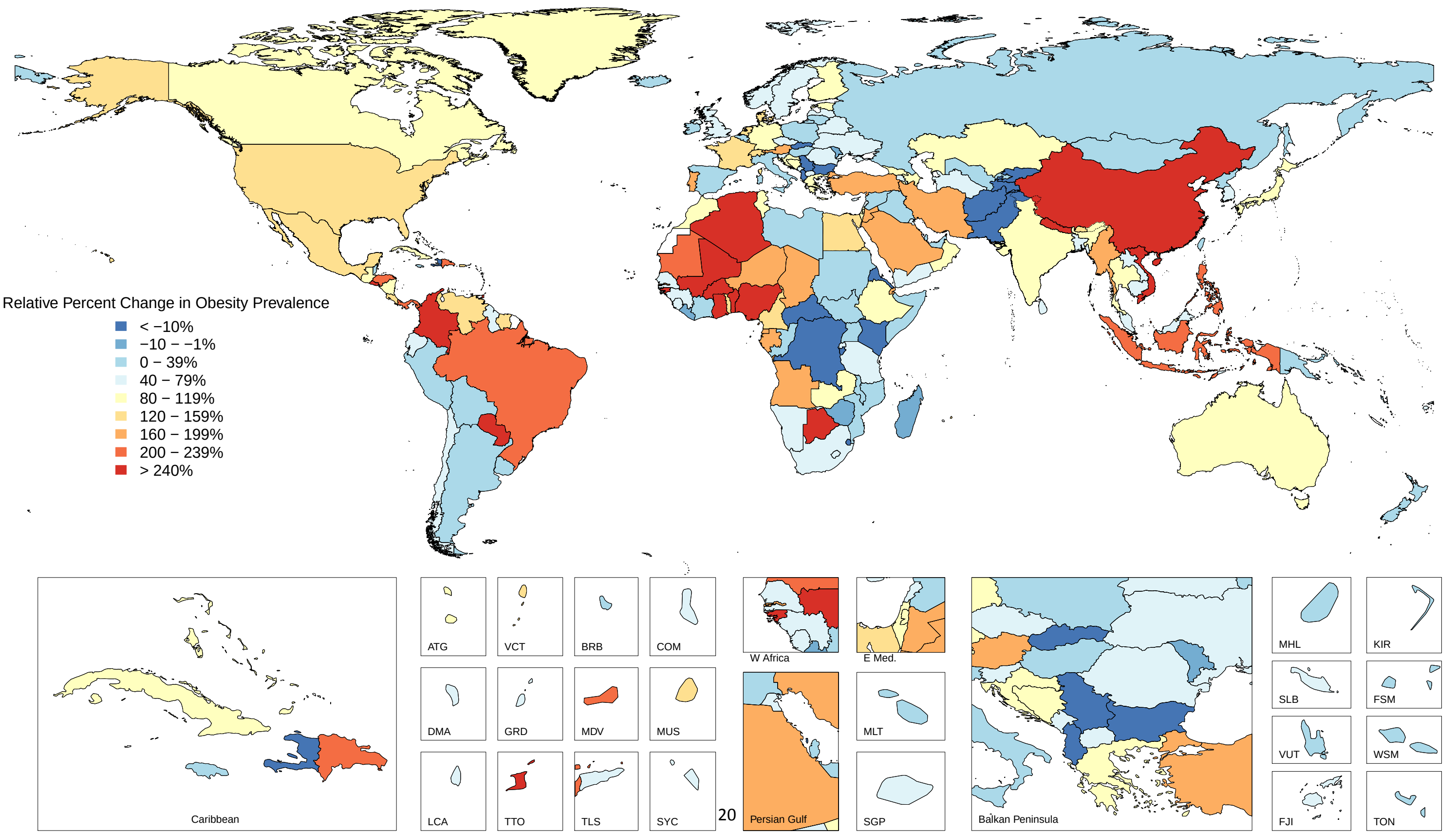

Figure S2a. Age−standardized Absolute Change of Prevalence of Obesity for Males (ages >= 20) in 2015

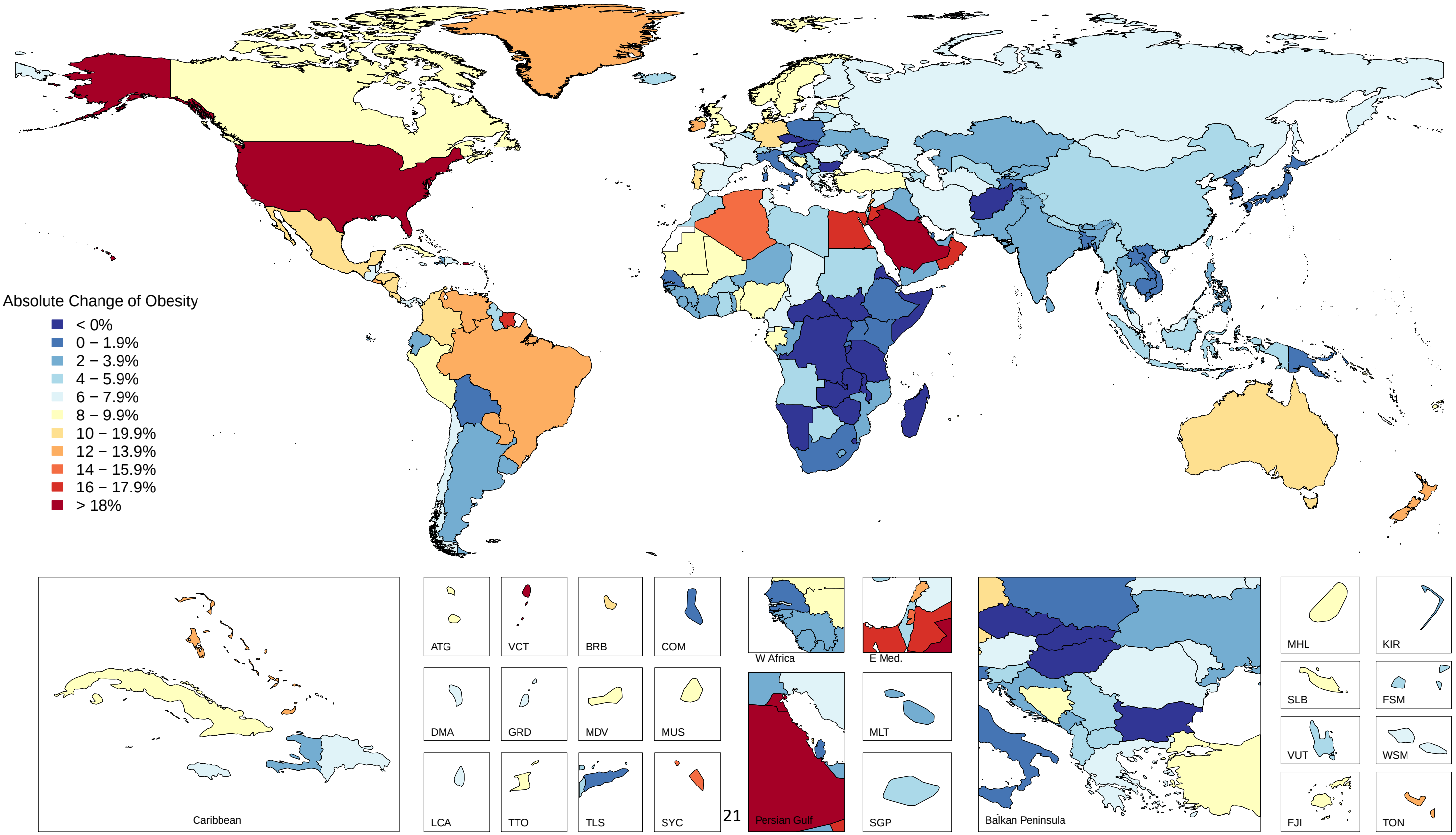

Figure S2b. Age−standardized Absolute Change of Prevalence of Obesity for Females (ages >= 20) in 2015

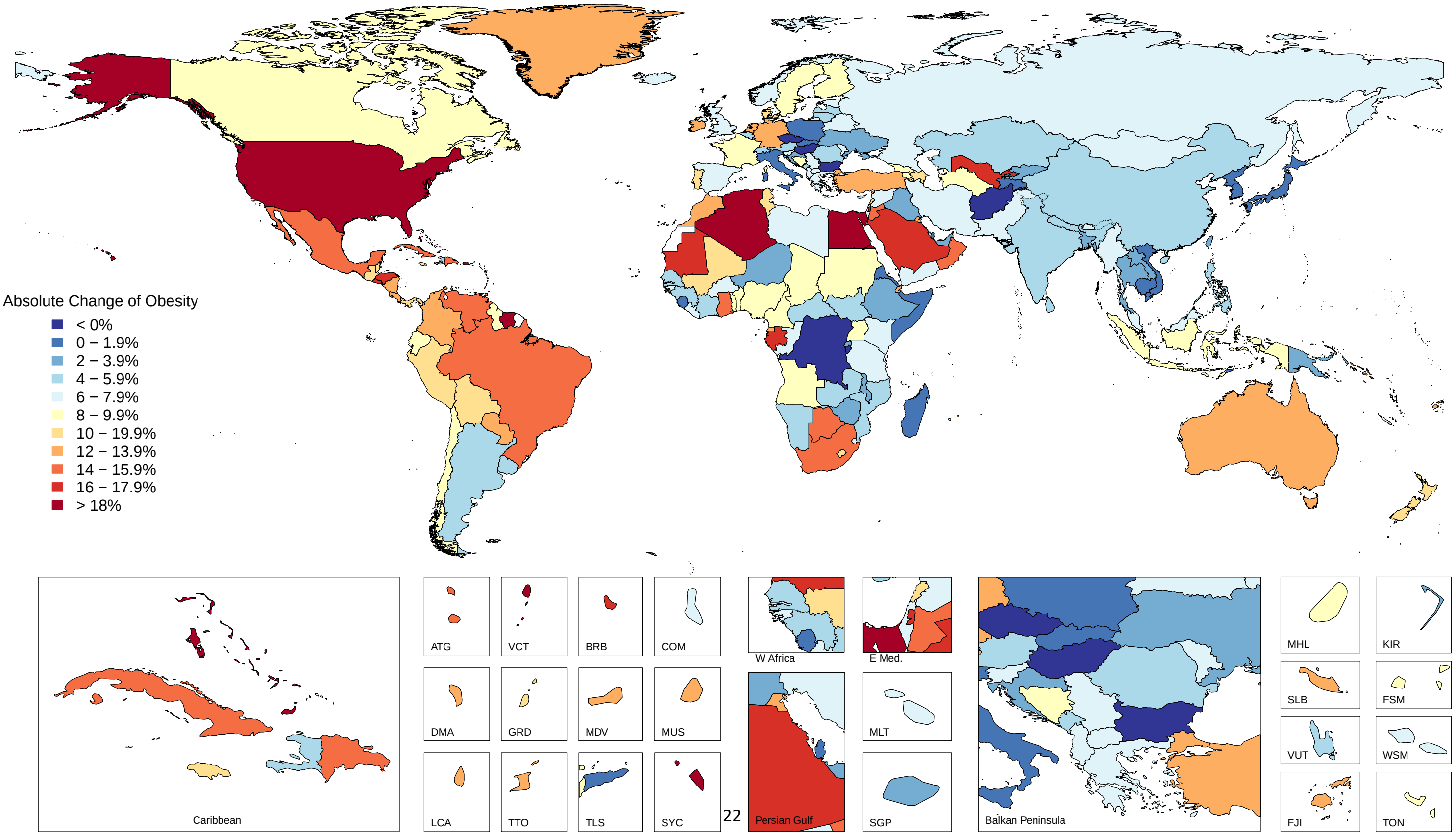

Figure S2c. Age−standardized Absolute Change of Prevalence of Obesity for Males (ages 2 − 19) in 2015

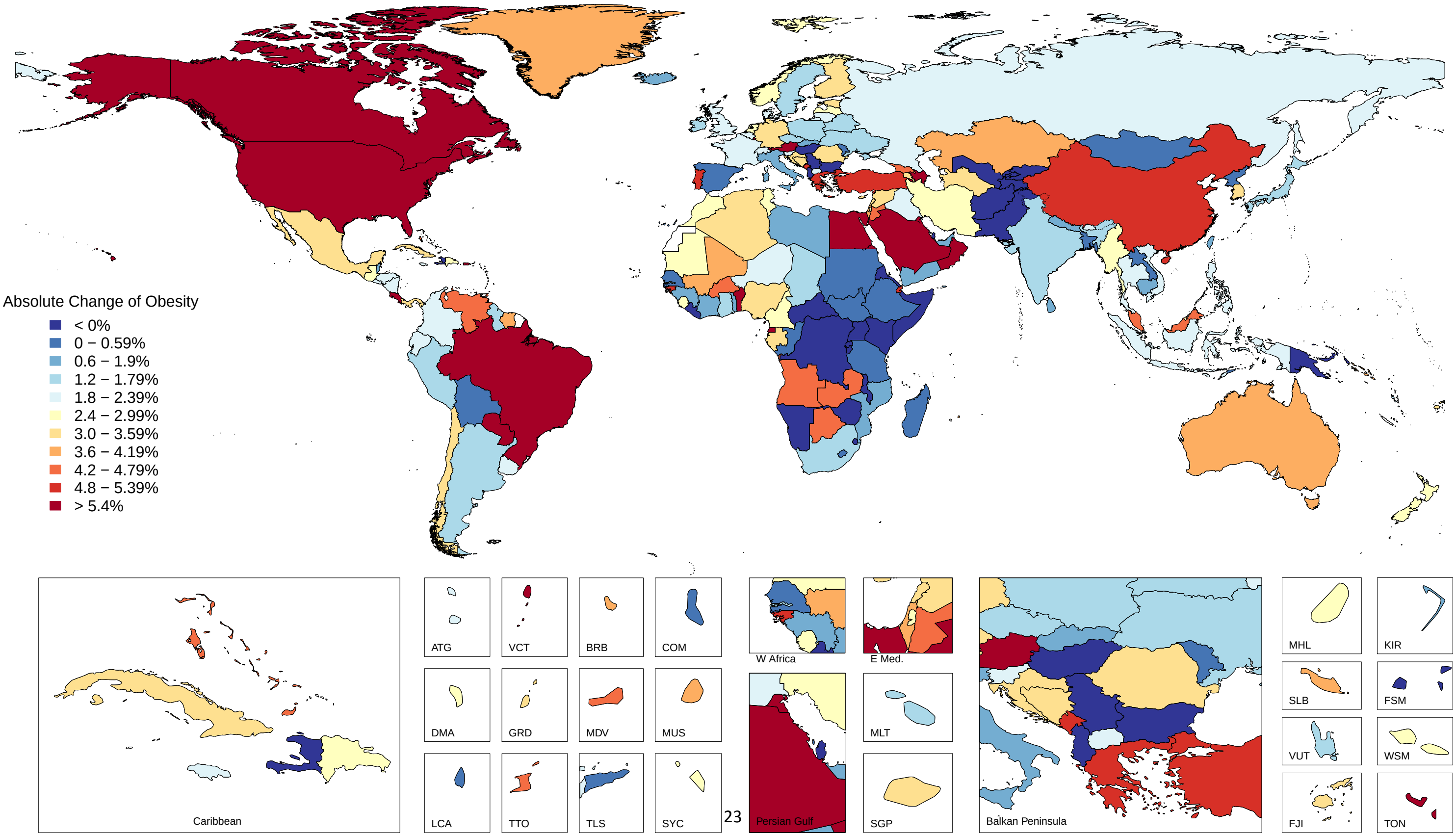

Figure S2d. Age−standardized Absolute Change of Prevalence of Obesity for Females (ages 2 − 19) in 2015

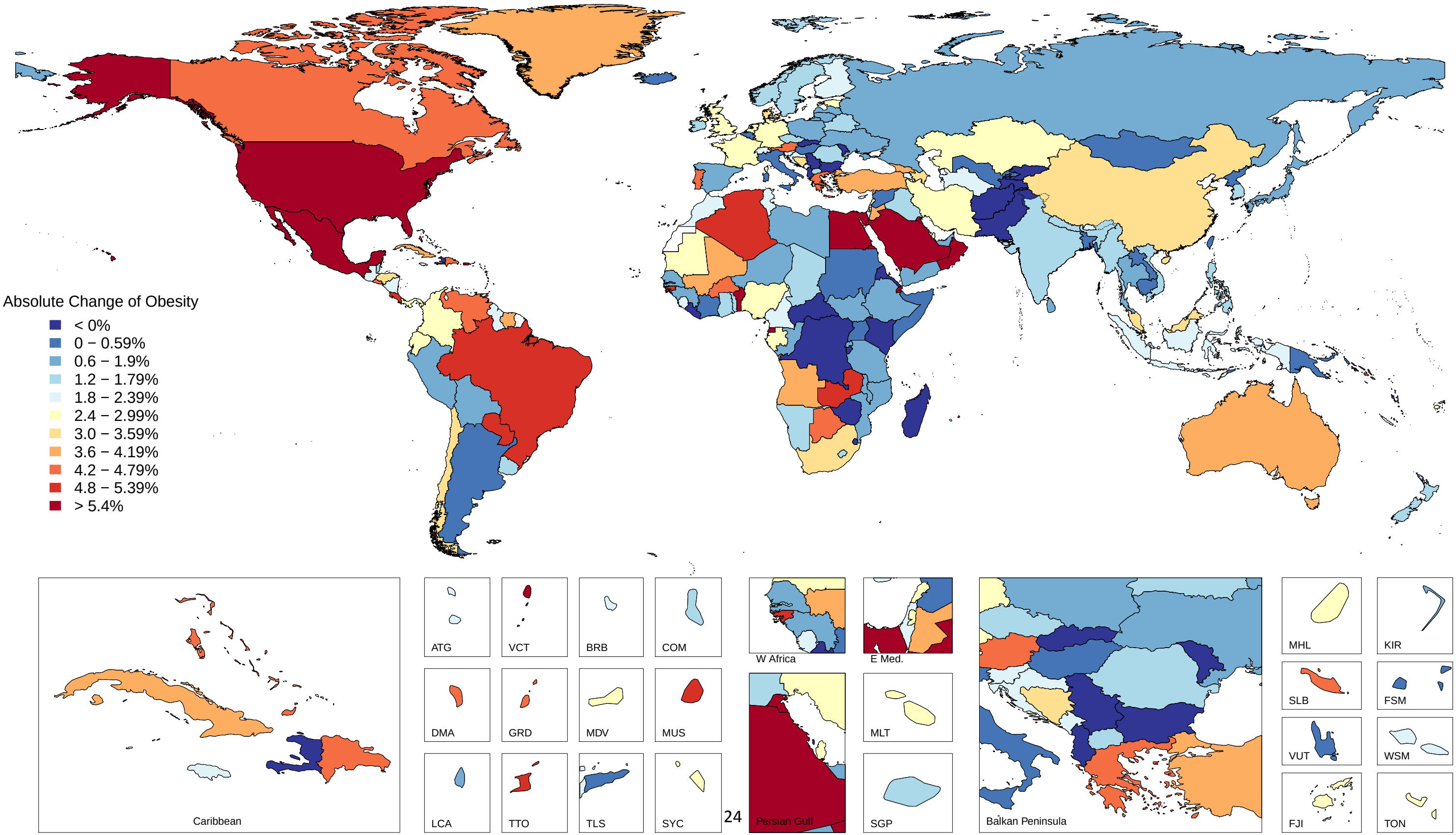

Figure S3a. Age−standardized Mortality Rate Attributable to High−BMI for Males (ages >= 25) in 2015

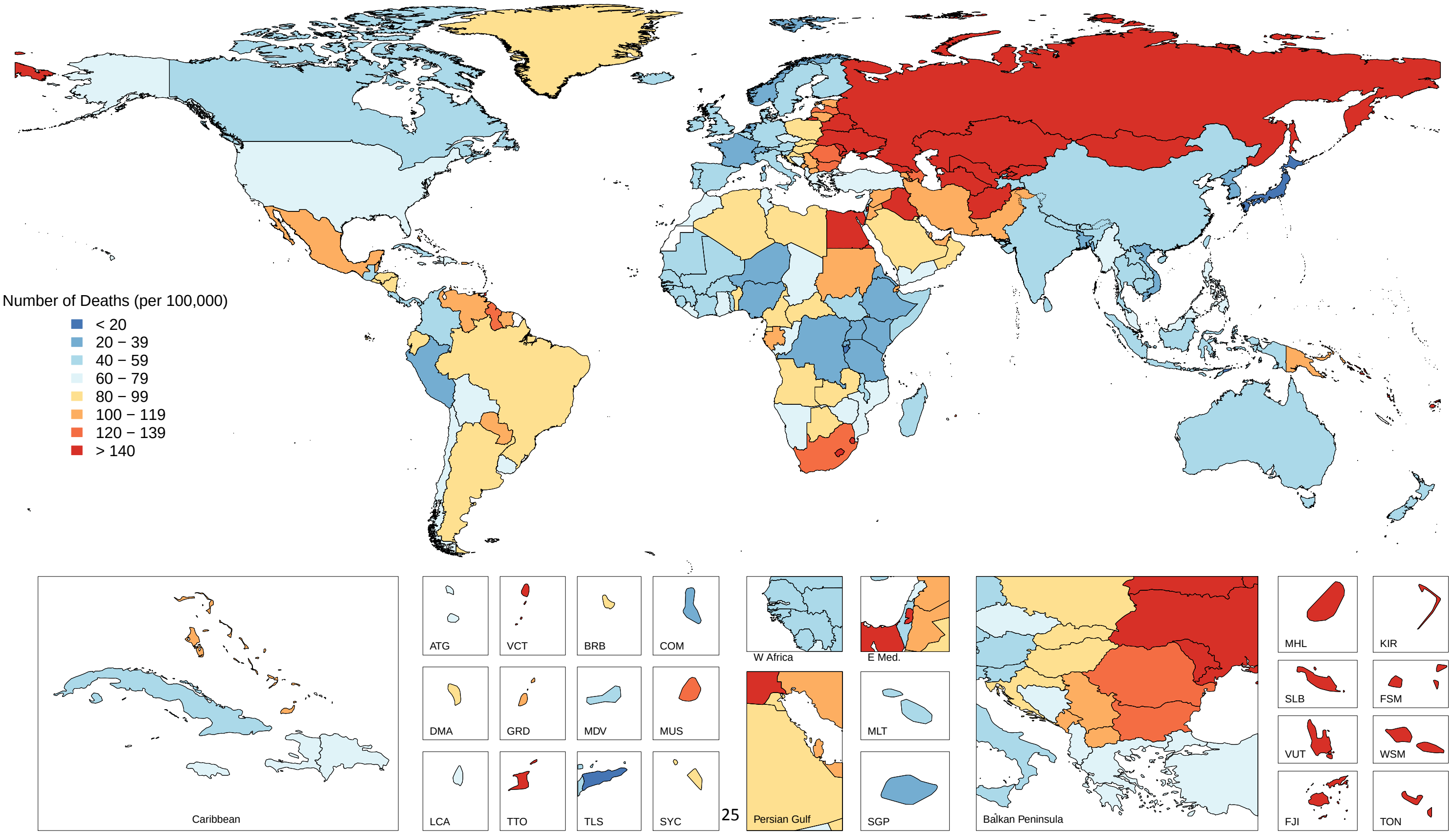

Figure S3b. Age−standardized Mortality Rate Attributable to High−BMI for Females (ages >= 25) in 2015

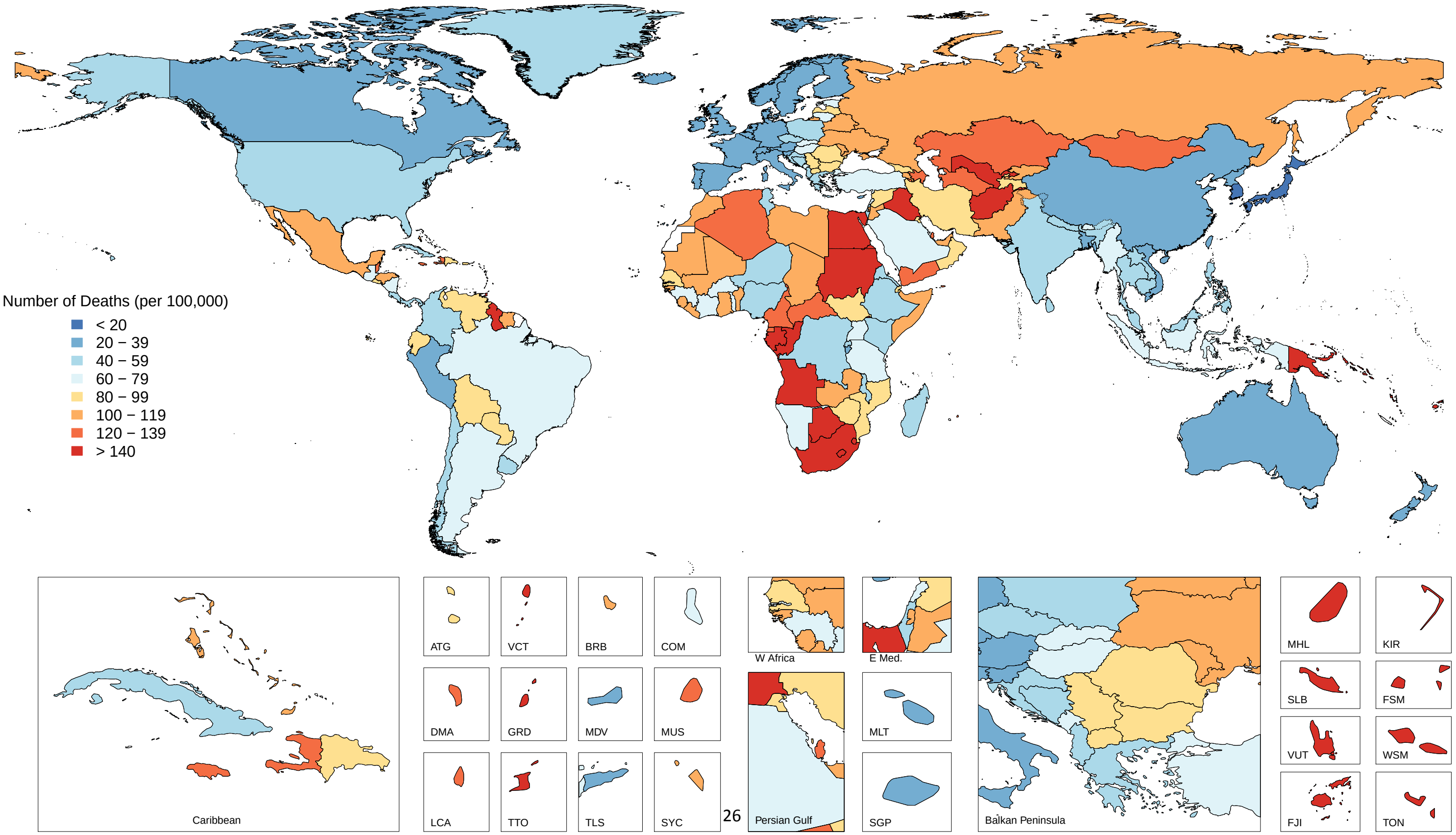

Figure S3c. Age−standardized DALY Rate Attributable to High−BMI for Males (ages >= 25) in 2015

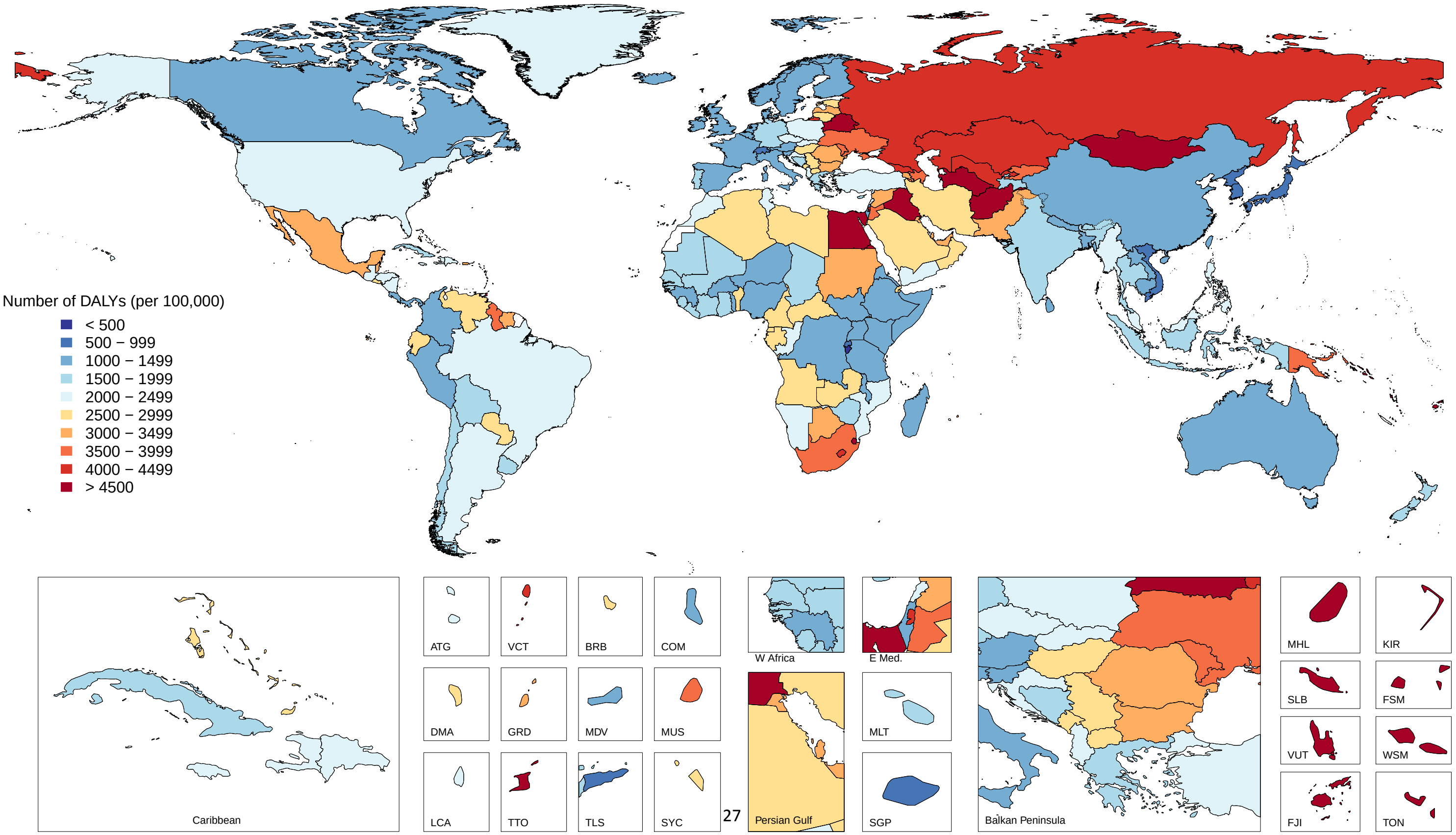

Figure S3d. Age−standardized DALY Rate Attributable to High−BMI for Females (ages >= 25) in 2015

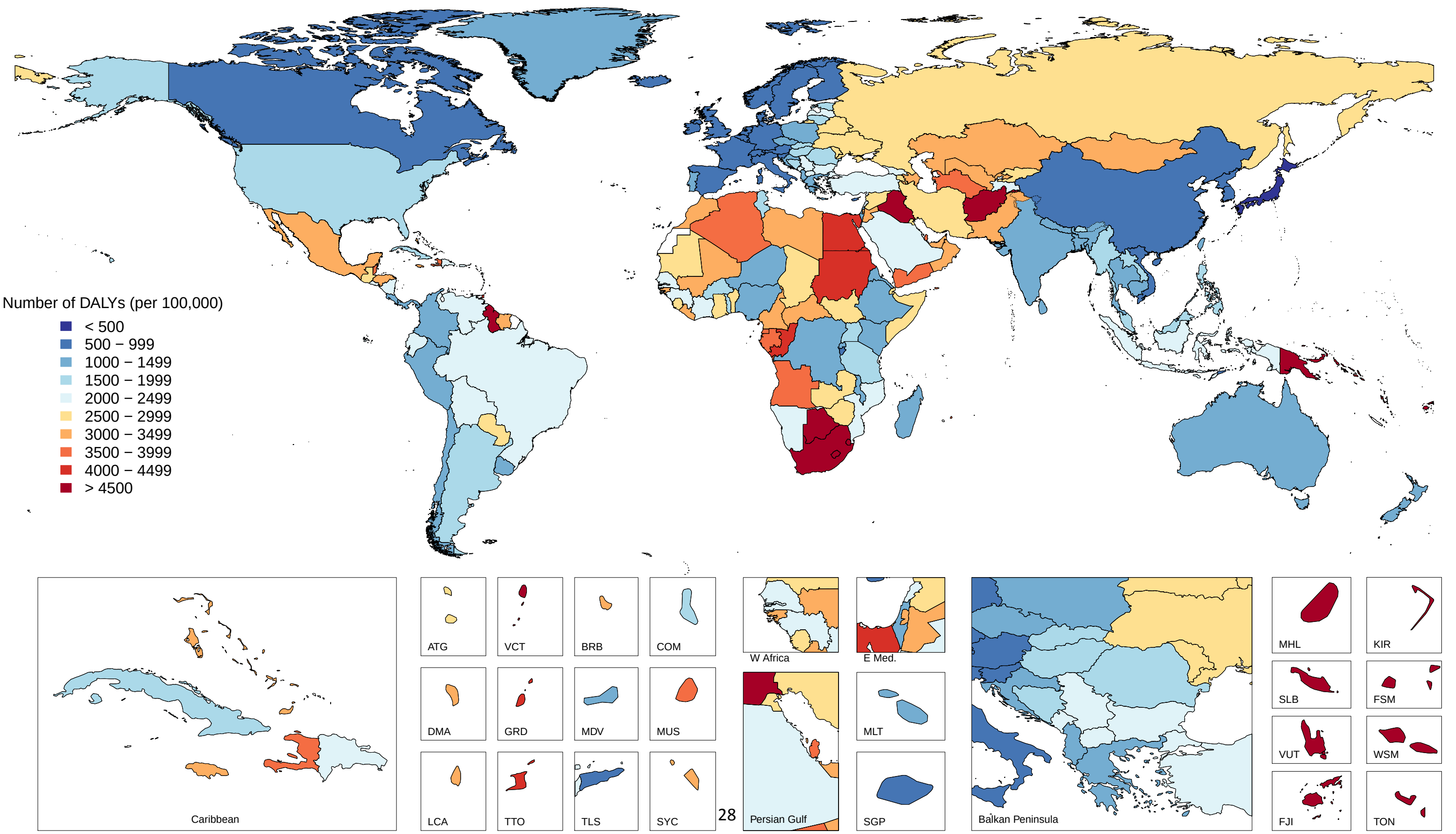

Figure S4. Selection process of scientific literature and survey data (PRISMA diagram)

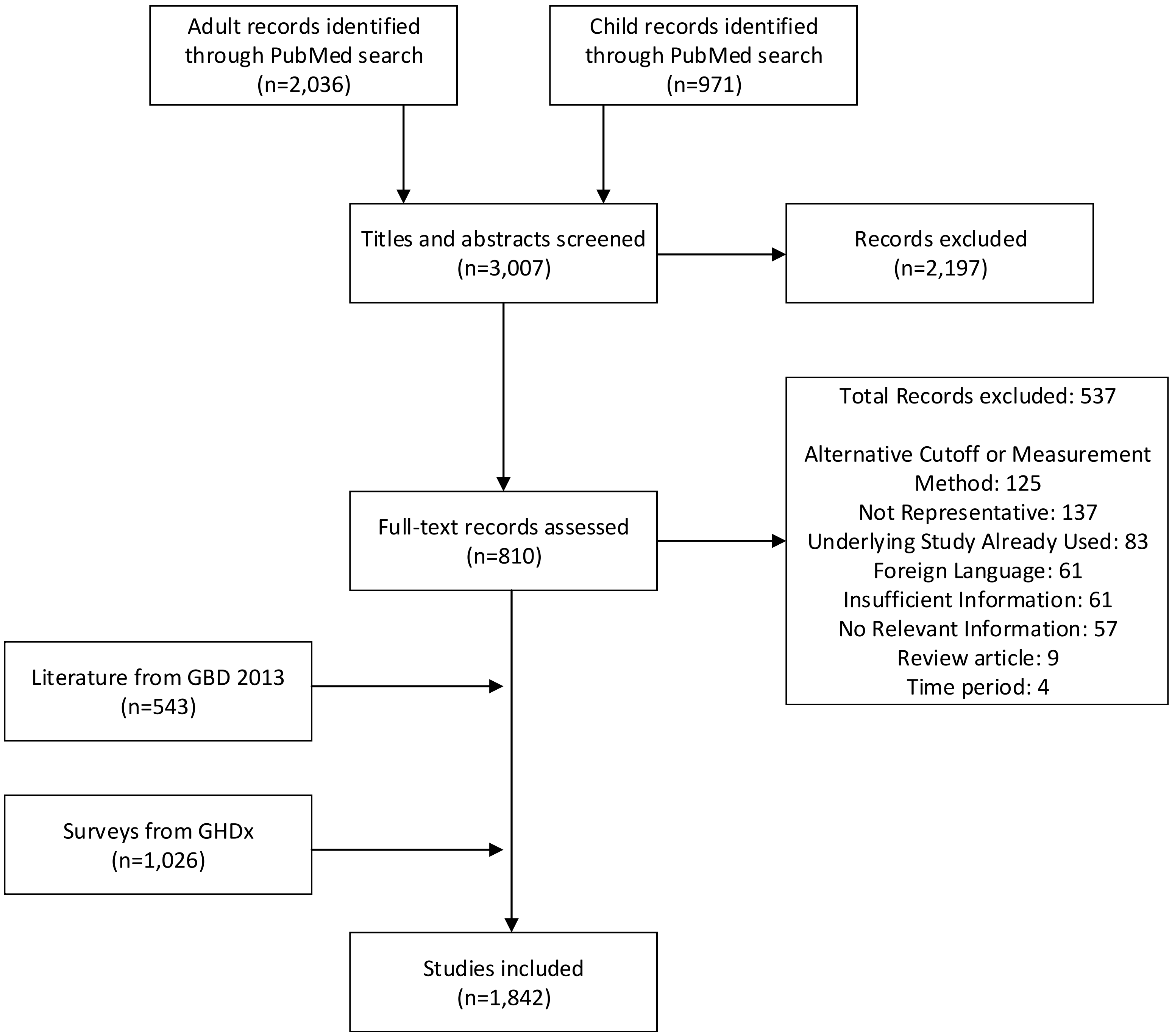

Figure S5. Process diagram for estimating the disease burden of high BMI

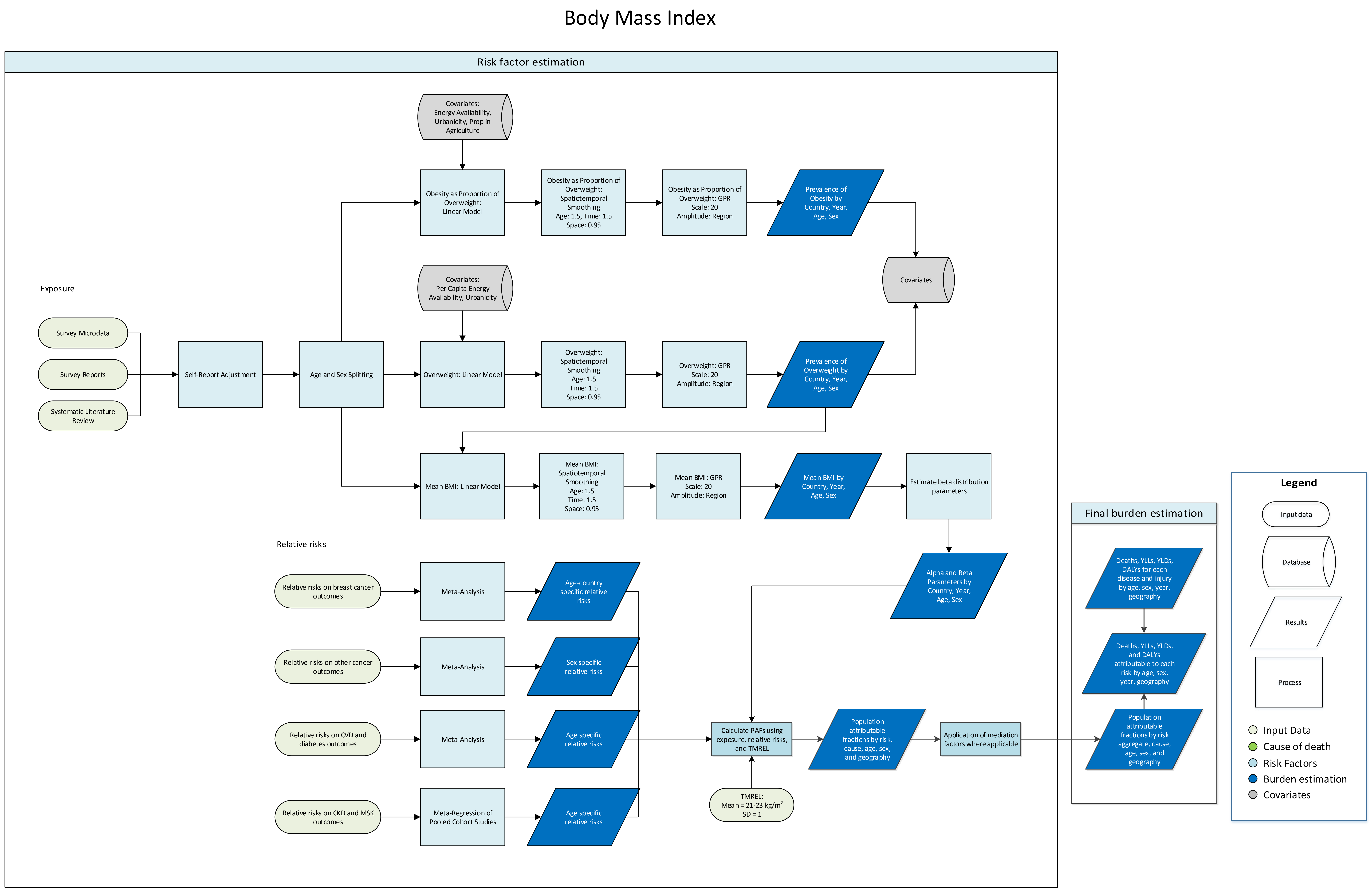

Table S1. **Epidemiological evidence supporting causality between high body mass index (BMI) and disease endpoints**.
To evaluate the magnitude of the effect sizes, we evaluated the RR comparing the 75th percentile with the 25th percentile of the exposure distribution at the global level. RR=relative risk.

Risk	Outcome	Prospective observational studies (n)*	Prospective observational studies with significant association in the opposite direction (%)	Lower limit of RR > 1.5	Dose-response relationship	Biologic plausibility†	Analogy‡
**Metabolic risks**							
High body-mass index	Oesophageal cancer	8	0	No	Yes	Yes	Yes
High body-mass index	Colon and rectum cancer	38	0	No	Yes	Yes	Yes
High body-mass index	Liver cancer	34	0	No	Yes	Yes	Yes
High body-mass index	Gallbladder and biliary tract cancer	10	0	No	Yes	Yes	Yes
High body-mass index	Pancreatic cancer	20	0	No	Yes	Yes	Yes
High body-mass index	Breast cancer (Post-menopause)	44	2	No	Yes	Yes	Yes
High body-mass index	Breast cancer (Pre-menopause)	25	8	No	Yes	Yes	No
High body-mass index	Uterine cancer	37	0	No	Yes	Yes	Yes
High body-mass index	Ovarian cancer	31	3	No	Yes	Yes	Yes
High body-mass index	Kidney cancer	28	0	No	Yes	Yes	Yes
High body-mass index	Thyroid cancer	16	0	No	Yes	Yes	Yes
High body-mass index	Leukaemia	17	0	No	Yes	Yes	Yes
High body-mass index	Ischaemic heart disease	129	..	No	Yes	Yes	Yes
High body-mass index	Ischemic stroke	102	..	No	Yes	Yes	Yes
High body-mass index	Hemorrhagic stroke	129	..	No	Yes	Yes	Yes
High body-mass index	Hypertensive heart disease	85	..	No	Yes	Yes	Yes
High body-mass index	Diabetes mellitus	85	..	Yes	Yes	Yes	No
High body-mass index	Chronic kidney disease	57	..	No	Yes	Yes	No
High body-mass index	Osteoarthritis	32	0	No	Yes	Yes	Yes
High body-mass index	Low back pain	5	0	No	Yes	Yes	Yes

● Yes
● No

Footnotes

*Prospective cohort studies or non-randomised interventions.

†Whether there is any biologic or mechanistic pathway that could potentially explain the relationship of the risk-outcome pair.

‡Whether the risk is associated with another outcome from the same category and there is evidence that it can cause the current outcome through the same pathway.

Table S2. Relative risks of high body mass index (BMI), by age and sex, for each disease endpoint															
Risk - Outcome	Category / Units	Morbidity / Mortality	Sex	25-29 years	30-34 years	35-39 years	40-44 years	45-49 years	50-54 years	55-59 years	60-64 years	65-69 years	70-74 years	75-79 years	80+ years
Oesophageal cancer	5 kg/m²	Both	Male	1·391 (1·076 to 1·758)	1·391 (1·076 to 1·758)	1·391 (1·076 to 1·758)	1·391 (1·076 to 1·758)	1·391 (1·076 to 1·758)	1·391 (1·076 to 1·758)	1·391 (1·076 to 1·758)	1·391 (1·076 to 1·758)	1·391 (1·076 to 1·758)	1·391 (1·076 to 1·758)	1·391 (1·076 to 1·758)	1·391 (1·076 to 1·758)
Oesophageal cancer	5 kg/m²	Both	Female	1·351 (1·012 to 1·745)	1·351 (1·012 to 1·745)	1·351 (1·012 to 1·745)	1·351 (1·012 to 1·745)	1·351 (1·012 to 1·745)	1·351 (1·012 to 1·745)	1·351 (1·012 to 1·745)	1·351 (1·012 to 1·745)	1·351 (1·012 to 1·745)	1·351 (1·012 to 1·745)	1·351 (1·012 to 1·745)	1·351 (1·012 to 1·745)
Colon and rectum cancer	5 kg/m²	Both	Male	1·177 (1·145 to 1·208)	1·177 (1·145 to 1·208)	1·177 (1·145 to 1·208)	1·177 (1·145 to 1·208)	1·177 (1·145 to 1·208)	1·177 (1·145 to 1·208)	1·177 (1·145 to 1·208)	1·177 (1·145 to 1·208)	1·177 (1·145 to 1·208)	1·177 (1·145 to 1·208)	1·177 (1·145 to 1·208)	1·177 (1·145 to 1·208)
Colon and rectum cancer	5 kg/m²	Both	Female	1·059 (1·031 to 1·083)	1·059 (1·031 to 1·083)	1·059 (1·031 to 1·083)	1·059 (1·031 to 1·083)	1·059 (1·031 to 1·083)	1·059 (1·031 to 1·083)	1·059 (1·031 to 1·083)	1·059 (1·031 to 1·083)	1·059 (1·031 to 1·083)	1·059 (1·031 to 1·083)	1·059 (1·031 to 1·083)	1·059 (1·031 to 1·083)
Gallbladder and biliary tract cancer	5 kg/m²	Both	Male	1·155 (1·033 to 1·282)	1·155 (1·033 to 1·282)	1·155 (1·033 to 1·282)	1·155 (1·033 to 1·282)	1·155 (1·033 to 1·282)	1·155 (1·033 to 1·282)	1·155 (1·033 to 1·282)	1·155 (1·033 to 1·282)	1·155 (1·033 to 1·282)	1·155 (1·033 to 1·282)	1·155 (1·033 to 1·282)	1·155 (1·033 to 1·282)
Gallbladder and biliary tract cancer	5 kg/m²	Both	Female	1·344 (1·223 to 1·478)	1·344 (1·223 to 1·478)	1·344 (1·223 to 1·478)	1·344 (1·223 to 1·478)	1·344 (1·223 to 1·478)	1·344 (1·223 to 1·478)	1·344 (1·223 to 1·478)	1·344 (1·223 to 1·478)	1·344 (1·223 to 1·478)	1·344 (1·223 to 1·478)	1·344 (1·223 to 1·478)	1·344 (1·223 to 1·478)
Pancreatic cancer	5 kg/m²	Both	Male	1·071 (0·999 to 1·154)	1·071 (0·999 to 1·154)	1·071 (0·999 to 1·154)	1·071 (0·999 to 1·154)	1·071 (0·999 to 1·154)	1·071 (0·999 to 1·154)	1·071 (0·999 to 1·154)	1·071 (0·999 to 1·154)	1·071 (0·999 to 1·154)	1·071 (0·999 to 1·154)	1·071 (0·999 to 1·154)	1·071 (0·999 to 1·154)
Pancreatic cancer	5 kg/m²	Both	Female	1·092 (1·037 to 1·144)	1·092 (1·037 to 1·144)	1·092 (1·037 to 1·144)	1·092 (1·037 to 1·144)	1·092 (1·037 to 1·144)	1·092 (1·037 to 1·144)	1·092 (1·037 to 1·144)	1·092 (1·037 to 1·144)	1·092 (1·037 to 1·144)	1·092 (1·037 to 1·144)	1·092 (1·037 to 1·144)	1·092 (1·037 to 1·144)
Breast cancer, post-menopausal	5 kg/m²	Both	Both	1·149 (1·036 to 1·268)	1·149 (1·036 to 1·268)	1·149 (1·036 to 1·268)	1·149 (1·036 to 1·268)	1·149 (1·036 to 1·268)	1·348 (1·128 to 1·615)	1·348 (1·128 to 1·615)	1·348 (1·128 to 1·615)	1·348 (1·128 to 1·615)	1·348 (1·128 to 1·615)	1·348 (1·128 to 1·615)	1·348 (1·128 to 1·615)
Uterine cancer	5 kg/m²	Both	Both	1·613 (1·543 to 1·681)	1·613 (1·543 to 1·681)	1·613 (1·543 to 1·681)	1·613 (1·543 to 1·681)	1·613 (1·543 to 1·681)	1·613 (1·543 to 1·681)	1·613 (1·543 to 1·681)	1·613 (1·543 to 1·681)	1·613 (1·543 to 1·681)	1·613 (1·543 to 1·681)	1·613 (1·543 to 1·681)	1·613 (1·543 to 1·681)
Ovarian cancer	5 kg/m²	Both	Both	1·038 (0·998 to 1·077)	1·038 (0·998 to 1·077)	1·038 (0·998 to 1·077)	1·038 (0·998 to 1·077)	1·038 (0·998 to 1·077)	1·038 (0·998 to 1·077)	1·038 (0·998 to 1·077)	1·038 (0·998 to 1·077)	1·038 (0·998 to 1·077)	1·038 (0·998 to 1·077)	1·038 (0·998 to 1·077)	1·038 (0·998 to 1·077)
Kidney cancer	5 kg/m²	Both	Male	1·240 (1·171 to 1·313)	1·240 (1·171 to 1·313)	1·240 (1·171 to 1·313)	1·240 (1·171 to 1·313)	1·240 (1·171 to 1·313)	1·240 (1·171 to 1·313)	1·240 (1·171 to 1·313)	1·240 (1·171 to 1·313)	1·240 (1·171 to 1·313)	1·240 (1·171 to 1·313)	1·240 (1·171 to 1·313)	1·240 (1·171 to 1·313)
Kidney cancer	5 kg/m²	Both	Female	1·320 (1·254 to 1·395)	1·320 (1·254 to 1·395)	1·320 (1·254 to 1·395)	1·320 (1·254 to 1·395)	1·320 (1·254 to 1·395)	1·320 (1·254 to 1·395)	1·320 (1·254 to 1·395)	1·320 (1·254 to 1·395)	1·320 (1·254 to 1·395)	1·320 (1·254 to 1·395)	1·320 (1·254 to 1·395)	1·320 (1·254 to 1·395)
Thyroid cancer	5 kg/m²	Both	Male	1·221 (1·067 to 1·382)	1·221 (1·067 to 1·382)	1·221 (1·067 to 1·382)	1·221 (1·067 to 1·382)	1·221 (1·067 to 1·382)	1·221 (1·067 to 1·382)	1·221 (1·067 to 1·382)	1·221 (1·067 to 1·382)	1·221 (1·067 to 1·382)	1·221 (1·067 to 1·382)	1·221 (1·067 to 1·382)	1·221 (1·067 to 1·382)
Thyroid cancer	5 kg/m²	Both	Female	1·136 (1·094 to 1·178)	1·136 (1·094 to 1·178)	1·136 (1·094 to 1·178)	1·136 (1·094 to 1·178)	1·136 (1·094 to 1·178)	1·136 (1·094 to 1·178)	1·136 (1·094 to 1·178)	1·136 (1·094 to 1·178)	1·136 (1·094 to 1·178)	1·136 (1·094 to 1·178)	1·136 (1·094 to 1·178)	1·136 (1·094 to 1·178)
Leukaemia	5 kg/m²	Both	Male	1·086 (1·053 to 1·119)	1·086 (1·053 to 1·119)	1·086 (1·053 to 1·119)	1·086 (1·053 to 1·119)	1·086 (1·053 to 1·119)	1·086 (1·053 to 1·119)	1·086 (1·053 to 1·119)	1·086 (1·053 to 1·119)	1·086 (1·053 to 1·119)	1·086 (1·053 to 1·119)	1·086 (1·053 to 1·119)	1·086 (1·053 to 1·119)
Leukaemia	5 kg/m²	Both	Female	1·131 (1·061 to 1·208)	1·131 (1·061 to 1·208)	1·131 (1·061 to 1·208)	1·131 (1·061 to 1·208)	1·131 (1·061 to 1·208)	1·131 (1·061 to 1·208)	1·131 (1·061 to 1·208)	1·131 (1·061 to 1·208)	1·131 (1·061 to 1·208)	1·131 (1·061 to 1·208)	1·131 (1·061 to 1·208)	1·131 (1·061 to 1·208)
Ischaemic heart disease	5 kg/m²	Both	Both	2·274 (1·257 to 3·686)	2·018 (1·296 to 3·109)	1·724 (1·532 to 1·932)	1·599 (1·418 to 1·785)	1·567 (1·457 to 1·680)	1·520 (1·417 to 1·631)	1·466 (1·372 to 1·557)	1·414 (1·324 to 1·504)	1·364 (1·287 to 1·448)	1·319 (1·242 to 1·400)	1·274 (1·187 to 1·365)	1·170 (1·091 to 1·253)
Ischaemic stroke	5 kg/m²	Both	Both	2·472 (1·399 to 3·980)	2·235 (1·454 to 3·334)	1·979 (1·694 to 2·313)	1·826 (1·600 to 2·076)	1·733 (1·581 to 1·898)	1·635 (1·479 to 1·796)	1·543 (1·441 to 1·653)	1·455 (1·345 to 1·566)	1·380 (1·310 to 1·458)	1·304 (1·233 to 1·376)	1·228 (1·159 to 1·305)	1·068 (0·992 to 1·143)
Hemorrhagic stroke	5 kg/m²	Both	Both	3·066 (1·750 to 5·337)	2·913 (1·860 to 4·399)	2·597 (1·974 to 3·387)	2·389 (1·869 to 3·002)	2·199 (1·821 to 2·673)	1·996 (1·625 to 2·419)	1·805 (1·573 to 2·060)	1·665 (1·437 to 1·933)	1·523 (1·377 to 1·684)	1·410 (1·265 to 1·571)	1·295 (1·162 to 1·439)	1·070 (0·928 to 1·220)
Hypertensive heart disease	5 kg/m²	Both	Both	3·122 (1·588 to 5·502)	3·000 (1·748 to 4·912)	2·769 (1·814 to 4·217)	2·573 (1·741 to 3·647)	2·407 (1·716 to 3·296)	2·281 (1·597 to 3·189)	2·159 (1·499 to 3·039)	2·035 (1·451 to 2·822)	1·955 (1·342 to 2·700)	1·860 (1·296 to 2·617)	1·792 (1·169 to 2·553)	1·697 (1·067 to 2·620)
Diabetes mellitus	5 kg/m²	Both	Both	3·547 (2·308 to 5·228)	3·455 (2·509 to 4·693)	3·349 (2·803 to 3·919)	3·160 (2·694 to 3·700)	2·864 (2·450 to 3·314)	2·624 (2·224 to 3·038)	2·417 (2·086 to 2·779)	2·215 (1·865 to 2·608)	2·046 (1·724 to 2·382)	1·896 (1·596 to 2·229)	1·740 (1·444 to 2·079)	1·461 (1·207 to 1·760)
Chronic kidney disease due to diabetes mellitus	5 kg/m²	Both	Both			1·746 (1·053 to 2·748)	1·746 (1·053 to 2·748)	1·746 (1·053 to 2·748)	1·746 (1·053 to 2·748)	1·746 (1·053 to 2·748)	2·036 (1·298 to 3·056)	2·036 (1·298 to 3·056)	1·621 (1·061 to 2·380)	1·621 (1·061 to 2·380)	1·431 (0·800 to 2·404)
Chronic kidney disease due to hypertension	5 kg/m²	Both	Both			1·763 (1·088 to 2·760)	1·763 (1·088 to 2·760)	1·763 (1·088 to 2·760)	1·763 (1·088 to 2·760)	1·763 (1·088 to 2·760)	2·044 (1·302 to 3·089)	2·044 (1·302 to 3·089)	1·605 (1·066 to 2·327)	1·605 (1·066 to 2·327)	1·437 (0·828 to 2·426)
Chronic kidney disease due to glomerulonephritis	5 kg/m²	Both	Both			1·742 (1·019 to 2·791)	1·742 (1·019 to 2·791)	1·742 (1·019 to 2·791)	1·742 (1·019 to 2·791)	1·742 (1·019 to 2·791)	2·044 (1·254 to 3·155)	2·044 (1·254 to 3·155)	1·604 (1·108 to 2·255)	1·604 (1·108 to 2·255)	1·452 (0·851 to 2·350)
Chronic kidney disease due to other causes	5 kg/m²	Both	Both			1·732 (1·047 to 2·684)	1·732 (1·047 to 2·684)	1·732 (1·047 to 2·684)	1·732 (1·047 to 2·684)	1·732 (1·047 to 2·684)	2·032 (1·214 to 3·105)	2·032 (1·214 to 3·105)	1·625 (1·068 to 2·368)	1·625 (1·068 to 2·368)	1·433 (0·776 to 2·345)
Low back pain	5 kg/m²	Morbidity	Both	1·100 (1·073 to 1·126)	1·100 (1·073 to 1·127)	1·101 (1·076 to 1·128)	1·100 (1·074 to 1·126)	1·099 (1·075 to 1·123)	1·100 (1·075 to 1·128)	1·100 (1·075 to 1·126)	1·101 (1·077 to 1·126)	1·100 (1·075 to 1·126)	1·100 (1·076 to 1·124)	1·100 (1·075 to 1·124)	1·100 (1·074 to 1·125)

Table S3. The age-standardized prevalence of overweight and obesity among children and adults by sex in 1980 and 2015 among 195 countries.

Country	SDI Level	1980				2015				1980				2015			
		Adult (ages >= 20) Prevalence of Overweight		Adult (ages >= 20) Prevalence of Obesity		Adult (ages >= 20) Prevalence of Overweight		Adult (ages >= 20) Prevalence of Obesity		Child (ages 2 - 19) Prevalence of Overweight		Child (ages 2 - 19) Prevalence of Obesity		Child (ages 2 - 19) Prevalence of Overweight		Child (ages 2 - 19) Prevalence of Obesity	
		Males	*Females*	*Males*	*Females*	*Males*	*Females*	*Males*	*Females*	*Males*	*Females*	*Males*	*Females*	*Males*	*Females*	*Males*	*Females*
Afghanistan	Low SDI	**29.88 (27.41 - 32.59)**	**27.25 (24.88 - 29.28)**	**12.72 (10.9 - 14.59)**	**17.7 (15.61 - 20.11)**	**23.1 (20.94 - 25.37)**	**22.04 (21.08 - 22.95)**	**7.87 (6.81 - 8.95)**	**12.12 (11.14 - 13.14)**	**7.86 (5.8 - 10.17)**	**14.08 (11.18 - 17.28)**	**4.02 (2.77 - 5.58)**	**6.23 (4.65 - 8.19)**	**4.86 (3.71 - 6.29)**	**9.34 (7.24 - 11.59)**	**2.13 (1.47 - 2.99)**	**4.14 (2.94 - 5.53)**
Albania	High-middle SDI	33.96 (30.77 - 36.79)	27.45 (24.92 - 30.17)	5.77 (4.51 - 7.18)	7.97 (6.43 - 9.69)	49.98 (48 - 51.49)	35.52 (33.24 - 37.03)	11.79 (9.88 - 13.98)	14.89 (12.7 - 17.42)	10.53 (8.77 - 11.81)	11.46 (9.25 - 13.81)	6.97 (4.93 - 9.52)	7.29 (5.14 - 9.77)	18.5 (15.87 - 21.28)	13.6 (11.44 - 15.91)	7.71 (5.58 - 10.3)	7.1 (4.99 - 9.45)
Algeria	Middle SDI	12.5 (10.74 - 14.46)	16.39 (14.57 - 18.26)	2.85 (2.32 - 3.43)	10.21 (8.59 - 11.94)	32.08 (29.66 - 34.14)	21.92 (20.49 - 23.35)	18.06 (16.04 - 20.06)	32.66 (29.85 - 35.76)	2.46 (1.79 - 3.28)	5.23 (3.87 - 6.91)	0.46 (0.31 - 0.67)	1.25 (0.85 - 1.78)	11.04 (8.52 - 13.84)	12.95 (9.96 - 16.02)	3.83 (2.74 - 5.19)	6.17 (4.41 - 8.31)
American Samoa	High-middle SDI	32.44 (32.14 - 32.2)	23.14 (23.88 - 21.87)	45.24 (41.46 - 49.02)	62.22 (57.53 - 66.69)	30.49 (30.9 - 30.04)	21.29 (22.58 - 20.08)	55.33 (51.98 - 58.71)	68.35 (63.83 - 72.28)	17.53 (14.91 - 19.62)	19.31 (18.03 - 20.5)	29.37 (22.39 - 36.53)	35.93 (27.78 - 44.51)	19.65 (17.83 - 21.17)	20.08 (19.28 - 20.21)	37.97 (30.38 - 45.88)	42.9 (34.33 - 51.97)
Andorra	High SDI	38.41 (35.73 - 41.03)	30.01 (27.75 - 32.14)	6.99 (5.78 - 8.62)	8.27 (6.95 - 9.87)	26.8 (24.96 - 28.73)	30.12 (28 - 32.48)	12.49 (10.58 - 14.57)	8.94 (7.44 - 10.6)	7.8 (5.89 - 9.87)	7.84 (6.09 - 9.77)	7.87 (5.1 - 11.53)	6.88 (4.72 - 9.51)	7.67 (5.81 - 9.71)	9.75 (7.65 - 12.32)	10.59 (7.12 - 14.84)	8.76 (6.13 - 12.11)
Angola	Low-middle SDI	10.96 (10.18 - 11.83)	10.8 (9.71 - 12)	1.36 (1.08 - 1.72)	2.8 (2.34 - 3.34)	23.05 (22.07 - 23.74)	23.31 (21.82 - 24.9)	6.67 (5.49 - 8.07)	14.11 (12.43 - 16)	5.38 (4.26 - 6.59)	7.44 (5.81 - 9.16)	2.26 (1.53 - 3.29)	2.29 (1.59 - 3.18)	12.58 (10.57 - 14.91)	14.05 (11.61 - 16.85)	6.75 (4.84 - 9.1)	6.17 (4.58 - 8.16)
Antigua and Barbuda	High SDI	17.54 (15.48 - 19.49)	27.47 (25.3 - 29.77)	4.96 (4.2 - 5.85)	13.28 (11.82 - 14.9)	31.65 (29.49 - 33.45)	31.43 (30.13 - 32.91)	13.64 (11.89 - 15.53)	26.15 (23.91 - 28.34)	5.94 (4.77 - 7.28)	8.28 (6.84 - 10.1)	1.84 (1.31 - 2.54)	2.26 (1.68 - 2.93)	8.19 (6.67 - 10.1)	10.92 (9.26 - 12.79)	3.98 (2.88 - 5.45)	4.59 (3.44 - 6)
Argentina	High-middle SDI	42.05 (38.64 - 44.9)	32.25 (30.3 - 34.06)	17.59 (15.34 - 20.14)	21.33 (18.61 - 24.09)	43.51 (40.77 - 45.96)	33.43 (31.9 - 34.81)	20.94 (18.4 - 23.78)	25.14 (22.39 - 28.07)	16 (11.91 - 20.82)	13.18 (10.61 - 15.64)	5.51 (3.7 - 7.99)	4.99 (3.52 - 6.67)	20.74 (15.94 - 25.37)	14.82 (12.41 - 17.43)	7.18 (4.96 - 9.84)	5.53 (3.91 - 7.34)
Armenia	High-middle SDI	29.44 (27.3 - 31.38)	32.39 (31.23 - 33.72)	7.79 (6.41 - 9.48)	19.95 (18.17 - 21.73)	37.45 (36.28 - 38.32)	34.05 (33.17 - 34.55)	14.06 (11.91 - 16.53)	28.05 (26.22 - 30.25)	11.06 (8.86 - 13.26)	13.31 (11.04 - 15.8)	4.54 (3.11 - 6.27)	3.47 (2.59 - 4.61)	15.72 (12.67 - 18.37)	13.29 (11.2 - 15.47)	7.39 (5.23 - 10.04)	5.57 (4.24 - 7.16)
Australia	High SDI	41.96 (39.12 - 44.17)	26.34 (23.86 - 28.63)	15.69 (13.57 - 18.15)	18.28 (15.76 - 21.1)	42.12 (40.9 - 43.18)	28.13 (26.93 - 29.02)	26.02 (23.6 - 28.49)	30.74 (27.78 - 33.72)	13.7 (11.05 - 16.92)	13.86 (10.57 - 17.35)	3.92 (2.9 - 5.34)	4.03 (2.9 - 5.55)	17.93 (15.13 - 20.85)	16.51 (13.52 - 19.65)	7.8 (6 - 9.87)	7.54 (5.57 - 9.72)
Austria	High SDI	38.11 (35.9 - 39.96)	18.68 (17.37 - 19.67)	8.56 (7.21 - 10.11)	12.34 (10.63 - 14.18)	46.07 (44.28 - 47.66)	27.3 (26.1 - 28.66)	14.05 (12.07 - 16.23)	16.46 (14.38 - 18.58)	11.43 (8.27 - 14.7)	8.08 (6.02 - 10.64)	3.89 (2.26 - 6)	2.59 (1.56 - 3.95)	8.69 (6.72 - 10.89)	7.56 (5.88 - 9.65)	9.85 (6.83 - 13.61)	7.06 (4.87 - 9.76)
Azerbaijan	High-middle SDI	37.14 (34.57 - 39.39)	31.7 (30.67 - 32.76)	5.15 (4.18 - 6.27)	21.5 (19.61 - 23.42)	51.26 (49.83 - 52.91)	33.49 (33.26 - 33.63)	10.46 (8.77 - 12.3)	31.14 (29.29 - 32.92)	7.06 (5.47 - 8.48)	9.73 (8.13 - 11.52)	3.51 (2.28 - 4.94)	3.54 (2.69 - 4.56)	15.39 (12.97 - 18.16)	14.13 (11.88 - 16.87)	8.72 (6.35 - 11.57)	6.59 (5.04 - 8.33)
Bahrain	High-middle SDI	35.77 (33.35 - 37.92)	31.27 (29.68 - 32.57)	18.55 (16.46 - 20.73)	30.95 (28.04 - 33.99)	38.1 (36.72 - 38.91)	31.98 (31.28 - 32.61)	28.07 (25.84 - 30.67)	38.21 (35.29 - 41.22)	11.23 (8.73 - 14.06)	15.94 (12.91 - 19.1)	7.78 (5.59 - 10.2)	9.33 (7.02 - 12.21)	12.01 (9.39 - 14.93)	12.6 (9.9 - 15.69)	7.69 (5.56 - 10.35)	7.82 (5.88 - 10.41)
Bangladesh	Low-middle SDI	5.07 (4 - 6.3)	5.74 (4.56 - 7.13)	0.51 (0.36 - 0.73)	0.64 (0.47 - 0.85)	14.84 (12.57 - 17.31)	15.81 (13.37 - 18.36)	2.28 (1.76 - 3)	3.65 (2.86 - 4.54)	1.4 (0.99 - 2)	1.41 (0.95 - 2.04)	0.79 (0.47 - 1.25)	0.77 (0.45 - 1.28)	2.48 (1.76 - 3.48)	2.88 (1.97 - 3.98)	1.25 (0.79 - 1.87)	1.24 (0.76 - 1.91)
Barbados	High-middle SDI	33.23 (31.09 - 35.64)	40.83 (39.81 - 41.93)	12.33 (10.66 - 14.05)	25.17 (22.96 - 27.35)	34.19 (32.64 - 35.75)	31.53 (30.97 - 32.03)	22.33 (20.56 - 24.19)	40.14 (38.06 - 42.31)	12.61 (10.5 - 15.07)	15.87 (13.87 - 17.84)	6.84 (5.02 - 9.05)	9.56 (7.63 - 11.98)	11.47 (9.43 - 13.65)	19.31 (17.18 - 21.52)	10.73 (8.06 - 13.74)	11.09 (8.76 - 13.56)
Belarus	High SDI	38.49 (37.34 - 39.66)	30.51 (29.67 - 31.08)	12.14 (10.55 - 13.72)	21.55 (19.64 - 23.69)	43.62 (43.06 - 44.29)	30.72 (30.16 - 31.16)	18.44 (16.33 - 20.58)	27.07 (24.8 - 29.37)	8.15 (6.79 - 9.58)	8.92 (7.37 - 10.73)	2.15 (1.56 - 2.98)	2.53 (1.85 - 3.4)	11.83 (10.09 - 13.85)	11.16 (9.43 - 13.15)	3.44 (2.47 - 4.61)	3.59 (2.64 - 4.61)
Belgium	High SDI	42.43 (40.03 - 44.86)	26.83 (25.23 - 28.42)	8.71 (7.22 - 10.34)	12.98 (11.01 - 15.11)	44.7 (42.84 - 46.27)	27.7 (26.43 - 28.52)	14.09 (12.17 - 16.07)	18.28 (16.07 - 20.71)	15.05 (11.05 - 19.9)	10.54 (7.98 - 13.74)	3.25 (1.98 - 4.95)	4.17 (2.77 - 6.07)	15.17 (11.43 - 19.44)	13.1 (10.25 - 16.47)	5.02 (3.16 - 7.4)	4.29 (2.83 - 6.29)
Belize	Middle SDI	28.38 (26.28 - 30.52)	34.81 (33.69 - 35.79)	11.84 (10.31 - 13.48)	28.85 (26.39 - 31.16)	35.15 (33.13 - 36.79)	33.9 (33.16 - 34.35)	19.09 (17.15 - 21.17)	37.79 (35.31 - 40.36)	10.02 (7.9 - 12.05)	14.71 (12.31 - 17.05)	4.11 (2.91 - 5.57)	6.31 (4.94 - 7.89)	11.26 (9.14 - 13.54)	14.49 (12.33 - 16.88)	5.3 (3.88 - 6.96)	7.73 (6.02 - 9.84)
Benin	Low SDI	7.86 (6.69 - 9.11)	11.05 (9.7 - 12.59)	0.95 (0.71 - 1.24)	3.12 (2.42 - 3.91)	26.18 (24.27 - 28.25)	24.53 (23.38 - 25.79)	10.71 (8.8 - 12.81)	15.34 (13.21 - 17.58)	2.24 (1.58 - 3.1)	2.73 (2 - 3.67)	0.58 (0.35 - 0.92)	0.55 (0.32 - 0.9)	10.31 (8.05 - 12.63)	13.8 (11.09 - 16.43)	6.26 (4.14 - 8.86)	6.27 (4.06 - 8.98)
Bermuda	High SDI	24.05 (21.73 - 26.48)	29.18 (27.19 - 31.12)	7.19 (6.13 - 8.28)	15.41 (13.77 - 17.07)	38.93 (36.68 - 40.74)	32.51 (31.11 - 33.31)	17.15 (15.28 - 19.24)	29.45 (27.05 - 31.86)	9.53 (7.6 - 11.48)	10.43 (8.65 - 12.48)	2.83 (2.01 - 3.86)	2.87 (2.17 - 3.8)	13.49 (10.85 - 16.04)	14.24 (12.16 - 16.7)	6.2 (4.51 - 8.17)	5.95 (4.53 - 7.62)
Bhutan	Low-middle SDI	10.89 (8.83 - 13.34)	22.31 (18.7 - 26)	1.32 (0.96 - 1.76)	3.73 (2.88 - 4.8)	25.65 (24.64 - 26.78)	37.26 (36.04 - 38.44)	5.08 (4.72 - 5.49)	10.52 (9.86 - 11.25)	2.47 (1.73 - 3.43)	4.26 (2.86 - 5.73)	1.04 (0.64 - 1.6)	1.49 (0.92 - 2.29)	7.77 (5.5 - 10.28)	10.12 (7.6 - 12.99)	3.54 (2.18 - 5.18)	4.16 (2.73 - 6.16)
Bolivia	Middle SDI	25.3 (24.22 - 26.2)	29.79 (28.66 - 30.85)	11.74 (9.76 - 14.08)	12.22 (11.17 - 13.31)	36.5 (35.51 - 37.51)	37.01 (36.7 - 37.35)	13.37 (11.06 - 15.91)	25.11 (23.6 - 26.61)	11.88 (10.32 - 13.38)	15.75 (14.13 - 17.62)	3.08 (2.22 - 4.27)	3.34 (2.69 - 4.03)	15.34 (13.31 - 17.27)	20.95 (18.7 - 23.23)	3.72 (2.68 - 5.09)	4.52 (3.67 - 5.47)
Bosnia and Herzegovina	High-middle SDI	31.66 (29.22 - 34.11)	27.58 (25.83 - 29.3)	7.21 (5.79 - 8.71)	11.52 (9.54 - 13.75)	43.58 (41.62 - 45.16)	31.89 (30.21 - 33.07)	13.85 (11.51 - 16.25)	18.32 (15.82 - 21.18)	4.78 (3.87 - 5.89)	7.05 (5.45 - 8.57)	2.41 (1.5 - 3.61)	3.42 (2.22 - 5.04)	9.43 (7.99 - 10.95)	11.34 (9.24 - 13.75)	6.29 (4.33 - 8.68)	6.82 (4.72 - 9.21)
Botswana	Middle SDI	5.45 (4.4 - 6.64)	7.43 (6.75 - 8.21)	1.58 (1.16 - 2.1)	9.86 (8.71 - 11.2)	14.42 (12.35 - 16.57)	10.38 (9.76 - 11.04)	7.99 (6.56 - 9.64)	26.36 (24.18 - 28.62)	3.86 (2.73 - 5.28)	3.92 (3.24 - 4.62)	1.39 (0.82 - 2.28)	1.51 (1.07 - 2.05)	10.52 (7.88 - 13.23)	8.72 (7.82 - 9.9)	6.01 (3.98 - 8.73)	6.1 (4.6 - 7.8)
Brazil	Middle SDI	26.3 (25.38 - 27.19)	28.25 (27.61 - 28.82)	6.41 (5.84 - 7.07)	12.22 (11.44 - 13.08)	37.59 (37.3 - 37.67)	33.25 (33.1 - 33.35)	18.95 (17.98 - 20.11)	25.42 (24.48 - 26.46)	7.81 (7.08 - 8.53)	9.46 (8.73 - 10.26)	2.12 (1.8 - 2.51)	2.42 (2.13 - 2.77)	17.53 (16.65 - 18.41)	15.66 (14.59 - 16.87)	9.8 (8.61 - 11.14)	8.12 (7.25 - 9)
Brunei	High SDI	16.83 (15.63 - 17.9)	20.51 (18.93 - 21.92)	2.15 (1.65 - 2.76)	4.98 (4.16 - 5.97)	25.6 (24.54 - 26.38)	24.07 (22.46 - 25.64)	5.87 (4.61 - 7.38)	8.3 (6.99 - 9.62)	5.19 (4.38 - 6.01)	7.61 (6.17 - 9.25)	1.97 (1.38 - 2.78)	2.21 (1.5 - 3.18)	9.64 (8.51 - 10.75)	11.71 (9.83 - 13.46)	4.87 (3.37 - 6.69)	4.82 (3.38 - 6.68)
Bulgaria	High-middle SDI	49.09 (48.68 - 49.15)	30.65 (30.04 - 31.86)	21 (17.77 - 24.55)	24.1 (20.78 - 27.31)	44.5 (42.55 - 46.13)	29.41 (28.3 - 30.34)	13.5 (11.62 - 15.78)	18.33 (15.96 - 20.79)	23.89 (20.99 - 26.36)	15.96 (13.44 - 18.41)	10.21 (7.26 - 13.69)	8.12 (5.68 - 11.05)	19.11 (16.13 - 21.89)	14.97 (11.95 - 17.58)	6.65 (4.53 - 9.3)	6.04 (4.24 - 8.08)
Burkina Faso	Low SDI	2.52 (2.1 - 3)	4.37 (3.72 - 5.1)	0.14 (0.1 - 0.19)	0.42 (0.32 - 0.54)	21.2 (19.2 - 23.12)	20.1 (18.6 - 21.87)	5.2 (4.24 - 6.17)	9.6 (8.06 - 11.16)	1.54 (1.08 - 2.13)	1.99 (1.42 - 2.75)	0.43 (0.25 - 0.69)	0.43 (0.25 - 0.69)	9.16 (7 - 11.51)	6.84 (5.29 - 8.47)	4.98 (3.33 - 7.09)	4.45 (2.94 - 6.27)
Burundi	Low SDI	6.83 (5.27 - 8.94)	10.03 (8.51 - 11.6)	1.64 (0.9 - 2.67)	3.98 (2.96 - 5.25)	3.68 (2.62 - 4.94)	7.07 (6.03 - 8.23)	0.51 (0.27 - 0.89)	2.13 (1.59 - 2.79)	3.35 (1.91 - 5.18)	4.24 (3.04 - 5.96)	1.09 (0.5 - 2.05)	0.94 (0.54 - 1.58)	3.13 (1.86 - 4.79)	5.17 (3.68 - 7.13)	0.72 (0.31 - 1.33)	0.81 (0.45 - 1.39)
Cambodia	Low-middle SDI	5.76 (4.66 - 7.08)	8.63 (7.35 - 9.99)	0.46 (0.34 - 0.63)	1.22 (0.93 - 1.61)	13.58 (11.72 - 15.67)	15.23 (13.71 - 16.96)	1.75 (1.38 - 2.15)	2.92 (2.39 - 3.53)	1.22 (0.86 - 1.72)	1.52 (1.06 - 2.03)	0.52 (0.32 - 0.82)	0.64 (0.39 - 0.98)	2.5 (1.75 - 3.31)	2.55 (1.82 - 3.38)	1.25 (0.8 - 1.82)	1.19 (0.75 - 1.72)
Cameroon	Low-middle SDI	17.94 (15.88 - 20.31)	23.38 (21.25 - 25.61)	3.07 (2.37 - 3.87)	10.16 (8.3 - 12.12)	30.12 (28.09 - 32.22)	27.71 (26.15 - 28.79)	10.92 (8.93 - 13.04)	21.95 (19.33 - 24.74)	5.65 (4.14 - 7.48)	7.88 (5.93 - 10.06)	1.52 (0.91 - 2.42)	1.68 (1.07 - 2.5)	10.21 (7.55 - 13.17)	14.37 (11.06 - 17.67)	4.04 (2.61 - 5.73)	3.98 (2.64 - 5.64)
Canada	High SDI	41.62 (40.03 - 43.01)	26.56 (25.55 - 27.77)	10.93 (8.9 - 13.02)	13.29 (11.43 - 15.33)	42.65 (42.12 - 43.18)	30.65 (29.83 - 31.25)	19.79 (17.18 - 22.59)	20.77 (18.71 - 23)	12.31 (10.02 - 14.82)	10.78 (9.01 - 12.73)	5.15 (3.58 - 7.14)	5.14 (3.68 - 6.93)	15.12 (13.44 - 16.94)	12.73 (10.95 - 14.49)	11.88 (8.7 - 15.17)	9.84 (7.62 - 12.21)
Cape Verde	Low-middle SDI	8.27 (7.09 - 9.72)	15.35 (13.34 - 17.33)	1.03 (0.77 - 1.33)	3.93 (3.1 - 4.92)	26.91 (24.31 - 29.2)	28.34 (26.72 - 30.04)	8.1 (6.61 - 9.81)	16.51 (13.84 - 19.31)	2.08 (1.49 - 2.86)	4.51 (3.33 - 6.02)	0.54 (0.32 - 0.86)	1.04 (0.65 - 1.6)	8.62 (6.8 - 10.91)	11.91 (9.56 - 14.46)	3.83 (2.51 - 5.64)	5.11 (3.34 - 7.39)
Central African Republic	Low SDI	18.26 (17.51 - 18.94)	11.99 (10.71 - 13.4)	5.1 (4.17 - 6.22)	2.59 (2.11 - 3.23)	13.83 (13.02 - 14.46)	15.98 (14.75 - 17.26)	4.22 (3.52 - 4.94)	8.41 (7.17 - 9.83)	9.87 (7.89 - 11.76)	8.64 (7.03 - 10.53)	5.56 (3.91 - 7.59)	3.45 (2.32 - 4.83)	5.62 (4.4 - 6.97)	6.96 (5.56 - 8.45)	1.42 (0.95 - 2.06)	1.9 (1.28 - 2.72)
Chad	Low SDI	10.72 (9.31 - 12.43)	9.21 (8.01 - 10.48)	2.1 (1.62 - 2.65)	1.96 (1.54 - 2.49)	22.07 (20.12 - 23.97)	20.25 (18.54 - 21.57)	8.78 (7.22 - 10.44)	13.97 (12.05 - 16.27)	2.66 (1.93 - 3.46)	3.24 (2.45 - 4.27)	0.78 (0.47 - 1.22)	0.81 (0.49 - 1.26)	5.83 (4.28 - 7.74)	5.81 (4.27 - 7.48)	2.52 (1.59 - 3.78)	2.36 (1.52 - 3.5)

Table S3. The age-standardized prevalence of overweight and obesity among children and adults by sex in 1980 and 2015 among 195 countries.

Country	SDI Level	1980				2015				1980				2015			
		Adult (ages >= 20) Prevalence of Overweight		Adult (ages >= 20) Prevalence of Obesity		Adult (ages >= 20) Prevalence of Overweight		Adult (ages >= 20) Prevalence of Obesity		Child (ages 2 - 19) Prevalence of Overweight		Child (ages 2 - 19) Prevalence of Obesity		Child (ages 2 - 19) Prevalence of Overweight		Child (ages 2 - 19) Prevalence of Obesity	
		Males	*Females*	*Males*	*Females*	*Males*	*Females*	*Males*	*Females*	*Males*	*Females*	*Males*	*Females*	*Males*	*Females*	*Males*	*Females*
Chile	High-middle SDI	41.36 (38.23 - 44.54)	33.61 (31.56 - 35.26)	16 (13.81 - 18.23)	22.84 (20.12 - 25.56)	47.27 (45.16 - 48.98)	35.44 (34.44 - 36.32)	21.08 (18.74 - 23.46)	28.57 (26.12 - 31.25)	13.8 (10.42 - 17.66)	13.74 (11.62 - 16.1)	7.1 (4.7 - 9.93)	6.84 (4.95 - 9.08)	25.74 (20.53 - 30.82)	15.4 (12.88 - 17.73)	10.66 (7.72 - 14.24)	10.14 (7.75 - 12.99)
China	Middle SDI	5.23 (5.05 - 5.43)	9.28 (9.01 - 9.57)	0.33 (0.3 - 0.36)	0.9 (0.83 - 0.98)	25.71 (25.37 - 26.05)	22.78 (22.51 - 23.07)	5.02 (4.8 - 5.26)	5.51 (5.31 - 5.73)	2.54 (2.39 - 2.7)	2.87 (2.71 - 3.05)	0.8 (0.7 - 0.92)	0.83 (0.73 - 0.95)	12.34 (11.76 - 12.92)	9.82 (9.46 - 10.23)	5.91 (5.36 - 6.46)	4.24 (3.81 - 4.69)
Colombia	High-middle SDI	17.83 (15.34 - 20.42)	26.84 (24.4 - 29.59)	3.58 (2.68 - 4.74)	9 (7.33 - 10.82)	36.72 (34.53 - 38.42)	34.32 (33.01 - 35.55)	13.68 (11.44 - 16.02)	21.05 (18.57 - 23.63)	5 (3.55 - 6.63)	8.63 (6.37 - 11.46)	0.81 (0.46 - 1.3)	0.86 (0.54 - 1.33)	9.92 (7.45 - 12.69)	12.4 (9.54 - 15.31)	3.14 (2.01 - 4.63)	3.24 (2.13 - 4.75)
Comoros	Low SDI	13.45 (10.75 - 16.19)	17.48 (15.18 - 19.87)	3.93 (2.25 - 6.12)	4.7 (3.42 - 6.22)	17.1 (13.65 - 20.6)	25.63 (23.43 - 27.76)	4.5 (2.73 - 6.79)	12.32 (9.55 - 15.33)	9.71 (6.37 - 13.73)	12.07 (8.77 - 15.44)	3.93 (1.92 - 7.13)	2.83 (1.67 - 4.44)	10.66 (7.02 - 14.39)	14.78 (11.05 - 19.05)	4.33 (2.13 - 8.24)	4.05 (2.27 - 6.46)
Congo	Low-middle SDI	18.78 (17.82 - 19.74)	16.42 (14.86 - 18.03)	2.93 (2.33 - 3.62)	6.21 (5.27 - 7.23)	26.98 (26.01 - 27.89)	22.36 (21.17 - 23.56)	6.03 (4.9 - 7.27)	14.57 (12.99 - 16.23)	4.24 (3.37 - 5.24)	8.87 (7.1 - 11.02)	2.6 (1.78 - 3.68)	2.21 (1.56 - 3.1)	5.24 (4.28 - 6.43)	8.93 (6.85 - 10.9)	3.28 (2.37 - 4.5)	3 (2.11 - 4.14)
Costa Rica	High-middle SDI	25.43 (22.2 - 28.78)	28.52 (25.97 - 31.24)	5.56 (4.25 - 7.2)	12.66 (10.39 - 15.13)	42.82 (40.86 - 44.57)	35.2 (34.05 - 36.28)	15.78 (12.63 - 18.96)	26.11 (22.61 - 29.67)	8.08 (6.33 - 9.72)	9.18 (6.99 - 11.68)	3.66 (2.26 - 5.75)	3.85 (2.57 - 5.36)	17.39 (14.23 - 20.31)	17.71 (14.28 - 21.45)	9.57 (6.34 - 13.44)	9.38 (6.56 - 12.57)
Cote d'Ivoire	Low SDI	12.05 (10.4 - 13.79)	13.93 (12.13 - 15.85)	1.35 (1.02 - 1.71)	4.52 (3.56 - 5.5)	26.24 (23.77 - 28.8)	23.27 (21.39 - 25.28)	5.05 (3.99 - 6.33)	10.6 (8.79 - 12.52)	4.49 (3.23 - 6.12)	6.53 (4.93 - 8.37)	1.38 (0.82 - 2.12)	1.78 (1.13 - 2.72)	8 (5.99 - 10.53)	10.62 (7.92 - 13.51)	2.29 (1.47 - 3.45)	2.43 (1.58 - 3.6)
Croatia	High-middle SDI	40.53 (38.67 - 41.93)	29.38 (27.65 - 31.37)	13.17 (10.8 - 15.85)	13.61 (11.49 - 15.89)	46.7 (45.28 - 47.8)	31.27 (30.07 - 32.38)	16.42 (14.06 - 19.05)	14.44 (12.35 - 16.54)	12.98 (10.86 - 15.49)	7.4 (5.74 - 9.37)	3.62 (2.51 - 5.08)	2 (1.37 - 2.9)	18.42 (15.82 - 20.91)	10.62 (8.56 - 13.14)	7.06 (4.88 - 9.68)	4.31 (3.02 - 5.78)
Cuba	High-middle SDI	24.25 (23.21 - 25.54)	26.59 (26.04 - 26.92)	6.71 (5.93 - 7.46)	18.45 (16.88 - 20.07)	37.44 (34.96 - 39.61)	29.65 (28.41 - 30.78)	14.71 (13.01 - 16.58)	30.26 (27.79 - 32.67)	8.14 (6.49 - 9.97)	10.73 (9.06 - 12.79)	3.29 (2.27 - 4.47)	4.66 (3.51 - 6.03)	11.7 (9.57 - 14.37)	14.59 (12.46 - 17.24)	6.38 (4.69 - 8.47)	8.67 (6.71 - 10.81)
Cyprus	High SDI	44.54 (42.58 - 46.61)	27.93 (26.8 - 29.28)	12.6 (10.52 - 14.89)	16.62 (14.27 - 19.12)	47.29 (45.63 - 48.57)	28.67 (27.5 - 29.78)	16.44 (13.89 - 19.13)	20.47 (17.69 - 23.24)	12.92 (9.5 - 16.5)	11.55 (8.66 - 14.82)	4.39 (2.72 - 6.61)	4.46 (2.85 - 6.38)	17.87 (13.8 - 21.76)	15.47 (11.9 - 19.45)	7.68 (4.96 - 11.34)	6.64 (4.54 - 9.13)
Czech Republic	High SDI	47.12 (45.69 - 48.07)	32.68 (30.74 - 34.11)	16.04 (13.5 - 18.9)	18.25 (15.65 - 21.21)	52.45 (51.01 - 53.38)	31.22 (30.22 - 32.33)	13.46 (11.54 - 15.72)	14.99 (13.05 - 16.99)	14.65 (11.85 - 17.48)	10.54 (8.24 - 12.94)	3.52 (2.29 - 5.19)	2.29 (1.56 - 3.38)	17 (14.26 - 19.78)	11.21 (8.79 - 13.84)	4.79 (3.27 - 6.69)	3.63 (2.52 - 5.14)
Democratic Republic of the Congo	Low SDI	20.5 (19.64 - 21.23)	16.77 (15.22 - 18.36)	5.56 (4.6 - 6.66)	7.12 (6.06 - 8.25)	10.72 (10.09 - 11.2)	12.35 (11.23 - 13.82)	2.61 (2.12 - 3.23)	4.03 (3.38 - 4.72)	7.44 (5.95 - 8.92)	8.18 (6.32 - 10.02)	4.23 (2.9 - 5.95)	2.47 (1.73 - 3.46)	4.47 (3.51 - 5.44)	6.33 (5.07 - 7.69)	2.06 (1.41 - 2.91)	1.81 (1.22 - 2.55)
Denmark	High SDI	40.44 (37.8 - 43.1)	20.71 (19.17 - 22.37)	6.6 (5.52 - 7.89)	8.52 (7.01 - 10.07)	46.74 (45.31 - 47.95)	24.22 (23.32 - 25.05)	15.23 (13.46 - 17.11)	19.38 (17.44 - 21.45)	14.89 (11.19 - 18.9)	11.75 (8.94 - 15.12)	2.74 (1.62 - 4.23)	2.4 (1.47 - 3.44)	18.78 (14.85 - 23.54)	15.6 (12.14 - 19.43)	4.98 (3.24 - 7.28)	5.58 (3.88 - 7.83)
Djibouti	Low-middle SDI	9.69 (7.34 - 12.24)	13.15 (11.17 - 15.22)	2.28 (1.29 - 3.83)	3.33 (2.43 - 4.48)	25.31 (22.03 - 28.28)	27.13 (25.52 - 28.47)	9.8 (6.35 - 13.76)	19.43 (15.81 - 23.29)	4.82 (3.05 - 7.33)	8.75 (6.12 - 11.42)	2.05 (0.96 - 3.71)	3.61 (2.11 - 5.66)	11.63 (8.16 - 15.55)	15.49 (12.52 - 19.37)	7.38 (3.93 - 11.58)	10.54 (6.87 - 15.1)
Dominica	High-middle SDI	18.47 (16.45 - 20.63)	33.2 (31.85 - 34.39)	5.98 (5.07 - 6.95)	26.22 (24.03 - 28.51)	27.63 (25.64 - 29.87)	32.89 (31.94 - 33.48)	12.17 (10.62 - 13.86)	37.26 (34.96 - 39.58)	7.13 (5.68 - 8.84)	12.69 (10.88 - 14.72)	2.96 (2.08 - 3.96)	6.83 (5.28 - 8.66)	10.85 (8.82 - 12.95)	16.07 (14.07 - 18.08)	5.96 (4.35 - 8.07)	11.55 (9.08 - 14.14)
Dominican Republic	High-middle SDI	26.32 (23.88 - 28.67)	25.61 (23.59 - 27.6)	7.89 (6.79 - 9.14)	12.85 (11.46 - 14.37)	35.96 (34.85 - 37.23)	32.51 (31.63 - 33.35)	16.02 (14.67 - 17.43)	25.79 (24.11 - 27.47)	5.48 (4.24 - 6.94)	8.1 (6.59 - 9.82)	1.33 (0.92 - 1.81)	1.89 (1.4 - 2.45)	11.18 (8.96 - 13.4)	14.51 (12.25 - 16.8)	4.33 (3.19 - 5.7)	6.17 (4.73 - 7.86)
Ecuador	High-middle SDI	26.75 (25.87 - 27.22)	29.14 (28.66 - 29.68)	16.3 (13.85 - 19.13)	19.9 (18.5 - 21.45)	43.27 (43.27 - 43.17)	34.93 (34.92 - 34.89)	19.74 (17.58 - 21.96)	28.43 (27.05 - 29.89)	6.26 (5.33 - 6.97)	15.02 (13.57 - 16.79)	3.85 (2.85 - 5)	3.6 (2.92 - 4.37)	13.48 (11.93 - 15.16)	17.39 (15.61 - 19.07)	6.13 (4.64 - 7.83)	6.19 (5.09 - 7.37)
Egypt	Middle SDI	34.16 (31.34 - 36.86)	33.14 (31.45 - 34.78)	11.22 (9.76 - 12.81)	23.81 (21.3 - 26.37)	41.83 (40.59 - 43.11)	32.36 (32.11 - 32.57)	27.03 (24.74 - 29.33)	42.97 (40.28 - 45.57)	10.01 (7.52 - 12.92)	15.09 (11.81 - 18.81)	2.76 (1.92 - 3.79)	4.27 (3.08 - 5.85)	17.08 (13.5 - 20.57)	18.34 (15.33 - 21.95)	9.43 (6.9 - 12.17)	11.03 (8.38 - 13.93)
El Salvador	Middle SDI	17.23 (14.57 - 19.95)	27.1 (24.59 - 29.25)	4.24 (3.21 - 5.45)	11.74 (9.62 - 14.13)	39.99 (38.23 - 41.19)	36.32 (35.71 - 36.73)	17.89 (14.57 - 21.48)	29.99 (26.7 - 33.5)	4.14 (2.99 - 5.55)	9.08 (6.72 - 11.75)	0.76 (0.45 - 1.21)	1.62 (0.99 - 2.46)	9.74 (7.26 - 12.1)	15.87 (12.49 - 19.63)	3.15 (1.95 - 4.65)	5.65 (3.81 - 7.86)
Equatorial Guinea	Middle SDI	11.22 (10.46 - 11.94)	11.91 (10.74 - 13.26)	1.58 (1.24 - 1.98)	3.44 (2.86 - 4.06)	26.22 (25.32 - 26.63)	24.71 (23.05 - 26.03)	10.2 (8.52 - 12.13)	18.75 (16.7 - 21.09)	7.61 (6.17 - 9.12)	10.72 (8.52 - 13.24)	4.26 (2.9 - 5.87)	4.67 (3.3 - 6.28)	17.8 (15.45 - 19.79)	18.31 (15.93 - 20.31)	14.25 (10.86 - 17.95)	13.09 (10.14 - 16.52)
Eritrea	Low SDI	4.46 (3.33 - 5.65)	8.02 (6.72 - 9.32)	6.75 (4.57 - 9.44)	2.33 (1.71 - 3.19)	2.81 (2.17 - 3.4)	9.23 (7.71 - 10.79)	4.11 (2.79 - 6.11)	2.46 (1.79 - 3.26)	2.13 (1.3 - 3.29)	3.24 (2.27 - 4.55)	1.23 (0.59 - 2.15)	0.78 (0.43 - 1.34)	0.91 (0.56 - 1.62)	2.74 (1.92 - 3.8)	0.91 (0.47 - 1.57)	0.58 (0.32 - 0.98)
Estonia	High SDI	37.48 (36.2 - 38.71)	30.06 (29.13 - 30.65)	12.17 (10.62 - 13.69)	19.25 (17.39 - 21.28)	40.03 (39.43 - 40.58)	28.22 (27.76 - 28.73)	20.03 (18.01 - 22.02)	25.7 (23.62 - 27.71)	9.73 (8.13 - 11.37)	8.99 (7.43 - 10.79)	2.8 (2.07 - 3.74)	2.44 (1.75 - 3.24)	16.27 (14.06 - 18.62)	10.08 (8.48 - 11.59)	6.24 (4.75 - 8.03)	5.02 (3.81 - 6.52)
Ethiopia	Low SDI	5.8 (4.24 - 7.78)	6.13 (5.05 - 7.33)	1.19 (0.63 - 1.95)	1.18 (0.81 - 1.6)	11.79 (9.62 - 14.18)	12.58 (11.16 - 14.19)	2.66 (1.66 - 3.94)	4.79 (3.7 - 6.14)	2.15 (1.22 - 3.36)	3.67 (2.59 - 5.15)	0.99 (0.47 - 1.91)	1.01 (0.57 - 1.66)	3.35 (1.86 - 5.35)	5.52 (4.03 - 7.24)	1.62 (0.74 - 3.04)	2.02 (1.21 - 3.24)
Federated States of Micronesia	Middle SDI	26.21 (23.08 - 28.92)	26.68 (24.73 - 28.79)	12.47 (10.56 - 14.78)	24.8 (20.83 - 29.19)	31.14 (28.55 - 33.37)	31.95 (30.89 - 32.32)	17.56 (15.15 - 20.26)	34.97 (30.3 - 39.67)	8.44 (6.22 - 11.18)	12.4 (9.22 - 16.03)	5.63 (3.62 - 8.42)	9.74 (6.18 - 14.26)	8.89 (6.78 - 11.76)	13.56 (10.12 - 17.55)	5.84 (3.89 - 8.37)	10.71 (6.87 - 15.05)
Fiji	High-middle SDI	22.44 (19.7 - 25.38)	24.65 (22.15 - 27.26)	8.94 (7.23 - 10.88)	19.27 (15.71 - 23.04)	28.25 (25.72 - 30.77)	24.83 (23.33 - 26.56)	16.77 (14.33 - 19.4)	29.93 (25.31 - 34.7)	8.72 (6.26 - 11.65)	11.25 (8.13 - 14.81)	2.59 (1.54 - 3.98)	5.16 (2.93 - 7.96)	13.73 (10.42 - 17.63)	14.43 (10.79 - 18.19)	6.17 (3.92 - 8.99)	8.1 (5.17 - 11.79)
Finland	High SDI	45.68 (43.85 - 47.37)	28.54 (27.44 - 29.58)	12.94 (11.09 - 14.91)	16 (13.61 - 18.68)	52.39 (51.12 - 53.13)	30.29 (29.55 - 31.01)	17.7 (15.63 - 20.01)	24.15 (21.59 - 26.77)	19.25 (14.43 - 24.53)	13.23 (9.79 - 17.25)	4.65 (2.91 - 6.81)	2.78 (1.81 - 4.05)	20.61 (16.57 - 24.8)	15.07 (11.35 - 18.72)	8.19 (5.48 - 11.69)	5 (3.38 - 7.05)
France	High SDI	35.89 (33.62 - 38.41)	19.46 (17.88 - 21.41)	7.41 (6.12 - 8.82)	8.17 (6.79 - 9.63)	44.25 (42.74 - 45.52)	27.21 (25.99 - 28.42)	13.68 (12.22 - 15.24)	17.32 (15.58 - 19.12)	11.27 (8.21 - 14.71)	10.27 (7.54 - 12.89)	2.42 (1.44 - 3.86)	2.21 (1.43 - 3.16)	18.13 (13.94 - 22.92)	14.25 (10.92 - 18.18)	4.73 (3.04 - 6.76)	4.97 (3.33 - 6.86)
Gabon	Middle SDI	18.2 (17.22 - 19.25)	18.93 (17.22 - 20.55)	3.84 (3.07 - 4.66)	7.65 (6.57 - 8.82)	32.29 (32.27 - 32.51)	29.81 (28.91 - 30.17)	13.42 (11.28 - 15.5)	28.45 (26.24 - 30.93)	4.2 (3.27 - 5.38)	7.08 (5.52 - 8.82)	1.25 (0.81 - 1.8)	1.41 (0.96 - 1.93)	10.78 (8.89 - 12.85)	15.24 (12.46 - 18.15)	4.86 (3.51 - 6.63)	4.33 (3.13 - 5.76)
Georgia	High-middle SDI	35.54 (33.52 - 37.27)	29.74 (28.31 - 31.08)	8.56 (6.95 - 10.37)	15.5 (13.91 - 17.17)	43.45 (42.59 - 44.29)	31.25 (30.3 - 32.22)	15.76 (13.22 - 18.44)	23.33 (21.25 - 25.41)	12.38 (9.98 - 14.9)	11.08 (9.41 - 12.83)	4.2 (2.83 - 5.97)	4.28 (3.2 - 5.5)	17.68 (14.82 - 20.59)	13.78 (11.79 - 15.86)	8.46 (5.95 - 11.32)	7.85 (6.15 - 9.77)
Germany	High SDI	48.14 (45.38 - 50.74)	26.06 (24.47 - 27.72)	8.46 (7.07 - 10.07)	11.09 (9.33 - 13.03)	48.83 (48.09 - 49.34)	27.58 (26.75 - 28.14)	19.01 (16.89 - 21.25)	22.6 (20.15 - 25.1)	14.34 (10.58 - 18.3)	10.95 (7.99 - 14.39)	2.87 (1.83 - 4.41)	2.75 (1.8 - 4.01)	18.01 (13.88 - 22.39)	14.48 (11.18 - 18.63)	5.97 (4 - 8.4)	5.11 (3.4 - 7.13)
Ghana	Low-middle SDI	5.77 (4.87 - 6.86)	9.82 (8.5 - 11.36)	0.72 (0.54 - 0.95)	2.76 (2.17 - 3.49)	21.84 (19.56 - 23.94)	26.91 (25.62 - 28.01)	7.33 (6.16 - 8.67)	19.15 (16.76 - 21.69)	1.76 (1.32 - 2.38)	3.56 (2.65 - 4.74)	0.58 (0.33 - 0.91)	0.54 (0.33 - 0.82)	5.21 (3.84 - 6.98)	9.08 (6.69 - 11.62)	1.8 (1.16 - 2.71)	2.02 (1.31 - 3.03)
Greece	High-middle SDI	47.51 (45.58 - 49.31)	29.84 (28.82 - 31.14)	11.12 (9.21 - 13.33)	15.86 (13.51 - 18.19)	52.46 (51.34 - 53.14)	30.04 (28.58 - 31.11)	17.1 (14.42 - 19.81)	21.19 (18.64 - 23.85)	17.52 (13.76 - 21.38)	14.09 (10.63 - 17.98)	5.84 (3.67 - 8.61)	4.89 (3.13 - 6.99)	22.82 (18.52 - 26.84)	19.24 (15.24 - 22.85)	10.85 (7.52 - 14.8)	9.18 (6.41 - 12.37)
Greenland	High-middle SDI	40.37 (37.89 - 42.59)	23.28 (22.08 - 24.81)	8.07 (6.56 - 9.72)	9.49 (8 - 11.09)	46.53 (46.06 - 46.35)	29.04 (28.47 - 29.9)	20.39 (17.43 - 23.64)	21.73 (19 - 24.43)	11.17 (9.03 - 13.57)	12.57 (10.35 - 15.18)	3.35 (2.17 - 4.76)	3.75 (2.61 - 5.1)	16.33 (13.87 - 19.08)	16.66 (14.46 - 18.84)	6.9 (4.76 - 9.46)	7 (5.1 - 9.12)
Grenada	High-middle SDI	23.6 (21.06 - 25.9)	31.23 (29.64 - 32.79)	7.3 (6.28 - 8.52)	23.57 (21.38 - 25.7)	33.31 (31.69 - 34.92)	31.92 (31.05 - 32.34)	14.06 (12.6 - 15.72)	34.91 (32.88 - 37.13)	7.07 (5.59 - 8.6)	12.35 (10.76 - 14.47)	3.14 (2.2 - 4.36)	6.45 (4.86 - 8.15)	10.61 (9.03 - 12.69)	15.77 (13.73 - 18.1)	6.24 (4.5 - 8.19)	11.12 (8.71 - 13.78)
Guam	High SDI	37.42 (35.28 - 39.04)	25.92 (24.25 - 27.87)	21.54 (18.56 - 24.53)	27.54 (23.11 - 32.17)	40.79 (39.67 - 41.9)	31.48 (30.82 - 31.36)	30.9 (27.57 - 34.18)	42.57 (37.94 - 47.36)	25.56 (20.38 - 30.44)	12.88 (9.67 - 16.46)	10.32 (6.91 - 14.24)	10.3 (6.65 - 14.51)	28.72 (24.92 - 32.23)	16.69 (13.63 - 19.44)	18.26 (13.26 - 24.05)	19.38 (13.98 - 25.75)

Table S3. The age-standardized prevalence of overweight and obesity among children and adults by sex in 1980 and 2015 among 195 countries.

Country	SDI Level	1980				2015				1980				2015			
		Adult (ages >= 20) Prevalence of Overweight		Adult (ages >= 20) Prevalence of Obesity		Adult (ages >= 20) Prevalence of Overweight		Adult (ages >= 20) Prevalence of Obesity		Child (ages 2 - 19) Prevalence of Overweight		Child (ages 2 - 19) Prevalence of Obesity		Child (ages 2 - 19) Prevalence of Overweight		Child (ages 2 - 19) Prevalence of Obesity	
		Males	*Females*	*Males*	*Females*	*Males*	*Females*	*Males*	*Females*	*Males*	*Females*	*Males*	*Females*	*Males*	*Females*	*Males*	*Females*
Guatemala	Low-middle SDI	19.81 (17.03 - 22.68)	27.74 (24.52 - 30.37)	4.1 (2.92 - 5.5)	10.39 (8.49 - 12.48)	33.59 (31.42 - 35.57)	35.61 (34.01 - 36.99)	11.7 (9.33 - 14.19)	21.5 (18.57 - 24.78)	6.83 (5.1 - 8.9)	9.95 (7.53 - 12.92)	1.33 (0.79 - 2.13)	1.93 (1.25 - 2.82)	11.62 (9 - 14.78)	15.92 (12.6 - 19.37)	3.82 (2.38 - 5.64)	4.24 (2.78 - 6.2)
Guinea	Low SDI	7.43 (6.24 - 8.72)	10.7 (9.39 - 12.15)	0.81 (0.6 - 1.08)	3.04 (2.35 - 3.8)	15.5 (13.64 - 17.6)	19.17 (17.2 - 20.94)	3.7 (2.93 - 4.64)	9.03 (7.47 - 10.88)	3.33 (2.39 - 4.63)	4.87 (3.67 - 6.32)	1.04 (0.6 - 1.64)	1.38 (0.85 - 2.19)	5.4 (3.9 - 6.98)	7.67 (5.97 - 9.59)	2.18 (1.3 - 3.27)	2.4 (1.53 - 3.6)
Guinea-Bissau	Low SDI	3.49 (2.94 - 4.18)	7.41 (6.21 - 8.7)	0.21 (0.16 - 0.29)	1.3 (0.96 - 1.69)	17.13 (14.8 - 19.17)	21.67 (19.82 - 23.34)	4.35 (3.47 - 5.43)	10.54 (8.83 - 12.71)	1.77 (1.28 - 2.37)	3.41 (2.43 - 4.47)	0.61 (0.37 - 0.99)	0.96 (0.61 - 1.47)	7.57 (5.91 - 9.7)	9.91 (7.92 - 12.24)	5.51 (3.67 - 8.05)	6.13 (4.11 - 8.55)
Guyana	Middle SDI	21.07 (18.92 - 23.12)	30.55 (28.71 - 32.3)	6.48 (5.51 - 7.61)	20.7 (18.69 - 22.61)	27.74 (25.48 - 29.77)	31.71 (30.65 - 32.99)	10.44 (9.12 - 11.89)	27.43 (25.38 - 29.4)	4.59 (3.54 - 5.75)	7.66 (6.33 - 9.18)	2.04 (1.45 - 2.8)	3.13 (2.35 - 4.09)	6.48 (5.02 - 8.19)	9.93 (8.31 - 11.76)	3.37 (2.46 - 4.61)	5.48 (4.18 - 7.01)
Haiti	Low-middle SDI	16.94 (14.82 - 19.2)	16.56 (14.97 - 18.21)	3.43 (2.85 - 4.07)	7.95 (6.89 - 9.14)	22.04 (19.53 - 24.56)	22.65 (20.99 - 24.38)	5.98 (5.05 - 6.97)	12.86 (11.53 - 14.26)	5.45 (4.2 - 6.77)	7.08 (5.81 - 8.4)	1.67 (1.15 - 2.33)	2.13 (1.56 - 2.82)	7.38 (5.77 - 9.28)	7.84 (6.31 - 9.57)	1.67 (1.17 - 2.35)	1.69 (1.25 - 2.24)
Honduras	Middle SDI	13.99 (11.79 - 16.06)	25.02 (22.25 - 27.8)	3.21 (2.39 - 4.23)	9.46 (7.67 - 11.52)	36.86 (34.79 - 38.51)	35.27 (34.68 - 35.92)	15.49 (12.27 - 19.09)	28.05 (25.27 - 30.94)	3.51 (2.55 - 4.78)	8.08 (5.98 - 10.66)	0.55 (0.31 - 0.91)	1.43 (0.87 - 2.16)	9.65 (7.38 - 12.46)	14.47 (11.35 - 17.76)	2.8 (1.73 - 4.27)	4.49 (3.05 - 6.29)
Hungary	High SDI	46.34 (45.65 - 46.5)	30.58 (29.93 - 31.14)	22.68 (19.41 - 26.19)	25.52 (22.26 - 29.23)	43.36 (42.19 - 44.45)	29.65 (28.24 - 30.79)	17.5 (14.78 - 20.28)	20.31 (17.74 - 23.15)	18 (15.75 - 20.78)	13.55 (11.15 - 16.32)	7.58 (5.27 - 10.15)	5.3 (3.6 - 7.34)	16.34 (14.09 - 18.6)	14.22 (11.41 - 16.95)	7.17 (5.06 - 9.57)	5.84 (4.19 - 7.84)
Iceland	High SDI	53.51 (52.88 - 53.72)	30.05 (28.83 - 30.68)	18.9 (15.98 - 21.85)	24.78 (21.73 - 27.92)	53.07 (53.36 - 52.87)	32.02 (31.61 - 32.09)	24.01 (20.7 - 27.05)	29.76 (26.4 - 33.17)	24.38 (19.06 - 30.04)	16.03 (12.15 - 20.04)	5.8 (3.74 - 8.7)	5.21 (3.45 - 7.5)	21.87 (16.62 - 26.59)	15.68 (12.05 - 19.23)	6.76 (4.38 - 9.91)	5.63 (3.81 - 7.93)
India	Low-middle SDI	8.35 (7.94 - 8.8)	10.03 (9.5 - 10.54)	1.24 (1.13 - 1.36)	2.14 (1.96 - 2.32)	17.21 (16.59 - 17.84)	17.13 (16.47 - 17.74)	4.58 (4.32 - 4.85)	7.04 (6.68 - 7.43)	2.71 (2.49 - 2.99)	2.71 (2.49 - 2.99)	1.31 (1.16 - 1.48)	1.35 (1.15 - 1.57)	5.22 (4.77 - 5.72)	4.99 (4.57 - 5.43)	3 (2.7 - 3.35)	3.13 (2.78 - 3.5)
Indonesia	Middle SDI	5.78 (4.47 - 7.27)	10.94 (9.34 - 12.71)	0.51 (0.37 - 0.7)	2.23 (1.72 - 2.89)	17.33 (15.36 - 19.41)	21.59 (19.55 - 23.41)	5.21 (4.22 - 6.42)	10.71 (9.09 - 12.46)	1.58 (1.11 - 2.17)	2.46 (1.85 - 3.24)	0.86 (0.52 - 1.41)	0.95 (0.59 - 1.45)	4.48 (3.2 - 5.88)	5.37 (4.14 - 6.73)	3.11 (2.08 - 4.48)	3.11 (2.1 - 4.55)
Iran	High-middle SDI	23.88 (20.98 - 26.74)	31.7 (29.4 - 33.83)	5.8 (4.92 - 6.79)	16.61 (14.6 - 18.77)	35.33 (33.04 - 37.5)	32.33 (30.53 - 33.95)	13.83 (12.51 - 15.14)	24.05 (21.89 - 26.19)	4.13 (3.1 - 5.38)	6.43 (4.74 - 8.36)	1.23 (0.83 - 1.74)	1.66 (1.15 - 2.29)	8.91 (6.75 - 11.18)	10.25 (7.69 - 12.74)	4.25 (3.02 - 5.7)	4.45 (3.2 - 6.05)
Iraq	Middle SDI	36.71 (34.61 - 38.78)	30.69 (29.61 - 31.98)	23.17 (20.8 - 25.51)	33.94 (30.89 - 36.8)	37.2 (35.92 - 38.75)	30.62 (29.66 - 31.22)	25.76 (23.46 - 27.98)	36.95 (34.02 - 39.91)	8.61 (6.58 - 11.15)	11.36 (8.86 - 14.44)	3.71 (2.64 - 4.98)	4.22 (2.98 - 5.69)	9.89 (7.77 - 12.38)	12.67 (9.96 - 15.3)	5.61 (4.11 - 7.49)	5.83 (4.33 - 7.72)
Ireland	High SDI	43.69 (41.38 - 45.98)	27.84 (26.01 - 29.59)	10.42 (8.72 - 12.18)	12.85 (10.8 - 14.94)	41.83 (41.54 - 42.23)	29.97 (29.32 - 30.2)	22.87 (20.29 - 25.64)	25.53 (23.02 - 28.34)	16.79 (12.74 - 21.93)	14.2 (10.63 - 17.73)	4.83 (2.86 - 7.43)	4.49 (2.97 - 6.43)	18.06 (14.11 - 22.22)	15.79 (12.24 - 19.73)	6.08 (3.94 - 9)	5.79 (3.86 - 7.93)
Israel	High SDI	38.33 (36.15 - 40.24)	28.68 (27.14 - 30.07)	11.2 (9.24 - 13.27)	17.84 (15.43 - 20.28)	47.85 (46.36 - 48.95)	29.82 (29.12 - 30.17)	16.57 (14.53 - 18.9)	24.01 (21.25 - 26.7)	10.66 (7.82 - 13.68)	8.06 (6.01 - 10.44)	3 (1.78 - 4.73)	2.36 (1.52 - 3.61)	17.65 (13.72 - 22.5)	12.44 (9.5 - 15.71)	6.75 (4.2 - 9.84)	4.54 (3.02 - 6.6)
Italy	High SDI	45.25 (42.9 - 47.4)	24.37 (23.05 - 25.88)	9.04 (7.8 - 10.43)	11.57 (10.04 - 13.19)	42.87 (41.3 - 44.62)	25.14 (23.96 - 26.51)	10.86 (9.6 - 12.2)	11.08 (9.85 - 12.37)	18.87 (14.22 - 23.42)	12.01 (9.21 - 14.9)	6.44 (4.14 - 9.52)	5.72 (3.69 - 8.28)	21.01 (16.72 - 25.46)	16.75 (13.16 - 20.51)	7.24 (4.7 - 10.18)	5.86 (3.95 - 8.16)
Jamaica	High-middle SDI	21.52 (19.15 - 23.7)	30.76 (28.75 - 32.41)	7.03 (5.98 - 8.16)	20.75 (18.7 - 22.88)	29.8 (27.93 - 31.89)	29.73 (28.65 - 30.7)	14.44 (12.66 - 16.47)	31.89 (29.49 - 34.48)	7.5 (6.07 - 9.19)	12.22 (10.23 - 14.28)	3.58 (2.52 - 4.79)	6.35 (4.9 - 8.06)	9.76 (7.98 - 11.55)	14.64 (12.64 - 16.74)	5.96 (4.3 - 7.88)	8.4 (6.64 - 10.47)
Japan	High SDI	16.55 (16.3 - 16.8)	16.98 (16.71 - 17.26)	1.02 (0.96 - 1.08)	2.41 (2.33 - 2.5)	20.8 (20.55 - 21.07)	14.46 (14.21 - 14.68)	2.51 (2.36 - 2.66)	3.02 (2.9 - 3.14)	7.49 (7.27 - 7.72)	7.42 (7.15 - 7.77)	1.8 (1.66 - 1.94)	1.28 (1.19 - 1.38)	10.97 (10.69 - 11.24)	10.78 (10.43 - 11.21)	3.22 (2.98 - 3.46)	2.46 (2.27 - 2.65)
Jordan	High-middle SDI	36.37 (33.83 - 38.68)	31.38 (29.54 - 32.71)	14.79 (12.99 - 16.57)	27.19 (24.56 - 29.88)	41.6 (40.64 - 42.59)	30.1 (29.8 - 30.32)	31.77 (29.28 - 34.08)	43.39 (40.85 - 45.75)	6.28 (4.55 - 8.33)	9.59 (7.3 - 12.3)	1.6 (1.13 - 2.18)	2.32 (1.63 - 3.21)	13.03 (10.17 - 16.26)	15.44 (12.7 - 18.51)	6.28 (4.58 - 8.21)	6.02 (4.59 - 7.77)
Kazakhstan	High-middle SDI	36.17 (34.1 - 38.18)	29.99 (29.09 - 31.18)	8.51 (6.96 - 10.21)	20.86 (19 - 22.81)	41.53 (39.94 - 43.29)	32.21 (31.47 - 33.08)	11.89 (10.04 - 13.94)	24.38 (22.43 - 26.38)	10.62 (8.5 - 12.98)	11.97 (9.87 - 14.18)	3.01 (2.02 - 4.36)	2.53 (1.9 - 3.35)	14.41 (11.92 - 17.39)	13.22 (11.2 - 15.55)	6.56 (4.62 - 9.06)	4.81 (3.62 - 6.27)
Kenya	Low SDI	11.92 (11.48 - 12.33)	18.13 (17.81 - 18.44)	4.07 (3.73 - 4.45)	6.93 (6.66 - 7.22)	15.88 (15.22 - 16.55)	26.01 (25.8 - 26.22)	5.22 (4.75 - 5.77)	15.3 (14.74 - 15.86)	8.44 (7.86 - 9.11)	9.43 (8.97 - 9.96)	4.29 (3.8 - 4.85)	2.7 (2.46 - 2.96)	5.69 (5.23 - 6.12)	10.03 (9.5 - 10.61)	2.49 (2.2 - 2.82)	2.44 (2.23 - 2.67)
Kiribati	Low-middle SDI	35.89 (34.8 - 36.64)	26.97 (26.72 - 26.82)	32.34 (28.77 - 36.1)	46.41 (41.5 - 51.3)	36.64 (36.03 - 37.08)	28.14 (28.67 - 27.52)	34.44 (30.87 - 37.97)	49.36 (44.24 - 54.3)	15.9 (13.5 - 19.25)	18.92 (16.13 - 22.31)	19.62 (13.92 - 26.17)	28.84 (21.23 - 36.5)	16.72 (14.3 - 19.71)	20.14 (17.27 - 22.21)	20.07 (14.77 - 26.19)	29.45 (21.77 - 37.66)
Kuwait	High SDI	29.61 (27.65 - 31.05)	33.81 (32.95 - 34.71)	22.97 (20.52 - 25.45)	38.86 (36.08 - 41.61)	36.96 (37.09 - 36.97)	32.38 (32.51 - 32.11)	41.36 (39.07 - 43.49)	49.54 (47.76 - 51.39)	12.24 (9.64 - 15.33)	15.58 (12.95 - 18.58)	10.96 (8.06 - 13.99)	10.9 (8.42 - 13.91)	19.09 (16.22 - 21.84)	20.57 (17.52 - 23.63)	22.08 (17.81 - 26.96)	19.24 (15.16 - 23.38)
Kyrgyzstan	Middle SDI	33.57 (31.63 - 35.67)	28.81 (27.71 - 29.96)	8.34 (6.9 - 9.97)	17.15 (15.49 - 18.84)	36.28 (34.57 - 37.88)	33.76 (33.08 - 34.36)	11.98 (10.12 - 14.11)	21.06 (19.51 - 22.69)	13.18 (10.63 - 15.84)	11.3 (9.32 - 13.38)	3.46 (2.34 - 4.92)	3.06 (2.26 - 4.03)	9.99 (7.67 - 12.25)	10.14 (8.31 - 12.28)	2.7 (1.88 - 3.77)	2.31 (1.76 - 3.02)
Laos	Low-middle SDI	5.82 (4.7 - 7.06)	10.92 (9.28 - 12.82)	0.57 (0.41 - 0.79)	1.78 (1.38 - 2.27)	15.63 (13.42 - 18.03)	19.46 (17.32 - 21.81)	2.46 (1.88 - 3.2)	5.25 (4.15 - 6.44)	1.53 (1.09 - 2.1)	3.08 (2.23 - 4.14)	0.56 (0.34 - 0.89)	0.81 (0.5 - 1.23)	3.19 (2.25 - 4.39)	5.05 (3.7 - 6.63)	1.14 (0.74 - 1.69)	1.41 (0.9 - 2.03)
Latvia	High SDI	39.4 (38.01 - 40.67)	30.97 (30.18 - 31.68)	12.04 (10.65 - 13.59)	22.46 (20.48 - 24.44)	40.19 (39.37 - 40.79)	30.58 (30.02 - 31.17)	18.06 (16.33 - 19.97)	26.28 (24.49 - 28.15)	12.66 (10.67 - 14.68)	7.6 (6.43 - 9)	2.64 (1.88 - 3.59)	2.52 (1.84 - 3.32)	15.03 (12.93 - 17.33)	10.28 (8.7 - 11.96)	5.09 (3.8 - 6.75)	3.44 (2.59 - 4.52)
Lebanon	High-middle SDI	33.66 (30.68 - 36.84)	30.51 (27.76 - 33.1)	9.23 (7.97 - 10.53)	12.75 (11.08 - 14.5)	42.55 (40.76 - 43.94)	33.09 (31.72 - 34.42)	22.19 (20.18 - 24.09)	24.56 (22.36 - 27.04)	8.14 (6.24 - 10.44)	10.15 (8.03 - 12.43)	4.12 (2.93 - 5.59)	3.66 (2.57 - 4.96)	13.07 (10.13 - 15.8)	14.1 (11.37 - 17.37)	7.45 (5.54 - 9.71)	6.43 (4.79 - 8.46)
Lesotho	Low-middle SDI	9.74 (8.04 - 11.62)	26.41 (25.24 - 27.44)	2.27 (1.7 - 2.97)	16 (14.17 - 17.93)	16.54 (15.06 - 18.06)	27.96 (27.75 - 28.06)	6.59 (5.58 - 7.68)	28.94 (27.04 - 30.95)	3.45 (2.37 - 5.03)	8.27 (6.92 - 9.68)	1.68 (0.93 - 2.73)	2.6 (1.9 - 3.43)	5.92 (4.05 - 8.37)	11.98 (9.95 - 14.07)	2 (1.16 - 3.15)	4.2 (3.2 - 5.5)
Liberia	Low SDI	27.92 (25.68 - 29.83)	21.06 (19.4 - 23.12)	12.08 (10.09 - 14.28)	12.49 (10.34 - 14.65)	27.87 (26.3 - 29.33)	23.91 (22.64 - 25.28)	14.52 (12.55 - 16.64)	19.69 (17.27 - 22.5)	7.66 (5.8 - 9.35)	8.76 (6.74 - 10.99)	3.13 (2.04 - 4.59)	2.34 (1.5 - 3.45)	7.58 (5.66 - 9.78)	9.07 (6.92 - 11.67)	3.29 (2.16 - 4.84)	2.3 (1.44 - 3.41)
Libya	Middle SDI	38.13 (35.77 - 40.78)	32.15 (30.78 - 33.54)	16.85 (14.78 - 18.8)	27.71 (24.9 - 30.6)	39.5 (37.64 - 41.28)	32.25 (31.15 - 33.1)	21.79 (19.56 - 24.2)	34.85 (31.91 - 38.19)	12.76 (9.84 - 15.64)	17.33 (14.22 - 20.63)	7.49 (5.45 - 9.98)	8.66 (6.42 - 11.18)	13.72 (10.85 - 16.65)	17.79 (14.42 - 21.28)	8.84 (6.59 - 11.41)	9.75 (7.42 - 12.46)
Lithuania	High SDI	41.13 (39.86 - 42.13)	31.9 (30.96 - 32.91)	11.86 (10.2 - 13.59)	19.13 (17.22 - 21)	48.6 (47.75 - 49.37)	31.98 (31.32 - 32.67)	15.59 (13.94 - 17.24)	21.98 (20.37 - 23.72)	9.19 (7.78 - 10.65)	8.56 (7.06 - 10.36)	2.49 (1.82 - 3.31)	2.69 (1.98 - 3.54)	14.34 (12.04 - 16.64)	10.03 (8.4 - 11.91)	4.75 (3.5 - 6.18)	3.65 (2.7 - 4.88)
Luxembourg	High SDI	42.45 (39.84 - 44.6)	21.85 (20.48 - 22.96)	10.92 (9.26 - 12.89)	15.55 (13.34 - 17.93)	41.75 (40.87 - 42.81)	26.38 (25.12 - 27.55)	21.17 (18.36 - 23.98)	19.19 (16.52 - 21.72)	12.84 (9.33 - 17.1)	5.57 (4.12 - 7.45)	3.4 (2.11 - 5.31)	3.04 (1.97 - 4.47)	12.56 (9.51 - 15.28)	8.16 (6.11 - 10.76)	10.86 (7.27 - 15.05)	7.3 (4.83 - 9.97)
Macedonia	High-middle SDI	34.38 (32.37 - 35.95)	28.31 (26.77 - 30.03)	13.6 (11.44 - 16.25)	16.53 (14.15 - 18.97)	38.6 (37.2 - 39.92)	29.63 (28.42 - 30.78)	18.34 (15.48 - 21.47)	20.29 (17.37 - 23.25)	11.47 (9.52 - 13.46)	8.67 (6.68 - 10.76)	3.58 (2.37 - 5.24)	3.1 (2.08 - 4.4)	15.21 (12.76 - 17.39)	11.04 (9.02 - 13.47)	6.15 (4.32 - 8.4)	5.19 (3.66 - 7.05)
Madagascar	Low SDI	14.28 (11.58 - 17.11)	12.01 (10.36 - 13.72)	4.97 (3.01 - 7.73)	2.71 (1.91 - 3.71)	10.54 (7.96 - 13.28)	11.79 (10.17 - 13.68)	2.61 (1.55 - 4.15)	3.1 (2.31 - 4.04)	6.69 (4.16 - 9.95)	5.56 (3.9 - 7.38)	1.63 (0.7 - 3.13)	1.41 (0.79 - 2.33)	4.41 (2.83 - 6.59)	4.11 (2.88 - 5.69)	1.8 (0.82 - 3.42)	1.47 (0.81 - 2.49)
Malawi	Low SDI	16.71 (13.37 - 20.49)	14.09 (11.89 - 16.25)	5.03 (2.96 - 8.02)	2.97 (2.14 - 4.06)	17.15 (14.34 - 20.06)	19.31 (17.31 - 21.28)	4.41 (2.85 - 6.53)	7.43 (5.93 - 9.16)	14.09 (9.95 - 18.79)	11.38 (8.52 - 14.85)	7 (3.51 - 11.71)	4.32 (2.52 - 6.8)	10.78 (7.13 - 15.45)	15.4 (11.8 - 18.96)	4.3 (2.32 - 7.19)	5.11 (3.19 - 7.73)
Malaysia	High-middle SDI	23.79 (20.74 - 26.92)	25.8 (23.25 - 27.95)	6.43 (5.01 - 8.02)	9.88 (8.06 - 11.92)	33.74 (30.93 - 36.35)	30.27 (28.25 - 32.12)	12.97 (10.83 - 15.18)	17.48 (14.99 - 20.19)	6.69 (4.85 - 8.83)	7.89 (6.04 - 9.84)	5.04 (3.38 - 7.02)	5.1 (3.49 - 7.13)	12.5 (9.43 - 15.45)	11.97 (9.49 - 14.52)	9.99 (7.18 - 13.59)	8.91 (6.17 - 11.98)

Table S3. The age-standardized prevalence of overweight and obesity among children and adults by sex in 1980 and 2015 among 195 countries.

Country	SDI Level	1980				2015				1980				2015			
		Adult (ages >= 20) Prevalence of Overweight		Adult (ages >= 20) Prevalence of Obesity		Adult (ages >= 20) Prevalence of Overweight		Adult (ages >= 20) Prevalence of Obesity		Child (ages 2 - 19) Prevalence of Overweight		Child (ages 2 - 19) Prevalence of Obesity		Child (ages 2 - 19) Prevalence of Overweight		Child (ages 2 - 19) Prevalence of Obesity	
		Males	*Females*	*Males*	*Females*	*Males*	*Females*	*Males*	*Females*	*Males*	*Females*	*Males*	*Females*	*Males*	*Females*	*Males*	*Females*
Maldives	Middle SDI	12.52 (10.4 - 14.93)	20.64 (18.02 - 23.29)	1.81 (1.37 - 2.34)	5.95 (4.77 - 7.21)	33.05 (30.98 - 35.17)	36.74 (35.67 - 37.7)	11 (9.45 - 12.9)	20.05 (17.86 - 22.42)	2.73 (1.96 - 3.78)	4.33 (3.16 - 5.74)	1.53 (0.99 - 2.26)	1.23 (0.79 - 1.88)	7.35 (5.61 - 9.81)	8.62 (6.67 - 11.2)	5.79 (3.96 - 7.9)	3.86 (2.64 - 5.51)
Mali	Low SDI	5.02 (4.15 - 5.88)	7 (6.01 - 8.2)	0.45 (0.34 - 0.6)	1.2 (0.93 - 1.54)	25.19 (23.19 - 26.97)	24.26 (22.52 - 25.57)	9.79 (7.99 - 11.79)	17.23 (14.91 - 19.87)	1.25 (0.89 - 1.74)	1.77 (1.29 - 2.39)	0.31 (0.19 - 0.51)	0.33 (0.2 - 0.52)	7.96 (6.05 - 10.01)	8.14 (6.28 - 10.23)	4.25 (2.79 - 6.19)	3.9 (2.52 - 5.74)
Malta	High-middle SDI	49.54 (49 - 50.01)	30.41 (29.87 - 31.23)	20.48 (17.49 - 23.6)	23.3 (20.26 - 26.31)	49.66 (49.18 - 49.59)	31.04 (29.98 - 31.87)	23.93 (20.78 - 27.4)	27.99 (24.9 - 31.23)	21.17 (16.07 - 25.47)	17.11 (13.3 - 20.69)	10.46 (7.18 - 15.13)	7.55 (5.16 - 10.69)	23.25 (19.25 - 28.07)	18.9 (15.48 - 22.76)	12.22 (8.45 - 16.5)	10.66 (7.6 - 14.31)
Marshall Islands	Middle SDI	34.23 (32.11 - 36.13)	32.45 (31.18 - 33.36)	20.76 (17.92 - 23.65)	35.29 (30.72 - 39.71)	37.92 (35.97 - 38.67)	32.79 (32.47 - 32.35)	28.9 (25.82 - 32.54)	43.78 (38.92 - 48.68)	12.07 (9.07 - 15.27)	14.99 (11.48 - 18.43)	5.88 (3.74 - 8.82)	8.45 (5.46 - 12.68)	16.37 (12.23 - 19.63)	18.21 (14.11 - 21.93)	8.91 (5.91 - 13.08)	11.82 (7.73 - 16.8)
Mauritania	Low SDI	9.84 (8.5 - 11.44)	18.54 (16.6 - 20.43)	1.21 (0.93 - 1.59)	8.24 (6.7 - 9.96)	28.95 (26.93 - 30.69)	28.36 (27.77 - 29.5)	11.45 (9.51 - 13.64)	27.52 (24.06 - 30.81)	1.88 (1.3 - 2.6)	5.26 (3.94 - 6.83)	0.69 (0.43 - 1.08)	1.35 (0.87 - 1.99)	5.41 (4.12 - 7.02)	8.56 (6.65 - 10.76)	3.12 (2.05 - 4.6)	4.16 (2.88 - 5.83)
Mauritius	High-middle SDI	20.4 (17.55 - 23.58)	22.29 (20.08 - 24.34)	3.35 (2.6 - 4.2)	8.81 (7.21 - 10.55)	35.68 (33.15 - 38.25)	34.71 (33.08 - 36.01)	11.39 (9.53 - 13.47)	20.31 (17.99 - 22.82)	7.05 (5.13 - 9.27)	6.41 (4.81 - 8.12)	2.4 (1.56 - 3.61)	3.28 (2.2 - 4.79)	15.74 (12.31 - 19.13)	12.77 (9.92 - 15.83)	6.69 (4.51 - 9.32)	8.52 (6.09 - 11.42)
Mexico	Middle SDI	37.32 (36.99 - 37.65)	38.63 (38.36 - 38.88)	13.64 (12.95 - 14.36)	20.52 (19.87 - 21.16)	43.46 (43.29 - 43.54)	37.68 (37.61 - 37.8)	23.4 (22.61 - 24.27)	32.66 (31.89 - 33.41)	15.21 (14.42 - 15.89)	11.83 (11.3 - 12.41)	6.41 (5.78 - 7.07)	3.65 (3.33 - 4.02)	19.12 (18.31 - 19.87)	18.67 (17.88 - 19.47)	9.59 (8.84 - 10.41)	9.21 (8.54 - 9.93)
Moldova	High-middle SDI	33.62 (32.23 - 34.87)	30.13 (29.63 - 30.6)	12.91 (11.47 - 14.55)	23.52 (21.39 - 25.63)	40.37 (40.48 - 40.22)	29.59 (29.15 - 29.97)	19.93 (18.41 - 21.41)	29.76 (28.27 - 31.36)	8.05 (6.72 - 9.43)	7.37 (6.17 - 8.72)	2.84 (2.05 - 3.75)	3.72 (2.8 - 4.96)	11.19 (9.43 - 13.06)	8.42 (6.97 - 9.98)	3.2 (2.4 - 4.08)	3.37 (2.56 - 4.32)
Mongolia	High-middle SDI	31.22 (29.55 - 32.82)	32.39 (30.93 - 33.73)	9.72 (8 - 11.49)	15.94 (14.35 - 17.61)	37.52 (36.25 - 38.48)	36.32 (35.52 - 37.23)	15.33 (13.08 - 17.95)	22.84 (21.02 - 24.72)	10.03 (8.05 - 12.28)	12.33 (10.29 - 14.51)	4.31 (2.86 - 6.1)	3.93 (2.94 - 5.05)	14.16 (11.25 - 17.21)	14.71 (12.36 - 17.21)	4.61 (3.21 - 6.23)	4.19 (3.25 - 5.32)
Montenegro	High-middle SDI	40.3 (38.55 - 41.75)	29.49 (27.97 - 30.64)	13.64 (11.5 - 16.19)	17.65 (15.01 - 20.45)	43.51 (41.69 - 44.69)	30.37 (29.16 - 31.63)	15.28 (12.79 - 18.07)	19.42 (16.59 - 22.39)	13.48 (11.24 - 15.82)	12.86 (10.17 - 15.8)	5.4 (3.75 - 7.47)	4.64 (3.29 - 6.48)	18.23 (15.92 - 20.57)	14.88 (12.32 - 17.94)	10.77 (8.05 - 13.96)	7.27 (5.22 - 9.52)
Morocco	Low-middle SDI	24.74 (22.03 - 27.65)	24.79 (22.69 - 27.23)	6.04 (5.13 - 6.99)	11.37 (9.71 - 13.13)	37.18 (34.28 - 39.9)	32.35 (30.52 - 33.89)	11.82 (10.43 - 13.31)	23.08 (20.56 - 25.71)	6.4 (4.75 - 8.46)	10.42 (8.01 - 13.41)	2.09 (1.45 - 2.86)	2.64 (1.9 - 3.56)	10.82 (8.3 - 13.93)	13.25 (10.39 - 16.31)	4.65 (3.29 - 6.45)	4.9 (3.54 - 6.52)
Mozambique	Low SDI	9.49 (7.05 - 11.8)	12.98 (11.27 - 14.89)	2.65 (1.49 - 4.28)	4.1 (3.01 - 5.53)	16.46 (13.4 - 19.69)	19.13 (17.45 - 20.86)	6.29 (4.06 - 9.19)	11.35 (9.16 - 13.85)	5.03 (3.12 - 7.55)	6.36 (4.54 - 8.82)	2.12 (1.01 - 4.07)	2.2 (1.21 - 3.6)	8.88 (5.63 - 12.1)	10.87 (7.88 - 14.04)	3.29 (1.58 - 6.32)	2.89 (1.67 - 4.68)
Myanmar	Low-middle SDI	5.64 (4.44 - 6.96)	11.89 (10.07 - 13.85)	0.55 (0.41 - 0.73)	2.36 (1.82 - 3.05)	17.89 (15.69 - 20.02)	23.4 (20.96 - 25.22)	6.08 (5.03 - 7.41)	9.99 (8.52 - 11.65)	1.35 (0.95 - 1.89)	2.15 (1.59 - 2.82)	0.54 (0.32 - 0.89)	0.9 (0.56 - 1.37)	5.22 (3.87 - 6.97)	5.57 (4.22 - 7.14)	3.26 (2.15 - 4.77)	2.91 (1.94 - 4.2)
Namibia	Middle SDI	12.83 (10.89 - 14.96)	17.02 (15.7 - 17.98)	6.15 (4.84 - 7.61)	11.44 (9.97 - 13.07)	13.88 (12.52 - 15.18)	21.47 (20.78 - 22.25)	6.33 (5.31 - 7.46)	17.29 (15.95 - 18.6)	5.24 (3.81 - 7.14)	6.22 (5.13 - 7.3)	2.79 (1.75 - 4.2)	1.92 (1.4 - 2.55)	5.28 (3.74 - 7.23)	6.95 (5.92 - 8.07)	2.54 (1.56 - 3.87)	3.23 (2.39 - 4.25)
Nepal	Low-middle SDI	3.85 (3.04 - 4.72)	2.77 (2.17 - 3.48)	0.31 (0.22 - 0.42)	0.28 (0.2 - 0.38)	25.35 (23.49 - 27.33)	20.98 (19.01 - 23.06)	4.81 (3.94 - 5.75)	6.04 (5.02 - 7.24)	1.21 (0.8 - 1.8)	1.35 (0.88 - 1.94)	0.17 (0.1 - 0.28)	0.25 (0.14 - 0.41)	4.61 (3.13 - 6.45)	3.48 (2.46 - 4.76)	1.1 (0.69 - 1.65)	1.66 (1.06 - 2.47)
Netherlands	High SDI	37.93 (35 - 40.95)	27.17 (25.27 - 28.89)	4.9 (4.07 - 5.83)	10.86 (9.24 - 12.77)	46.39 (44.95 - 47.96)	30.62 (29.87 - 31.2)	12.51 (11.24 - 13.75)	22.78 (20.84 - 24.86)	8.28 (5.92 - 11.51)	7.01 (5.21 - 9.18)	1.62 (0.98 - 2.54)	2.42 (1.57 - 3.53)	13.44 (9.86 - 17.37)	11.98 (9.3 - 14.65)	4.21 (2.74 - 6.09)	5.09 (3.4 - 7.14)
New Zealand	High SDI	45.5 (42.52 - 48.37)	28.77 (26.09 - 31.01)	14.27 (12.27 - 16.4)	17.98 (15.65 - 20.67)	44.35 (43.21 - 45.13)	30.7 (29.51 - 32.32)	25.57 (23.03 - 28.19)	28.43 (25.81 - 31.11)	18.57 (14.8 - 22.4)	16.72 (13.7 - 20.27)	6.63 (4.77 - 9.06)	7.19 (5.06 - 9.71)	20.5 (17.19 - 24.11)	19.75 (16.15 - 23.78)	8.68 (6.56 - 10.98)	8.58 (6.35 - 11.3)
Nicaragua	Middle SDI	16.72 (14.59 - 19.24)	30.31 (28.25 - 32.41)	5.52 (4.25 - 7.11)	16.11 (13.31 - 19.03)	34.15 (32.22 - 36.02)	35.81 (34.97 - 36.15)	16.96 (13.8 - 20.31)	28.93 (25.55 - 32.7)	7 (5.33 - 9.06)	11.71 (9.22 - 15.07)	2.2 (1.36 - 3.53)	2.8 (1.92 - 4.08)	10.69 (8.17 - 13.13)	15.59 (12.43 - 18.89)	4.31 (2.71 - 6.39)	5.19 (3.45 - 7.33)
Niger	Low SDI	11.34 (9.74 - 13.15)	10.6 (9.15 - 12.13)	1.27 (0.96 - 1.67)	2.68 (2.11 - 3.34)	22.14 (19.72 - 24.57)	21.85 (19.92 - 24.14)	4.27 (3.37 - 5.32)	6.27 (5.15 - 7.61)	3.27 (2.31 - 4.45)	2.71 (1.93 - 3.51)	0.83 (0.5 - 1.27)	0.64 (0.38 - 1.03)	8.21 (6.15 - 10.69)	5.81 (4.21 - 7.62)	2.7 (1.77 - 3.9)	1.68 (1.08 - 2.47)
Nigeria	Low-middle SDI	6.49 (5.48 - 7.66)	10.22 (8.74 - 11.7)	0.75 (0.55 - 1)	2.54 (1.99 - 3.18)	28.2 (25.94 - 30.11)	22.76 (21.61 - 23.89)	9.81 (7.92 - 12.01)	13.54 (11.77 - 15.56)	2.53 (1.78 - 3.47)	3.74 (2.7 - 5.02)	0.64 (0.37 - 1.04)	0.69 (0.42 - 1.13)	7.56 (5.73 - 9.32)	8.56 (6.69 - 10.91)	3.82 (2.5 - 5.47)	3.48 (2.23 - 4.96)
North Korea	Middle SDI	6.58 (5.53 - 7.79)	8.34 (7.27 - 9.53)	0.52 (0.35 - 0.74)	1.32 (0.94 - 1.81)	7.09 (6.07 - 8.11)	8.29 (7.21 - 9.4)	0.78 (0.55 - 1.13)	1.39 (1.01 - 1.88)	2.07 (1.56 - 2.69)	1.95 (1.51 - 2.57)	0.65 (0.35 - 1.09)	0.53 (0.3 - 0.91)	2.13 (1.59 - 2.73)	2.09 (1.6 - 2.57)	0.63 (0.35 - 1.08)	0.51 (0.29 - 0.86)
Northern Mariana Islands	High SDI	33.83 (31.45 - 35.89)	24.41 (22.41 - 26.35)	17.29 (14.7 - 19.99)	21.33 (17.41 - 25.31)	37.43 (35.16 - 39.16)	32.34 (31.51 - 33.05)	21.98 (19.2 - 25.2)	32.85 (28.08 - 37.89)	16.01 (12.22 - 19.55)	12.97 (10.02 - 16.49)	10.17 (6.74 - 14.58)	9.69 (6.1 - 14.11)	16.72 (12.91 - 20.65)	15.04 (11.51 - 18.72)	10.01 (6.8 - 14.37)	10.39 (6.49 - 15)
Norway	High SDI	39.32 (36.36 - 42.07)	28.39 (26.35 - 30.03)	7.43 (6.05 - 8.98)	9.92 (8.39 - 11.76)	48.1 (46.64 - 49.36)	32.98 (31.51 - 33.93)	15.43 (13.52 - 17.62)	17.45 (15.32 - 19.82)	13.67 (9.81 - 18.12)	11.18 (8.29 - 14.51)	2.97 (1.81 - 4.72)	2.39 (1.47 - 3.69)	20.72 (15.73 - 25.8)	16.24 (12.43 - 20.53)	5.58 (3.68 - 8.21)	4.13 (2.68 - 6.11)
Oman	High-middle SDI	27.04 (24.3 - 29.88)	29 (26.78 - 30.84)	9.21 (7.94 - 10.54)	20.21 (18.2 - 22.64)	39.57 (38.09 - 40.87)	31.81 (30.98 - 32.38)	24.82 (22.62 - 27.1)	37.9 (35.18 - 40.79)	5.89 (4.33 - 7.88)	10.7 (8.46 - 13.23)	2.57 (1.77 - 3.57)	5.53 (4.03 - 7.42)	16.15 (12.66 - 19.51)	16.44 (13.42 - 19.38)	11.63 (8.61 - 14.91)	11.97 (9.07 - 15.34)
Pakistan	Low-middle SDI	16.83 (14.07 - 19.98)	21.3 (17.6 - 24.73)	3.62 (2.71 - 4.72)	6.97 (5.54 - 8.8)	24.26 (21.16 - 27.26)	24.44 (22.62 - 26.37)	7.3 (5.91 - 9.1)	14.49 (12.33 - 16.85)	5.52 (3.87 - 7.8)	5.79 (4.14 - 7.62)	3.03 (1.99 - 4.45)	3.02 (1.95 - 4.51)	3.4 (2.3 - 4.68)	5.34 (3.81 - 7.7)	1.96 (1.24 - 2.92)	2.18 (1.4 - 3.28)
Palestine	Middle SDI	26.99 (24.33 - 29.84)	32.43 (30.5 - 34.6)	10.06 (8.75 - 11.57)	20.96 (18.64 - 23.24)	38.58 (37.48 - 39.63)	37.15 (36.62 - 37.37)	24.5 (22.35 - 26.64)	38.11 (35.64 - 40.54)	6.98 (5.26 - 9.3)	9.34 (7.05 - 11.93)	1.98 (1.37 - 2.74)	2.31 (1.63 - 3.21)	11 (8.5 - 14.09)	12.27 (9.42 - 15.29)	4.62 (3.34 - 6.11)	4.88 (3.52 - 6.53)
Panama	High-middle SDI	6.28 (5.23 - 7.47)	10.94 (9.5 - 12.64)	1.52 (1.08 - 2.02)	4.44 (3.5 - 5.58)	21.45 (18.81 - 23.72)	23.48 (21.42 - 25.39)	7.85 (6.05 - 9.95)	14.98 (12.48 - 17.79)	3.55 (2.53 - 4.78)	5.15 (3.75 - 6.9)	0.9 (0.51 - 1.44)	1.05 (0.66 - 1.59)	10.05 (7.82 - 12.62)	9.96 (7.75 - 12.62)	4.12 (2.61 - 6.21)	3.57 (2.32 - 5.18)
Papua New Guinea	Low-middle SDI	24.77 (21.29 - 28.48)	22.87 (19.55 - 26.13)	5.41 (4.26 - 6.74)	9.12 (7.01 - 11.6)	26.94 (23.87 - 29.91)	27.97 (24.36 - 30.5)	5.93 (4.86 - 7.1)	11.3 (9.21 - 13.98)	9.8 (6.94 - 13.33)	13.57 (9.32 - 17.64)	3.09 (1.93 - 4.81)	2.76 (1.51 - 4.47)	9.94 (6.98 - 13.23)	12.8 (8.82 - 16.75)	3.08 (1.82 - 4.69)	3.17 (1.82 - 5.07)
Paraguay	Middle SDI	27.39 (24.88 - 29.85)	30.42 (28.62 - 31.93)	8.48 (6.33 - 11.08)	14.42 (11.58 - 17.7)	34.83 (33.59 - 36.12)	31.51 (30.68 - 32.08)	21.24 (17.87 - 24.97)	27.04 (23.3 - 30.81)	7.72 (5.65 - 10.13)	9.58 (6.75 - 12.84)	2.27 (1.19 - 3.62)	1.3 (0.73 - 2.16)	13.18 (10.3 - 15.73)	13.75 (10.89 - 17.41)	8.2 (5.18 - 12.56)	6.31 (3.97 - 9.31)
Peru	High-middle SDI	26.07 (24.59 - 27.49)	32.71 (32.07 - 33.69)	8.87 (7.23 - 10.71)	14.4 (13.25 - 15.52)	42.88 (42.77 - 42.8)	39.6 (39.36 - 39.7)	17.27 (15.74 - 18.94)	24.22 (23.17 - 25.31)	12.01 (10.48 - 13.6)	16.36 (14.71 - 18.1)	2.76 (1.91 - 3.85)	3.01 (2.43 - 3.68)	14.89 (13.18 - 17.2)	18.24 (16.44 - 20.05)	4.33 (3.19 - 5.57)	4.12 (3.39 - 4.91)
Philippines	Middle SDI	12.31 (10.22 - 14.58)	13.51 (11.74 - 15.38)	1.63 (1.22 - 2.13)	2.93 (2.3 - 3.7)	20.57 (18.12 - 22.82)	20.95 (18.97 - 22.88)	5.13 (4.17 - 6.29)	7.46 (6.19 - 8.71)	1.5 (1.06 - 2.06)	2.25 (1.66 - 2.94)	0.6 (0.39 - 0.9)	0.65 (0.41 - 0.99)	4.39 (3.18 - 5.79)	3.88 (2.84 - 5.1)	2.89 (1.97 - 4.12)	2.22 (1.5 - 3.11)
Poland	High SDI	48.55 (47.16 - 49.57)	27.29 (26.34 - 28.17)	16.37 (13.66 - 19.24)	19.38 (16.61 - 22.22)	47.71 (46.7 - 49)	29.8 (28.98 - 31.07)	16.54 (14.34 - 18.78)	17.69 (15.65 - 19.78)	14.77 (11.98 - 17.44)	12.3 (9.6 - 15.33)	3.82 (2.63 - 5.48)	3.31 (2.23 - 4.64)	16.05 (13.29 - 18.6)	12.59 (9.76 - 15.08)	5.67 (3.88 - 7.75)	4.05 (2.86 - 5.75)
Portugal	High-middle SDI	38.66 (35.86 - 41.44)	28.19 (26.27 - 29.87)	7.08 (5.73 - 8.53)	12.13 (10.21 - 14.26)	47.35 (46.24 - 48.52)	30.99 (30.41 - 31.47)	17.24 (15.13 - 19.46)	22.5 (19.92 - 25.13)	13.42 (9.69 - 17.31)	10.71 (8.18 - 13.48)	3.47 (2.11 - 5.35)	3.08 (1.97 - 4.74)	20.07 (15.81 - 24.34)	16.83 (13.49 - 20.72)	8.75 (5.79 - 12.29)	8.02 (5.51 - 11.04)
Puerto Rico	High SDI	33.52 (31.48 - 35.59)	30.84 (29.3 - 32.19)	14.43 (12.67 - 16.25)	23.7 (21.6 - 26.01)	43.88 (43.34 - 44.39)	29.52 (29.15 - 29.87)	32.64 (30.25 - 35.08)	43.85 (41.25 - 46.39)	13.51 (11.1 - 16.27)	15.19 (13.14 - 17.34)	5.73 (4.12 - 7.78)	5.72 (4.38 - 7.28)	21.43 (19.13 - 23.78)	20.22 (18.06 - 22.38)	15.58 (12.2 - 19.23)	13.29 (10.85 - 16.09)
Qatar	High-middle SDI	39.11 (39.24 - 39.16)	18.63 (18.08 - 19.44)	43.46 (40.58 - 46.16)	52.96 (49.38 - 56.16)	36.78 (36.66 - 36.82)	23.64 (23.73 - 23.63)	42.52 (40.13 - 44.82)	52.42 (50.25 - 54.5)	14.68 (12.23 - 17.13)	8.97 (7.2 - 10.84)	20.49 (16.09 - 25.33)	10.61 (7.92 - 13.75)	16.11 (13.3 - 18.72)	13.43 (11 - 16.01)	20.84 (16.59 - 25.16)	13.51 (10.34 - 17.17)

Table S3. The age-standardized prevalence of overweight and obesity among children and adults by sex in 1980 and 2015 among 195 countries.

Country	SDI Level	1980				2015				1980				2015			
		Adult (ages >= 20) Prevalence of Overweight		Adult (ages >= 20) Prevalence of Obesity		Adult (ages >= 20) Prevalence of Overweight		Adult (ages >= 20) Prevalence of Obesity		Child (ages 2 - 19) Prevalence of Overweight		Child (ages 2 - 19) Prevalence of Obesity		Child (ages 2 - 19) Prevalence of Overweight		Child (ages 2 - 19) Prevalence of Obesity	
		Males	*Females*	*Males*	*Females*	*Males*	*Females*	*Males*	*Females*	*Males*	*Females*	*Males*	*Females*	*Males*	*Females*	*Males*	*Females*
Romania	High-middle SDI	39.73 (37.84 - 41.56)	27.9 (26.46 - 29.54)	13.48 (11.15 - 16.04)	14.59 (12.14 - 17.2)	38.43 (37.05 - 39.11)	28.56 (26.98 - 29.79)	19.54 (16.81 - 22.75)	17.48 (14.88 - 20.4)	8.98 (7.45 - 10.46)	7.92 (6.17 - 10.05)	4.18 (2.87 - 5.99)	3.1 (2.07 - 4.38)	12.95 (10.93 - 15.24)	10.15 (8.15 - 12.58)	7.85 (5.59 - 10.46)	5.17 (3.58 - 7.18)
Russia	High SDI	34.33 (33.19 - 35.58)	31.86 (31.22 - 32.44)	10.23 (8.97 - 11.59)	24.41 (22.31 - 26.59)	41.06 (40.57 - 41.35)	30.86 (30.71 - 30.92)	16.42 (14.58 - 18.46)	30.21 (28.09 - 32.46)	10.47 (9.14 - 11.75)	10.27 (8.79 - 11.86)	6.13 (4.69 - 7.92)	5.53 (4.13 - 7.11)	14.74 (12.99 - 16.59)	10.8 (9.07 - 12.57)	7.37 (5.66 - 9.32)	5.62 (4.31 - 7.23)
Rwanda	Low SDI	6.1 (4.48 - 8.09)	15.33 (12.92 - 17.59)	1.01 (0.56 - 1.69)	3.28 (2.4 - 4.29)	10.88 (8.83 - 13.22)	22.75 (20.74 - 24.83)	1.42 (0.94 - 2.06)	6.51 (5.35 - 7.82)	9.16 (5.68 - 13.23)	12.22 (8.9 - 16.49)	3.03 (1.37 - 5.83)	2.29 (1.29 - 3.72)	9.25 (6.03 - 12.89)	13.99 (10 - 18.01)	3.07 (1.35 - 5.69)	3.41 (2.02 - 5.29)
Saint Lucia	High-middle SDI	22.07 (19.95 - 24.4)	33.12 (31.76 - 34.39)	6.53 (5.57 - 7.68)	26.29 (24.16 - 28.69)	31.95 (30.48 - 33.29)	32.83 (32.2 - 33.26)	12.92 (11.91 - 14.02)	37.29 (35.46 - 39.13)	5.07 (4 - 6.27)	7.99 (6.65 - 9.51)	2.03 (1.44 - 2.75)	2.62 (1.96 - 3.46)	4.47 (3.56 - 5.71)	7.95 (6.66 - 9.39)	2.67 (1.95 - 3.62)	3.7 (2.8 - 4.73)
Saint Vincent and the Grenadines	High-middle SDI	23.86 (21.69 - 25.93)	30.21 (28.46 - 31.68)	9.33 (8.06 - 10.68)	19.66 (17.63 - 21.89)	39.34 (37.81 - 40.13)	30.97 (30.17 - 31.25)	26.52 (24.28 - 29.13)	40.34 (37.74 - 43.14)	9 (7.22 - 10.74)	10.97 (8.99 - 12.83)	3.63 (2.59 - 5.02)	3.98 (3.01 - 5.11)	15.17 (12.89 - 17.7)	16.56 (14.16 - 18.49)	10.51 (8.03 - 13.4)	10.39 (8.08 - 13.08)
Samoa	Middle SDI	36.53 (35.26 - 37.46)	26.37 (26.67 - 25.78)	30.63 (27.35 - 34.18)	54.61 (49.73 - 59.53)	36.93 (36.05 - 37.11)	26.09 (26.57 - 25.28)	36.6 (32.97 - 40.32)	58.21 (53.16 - 62.95)	14 (11.2 - 16.76)	16.33 (13.63 - 18.88)	16.48 (11.49 - 21.55)	20.67 (14.37 - 27.46)	15.41 (12.84 - 18.22)	17.12 (14.45 - 20.36)	19.12 (13.74 - 25.36)	22.63 (15.95 - 29.57)
Sao Tome and Principe	Low-middle SDI	16.52 (14.48 - 18.75)	19.91 (18.04 - 21.64)	3.55 (2.77 - 4.45)	9.19 (7.47 - 11.18)	25.52 (23.6 - 27.33)	26 (24.22 - 27.64)	8.29 (6.74 - 10.13)	17.3 (14.75 - 20)	4.71 (3.46 - 6.24)	8.14 (6.14 - 10.31)	1.89 (1.19 - 2.86)	2.6 (1.69 - 3.86)	7.23 (5.3 - 9.62)	9.87 (7.33 - 12.56)	2.38 (1.55 - 3.44)	2.82 (1.89 - 3.99)
Saudi Arabia	High-middle SDI	32.35 (31.33 - 33.24)	31.95 (31.13 - 32.49)	11.41 (10.85 - 11.97)	26.51 (25.69 - 27.53)	38.78 (38.34 - 39.18)	30.32 (30.17 - 30.45)	29.75 (28.89 - 30.67)	43.07 (42.11 - 44.07)	6.12 (5.52 - 6.76)	11.06 (10.19 - 11.98)	2.58 (2.24 - 2.97)	4.48 (3.98 - 5.06)	15.34 (14.21 - 16.52)	19.31 (17.96 - 20.47)	11.1 (10.05 - 12.17)	13.81 (12.53 - 15.19)
Senegal	Low SDI	14.68 (12.68 - 16.91)	15.26 (13.24 - 17.17)	2.92 (2.28 - 3.68)	5.63 (4.48 - 6.89)	19.43 (17.47 - 21.42)	23.24 (21.54 - 24.83)	4.69 (3.86 - 5.59)	11.45 (9.7 - 13.41)	3.24 (2.3 - 4.4)	4.96 (3.69 - 6.4)	0.99 (0.61 - 1.5)	1.26 (0.8 - 1.84)	2.8 (2.07 - 3.83)	6.31 (4.7 - 8.06)	1.53 (0.97 - 2.23)	2.17 (1.45 - 3.21)
Serbia	High-middle SDI	39.84 (38.18 - 41.25)	29.23 (27.58 - 30.99)	14.67 (12.17 - 17.35)	18.14 (15.65 - 21)	42.02 (41.15 - 42.74)	29.87 (29.14 - 30.54)	18.34 (17.13 - 19.55)	22.49 (21.12 - 23.92)	10.83 (9.16 - 12.7)	13.08 (10.54 - 15.65)	5.4 (3.62 - 7.43)	5.63 (3.87 - 7.69)	12.39 (10.19 - 14.94)	13.57 (10.78 - 16.21)	5.67 (4.24 - 7.48)	5.31 (3.88 - 7.17)
Seychelles	High-middle SDI	19.74 (16.73 - 22.73)	27.12 (25.38 - 28.89)	3.51 (2.73 - 4.39)	16.66 (14.19 - 19.25)	32.96 (31.39 - 34.65)	32.33 (31.53 - 33.29)	16.5 (14.7 - 18.41)	33.52 (31.38 - 35.86)	5.15 (3.66 - 6.77)	8.31 (6.36 - 10.76)	2.44 (1.53 - 3.65)	3.5 (2.36 - 5.04)	8.2 (6.03 - 10.81)	11.6 (8.96 - 14.7)	4.8 (3.26 - 6.81)	5.86 (4.05 - 8.06)
Sierra Leone	Low SDI	8.78 (7.54 - 10.07)	16.57 (14.73 - 18.66)	2.49 (1.93 - 3.19)	6.92 (5.6 - 8.49)	13.74 (12.2 - 15.03)	19.58 (18.18 - 20.87)	6.71 (5.68 - 7.98)	10.7 (9.37 - 12.14)	4.63 (3.5 - 6.1)	7.43 (5.71 - 9.56)	2.41 (1.51 - 3.57)	2.71 (1.75 - 4.06)	7.22 (5.52 - 8.98)	10.25 (8.07 - 12.8)	4.94 (3.33 - 7.02)	4.93 (3.29 - 6.93)
Singapore	High SDI	24.39 (23.17 - 25.29)	21.18 (19.6 - 22.65)	4.3 (3.37 - 5.51)	6.2 (5.11 - 7.39)	29.46 (28.41 - 30.31)	22.74 (21.4 - 24.2)	8.38 (6.78 - 10.12)	8.85 (7.58 - 10.29)	9.78 (8.46 - 11.02)	8.35 (6.87 - 9.93)	3.33 (2.28 - 4.68)	2.61 (1.78 - 3.62)	13.59 (12.16 - 15.06)	10.89 (9.03 - 12.88)	6.63 (4.68 - 8.9)	4.06 (2.72 - 5.65)
Slovakia	High SDI	45.74 (44.03 - 47.08)	28.95 (27.8 - 30.54)	14.91 (12.63 - 17.6)	19.12 (16.53 - 21.76)	50.4 (48.89 - 51.91)	28.25 (27.54 - 29.41)	13.24 (11.29 - 15.25)	17.64 (15.13 - 19.79)	10.45 (8.46 - 12.12)	9.51 (7.52 - 11.67)	4.22 (2.89 - 6.04)	3.16 (2.13 - 4.5)	12.23 (10.15 - 14.62)	8.28 (6.47 - 10.44)	5.47 (3.82 - 7.59)	2.81 (1.88 - 3.93)
Slovenia	High SDI	41.72 (40.05 - 43.45)	28.76 (27.04 - 30.07)	13.36 (11.02 - 15.83)	14.48 (12.19 - 17.02)	45.67 (44.21 - 46.71)	27.07 (26.18 - 27.87)	17.16 (14.9 - 19.53)	18.45 (16.01 - 21.11)	15.13 (12.48 - 17.72)	13.54 (11.04 - 16.53)	4.74 (3.27 - 6.66)	3.83 (2.67 - 5.24)	21.1 (17.81 - 23.97)	15.48 (12.77 - 18.38)	7 (5.02 - 9.59)	5.7 (4.13 - 7.8)
Solomon Islands	Low-middle SDI	26.49 (23.66 - 29.22)	28.25 (25.6 - 30.47)	12.2 (9.95 - 14.49)	22.76 (18.77 - 26.89)	34.32 (32.09 - 35.92)	31.1 (30.05 - 31.99)	22.12 (19.33 - 25.19)	34.48 (29.61 - 39.37)	8.04 (5.73 - 10.49)	15.14 (11.59 - 18.94)	4.35 (2.73 - 6.59)	9.16 (5.73 - 13.57)	12.83 (9.62 - 16.38)	19.25 (15.64 - 23.31)	8.47 (5.68 - 11.91)	14.38 (9.32 - 20.25)
Somalia	Low SDI	9.37 (7.15 - 11.69)	17.95 (15.72 - 20.17)	2.49 (1.43 - 4.15)	8.43 (6.47 - 10.68)	10.69 (8.32 - 13.17)	19.39 (17.61 - 21.13)	2.48 (1.5 - 3.95)	10.46 (8.33 - 12.79)	4.48 (2.83 - 6.44)	6.26 (4.49 - 8.1)	2.6 (1.27 - 4.78)	2.83 (1.73 - 4.37)	4.26 (2.74 - 6.18)	7 (5.01 - 9.3)	2.45 (1.21 - 4.3)	3.19 (1.96 - 4.83)
South Africa	Middle SDI	25.39 (24.56 - 26.45)	28.19 (28.09 - 28.27)	12.98 (12 - 13.92)	31.21 (30.24 - 32.28)	26.14 (25.14 - 27.04)	28.24 (28.06 - 28.41)	14.69 (13.81 - 15.54)	44.57 (43.74 - 45.35)	11.27 (10.13 - 12.7)	13.56 (12.89 - 14.22)	5.95 (4.97 - 7.02)	6.46 (5.79 - 7.18)	12.46 (11.21 - 13.74)	16.75 (16.01 - 17.61)	7.66 (6.54 - 8.96)	9.97 (9.16 - 10.83)
South Korea	High SDI	17.14 (15.92 - 18.39)	19.88 (18.12 - 21.59)	1.27 (0.92 - 1.69)	2.47 (2.01 - 3.05)	27.69 (26.32 - 29.07)	21.53 (19.96 - 23.09)	2.81 (2.23 - 3.51)	3.11 (2.65 - 3.61)	11.02 (9.5 - 12.45)	9.46 (7.58 - 11.22)	2.8 (1.8 - 4.08)	2 (1.32 - 2.91)	18.06 (16.17 - 19.65)	12 (10.03 - 14.01)	6.43 (4.36 - 9.01)	3.68 (2.47 - 5.25)
South Sudan	Low SDI	11.32 (8.85 - 14.34)	13.73 (11.89 - 15.8)	3.53 (2.09 - 5.42)	4.04 (2.98 - 5.45)	13.72 (10.53 - 16.76)	20.86 (18.79 - 23.03)	3.79 (2.3 - 5.83)	9.97 (7.72 - 12.55)	6.54 (4.16 - 9.63)	8.71 (6.66 - 11.43)	4.84 (2.58 - 8.04)	5.62 (3.53 - 8.32)	6.48 (4.25 - 9.14)	9.42 (7.21 - 11.97)	5.04 (2.51 - 8.79)	6.4 (4.04 - 9.44)
Spain	High-middle SDI	45.04 (42.64 - 47.06)	29.17 (27.51 - 30.76)	9.55 (8.1 - 11.35)	14.13 (12.14 - 16.29)	46.53 (44.84 - 47.67)	26.78 (25.36 - 28.2)	15.96 (14.15 - 18.15)	19.59 (17.66 - 21.7)	17.85 (14.49 - 21.24)	15.01 (11.54 - 18.1)	8.35 (5.42 - 12.19)	6.85 (4.6 - 9.77)	19.16 (15.26 - 22.78)	16.34 (13.11 - 20.04)	8.63 (5.8 - 12.22)	7.43 (4.98 - 10.01)
Sri Lanka	High-middle SDI	12.34 (10.14 - 14.89)	19.28 (16.92 - 21.92)	1.52 (1.11 - 2.05)	5.02 (4.05 - 6.13)	19.44 (17.55 - 21.47)	28.9 (27.25 - 30.38)	3.52 (2.87 - 4.36)	9.02 (7.77 - 10.42)	3.18 (2.29 - 4.5)	4.07 (3.01 - 5.19)	1.82 (1.15 - 2.7)	1.77 (1.14 - 2.79)	4.94 (3.61 - 6.42)	6.65 (4.86 - 8.34)	2.87 (1.87 - 4.29)	2.99 (2 - 4.32)
Sudan	Low-middle SDI	19.68 (17.36 - 22.22)	27.97 (25.89 - 29.94)	5.48 (4.61 - 6.5)	18.23 (15.99 - 20.54)	28.62 (26.1 - 31.06)	30.31 (28.74 - 31.63)	11.47 (10.01 - 13.12)	28.33 (25.6 - 31.22)	2.85 (2.14 - 3.7)	5.36 (4.1 - 6.72)	1.51 (1.04 - 2.16)	2.23 (1.54 - 3.15)	3.33 (2.44 - 4.37)	5.34 (4.02 - 6.85)	2.13 (1.49 - 3)	2.69 (1.97 - 3.58)
Suriname	High-middle SDI	15.92 (14.07 - 17.82)	28.35 (26.47 - 30.26)	4.7 (3.94 - 5.56)	14.59 (13.03 - 16.29)	35.55 (33.46 - 37.43)	31.81 (31.14 - 32.37)	20.39 (18.24 - 22.58)	36.57 (34.01 - 39.16)	4.44 (3.5 - 5.6)	7.22 (5.94 - 8.67)	1.45 (1 - 2.07)	2.33 (1.74 - 3.05)	9.68 (7.79 - 11.78)	12.16 (10.44 - 14.3)	5.2 (3.78 - 6.95)	5.88 (4.43 - 7.54)
Swaziland	Middle SDI	26.02 (23 - 28.84)	28.79 (27.83 - 29.54)	11.84 (9.69 - 14.2)	27.85 (25.49 - 30.42)	23.68 (22.53 - 24.57)	29.19 (28.86 - 29.38)	12.66 (11.3 - 14.1)	36.82 (35.06 - 38.57)	11.96 (8.7 - 15.55)	16.41 (14.13 - 19.05)	4.4 (2.82 - 6.37)	6 (4.55 - 7.75)	8.2 (5.95 - 11.26)	14.87 (12.71 - 17.23)	2.39 (1.54 - 3.56)	5.04 (3.77 - 6.47)
Sweden	High SDI	41.42 (39.02 - 43.48)	25.2 (23.93 - 26.51)	7.59 (6.51 - 8.87)	11.94 (10.38 - 13.75)	47.7 (46.43 - 48.85)	28.65 (27.72 - 29.61)	15.94 (13.97 - 18.01)	20.6 (18.31 - 22.91)	13.9 (10.55 - 17.27)	13.54 (10.75 - 16.91)	2.75 (1.83 - 3.97)	3.02 (2.13 - 4.17)	20.61 (16.44 - 24.86)	15.76 (12.67 - 19.02)	4.37 (3.09 - 6.11)	4.24 (3.08 - 5.65)
Switzerland	High SDI	41.02 (38.08 - 43.57)	21.44 (19.59 - 23.23)	7.74 (6.41 - 9.22)	9.37 (7.76 - 11)	45.28 (43.35 - 46.76)	25.96 (24.66 - 27.05)	12.37 (10.85 - 14.14)	14.52 (12.96 - 16.31)	14.38 (10.54 - 18.47)	10.18 (7.58 - 13.95)	2.55 (1.47 - 4.01)	1.7 (1.02 - 2.65)	14.89 (10.99 - 18.93)	11.01 (8.12 - 14.17)	4.38 (2.9 - 6.31)	3.62 (2.37 - 5.16)
Syria	Middle SDI	38.52 (36.13 - 41.12)	34.34 (32.38 - 35.97)	14.16 (12.57 - 15.88)	23.62 (21.25 - 26.03)	43.08 (40.91 - 45.04)	35.09 (33.73 - 36.18)	19.89 (17.94 - 21.98)	30.22 (27.58 - 32.96)	10 (7.6 - 12.52)	12.28 (9.62 - 15.16)	3.56 (2.44 - 4.9)	5.29 (3.83 - 7.04)	13.34 (10.6 - 16.36)	13.03 (10.52 - 15.98)	7.38 (5.4 - 9.79)	6.26 (4.57 - 8.32)
Taiwan	High SDI	23.48 (20.56 - 26.19)	22.83 (20.92 - 24.92)	3.13 (2.22 - 4.22)	5.5 (4.13 - 6.9)	32.02 (29.97 - 33.99)	24.05 (22.8 - 25.29)	7.6 (6.04 - 9.33)	8.25 (6.81 - 9.94)	13.78 (11.39 - 16.68)	10.74 (8.83 - 12.93)	6.8 (4.23 - 10.04)	3.82 (2.24 - 5.75)	16.16 (13.61 - 18.57)	11.85 (9.55 - 14.02)	8.35 (5.36 - 11.89)	4.34 (2.69 - 6.61)
Tajikistan	Middle SDI	20.25 (18.19 - 22.16)	23.43 (21.64 - 25.25)	3.39 (2.73 - 4.24)	9.95 (8.79 - 11.13)	19.82 (17.93 - 21.81)	26.11 (24.72 - 27.48)	3.93 (3.1 - 4.82)	12.94 (11.76 - 14.27)	7.77 (6.27 - 9.41)	8.94 (7.21 - 10.49)	3.97 (2.6 - 5.59)	3.08 (2.27 - 3.98)	5.84 (4.65 - 7.22)	7.33 (6.09 - 8.69)	3.06 (2.12 - 4.15)	2.47 (1.83 - 3.27)
Tanzania	Low-middle SDI	16.75 (13.53 - 20.36)	12.93 (11.48 - 14.61)	6.47 (4 - 9.81)	5.22 (3.95 - 6.79)	13.52 (11.72 - 15.57)	23.46 (21.99 - 24.7)	3.73 (2.72 - 4.92)	11.47 (9.94 - 13.28)	6.61 (4.23 - 9.91)	7.79 (5.56 - 10.76)	2.39 (1.11 - 4.64)	1.62 (0.89 - 2.64)	7.09 (4.37 - 10.23)	9.92 (7.29 - 13.15)	2.81 (1.49 - 4.87)	2.76 (1.66 - 4.31)
Thailand	High-middle SDI	16.36 (13.79 - 19.21)	16.26 (14.27 - 18.31)	3.06 (2.3 - 3.92)	4.95 (3.91 - 6.09)	25.48 (22.81 - 28.19)	26.12 (24.07 - 28.12)	7.84 (6.25 - 9.51)	12.19 (10.33 - 14.06)	4.71 (3.5 - 6.22)	5.28 (4.03 - 6.78)	2.72 (1.8 - 4.02)	3.32 (2.24 - 4.73)	9.26 (6.72 - 12.24)	8.46 (6.62 - 10.54)	6.39 (4.38 - 8.76)	5.67 (3.97 - 7.79)
The Bahamas	High SDI	25.13 (22.94 - 27.39)	30.72 (28.61 - 32.65)	8.26 (7.2 - 9.58)	18.25 (16.42 - 20.09)	39.99 (38.02 - 41.57)	32.68 (32.02 - 33.18)	19.96 (17.86 - 22.09)	34.28 (31.8 - 36.87)	10.7 (8.63 - 12.92)	12.38 (10.62 - 14.49)	3.58 (2.55 - 5)	3.83 (2.87 - 4.89)	15.06 (12.33 - 17.81)	17.03 (14.63 - 19.42)	7.99 (5.96 - 10.37)	8.15 (6.42 - 10.26)
The Gambia	Low SDI	11.31 (9.74 - 13.15)	15.56 (13.56 - 17.69)	1.78 (1.4 - 2.27)	4.59 (3.66 - 5.56)	27.35 (25.2 - 29.55)	23.51 (22.29 - 24.72)	9.16 (7.62 - 10.84)	13.27 (11.87 - 14.84)	3.18 (2.3 - 4.44)	6.19 (4.49 - 8.13)	0.55 (0.32 - 0.86)	0.86 (0.51 - 1.3)	7.25 (5.48 - 9.58)	8.78 (6.74 - 11.19)	2.09 (1.29 - 3.1)	2.45 (1.57 - 3.55)
Timor-Leste	Low-middle SDI	2.61 (2.09 - 3.25)	5.77 (4.81 - 6.89)	0.24 (0.17 - 0.36)	1.01 (0.77 - 1.29)	8.17 (7.16 - 9.3)	12.18 (10.69 - 13.71)	0.82 (0.67 - 1.01)	2.1 (1.76 - 2.51)	2.5 (1.78 - 3.45)	2.33 (1.68 - 3.14)	1.71 (1.07 - 2.51)	1.27 (0.77 - 1.98)	2.82 (1.99 - 3.92)	4.11 (3.08 - 5.13)	2.33 (1.49 - 3.53)	2.4 (1.55 - 3.6)

Table S3. The age-standardized prevalence of overweight and obesity among children and adults by sex in 1980 and 2015 among 195 countries.

Country	SDI Level	1980				2015				1980				2015			
		Adult (ages >= 20) Prevalence of Overweight		Adult (ages >= 20) Prevalence of Obesity		Adult (ages >= 20) Prevalence of Overweight		Adult (ages >= 20) Prevalence of Obesity		Child (ages 2 - 19) Prevalence of Overweight		Child (ages 2 - 19) Prevalence of Obesity		Child (ages 2 - 19) Prevalence of Overweight		Child (ages 2 - 19) Prevalence of Obesity	
		Males	***Females***	***Males***	***Females***	***Males***	***Females***	***Males***	***Females***	***Males***	***Females***	***Males***	***Females***	***Males***	***Females***	***Males***	***Females***
Togo	Low SDI	8.07 (6.81 - 9.41)	11.13 (9.69 - 12.6)	1.01 (0.77 - 1.32)	3.04 (2.38 - 3.82)	16.17 (14.48 - 17.9)	23.01 (21.45 - 24.54)	3.4 (2.83 - 4.02)	13.18 (11.34 - 15.12)	2.5 (1.78 - 3.31)	3.39 (2.54 - 4.43)	0.52 (0.31 - 0.83)	0.97 (0.61 - 1.5)	5.7 (4.13 - 7.52)	7.2 (5.48 - 9.19)	1.39 (0.87 - 2.08)	2.4 (1.61 - 3.5)
Tonga	Middle SDI	32.97 (31.49 - 33.61)	29.15 (29.81 - 28)	35.25 (31.73 - 39.08)	53.27 (48.31 - 58)	32.72 (33.38 - 32.32)	26.57 (27.36 - 25.07)	48.22 (44.4 - 51.82)	61.32 (56.73 - 65.56)	14.34 (11.12 - 18.06)	18.02 (14.27 - 21.19)	10.47 (6.92 - 14.82)	13.96 (9.58 - 19.48)	23.9 (20.05 - 27.75)	25.69 (21.43 - 29.83)	16.22 (11.74 - 21.57)	16.35 (11.02 - 22.49)
Trinidad and Tobago	High SDI	28.09 (26.07 - 30.29)	29.87 (28.55 - 31.25)	12.76 (11.1 - 14.47)	23.11 (21.05 - 25.21)	36.03 (34.79 - 37.2)	26.94 (26.3 - 27.56)	20.3 (18.5 - 22.07)	34.25 (31.82 - 36.66)	3.37 (2.63 - 4.16)	5.36 (4.42 - 6.45)	1.69 (1.17 - 2.33)	1.34 (0.97 - 1.79)	10.76 (8.78 - 12.72)	12.01 (10.02 - 14.09)	6.29 (4.62 - 8.22)	6.42 (4.94 - 8.02)
Tunisia	Middle SDI	27.2 (24.46 - 30.46)	29.04 (26.6 - 31.58)	5.37 (4.51 - 6.32)	13.55 (11.76 - 15.46)	40.06 (37.16 - 42.39)	33.42 (31.63 - 35.1)	12.08 (10.69 - 13.77)	23.25 (20.82 - 25.79)	6.01 (4.43 - 7.98)	7.37 (5.74 - 9.29)	1.42 (1 - 2)	2.05 (1.38 - 2.84)	13.04 (9.9 - 16.46)	13.46 (10.65 - 16.5)	4.1 (2.89 - 5.53)	4.23 (3.05 - 5.77)
Turkey	High-middle SDI	36.38 (33.62 - 39.28)	29.95 (28.04 - 31.6)	10.95 (9.6 - 12.38)	21.46 (19.05 - 23.85)	44.85 (43.3 - 46.15)	32.4 (31.93 - 32.86)	20.96 (19.33 - 22.66)	35.02 (33.18 - 36.96)	7.04 (5.16 - 9.3)	9.03 (7.03 - 11.6)	1.63 (1.12 - 2.28)	1.94 (1.37 - 2.66)	12.66 (10.03 - 15.51)	11.02 (8.75 - 14.2)	6.97 (5.2 - 9.01)	5.85 (4.2 - 7.77)
Turkmenistan	High-middle SDI	35.3 (33.4 - 37.29)	29.75 (28.53 - 30.97)	9.35 (7.77 - 11.1)	17.7 (15.97 - 19.55)	41.58 (40.66 - 42.08)	32.47 (31.84 - 33.63)	17.04 (14.32 - 19.97)	26 (23.83 - 28.13)	10.33 (8.39 - 12.61)	11.04 (9.21 - 12.88)	4.06 (2.68 - 5.85)	3.54 (2.59 - 4.61)	15.44 (12.79 - 18.17)	13.83 (11.65 - 15.84)	7.38 (5.2 - 10)	5.82 (4.43 - 7.44)
Uganda	Low SDI	8.15 (6.11 - 10.52)	13.64 (11.81 - 15.57)	1.56 (0.83 - 2.59)	3.75 (2.7 - 4.94)	10.87 (9.17 - 12.88)	25.53 (23.5 - 27.47)	2.43 (1.84 - 3.2)	12.68 (10.99 - 14.49)	6.28 (3.7 - 9.51)	8.78 (6.22 - 11.45)	1.79 (0.78 - 3.52)	1.82 (1.03 - 3.11)	4.61 (2.61 - 6.88)	10.5 (7.69 - 13.94)	1.33 (0.61 - 2.61)	2.36 (1.37 - 3.52)
Ukraine	High-middle SDI	38.41 (37.13 - 39.62)	30.35 (29.84 - 30.9)	11.33 (9.89 - 12.9)	23.57 (21.42 - 25.73)	38.91 (37.62 - 39.92)	30.36 (29.9 - 30.86)	13.35 (11.87 - 15.09)	26.06 (23.86 - 28.17)	7.52 (6.24 - 9.01)	5.49 (4.43 - 6.69)	1.95 (1.41 - 2.58)	1.46 (1.03 - 1.96)	10.66 (9.04 - 12.45)	7.06 (5.73 - 8.57)	3.13 (2.23 - 4.22)	2 (1.46 - 2.69)
United Arab Emirates	High SDI	38.48 (37.18 - 39.89)	28.87 (27.58 - 30.1)	25.19 (22.55 - 27.54)	31.2 (28.11 - 34.14)	38.09 (36.4 - 39.81)	31.43 (30.31 - 32.49)	25.66 (23.21 - 28.19)	31.63 (28.86 - 34.55)	17.16 (13.88 - 20.73)	18.13 (14.23 - 21.67)	10.48 (7.99 - 13.42)	9.72 (7.3 - 12.68)	16.27 (12.91 - 19.49)	17.58 (13.99 - 21.56)	11.33 (8.54 - 14.24)	10.28 (7.87 - 13.32)
United Kingdom	High SDI	42.88 (42.35 - 43.37)	30.33 (29.83 - 30.78)	14.8 (14.07 - 15.51)	17.55 (16.78 - 18.33)	43.51 (43.36 - 43.63)	31.98 (31.73 - 32.18)	23.4 (22.64 - 24.12)	24.88 (24.15 - 25.67)	13.46 (10.65 - 16.55)	16.19 (13.31 - 19.4)	5.03 (3.51 - 6.84)	5.86 (4.31 - 7.79)	18.97 (15.46 - 22.8)	19.95 (16.6 - 23.58)	7.03 (4.93 - 9.64)	8.01 (6.14 - 10.19)
United States	High SDI	40.34 (39.99 - 40.65)	26.35 (26.17 - 26.57)	10.67 (10.31 - 11.03)	14.98 (14.57 - 15.4)	39.84 (39.95 - 39.73)	29.46 (29.47 - 29.4)	30.66 (30.07 - 31.27)	35.45 (34.92 - 36)	12.2 (11.81 - 12.71)	11.61 (11.21 - 11.99)	5 (4.68 - 5.32)	5.11 (4.8 - 5.45)	15.79 (15.39 - 16.14)	15.82 (15.34 - 16.22)	12.99 (12.29 - 13.72)	12.4 (11.79 - 13.06)
Uruguay	High-middle SDI	40.82 (37.52 - 43.78)	31.15 (29.21 - 32.89)	15.03 (12.74 - 17.14)	19.85 (17.47 - 22.42)	41.96 (39.32 - 44.29)	31.82 (30.3 - 33.41)	18.99 (16.52 - 21.54)	23.64 (21.02 - 26.21)	9.42 (6.68 - 12.6)	11.83 (9.82 - 13.46)	3.15 (2.01 - 4.65)	9.28 (6.97 - 12.48)	14.84 (10.75 - 19.27)	13.63 (11.53 - 15.82)	5.57 (3.69 - 7.98)	11.06 (8.45 - 14.05)
Uzbekistan	High-middle SDI	36.23 (34.58 - 37.49)	27.39 (25.62 - 28.76)	14.31 (12.17 - 16.75)	13.15 (11.8 - 14.66)	36.65 (36.14 - 37.14)	30.93 (30.36 - 31.26)	17.4 (16.58 - 18.29)	27.99 (26.66 - 29.42)	12.13 (10.34 - 14.05)	9.96 (8.42 - 11.48)	9.91 (7.18 - 13.05)	6.02 (4.58 - 7.82)	13.96 (11.63 - 16.52)	13.35 (11.28 - 15.43)	6.99 (5.09 - 9.45)	6.46 (5.05 - 8.07)
Vanuatu	Low-middle SDI	22.15 (19.38 - 25.17)	18.29 (16.16 - 20.2)	7.79 (6.34 - 9.49)	19.08 (15.89 - 22.43)	29.15 (27.39 - 31.02)	33.91 (32.94 - 34.93)	13.33 (12.04 - 14.74)	23.33 (20.82 - 25.91)	5.76 (4.06 - 7.64)	7.6 (5.38 - 9.99)	2.86 (1.76 - 4.38)	5.49 (3.39 - 8.46)	8.08 (5.8 - 10.55)	12.79 (9.03 - 16.76)	4.31 (2.81 - 6.3)	5.64 (3.5 - 8.42)
Venezuela	High-middle SDI	20 (17.63 - 22.35)	27.34 (25.23 - 29.33)	6.71 (5.21 - 8.48)	15.88 (13.33 - 18.97)	39.6 (38.12 - 40.58)	35.88 (35.35 - 36.54)	19.18 (15.53 - 23.15)	30.61 (26.64 - 34.29)	10.44 (7.87 - 13.26)	13.47 (10.29 - 16.82)	3.32 (2.02 - 4.9)	3.74 (2.48 - 5.29)	16.17 (13.24 - 19.09)	18.65 (15.1 - 22.59)	8 (5.33 - 11.42)	8.46 (5.97 - 11.62)
Vietnam	Middle SDI	3.03 (2.39 - 3.87)	4.98 (4.16 - 5.97)	0.14 (0.1 - 0.19)	0.58 (0.44 - 0.78)	10.55 (8.98 - 12.25)	11.62 (10.22 - 13.23)	1.23 (0.95 - 1.56)	2.03 (1.61 - 2.53)	1.17 (0.8 - 1.6)	1.28 (0.91 - 1.75)	0.36 (0.22 - 0.59)	0.48 (0.29 - 0.75)	4.8 (3.49 - 6.42)	4.48 (3.27 - 5.79)	2.36 (1.56 - 3.39)	1.94 (1.21 - 2.96)
Virgin Islands, U.S.	High SDI	27.5 (25.2 - 29.91)	31.91 (30.25 - 33.81)	10.6 (9.17 - 12.14)	22.2 (20.13 - 24.23)	42.25 (40.98 - 43.09)	31.23 (30.85 - 31.83)	28.06 (25.52 - 30.71)	43.34 (40.69 - 45.79)	13.02 (10.8 - 15.34)	16.28 (14.08 - 18.42)	7.44 (5.45 - 9.77)	9.85 (7.76 - 12.36)	19.35 (17.13 - 21.54)	20.53 (18.81 - 22.81)	15.81 (12.37 - 19.26)	18.62 (15.29 - 21.85)
Yemen	Low-middle SDI	13.5 (11.57 - 15.64)	19.91 (17.29 - 22.65)	1.9 (1.56 - 2.29)	4.79 (3.94 - 5.78)	24.47 (21.85 - 27.26)	25.35 (23.89 - 26.82)	5.34 (4.5 - 6.29)	12.77 (11.52 - 14.21)	2.69 (1.98 - 3.58)	9.27 (7.12 - 11.63)	0.45 (0.29 - 0.64)	2.48 (1.73 - 3.37)	5.13 (3.78 - 6.71)	8.62 (6.57 - 10.97)	1.28 (0.88 - 1.79)	3.73 (2.64 - 5.16)
Zambia	Low-middle SDI	20.85 (17.59 - 23.9)	21.28 (19.28 - 23.51)	9.14 (6.02 - 13.07)	9.09 (7.04 - 11.17)	17.66 (14.54 - 20.3)	24.98 (23.64 - 25.98)	6.81 (4.54 - 9.59)	14.22 (12.03 - 16.8)	14.25 (9.78 - 19.09)	12.49 (9.33 - 15.41)	7.28 (3.87 - 11.91)	4.57 (2.66 - 7.06)	15.01 (11 - 19.73)	16.95 (13.99 - 19.41)	11.56 (7.12 - 17.59)	9.22 (5.77 - 13.66)
Zimbabwe	Low-middle SDI	17.75 (15.33 - 20.45)	23.36 (22.34 - 24.61)	5.85 (4.57 - 7.35)	14.77 (13.04 - 16.54)	14.83 (12.83 - 16.91)	24.74 (23.89 - 25.52)	4.83 (3.88 - 5.87)	19.16 (17.18 - 21.16)	8.99 (6.44 - 12.04)	10.14 (8.39 - 11.96)	2.86 (1.8 - 4.26)	2.4 (1.76 - 3.24)	5.62 (3.94 - 7.93)	11.13 (9.18 - 13.2)	1.86 (1.19 - 2.72)	2.29 (1.66 - 3.03)

Table S4. Disability adjusted life years (DALYs), deaths, and years lived with disability (YLDs) attributable to high body mass index (BMI) caused by the below causes in males and females in 1990 and 2015. Rates presented as number per 100,000 at the Global level for all adults over the age of 25, age-standardized.

Cause	1990 DALYs		2015 DALYs		1990 Deaths		2015 Deaths		1990 YLDs		2015 YLDs	
	Males	Females	Males	Females	Males	Females	Males	Females	Males	Females	Males	Females
Ischemic heart disease	674.06 (374.26 - 1012.62)	441.67 (279.54 - 624.03)	597.59 (350.59 - 870.58)	360.63 (240.5 - 491.5)	30.21 (16.27 - 46.88)	23.49 (14.3 - 34.15)	25.44 (14.49 - 38.17)	18.65 (11.85 - 26.53)	20.15 (9.34 - 35.54)	18.16 (9.95 - 29.08)	23.42 (11.47 - 39.59)	20.31 (11.53 - 31.72)
Ischemic stroke	133.92 (69.47 - 213.4)	140.95 (87.91 - 209.12)	116.91 (64.62 - 177.7)	99.76 (64.73 - 141.92)	6.19 (3.11 - 10.32)	6.98 (4.05 - 10.98)	5.13 (2.71 - 8.33)	4.8 (2.84 - 7.42)	9.03 (4.01 - 16.26)	10.09 (5.56 - 16.96)	10.51 (5.1 - 17.71)	10.87 (6.33 - 17.44)
Hemorrhagic stroke	291.15 (148.11 - 467.83)	309.29 (186.37 - 454.3)	302.92 (174.16 - 443.92)	243.39 (161.33 - 335.02)	10.21 (4.99 - 16.88)	11.03 (6.4 - 16.81)	10.23 (5.59 - 15.62)	8.61 (5.46 - 12.37)	8.27 (3.85 - 14.63)	10.56 (5.88 - 17.35)	10.75 (5.53 - 17.83)	11.5 (6.86 - 17.91)
Hypertensive heart disease	88.7 (42.88 - 148.14)	102.72 (58.83 - 154.28)	90.99 (52.06 - 137.94)	93.2 (59.61 - 128.6)	4.23 (1.84 - 7.6)	5.26 (2.68 - 8.47)	4.4 (2.22 - 7.34)	5.1 (2.79 - 7.71)	2.15 (0.88 - 4.08)	2.89 (1.48 - 4.88)	3.04 (1.39 - 5.39)	3.88 (2.08 - 6.28)
Diabetes mellitus	263.9 (150.09 - 400.29)	321.24 (212.24 - 451.08)	419 (260.66 - 598.06)	435.92 (309.22 - 588.74)	5.36 (3.02 - 8.02)	6.9 (4.59 - 9.46)	8.04 (4.93 - 11.47)	8.61 (6.02 - 11.47)	137.85 (68.76 - 225.53)	165.59 (98.85 - 254.06)	229.05 (125.88 - 352.47)	245.55 (153.55 - 361.77)
Chronic kidney disease	92.04 (45.88 - 149.24)	96.69 (56.26 - 146.39)	129.17 (70.53 - 197.9)	123.96 (77.1 - 176.01)	3.33 (1.56 - 5.62)	3.2 (1.67 - 4.98)	4.85 (2.45 - 7.7)	4.51 (2.5 - 6.83)	23.43 (11.5 - 39.59)	29.1 (16.37 - 45.16)	29.06 (15.5 - 46.42)	33.71 (19.94 - 50.8)
Esophageal cancer	36.89 (6.28 - 82.55)	16.67 (0.62 - 37.75)	36.34 (6.86 - 74.45)	12.25 (0.49 - 25.58)	1.61 (0.27 - 3.61)	0.75 (0.03 - 1.71)	1.65 (0.31 - 3.41)	0.58 (0.02 - 1.23)	0.39 (0.07 - 0.88)	0.18 (0.01 - 0.41)	0.49 (0.09 - 1.04)	0.18 (0.01 - 0.38)
Colon and rectum cancer	29.43 (14.81 - 46.42)	9.63 (4.24 - 17.23)	31.52 (17.01 - 48.05)	8.95 (4.06 - 15.68)	1.49 (0.74 - 2.38)	0.49 (0.21 - 0.87)	1.59 (0.85 - 2.44)	0.46 (0.2 - 0.8)	1.14 (0.52 - 1.94)	0.36 (0.15 - 0.67)	1.59 (0.79 - 2.6)	0.44 (0.19 - 0.81)
Liver cancer	39.98 (9.72 - 90.75)	13.7 (2.14 - 31.98)	54.14 (15.26 - 109.67)	14.26 (2.41 - 30.8)	1.59 (0.4 - 3.56)	0.57 (0.09 - 1.33)	2.17 (0.61 - 4.41)	0.65 (0.11 - 1.41)	0.32 (0.07 - 0.75)	0.12 (0.02 - 0.3)	0.51 (0.14 - 1.05)	0.17 (0.03 - 0.38)
Gallbladder and biliary tract cancer	3.46 (0.59 - 7.54)	12.88 (6.94 - 20.19)	2.91 (0.51 - 6.23)	8.71 (4.89 - 13.44)	0.18 (0.03 - 0.4)	0.66 (0.35 - 1.04)	0.16 (0.03 - 0.34)	0.46 (0.25 - 0.72)	0.05 (0.01 - 0.11)	0.17 (0.08 - 0.29)	0.05 (0.01 - 0.1)	0.13 (0.07 - 0.22)
Pancreatic cancer	5.68 (-0.09 - 14.14)	6.07 (2.24 - 11.3)	6.49 (-0.1 - 15.53)	7.1 (2.71 - 12.77)	0.27 (0 - 0.68)	0.32 (0.12 - 0.59)	0.32 (-0.01 - 0.77)	0.38 (0.14 - 0.68)	0.05 (0 - 0.14)	0.06 (0.02 - 0.13)	0.07 (0 - 0.17)	0.08 (0.03 - 0.16)
Breast cancer	-	15.96 (8.49 - 25.9)	-	21.77 (12.39 - 34.07)	-	0.79 (0.44 - 1.24)	-	0.98 (0.58 - 1.49)	-	1.84 (0.9 - 3.18)	-	3.09 (1.61 - 5.17)
Uterine cancer	-	22.12 (14.07 - 31.48)	-	21.68 (14.96 - 28.92)	-	0.92 (0.58 - 1.29)	-	0.88 (0.61 - 1.17)	-	1.72 (1.01 - 2.68)	-	2.68 (1.66 - 4.07)
Ovarian cancer	-	3.42 (-0.11 - 7.88)	-	3.41 (-0.12 - 7.58)	-	0.14 (0 - 0.32)	-	0.14 (0 - 0.31)	-	0.11 (0 - 0.27)	-	0.12 (0 - 0.29)
Kidney cancer	8.12 (4.2 - 13.06)	6.23 (3.78 - 9.16)	9.59 (5.32 - 15)	6.35 (4.1 - 8.95)	0.37 (0.19 - 0.6)	0.29 (0.17 - 0.42)	0.44 (0.24 - 0.7)	0.3 (0.19 - 0.42)	0.31 (0.14 - 0.55)	0.25 (0.14 - 0.42)	0.64 (0.31 - 1.08)	0.5 (0.3 - 0.79)
Thyroid cancer	0.98 (0.28 - 2.01)	1.17 (0.61 - 1.94)	1.39 (0.42 - 2.76)	1.24 (0.68 - 1.93)	0.04 (0.01 - 0.09)	0.05 (0.03 - 0.09)	0.05 (0.02 - 0.1)	0.05 (0.03 - 0.07)	0.08 (0.02 - 0.16)	0.11 (0.06 - 0.21)	0.36 (0.1 - 0.71)	0.32 (0.17 - 0.55)
Leukemia	5.86 (2.44 - 10.92)	7.18 (2.99 - 13.26)	6.84 (3.13 - 12.22)	7.69 (3.43 - 13.52)	0.25 (0.11 - 0.47)	0.29 (0.12 - 0.54)	0.3 (0.14 - 0.54)	0.32 (0.14 - 0.56)	0.22 (0.08 - 0.42)	0.24 (0.09 - 0.45)	0.32 (0.14 - 0.61)	0.34 (0.14 - 0.61)
Osteoarthritis	17.21 (7.73 - 30.65)	32.13 (17.42 - 52.46)	24.57 (11.76 - 42.2)	43.58 (24.85 - 69.51)	-	-	-	-	17.21 (7.73 - 30.65)	32.13 (17.42 - 52.46)	24.57 (11.76 - 42.2)	43.58 (24.85 - 69.51)
Low back pain	33.61 (14.47 - 62.1)	42.68 (21.97 - 74.73)	40.66 (19.07 - 72.26)	50.64 (27.81 - 84.65)	-	-	-	-	33.61 (14.47 - 62.1)	42.68 (21.97 - 74.73)	40.66 (19.07 - 72.26)	50.64 (27.81 - 84.65)

Table S5. Decomposition of percent changes in cause-specific deaths and disability-adjusted life years (DALYs) related to high body mass index from 1990 to 2015 due to population growth, population ageing, risk exposure and the underlying rates of deaths and DALYs by quintiles of Socio−demographic Index.

**Deaths**	**Low SDI**	**Low-middle SDI**	**Middle SDI**	**High-middle SDI**	**High SDI**
Cardiovascular Diseases					
% change due to population ageing	-1.26	34.71	59.19	43.7	21.65
% change due to population growth	125.05	74.34	41.71	21.29	7.59
% change due to risk exposure	60.28	78.94	76.29	35.93	23.22
% change due to risk-deleted mortality rate	-41.17	-40.31	-71.43	-63.86	-57.14
Total % change	142.9	147.68	105.77	37.05	-4.68
Diabetes Mellitus					
% change due to population ageing	-5	45.92	70.32	54.77	26.18
% change due to population growth	140.08	92.22	60.83	45.52	14.91
% change due to risk exposure	96.21	105.64	105.44	45.98	39.85
% change due to risk-deleted mortality rate	-18.44	31.3	-21.08	-46.51	-59.3
Total % change	212.84	275.09	215.51	99.75	21.64
Neoplasms					
% change due to population ageing	-6.43	32.8	75.98	56.57	28.75
% change due to population growth	126.68	76.4	26.57	27.67	11.88
% change due to risk exposure	80.73	77.86	86.7	49.82	25.97
% change due to risk-deleted mortality rate	-33.68	-31.4	-62.94	-46.97	-24.11
Total % change	167.3	155.65	126.31	87.09	42.49
**DALYs**	**Low SDI**	**Low-middle SDI**	**Middle SDI**	**High-middle SDI**	**High SDI**
Cardiovascular Diseases					
% change due to population ageing	-0.93	34.53	59.27	47.01	24.45
% change due to population growth	128.18	77.64	45.19	25.89	9.75
% change due to risk exposure	60.04	85.16	84.96	43.95	29.01
% change due to risk-deleted DALY rate	-43.37	-44.59	-78.19	-73.99	-64.7
Total % change	143.92	152.75	111.23	42.87	-1.49

Table S5. Decomposition of percent changes in cause-specific deaths and disability-adjusted life years (DALYs) related to high body mass index from 1990 to 2015 due to population growth, population ageing, risk exposure and the underlying rates of deaths and DALYs by quintiles of Socio−demographic Index.

Diabetes Mellitus					
% change due to population ageing	-3	45.51	70.77	60.92	31.73
% change due to population growth	147.1	95.43	64.98	51.83	21.92
% change due to risk exposure	89.39	116.9	119.9	65.25	61.79
% change due to risk-deleted DALY rate	-7.55	18.65	-25.83	-35.34	-47.3
Total % change	225.94	276.49	229.82	142.67	68.14
Neoplasms					
% change due to population ageing	-5.85	33.2	76.29	60.79	32.94
% change due to population growth	129.03	79.77	28.37	30.79	14.1
% change due to risk exposure	82.2	82.87	94.42	57.59	30.07
% change due to risk-deleted DALY rate	-36.3	-34.11	-64.53	-53.09	-25.64
Total % change	169.07	161.73	134.56	96.08	51.47
Musculoskeletal disorders					
% change due to population ageing	-3.5	35.81	70.46	54.23	23.74
% change due to population growth	137.06	82.99	52.06	35.28	15.27
% change due to risk exposure	72.32	81.12	83.59	42.83	32.42
% change due to risk-deleted DALY rate	2.19	-5.54	-2.59	-2.18	-4.12
Total % change	208.07	194.38	203.51	130.17	67.32

Table S6. Disability-adjusted life years (DALYs) and mortality rates attributable to high body mass index (BMI) in age-standardized males and females in 1990 and 2015 among 195 countries. Rates presented as number of deaths and DALYs per 100,000.

Country	1990 Deaths		2015 Deaths		1990 DALYs		2015 DALYs	
	Males	Females	Males	Females	Males	Females	Males	Females
Afghanistan	166.87 (80.88 - 283.78)	296.28 (170.86 - 460.01)	160.45 (78.57 - 268.15)	292.09 (166.35 - 450.74)	4767.49 (2400.07 - 7893.78)	8437.91 (4934.18 - 12974.5)	4677.45 (2411.08 - 7598.24)	8491.89 (5022.71 - 13139.81)
Albania	67.07 (33.24 - 106.17)	45.15 (24.95 - 68.65)	76.44 (43.78 - 114.91)	47.21 (28.08 - 67.82)	1693.85 (870.18 - 2592.06)	1164.71 (675.02 - 1696.52)	2040.08 (1228.81 - 2911.72)	1250.38 (803.29 - 1722.95)
Algeria	68.16 (35.08 - 107.09)	99.85 (67.23 - 135.31)	83.08 (52.84 - 119.44)	121.92 (92.75 - 156.04)	2102.72 (1156.86 - 3168.52)	3108.69 (2291.23 - 4022.68)	2637.58 (1808.99 - 3554.99)	3668.29 (2926.85 - 4496.76)
American Samoa	282.03 (218.61 - 345.89)	194.8 (158.61 - 233.08)	244.71 (180.79 - 313.55)	226.3 (169.83 - 290.15)	8274.29 (6604.11 - 10017.83)	5366.11 (4491.37 - 6348.11)	7224.62 (5612.79 - 9021.73)	6322.02 (4961 - 7876.81)
Andorra	38.59 (18.5 - 61.48)	17.48 (8.99 - 27.51)	28.82 (15.88 - 44.48)	11.07 (5.43 - 17.88)	1018.09 (527.6 - 1563.26)	516.56 (286.94 - 790.81)	845.65 (510.67 - 1204.28)	393.55 (216.1 - 618.1)
Angola	45.31 (7.28 - 127.09)	68.19 (16.34 - 168.53)	88.55 (26.45 - 237.07)	149.64 (42.75 - 376.46)	1362.84 (237.82 - 3808.45)	1681.98 (432.43 - 4290.26)	2578.44 (843.84 - 6820.75)	3920.97 (1250.62 - 9963.6)
Antigua and Barbuda	77.1 (40.53 - 120.12)	96.04 (61.29 - 133.93)	79.95 (44.64 - 120.15)	91.52 (62.91 - 121.32)	2306.3 (1291.04 - 3434.62)	2657.58 (1826.17 - 3514.24)	2348.21 (1440.35 - 3285.02)	2541.95 (1909.01 - 3235.57)
Argentina	121.84 (77.24 - 167.19)	82.48 (55.92 - 108.65)	94.64 (61.07 - 127.71)	60.21 (42.14 - 78.99)	3049.31 (2011.51 - 4072.53)	2061.46 (1477.52 - 2635.56)	2364.23 (1622.3 - 3078.99)	1535.51 (1130.12 - 1945.6)
Armenia	95.42 (49.32 - 148.64)	113.9 (77.05 - 153.99)	118.64 (71.2 - 168.73)	105.37 (76.16 - 136.49)	2497.03 (1371.22 - 3744.95)	2621.08 (1878.24 - 3386.56)	3276.44 (2077.67 - 4460.77)	2683.16 (2047.21 - 3355.45)
Australia	73.03 (42.55 - 106.6)	50.26 (32.73 - 69.29)	44.7 (29.13 - 61.47)	34.8 (24.12 - 46.24)	1802.66 (1107.21 - 2520.58)	1228.27 (858.05 - 1640.37)	1295.7 (890.56 - 1715.91)	1029.6 (777.47 - 1322.39)
Austria	87.31 (49.64 - 126.5)	54.67 (34 - 76.91)	54.23 (32.58 - 76.99)	39.02 (25.6 - 53.9)	2037.93 (1203.15 - 2857.83)	1215.56 (812.49 - 1641.27)	1337.93 (852.89 - 1824.32)	872.18 (610.91 - 1163.24)
Azerbaijan	140.41 (69.24 - 218.87)	149.65 (99.5 - 204.04)	137.3 (80.41 - 197.05)	122.07 (88.39 - 157.07)	3442.38 (1753.96 - 5252.77)	3403.73 (2439.68 - 4375.73)	3598.09 (2176 - 4990.76)	3157.04 (2418.95 - 3927.35)
Bahrain	133.15 (84.79 - 191.67)	175.15 (129.1 - 222.44)	97.7 (62.06 - 141.26)	99.15 (68.94 - 130.35)	4039.88 (2769.77 - 5453.55)	4907.81 (3800.69 - 6091.08)	3109.58 (2127.83 - 4224.11)	3177.75 (2414.84 - 4028.72)
Bangladesh	13.5 (3.26 - 30.84)	19.07 (5.22 - 42.54)	37.64 (13.28 - 73.96)	37.92 (15.85 - 70.73)	444.23 (116.48 - 967.64)	564.76 (158.87 - 1216.55)	1234.47 (480.89 - 2288.54)	1170.47 (529.42 - 2024.95)
Barbados	96.07 (55.55 - 140.91)	104.02 (71.56 - 137.56)	85.6 (54.87 - 121.05)	111.43 (84.2 - 142.01)	2831.69 (1772.18 - 3926.16)	2953.24 (2156.85 - 3792.11)	2676.88 (1829.98 - 3538.15)	3167.41 (2525.52 - 3875.71)
Belarus	149.01 (90.54 - 210.69)	114.91 (80.13 - 152.62)	199.3 (126.24 - 273.9)	113.55 (80.17 - 149.46)	3837.23 (2391.03 - 5249.27)	2677.73 (1989.91 - 3395.44)	4947.93 (3239.5 - 6632.98)	2620.93 (1966.71 - 3281.37)
Belgium	67.89 (37.45 - 100.53)	46.13 (28.94 - 65.08)	41.75 (24.77 - 59.59)	26.97 (17.6 - 36.9)	1714.48 (980.56 - 2461.55)	1154.11 (758.16 - 1604.46)	1193.49 (749.99 - 1651.74)	826.53 (574.87 - 1119.64)
Belize	85.09 (50.93 - 123.72)	116.86 (83.39 - 152.33)	109.42 (70.96 - 153.13)	136.03 (101.92 - 172.1)	2475.9 (1600.73 - 3414.51)	3194.68 (2399.45 - 3995.25)	3122.35 (2122.01 - 4171.21)	3735.19 (2941.31 - 4605.26)
Benin	26.46 (9.01 - 52.29)	39.36 (19.12 - 64.67)	92.56 (33.97 - 192.65)	112.36 (43.86 - 233.95)	816.94 (295.98 - 1576.44)	1047.67 (539.06 - 1670.68)	2690.32 (1051.46 - 5529.48)	2842.63 (1211.08 - 5766.79)
Bermuda	112.25 (61.38 - 167.84)	89.46 (57.95 - 122.59)	70.5 (42.71 - 101.05)	50.91 (34.13 - 69.7)	3220.42 (1855.25 - 4598.41)	2465.38 (1723.01 - 3261.2)	2045.66 (1316.67 - 2803.49)	1506.16 (1129.33 - 1969.57)
Bhutan	31.38 (10.4 - 62.72)	62.72 (28.06 - 116.79)	49.53 (21.06 - 90.32)	63.52 (31.75 - 102.36)	952.57 (345.06 - 1852.57)	1732.63 (800.43 - 3062.1)	1532.2 (712.98 - 2617.74)	1853.75 (1056.07 - 2805.43)
Bolivia	71.58 (39.69 - 106.24)	82.76 (49.9 - 120.33)	60.45 (34.16 - 92.66)	92.29 (59.68 - 130.84)	2189.42 (1302.45 - 3100.42)	2392.2 (1537.88 - 3341.88)	1869.7 (1121.46 - 2759.11)	2481.92 (1743.71 - 3345.03)
Bosnia and Herzegovina	80.56 (42.52 - 123.06)	72.78 (45.91 - 100.53)	64.9 (38.97 - 93.16)	54.74 (36.96 - 73.48)	2256.93 (1279.56 - 3296.63)	1891.71 (1246.16 - 2556.95)	1952.75 (1228.86 - 2690.97)	1547.51 (1085.89 - 2049.31)
Botswana	46.67 (11.33 - 124.73)	127.87 (41.75 - 309.02)	98.88 (26.34 - 281.81)	208.88 (76.81 - 642.13)	1468.55 (415.24 - 3686.86)	3558.22 (1394.01 - 8356.2)	3103.16 (959.1 - 8574.19)	5679.74 (2467.56 - 17174.47)
Brazil	93.78 (49.44 - 143.68)	90.76 (58.72 - 124.48)	87.4 (55.12 - 120.75)	75.67 (54.37 - 97.69)	2642.58 (1478.24 - 3870.58)	2538.92 (1741.35 - 3354.51)	2457.46 (1665.69 - 3247.79)	2093.18 (1586.49 - 2619.7)
Brunei	49.43 (19.86 - 86.42)	60.86 (31.99 - 95.91)	53.57 (25.56 - 85.77)	50.24 (28.26 - 74.15)	1559.58 (672.26 - 2616.38)	1806.17 (992.47 - 2753.88)	1617.02 (832.5 - 2494.84)	1439.09 (860.64 - 2071.69)
Bulgaria	185.49 (120.08 - 250.36)	138.64 (97.46 - 179.97)	133.95 (79.92 - 187.93)	88.19 (58.35 - 119.32)	4615.63 (3115.67 - 5996.83)	3159.5 (2333.15 - 3983.41)	3380.52 (2097.87 - 4605.07)	2105.47 (1477.87 - 2756.57)
Burkina Faso	13.8 (3.4 - 31.76)	19.32 (6.67 - 39.19)	46.84 (17.41 - 97.24)	71.35 (29.01 - 144.12)	437.55 (115.36 - 979.34)	501.36 (175.05 - 1024.18)	1371.47 (552.84 - 2773.34)	1809.71 (828.35 - 3499.87)
Burundi	43.81 (10.84 - 105.1)	76.85 (26.49 - 154.77)	14.99 (2.72 - 42.33)	28.95 (7.93 - 68.39)	1417.69 (362.59 - 3402.08)	2159.26 (759.94 - 4236.41)	451.93 (89.41 - 1229.7)	799.72 (250.65 - 1815.81)
Cambodia	27.11 (7.29 - 59.88)	44.09 (17.03 - 81.7)	42.03 (15.03 - 81.14)	44.65 (19.35 - 78.02)	835.43 (240.09 - 1801.09)	1218.78 (465.33 - 2251)	1203.34 (451.63 - 2270.1)	1211.87 (532.21 - 2100.78)
Cameroon	45.47 (19.6 - 80.65)	80.12 (46.45 - 121.48)	88.83 (36.09 - 185.19)	128.85 (54.34 - 267.12)	1336.31 (594.93 - 2301.41)	2143.94 (1321.3 - 3107.45)	2568.35 (1130.56 - 5288.09)	3313.05 (1547.61 - 6571.49)
Canada	62.45 (34.66 - 92.65)	37.22 (22.71 - 53.63)	44.37 (27.86 - 61.66)	27.81 (18.11 - 38.05)	1731.91 (1017.28 - 2471.59)	1119.4 (747.21 - 1547.48)	1334.88 (873.16 - 1793.41)	930.14 (648.14 - 1239.68)
Cape Verde	37.82 (15.99 - 67.45)	41.74 (23.57 - 62.82)	58.16 (28.43 - 102.58)	64.86 (40.2 - 98.44)	1186.07 (527.39 - 2026.45)	1243.13 (745.21 - 1808.94)	1744.94 (934.05 - 2869.18)	1868.57 (1250.25 - 2692.75)
Central African Republic	64.34 (25.87 - 120.41)	44.27 (14.53 - 88.27)	90.28 (21.29 - 205.12)	120.49 (30.08 - 282.31)	1936.57 (802.22 - 3539.22)	1122.29 (384.57 - 2198.62)	2802.86 (765.65 - 6327.03)	3233.64 (954.51 - 7570.69)
Chad	19.1 (6.78 - 38.35)	28.73 (12.69 - 53.11)	64.21 (20.97 - 146.39)	101.71 (33.62 - 232.15)	613.74 (228.92 - 1193.4)	728.29 (313.78 - 1347.12)	1982.69 (730.96 - 4339.42)	2702.64 (1059.54 - 5972.82)
Chile	96.31 (60.13 - 134.23)	83.08 (56.48 - 110.38)	67.51 (41.81 - 93.33)	53.35 (36.79 - 71.09)	2342.6 (1527.2 - 3144.93)	2011.49 (1447.87 - 2565.75)	1665.55 (1098.5 - 2221.75)	1348.73 (988.37 - 1733.36)
China	37.75 (12.29 - 74.78)	35.81 (14.87 - 64.92)	49.07 (22.02 - 82.34)	33.66 (17.72 - 53.01)	998.51 (329.11 - 1941.12)	986.18 (416.75 - 1747.66)	1392.88 (670.2 - 2249.33)	929.69 (504.13 - 1435.65)
Colombia	55.12 (26.97 - 88.85)	69.8 (42.48 - 99.22)	47.8 (26.26 - 71.65)	48.75 (30.89 - 67.67)	1549.46 (805.85 - 2390.86)	1892.67 (1217.59 - 2597.71)	1369.78 (827.38 - 1935.08)	1391.62 (976.29 - 1854.95)
Comoros	52.14 (14.07 - 120.78)	63.03 (20.45 - 141.32)	34.18 (10.96 - 78.45)	60.24 (23.28 - 111.55)	1705.79 (534.69 - 3678.89)	1749.55 (618.92 - 3763.81)	1175.4 (449.13 - 2486.86)	1852.3 (873.5 - 3155.14)
Congo	101.47 (40.57 - 183.6)	137.85 (73.54 - 226.72)	71.46 (27.65 - 157.06)	172.85 (73.2 - 326.76)	2822.57 (1160.39 - 4975.39)	3392.79 (1803.68 - 5641.98)	2048.45 (854.89 - 4228.07)	4420.66 (2066.68 - 8045.85)
Costa Rica	52.68 (28.2 - 79.81)	52.87 (32.78 - 72.85)	49.81 (30.42 - 71.88)	44.99 (30.02 - 60.76)	1408.8 (807.48 - 2032.88)	1508.13 (1013.43 - 2025.01)	1399.18 (910.26 - 1902.36)	1327.32 (959.85 - 1722.62)
Cote d'Ivoire	29.12 (9.62 - 60.01)	35.56 (15.38 - 65.73)	55.95 (16.48 - 123.76)	75.66 (27.83 - 153.27)	886.86 (301.54 - 1779.76)	1040.01 (491.09 - 1808.63)	1828 (606.4 - 3867.63)	2172.11 (922.87 - 4189.08)
Croatia	118.66 (67.42 - 170.83)	88.35 (54.5 - 122.64)	85.83 (52.69 - 120.97)	58.03 (37.01 - 80.22)	2980.64 (1804.98 - 4144.68)	1979.34 (1306.55 - 2661.8)	2164.11 (1370.51 - 2940.75)	1341.02 (900.74 - 1828.79)

Cuba	61.33 (32.94 - 92.66)	77.62 (52.69 - 102.49)	55.45 (32.34 - 81.15)	58.86 (40.99 - 77.61)	1771.83 (1008.69 - 2600.09)	2307.43 (1684.96 - 2939.11)	1600.61 (990.24 - 2235.73)	1731.62 (1292.9 - 2187.46)
Cyprus	97.76 (57.4 - 141.78)	60.85 (40.47 - 84.72)	57.56 (34.88 - 81.72)	27.82 (18.14 - 38.05)	2301.62 (1460.93 - 3133.88)	1478.57 (1056.83 - 1947.85)	1628.73 (1048.66 - 2214.83)	907.02 (633.97 - 1205.92)
Czech Republic	173.66 (107.9 - 239.32)	103.21 (67.43 - 141.45)	78.64 (47.99 - 110.46)	51.9 (33.1 - 72.73)	4168.92 (2669.83 - 5566.56)	2359.4 (1623.55 - 3111.49)	2032.67 (1297.66 - 2758.4)	1263.96 (848.66 - 1732.41)
Democratic Republic of the Congo	55.66 (22.01 - 110.12)	68.45 (29.65 - 126.84)	39.84 (13.86 - 84.04)	44.32 (15.87 - 91.93)	1590.25 (672.79 - 2995.69)	1789.5 (840.85 - 3210.61)	1185.67 (453.42 - 2414.03)	1222.77 (499.17 - 2396.83)
Denmark	80.35 (42.1 - 120.89)	42.08 (24.63 - 62.18)	46.21 (27.01 - 65.98)	27.96 (18.4 - 38.97)	1961.24 (1072.02 - 2839.59)	1066.76 (653.29 - 1530.51)	1279.12 (809.83 - 1764.61)	838.75 (590.29 - 1123.09)
Djibouti	37.11 (9.83 - 94)	36.73 (11.8 - 82.76)	102.03 (35.33 - 229.25)	99.06 (37.72 - 231.07)	1189.71 (362.13 - 2830.12)	1023.34 (377.97 - 2220.03)	2974.78 (1137.97 - 6633.14)	2633.35 (1192.19 - 5883.4)
Dominica	64.66 (33.29 - 102.28)	147.27 (106.09 - 188.92)	85.19 (49.63 - 129.42)	122.54 (90.37 - 159.86)	1985.93 (1101.21 - 2985.84)	3781.66 (2890.18 - 4672.42)	2715.88 (1723.57 - 3845.84)	3323.32 (2568.03 - 4108.44)
Dominican Republic	54.3 (28.55 - 83.33)	63.65 (42.06 - 87.97)	79.88 (47.23 - 114.06)	80.58 (58.16 - 104.81)	1620.38 (911.15 - 2371.62)	1892.89 (1329.09 - 2481.62)	2226.08 (1427.04 - 3019.58)	2176.39 (1656.64 - 2712.73)
Ecuador	75.59 (48.42 - 102.87)	68.47 (49.27 - 89.05)	91.7 (59.56 - 128.47)	84.15 (61.09 - 109.63)	2282.92 (1569.15 - 2992.46)	2209.18 (1689.12 - 2763.86)	2607.88 (1779.1 - 3465.2)	2472.1 (1907.52 - 3063.68)
Egypt	153.2 (98.23 - 207.29)	154.38 (116.89 - 192.18)	176.16 (123.4 - 227.98)	154.56 (121.52 - 188.62)	4317.33 (2880.28 - 5717.92)	4251.73 (3367.61 - 5152.98)	5025.64 (3688.34 - 6374.74)	4435.23 (3623.18 - 5284.87)
El Salvador	60.15 (31.1 - 92.84)	69.65 (45.38 - 95.83)	85.79 (52.56 - 121.49)	81.58 (57.55 - 107.54)	1825.31 (1024.68 - 2685.67)	2126.33 (1470.05 - 2813.83)	2639.15 (1718.54 - 3589.48)	2484.47 (1872.69 - 3120.57)
Equatorial Guinea	70.61 (13.3 - 175.15)	105.04 (28.31 - 240.45)	98.44 (36.68 - 277.15)	139.32 (48.2 - 374.16)	2151.62 (459.97 - 5271.16)	2608.79 (751.06 - 6330.46)	2880.55 (1199.94 - 7845.58)	3688.22 (1489.58 - 9771.04)
Eritrea	60.28 (27.59 - 105.48)	51.92 (22.34 - 95.38)	31.31 (8.43 - 81.5)	43.71 (10.76 - 108.58)	1888.27 (918.47 - 3208.16)	1310.92 (565.27 - 2361.21)	1008.58 (312.13 - 2525.4)	1192.83 (348.25 - 2943.19)
Estonia	142.26 (82.59 - 208.03)	94.71 (60.56 - 131.64)	102.1 (65.74 - 140.51)	72.51 (49.4 - 95.75)	3619.28 (2190.04 - 5076.49)	2191.23 (1509.03 - 2897)	2504.56 (1710.49 - 3315.22)	1629.75 (1214.71 - 2052.52)
Ethiopia	32.14 (9.85 - 67.7)	33.25 (11.82 - 66.83)	39.56 (12.29 - 92.04)	50.27 (18.48 - 105.34)	968.39 (299.54 - 1997.48)	853.39 (296.97 - 1729.09)	1100.1 (368.26 - 2404.45)	1299.36 (536.63 - 2602.51)
Federated States of Micronesia	179.19 (88.14 - 290.86)	218 (123.84 - 345.82)	199.34 (107.77 - 330.51)	217.81 (121.01 - 376.27)	5722.66 (3023.21 - 9112.93)	6298.26 (3853.56 - 9566.02)	6344.42 (3605.59 - 10325.01)	6401.5 (3975.87 - 10380.28)
Fiji	256.52 (156.02 - 369.01)	291.01 (199.56 - 404.89)	324.21 (213.49 - 447.77)	342.14 (241.99 - 470.98)	7853.9 (4919.66 - 11155.84)	8385.54 (6071.33 - 11332.08)	9344.54 (6421.25 - 12651.23)	9549.66 (7117.91 - 12408.73)
Finland	111.25 (67.84 - 155.53)	57.61 (36.43 - 81.15)	54.6 (33.98 - 76.13)	34.05 (22.91 - 46.33)	2756.8 (1713.11 - 3757.17)	1384.67 (927.76 - 1865.6)	1475.16 (968.61 - 1992.66)	910.07 (658.21 - 1190.27)
France	52.45 (28.48 - 78.69)	25.94 (15.33 - 37.57)	39.7 (23.13 - 57.15)	20.67 (13.12 - 28.97)	1357.16 (763.85 - 1955.41)	641.82 (391.21 - 919.12)	1096.75 (686.19 - 1526.29)	624.65 (428.86 - 852.1)
Gabon	108.73 (52.93 - 183.09)	117.83 (69.16 - 181.02)	100.29 (46.07 - 197.57)	153.61 (77.26 - 273.91)	3135.09 (1610.44 - 5093.62)	2853.01 (1742.05 - 4221.12)	2920.64 (1474.07 - 5465.05)	3969.06 (2249.74 - 6880.36)
Georgia	122.87 (60.88 - 192.54)	100.54 (62.76 - 140.95)	152.87 (88.08 - 219.47)	88.94 (58.27 - 121.52)	3338.09 (1777.98 - 4962.61)	2423.94 (1601.46 - 3298.78)	4142.28 (2540.66 - 5674.95)	2229.83 (1537.43 - 2922.43)
Germany	106.87 (63.34 - 150.53)	63.4 (39.98 - 89.41)	58.67 (37.25 - 80.18)	39.41 (25.81 - 53.97)	2491.62 (1508.18 - 3402.88)	1376.59 (900.56 - 1879.48)	1503.82 (1012.11 - 2006.17)	977.88 (694.48 - 1286.69)
Ghana	15.93 (4.24 - 38.48)	52.46 (18.75 - 110.29)	70.9 (28.18 - 151.68)	102.26 (37.87 - 222.18)	486.06 (132.99 - 1117.07)	1495.42 (586.9 - 3023.98)	1999.61 (834.75 - 4163.91)	2875.94 (1239.4 - 6017.4)
Greece	81.64 (47.11 - 116.61)	63.28 (40.62 - 87.73)	62.77 (38.64 - 87.02)	42.9 (28.66 - 58.35)	2016.5 (1219.51 - 2764.67)	1447.19 (989.27 - 1930.38)	1706.91 (1115.44 - 2277.36)	1051.95 (747.06 - 1389.23)
Greenland	102.36 (55.69 - 153.08)	72.18 (42.18 - 105.62)	80.86 (49.97 - 113.22)	50.95 (34.24 - 69.59)	2870.49 (1643.63 - 4136.73)	2028.51 (1265.69 - 2838.92)	2173.45 (1396.38 - 2919.9)	1496.47 (1076.48 - 1975.48)
Grenada	96.37 (50.69 - 145.87)	153.42 (112.36 - 197.36)	108.6 (64.51 - 154.93)	161.42 (124.51 - 202.48)	2748.26 (1591.86 - 4013.57)	4140.03 (3211.52 - 5145.22)	3185.76 (2033.34 - 4412.79)	4287.52 (3447.97 - 5181.72)
Guam	148.76 (98.8 - 205.82)	135.52 (98.59 - 176.95)	182.76 (121.62 - 250.29)	145.42 (100.63 - 195.09)	4258.35 (3046.97 - 5589.59)	3818.27 (2997.69 - 4674.07)	5393.04 (3796.38 - 7169.22)	4234.78 (3167.68 - 5350.58)
Guatemala	32.25 (13.84 - 54)	51.13 (31.02 - 73.27)	56.7 (30.38 - 85.9)	78.83 (50.11 - 113.63)	1123.16 (554.39 - 1760.54)	1652.95 (1064.43 - 2282.23)	2106.99 (1266.07 - 2970.31)	2620.24 (1830.98 - 3534.43)
Guinea	18.07 (5.8 - 37.41)	42.95 (20.61 - 71.81)	41.79 (14.51 - 95.13)	79.15 (31.1 - 153.23)	564.35 (189.83 - 1124.45)	1188.81 (587.52 - 1935.71)	1272.82 (461.84 - 2784.35)	2083.01 (896.26 - 3912.6)
Guinea-Bissau	21.94 (3.69 - 71.42)	39.44 (7.5 - 117.92)	57.37 (14.86 - 172.02)	113.3 (25.78 - 321.22)	697.13 (134.05 - 2190.42)	1184.66 (266.26 - 3346.78)	1766.88 (504.32 - 5221.28)	3219.39 (861.98 - 9396.39)
Guyana	144.51 (76.64 - 221.43)	206.88 (148.77 - 265.77)	136.96 (77.54 - 204.09)	199.69 (147.49 - 260.82)	4087.21 (2265.4 - 6058.52)	5498.79 (4098.66 - 6832.78)	3859.55 (2319.28 - 5579.41)	5281.51 (4104.85 - 6673.32)
Haiti	55.92 (22.11 - 101.96)	133.54 (75.64 - 203.99)	68.85 (31.13 - 121)	130.91 (74.6 - 205.77)	1643.7 (696.01 - 2886.14)	3689.8 (2153.79 - 5513.05)	2017.35 (963.78 - 3388.8)	3669.87 (2207.24 - 5647.59)
Honduras	48.68 (22.86 - 79.23)	69.56 (41.92 - 100.08)	80.92 (42.45 - 137.16)	113.6 (69.89 - 163.64)	1576.19 (790.47 - 2429.87)	2141.56 (1371.49 - 2928.42)	2304.8 (1325.83 - 3643.4)	3045.78 (2000.76 - 4212.52)
Hungary	189.34 (124.94 - 249.59)	125.21 (89.45 - 160.77)	98.57 (62.5 - 135.81)	61.85 (40.86 - 84.35)	4992.96 (3449.34 - 6420.61)	3055.28 (2287.77 - 3806)	2682.21 (1776.03 - 3598.38)	1681.19 (1195.32 - 2205.78)
Iceland	78.97 (48.16 - 110.33)	49.13 (33.43 - 67.22)	46.47 (29.12 - 64.41)	22.55 (14.9 - 31.12)	1945.74 (1263.42 - 2601.01)	1214.54 (873.53 - 1571.91)	1256.48 (833.66 - 1659.6)	733.88 (522.76 - 979.62)
India	36.88 (15.71 - 64.97)	42.81 (21.68 - 69.31)	54.07 (26.2 - 88.03)	43.29 (23.71 - 67.04)	1080.45 (470.1 - 1869.68)	1177.61 (609.39 - 1880.97)	1603.01 (815.43 - 2555.55)	1213.51 (696.79 - 1816.26)
Indonesia	22.64 (7.22 - 45.97)	42.06 (19.29 - 71.42)	55.99 (25.66 - 98.23)	74.43 (42.4 - 114.99)	766.38 (257.28 - 1489.86)	1385.15 (673.34 - 2261.68)	1965.58 (999.23 - 3247.65)	2479.05 (1524.74 - 3673.47)
Iran	90.65 (44.82 - 146.47)	98.91 (63.33 - 138.24)	102.34 (56.93 - 162.04)	94.37 (59.81 - 137.6)	2509.36 (1318.44 - 3910.86)	2733.14 (1877.62 - 3632.21)	2846.58 (1712.44 - 4306.27)	2696.7 (1880.04 - 3776.55)
Iraq	234.36 (158.28 - 335.3)	243.66 (171.04 - 324.3)	217.44 (129.14 - 321.12)	206.31 (136.01 - 295.65)	6502.45 (4537.22 - 8970.97)	6404.78 (4660.04 - 8287.78)	6320.96 (4108.69 - 9096.41)	5726.87 (4046.82 - 7892.32)
Ireland	78.95 (44.1 - 118.31)	45.43 (27.36 - 65.43)	53.52 (34.39 - 74.23)	26.08 (16.62 - 37.12)	1947.56 (1137.71 - 2790.98)	1141.6 (729.68 - 1583.93)	1390.15 (956.42 - 1844.05)	760.76 (532.91 - 1026.46)
Israel	87.71 (52.09 - 123.95)	82.56 (57.19 - 107.01)	57.11 (36.35 - 78.59)	41.12 (28.52 - 54.19)	2154.48 (1327.01 - 2966.16)	1880.82 (1365.65 - 2412.64)	1470.18 (978.76 - 1961.47)	1093.15 (817.99 - 1411.84)
Italy	70.09 (37.72 - 103.91)	45.78 (27.83 - 65.07)	45.17 (25.2 - 65.96)	29.71 (17.99 - 42.27)	1704.79 (958.11 - 2435.88)	1059.64 (679.19 - 1470.44)	1122.19 (660.37 - 1596.6)	717.81 (464.13 - 1007.66)
Jamaica	61.17 (31.6 - 96.5)	109.22 (74.03 - 146.48)	79.54 (47.68 - 116.22)	120.56 (85.95 - 158.06)	1740.2 (969.89 - 2596.6)	3063.88 (2231.86 - 3943.18)	2345.32 (1520.86 - 3265.9)	3334.87 (2592.91 - 4139.29)
Japan	23.83 (8.11 - 44.74)	18.07 (7.48 - 31.32)	15.64 (5.62 - 28.85)	9.87 (4.22 - 17.05)	720.53 (263 - 1308.63)	509.14 (220.08 - 875.18)	526.84 (203.33 - 937.49)	321.99 (143.34 - 558.01)
Jordan	144.76 (93.66 - 209.64)	198.5 (146.55 - 258.18)	118.79 (80.11 - 159.98)	107.32 (80.38 - 136.14)	4299.24 (2938.7 - 5943.18)	5362.91 (4165.97 - 6772.99)	3633.73 (2593.68 - 4668.33)	3176.42 (2469.61 - 3918.3)

Kazakhstan	139.79<br>(80.95 - 200.99)	129.61<br>(90.34 - 170.01)	161.11<br>(98.9 - 222.35)	127.32<br>(90.8 - 168.11)	3532.75<br>(2042.32 - 5027.14)	3028.5<br>(2211.63 - 3831.81)	4173<br>(2598.28 - 5691.67)	3069.41<br>(2292.48 - 3913.11)
Kenya	20.21<br>(8.47 - 37.83)	37.15<br>(19.64 - 60)	35.74<br>(18.69 - 58.6)	51.58<br>(32.74 - 74.7)	681.79<br>(308.69 - 1202.54)	1042.78<br>(582.56 - 1603.22)	1125.04<br>(611.97 - 1776.45)	1493.03<br>(1009.37 - 2060.93)
Kiribati	310.73<br>(221.24 - 403.87)	282.39<br>(215.32 - 357.21)	374.79<br>(262.44 - 504.61)	255.54<br>(186.24 - 335.26)	9694.59<br>(7227.64 - 12341.19)	8250.02<br>(6532.28 - 10180.23)	11692.15<br>(8466.92 - 15510.3)	7438.98<br>(5723.86 - 9502.68)
Kuwait	92.16<br>(64.49 - 119.95)	115.07<br>(89.13 - 142.14)	93.92<br>(65.23 - 126.12)	93.62<br>(70.55 - 120.66)	2860.98<br>(2115.13 - 3562.76)	3229.06<br>(2627.21 - 3868.53)	3026.52<br>(2239 - 3850.22)	2715.3<br>(2167.35 - 3389.32)
Kyrgyzstan	117.63<br>(62.71 - 180.12)	116.24<br>(76.96 - 158.39)	141.52<br>(81.43 - 205.88)	119.01<br>(79.83 - 159.89)	3107.68<br>(1754.55 - 4520.7)	2865.07<br>(2007.27 - 3749.96)	3772.79<br>(2277 - 5302.64)	2901.38<br>(2069.46 - 3752.34)
Laos	31.64<br>(8.5 - 72.24)	58.68<br>(22.12 - 117.79)	46.12<br>(17.17 - 86.79)	59.17<br>(27.23 - 104.16)	1005.78<br>(287.27 - 2209.89)	1712.9<br>(668.82 - 3330.3)	1494.46<br>(606.01 - 2673.8)	1839.46<br>(930.97 - 3011.33)
Latvia	161.65<br>(95.2 - 230.05)	117.38<br>(80.17 - 155.53)	123.46<br>(79.11 - 167.73)	82.54<br>(57.88 - 107.7)	4064.07<br>(2495.09 - 5596.25)	2672.42<br>(1942.43 - 3416.35)	3187.14<br>(2134.66 - 4208.53)	2009.72<br>(1506.48 - 2526.88)
Lebanon	127.05<br>(70.44 - 198.48)	122.63<br>(76.94 - 179.04)	69.44<br>(35.27 - 113.1)	75.21<br>(44.75 - 109.7)	3664.48<br>(2191.82 - 5471.83)	3454.11<br>(2306.49 - 4827.33)	2251.49<br>(1351.61 - 3307.16)	2479.97<br>(1658.62 - 3401.02)
Lesotho	50.14<br>(18.72 - 96.02)	112.14<br>(64.94 - 176.69)	144.66<br>(50.77 - 290.28)	248.06<br>(112.24 - 492.01)	1469.42<br>(583.11 - 2734.7)	2951.34<br>(1848.06 - 4392.29)	4381.54<br>(1629.03 - 8589.17)	6521.84<br>(3271.9 - 13015.85)
Liberia	65.2<br>(31.06 - 113.45)	101.11<br>(55.69 - 166.62)	65.9<br>(30.16 - 125.8)	116.73<br>(53.27 - 202.76)	1957.56<br>(1009.77 - 3274.97)	2621.25<br>(1508.18 - 4171.01)	1987.42<br>(1012.48 - 3610)	3058.01<br>(1547.35 - 5044.34)
Libya	81.82<br>(49.93 - 119.9)	103.47<br>(72.05 - 137.55)	96.82<br>(59.59 - 141.38)	109.74<br>(78.24 - 148.04)	2379.37<br>(1551.14 - 3309.51)	3037.53<br>(2274.4 - 3882.65)	2904.28<br>(1926.13 - 3953.48)	3285.73<br>(2464.74 - 4171.65)
Lithuania	127.93<br>(75.43 - 182.99)	95.26<br>(63.81 - 128.71)	115.01<br>(72.37 - 157.2)	68.25<br>(48.52 - 89.12)	3347.71<br>(2047 - 4626.57)	2196.22<br>(1565.19 - 2852.87)	2979.35<br>(1938.84 - 3993.89)	1690.43<br>(1249.6 - 2156.43)
Luxembourg	89.49<br>(53.56 - 126.18)	56.22<br>(37.41 - 76.05)	43<br>(28.21 - 58.41)	28.34<br>(19.45 - 37.96)	2195.13<br>(1356.56 - 3025.54)	1417.75<br>(994.88 - 1870.89)	1282.1<br>(874.31 - 1700.92)	874.48<br>(623.11 - 1162.07)
Macedonia	117.33<br>(69.58 - 166.68)	128.51<br>(90.22 - 167.84)	108.05<br>(67.72 - 150.65)	98.1<br>(69.95 - 127.64)	3027.01<br>(1906.35 - 4201.44)	2975.18<br>(2137.28 - 3789.24)	2885.24<br>(1918.94 - 3876.37)	2430.05<br>(1797.18 - 3095.54)
Madagascar	53.19<br>(23.19 - 92.53)	54.28<br>(22.76 - 98.54)	44.08<br>(12.94 - 107.34)	56.08<br>(19.49 - 124.04)	1714.16<br>(774.53 - 2897.74)	1378.82<br>(599.41 - 2463.01)	1336.8<br>(404.21 - 3209.41)	1402.81<br>(507.58 - 2974.95)
Malawi	21.72<br>(6.69 - 47.63)	30.85<br>(10.42 - 63.14)	48.36<br>(16.89 - 105.87)	45.69<br>(17.2 - 95.21)	717.1<br>(242.44 - 1492.73)	846.79<br>(297.31 - 1682.98)	1298.07<br>(499.39 - 2755.89)	1264.21<br>(539.53 - 2423.32)
Malaysia	67.74<br>(34.8 - 105.17)	63.94<br>(35.86 - 94.81)	75.58<br>(42.7 - 114.07)	54.32<br>(33.42 - 79.24)	1958.5<br>(1056.22 - 2923.89)	1859.06<br>(1113.62 - 2674.9)	2313.08<br>(1374.79 - 3332.28)	1730.12<br>(1127.18 - 2408.34)
Maldives	32.05<br>(12.29 - 58.45)	66.95<br>(33.92 - 109.34)	42.53<br>(22.4 - 67.33)	38.68<br>(23.55 - 57.28)	960.26<br>(398.87 - 1688.25)	1885.77<br>(1007.23 - 2909.31)	1253.54<br>(720.79 - 1851.67)	1164.93<br>(790.65 - 1603.9)
Mali	20.83<br>(6.62 - 42.57)	43.58<br>(17.93 - 82.81)	51.57<br>(23.41 - 102.07)	116.76<br>(52.91 - 217.25)	647.59<br>(210.61 - 1291.5)	1168.65<br>(499.16 - 2177.12)	1534.03<br>(747.15 - 2864.98)	3034.3<br>(1485.17 - 5397.15)
Malta	97.03<br>(59.91 - 135.99)	71.37<br>(49.32 - 93.74)	57.89<br>(37.31 - 79.3)	39.14<br>(27.27 - 52.84)	2432.21<br>(1584.2 - 3283.97)	1817.64<br>(1310.08 - 2314.82)	1629.32<br>(1119.78 - 2151.41)	1126.04<br>(825.17 - 1459.26)
Marshall Islands	237.27<br>(161.39 - 320.22)	244.33<br>(183.01 - 309.97)	282.16<br>(192.94 - 386.96)	301.65<br>(220.57 - 403.57)	7584.3<br>(5375.62 - 9968.88)	7283.93<br>(5708.17 - 8923.12)	9109.48<br>(6533.19 - 12102.08)	8925.01<br>(6792.38 - 11427.77)
Mauritania	40.08<br>(15.86 - 71.92)	86.44<br>(50.19 - 130.57)	52.43<br>(19.11 - 104.76)	107.04<br>(47.95 - 195.6)	1224.86<br>(514.05 - 2140.5)	2435.26<br>(1545.45 - 3525.39)	1548.35<br>(697.19 - 2988.47)	2815.71<br>(1429.16 - 4942.57)
Mauritius	118.03<br>(54.92 - 193.57)	104.79<br>(62.3 - 151.74)	139.25<br>(80.85 - 202.18)	131.07<br>(88.85 - 175.79)	3334.17<br>(1620.48 - 5260.85)	2812.62<br>(1781.04 - 3869.78)	3989.98<br>(2460.64 - 5555.45)	3516.58<br>(2558.62 - 4503.27)
Mexico	87.74<br>(58.03 - 117.41)	91.26<br>(67.39 - 115.57)	103.54<br>(69.87 - 137.88)	105.02<br>(78.17 - 131.97)	2837.63<br>(1961.48 - 3693.97)	2875.83<br>(2223.25 - 3550.26)	3214.9<br>(2299.11 - 4130.54)	3003.63<br>(2318.1 - 3706.49)
Moldova	149.01<br>(94.95 - 205.24)	153.53<br>(109.13 - 198.62)	149.74<br>(102.39 - 197.85)	117.48<br>(86.57 - 150.69)	3627.68<br>(2365.38 - 4900.33)	3504.91<br>(2643.4 - 4345.08)	3654.53<br>(2626.04 - 4697.89)	2657.2<br>(2070.88 - 3268.1)
Mongolia	130.39<br>(71.56 - 196.03)	128.69<br>(81.61 - 181.1)	192.25<br>(113.35 - 277.52)	129.45<br>(83.53 - 178.24)	3475.6<br>(1988.97 - 5060.98)	3356.38<br>(2241.07 - 4530.1)	5357.29<br>(3326.43 - 7440.52)	3484.12<br>(2393.22 - 4651.26)
Montenegro	111.24<br>(68.85 - 157.38)	94.94<br>(63.83 - 129.8)	101.27<br>(62.66 - 142.82)	74.89<br>(51.83 - 100.87)	3025.56<br>(1939.6 - 4112.82)	2473.77<br>(1761.13 - 3213.7)	2751.89<br>(1793.74 - 3756.87)	2029.06<br>(1460.08 - 2628.93)
Morocco	85.04<br>(45.38 - 131.95)	103.3<br>(65.82 - 148.45)	77.39<br>(40.81 - 129.93)	102.62<br>(57.32 - 158.57)	2490.75<br>(1420.56 - 3677.57)	2952.07<br>(1987.75 - 4068)	2336.94<br>(1332.67 - 3654.03)	3032.87<br>(1949.93 - 4325.84)
Mozambique	32.15<br>(10.4 - 64.37)	45.35<br>(20.54 - 79.06)	74.43<br>(22.89 - 170.02)	88.21<br>(31.36 - 208.56)	964.47<br>(339.63 - 1860.1)	1133.36<br>(542.92 - 1887.96)	2229.34<br>(728.99 - 4894.81)	2246.96<br>(940.83 - 5083.49)
Myanmar	26.19<br>(6.71 - 63.25)	44.46<br>(15.53 - 92.41)	64.64<br>(27.92 - 115.37)	63.29<br>(30.27 - 111)	829.89<br>(228.71 - 1902.32)	1339.58<br>(521.56 - 2666.92)	2042.84<br>(955.75 - 3507.83)	1941.32<br>(1048.53 - 3193.51)
Namibia	58.53<br>(26.37 - 101.72)	82.15<br>(46.52 - 126.16)	66.26<br>(25.48 - 135.77)	76.16<br>(37.76 - 159.14)	1727.97<br>(815.53 - 2860.32)	2231.44<br>(1361.32 - 3279.71)	2101.91<br>(935.94 - 4050.43)	2288.85<br>(1320.28 - 4255.08)
Nepal	15.27<br>(4.26 - 34.53)	13.29<br>(4.08 - 29.85)	44.62<br>(19.06 - 80.98)	40.97<br>(18.17 - 72.57)	482.36<br>(141 - 1072.27)	356.33<br>(110.61 - 789.36)	1406.79<br>(647.16 - 2408.11)	1208.44<br>(599.88 - 2009.6)
Netherlands	63.16<br>(30.95 - 97.5)	44.04<br>(27.22 - 61.78)	39.55<br>(22.85 - 56.85)	25.96<br>(16.77 - 36.1)	1605.85<br>(817.76 - 2399.52)	1106.49<br>(711.07 - 1546.11)	1164.69<br>(721.15 - 1609.14)	842.99<br>(582.71 - 1137.95)
New Zealand	84.67<br>(49.05 - 122.06)	53.95<br>(34.53 - 74.62)	51.32<br>(33.5 - 70.2)	35.92<br>(24.27 - 48.65)	2191.01<br>(1341.35 - 3040.45)	1426.67<br>(982.33 - 1898.33)	1526.83<br>(1069.78 - 2012.09)	1149.77<br>(838.06 - 1489.49)
Nicaragua	43.08<br>(21.24 - 68.22)	53.05<br>(34.39 - 73.37)	83.07<br>(50.4 - 119.18)	77.97<br>(53.9 - 103.6)	1319.09<br>(713.1 - 1987.82)	1726.12<br>(1205.96 - 2296.69)	2430.63<br>(1557.6 - 3350.45)	2242.76<br>(1642.73 - 2846.2)
Niger	21.97<br>(7.26 - 46.79)	32.02<br>(14.2 - 59.01)	38.66<br>(13.58 - 82.49)	53.6<br>(20.28 - 110.29)	683.18<br>(239.49 - 1404.6)	880.74<br>(408.15 - 1553.39)	1184.14<br>(465.75 - 2437.48)	1443.28<br>(612.16 - 2827.31)
Nigeria	21.83<br>(5.47 - 53.9)	39.16<br>(14.2 - 87.41)	39.15<br>(18.42 - 78.91)	50.33<br>(25.01 - 110.12)	678.4<br>(189.05 - 1564.98)	1095.82<br>(431.55 - 2293.83)	1203.32<br>(602.93 - 2231.26)	1391.02<br>(771.71 - 2839.36)
North Korea	24.09<br>(5.89 - 57.28)	25.69<br>(7.84 - 57.31)	28.06<br>(7.29 - 63.46)	24.11<br>(7.88 - 49.5)	693.07<br>(172.17 - 1593.77)	739.16<br>(239.27 - 1553.19)	820.45<br>(226.6 - 1833.16)	697.33<br>(238.31 - 1376.74)
Northern Mariana Islands	95.28<br>(59.15 - 137.7)	131.27<br>(91.23 - 177.22)	102.25<br>(64.84 - 143.87)	99.24<br>(68.62 - 133.41)	3069.37<br>(2079.4 - 4173.73)	3974.39<br>(2941.67 - 5162.31)	3279.81<br>(2270.26 - 4357.73)	3123.31<br>(2353.25 - 3934.36)
Norway	69.36<br>(34.8 - 107.47)	34.61<br>(19.06 - 52.62)	36.66<br>(20.73 - 53.55)	21.82<br>(12.65 - 32.07)	1722.15<br>(911.24 - 2558.19)	881.35<br>(524.39 - 1282.02)	1064.29<br>(657.68 - 1483.58)	677.21<br>(445.25 - 957.36)
Oman	81.98<br>(46.3 - 130.96)	89.38<br>(51.93 - 140.87)	92.32<br>(56.93 - 131.3)	93.37<br>(62.9 - 123.7)	2471.6<br>(1505.95 - 3723.06)	2805.27<br>(1835.81 - 4077.19)	2927.13<br>(1965.5 - 3972.02)	3097.22<br>(2308.67 - 3934.54)
Pakistan	54.27<br>(21.95 - 98.36)	67.13<br>(33.15 - 113.44)	105.91<br>(54.68 - 169.46)	110.61<br>(64.32 - 166.31)	1543.67<br>(657.23 - 2752.17)	1869.41<br>(995.83 - 3001.1)	3009.85<br>(1619.45 - 4710.84)	3083.68<br>(1950.6 - 4398.84)
Palestine	114.35<br>(63.26 - 182.14)	127.99<br>(77.62 - 197.57)	149.74<br>(86.79 - 224.96)	118.18<br>(77.68 - 170.3)	3235.08<br>(1896.72 - 4980.17)	3467.53<br>(2290.59 - 5022.72)	4300.81<br>(2694.85 - 6204.24)	3206.53<br>(2240.24 - 4368.53)
Panama	25.76<br>(7.91 - 51.1)	31.93<br>(14.79 - 53.05)	41<br>(19.45 - 66.39)	44.99<br>(27.33 - 66.1)	732.58<br>(246.06 - 1390.32)	948.23<br>(488.1 - 1521.39)	1300.08<br>(695.74 - 1984.71)	1451.1<br>(974.43 - 2020.88)
Papua New Guinea	123.37<br>(53.16 - 215.73)	178.68<br>(92.51 - 285.77)	116.91<br>(48.57 - 218.45)	194.04<br>(93.88 - 334.01)	3988.13<br>(1823.29 - 6852.04)	5405.77<br>(2924.76 - 8577.4)	3784.7<br>(1651.05 - 6931.46)	5859.7<br>(3037.46 - 9686.84)

Paraguay	63.54 (34.77 - 96.48)	75.07 (45.79 - 106.49)	103.42 (65.21 - 150.61)	98.64 (64.25 - 136.03)	1823.48 (1040.49 - 2660.11)	2221.91 (1487.83 - 2985.19)	2955.81 (2011.57 - 4029.44)	2743.01 (1951.6 - 3588.21)
Peru	46.85 (23.87 - 72.8)	55.33 (34.99 - 77.37)	39.66 (24.39 - 58.19)	38.89 (25.26 - 54.17)	1347.86 (746.57 - 2003.47)	1568.4 (1055.78 - 2111.95)	1248.6 (817.1 - 1728.78)	1188.16 (850.98 - 1579.9)
Philippines	39.29 (15.72 - 70.01)	29.43 (13.64 - 48.6)	71.34 (33.39 - 116.73)	55.85 (30.33 - 85.68)	1313.19 (556.04 - 2243.42)	1011.3 (504.12 - 1624.57)	2280.94 (1135.61 - 3600.04)	1877.86 (1072.25 - 2765.8)
Poland	161.11 (98.98 - 222.03)	103.2 (68.39 - 138.85)	89.02 (57.62 - 122.14)	50.31 (34.08 - 68.06)	4211.88 (2724.49 - 5624.54)	2441.2 (1709.73 - 3162.83)	2441.4 (1631.49 - 3231.8)	1315.98 (922.77 - 1736.88)
Portugal	85.37 (47.02 - 125.79)	64.35 (41.6 - 88.66)	58.77 (38.12 - 79.59)	38.16 (26.75 - 49.63)	2160.98 (1244.43 - 3053.03)	1583.01 (1072.97 - 2121.41)	1528.95 (1016.13 - 2041.98)	1047.1 (763.39 - 1374.91)
Puerto Rico	99.09 (62.69 - 136.1)	88.03 (62.39 - 113.97)	103.67 (73.16 - 135.89)	84.4 (65.11 - 106.54)	2878.51 (1925.05 - 3811.16)	2445.48 (1856.56 - 3072.87)	3032.94 (2255.64 - 3828.18)	2371.82 (1897.4 - 2886.87)
Qatar	142.66 (101.74 - 187.76)	209.52 (161.33 - 263.62)	106.55 (69.25 - 154.4)	126.08 (88.18 - 169.05)	4074.55 (3099.85 - 5097.34)	5537.99 (4495.61 - 6656.91)	3236.71 (2297.72 - 4272.38)	3669.83 (2825.13 - 4589.9)
Romania	136.33 (82.46 - 192.54)	109.22 (70.41 - 148.62)	129.81 (85.25 - 178.54)	89.81 (60.73 - 118.77)	3392.47 (2131.31 - 4655.69)	2484.74 (1669.26 - 3285.76)	3228.95 (2198.51 - 4266.93)	1978.87 (1402.19 - 2542.71)
Russia	139.93 (80.48 - 204.05)	114.17 (80.11 - 149.82)	158.89 (101.21 - 217.74)	109.65 (81.2 - 140.42)	3487.66 (2042.64 - 4951.58)	2693.65 (2003.01 - 3411.37)	4088.1 (2678.5 - 5446.48)	2658.37 (2074.91 - 3282.38)
Rwanda	23.84 (5.53 - 57.27)	68.56 (25.73 - 130.94)	25.95 (6.28 - 67.59)	51.46 (18.24 - 114.56)	716.93 (179.72 - 1622.92)	1808.86 (725.08 - 3320.66)	768.66 (212.73 - 1913.88)	1367.71 (554.23 - 2810.1)
Saint Lucia	85.95 (45.55 - 132.95)	162.77 (120.95 - 204.94)	71.18 (40.25 - 106.26)	125.52 (92.66 - 161.58)	2455.66 (1393.76 - 3628.62)	4210.54 (3308.09 - 5122.23)	2195.61 (1365.77 - 3090.53)	3242.66 (2561.64 - 4018.28)
Saint Vincent and the Grenadines	101.35 (59.23 - 148.7)	158.34 (111.15 - 205.45)	152.44 (102.89 - 202.83)	183.56 (141.81 - 225.18)	2939.12 (1854.75 - 4072.5)	4253.15 (3190.69 - 5308.44)	4247.61 (3067.73 - 5482.49)	4731.67 (3847.18 - 5645.27)
Samoa	235.52 (158.21 - 326.5)	241.41 (174.27 - 326.38)	190.97 (131.86 - 259.63)	259.02 (186.47 - 348.77)	6950.64 (4839.33 - 9426.72)	6383.24 (4760.54 - 8383.72)	5436.76 (3922.31 - 7214.35)	6751.43 (5040.17 - 8743.92)
Sao Tome and Principe	36.8 (16.55 - 63.16)	58.11 (32.25 - 91)	60.08 (21.62 - 120.2)	103.16 (42.25 - 200.14)	1125.13 (538.93 - 1862.18)	1653.53 (975.35 - 2502.74)	1827.27 (722.57 - 3545.97)	2855.73 (1300.41 - 5491)
Saudi Arabia	75.17 (46.68 - 107.11)	81.94 (59.04 - 108.39)	84.52 (57.94 - 114.53)	68.3 (51.5 - 87.36)	2296.27 (1512.61 - 3138.79)	2558.78 (1946.15 - 3250.16)	2507.02 (1808.44 - 3261.9)	2165.44 (1693.72 - 2699.84)
Senegal	39.27 (17.06 - 68)	45.2 (23.52 - 77.71)	53.1 (17.79 - 122.56)	82.52 (28.04 - 176.53)	1110.89 (493.84 - 1921.41)	1294.69 (730.27 - 2147.98)	1508.76 (528.72 - 3454.63)	2281.83 (905.98 - 4666.99)
Serbia	133.37 (83.12 - 185.39)	117.83 (79.62 - 157.03)	111.82 (74.88 - 148.41)	91.27 (67.6 - 116.58)	3377.75 (2199.15 - 4583.02)	2855.81 (2045.27 - 3688.81)	2974.58 (2078.07 - 3859.68)	2300.85 (1772.37 - 2891.02)
Seychelles	87.8 (41.42 - 144.5)	127.86 (85.68 - 171.72)	91.19 (53.72 - 134.99)	117.3 (80.03 - 156.29)	2596.57 (1292.54 - 4119.88)	3248.34 (2383.9 - 4105.58)	2761.83 (1775.77 - 3856.73)	3044.79 (2257.81 - 3884.06)
Sierra Leone	23.91 (7.64 - 56.3)	35.09 (14.43 - 67.62)	49.04 (18.61 - 98.1)	104.77 (49.57 - 197.13)	756.55 (257.39 - 1671.38)	1016.8 (470.47 - 1897.32)	1540.04 (659.68 - 2965.6)	2827.4 (1447.3 - 5139.76)
Singapore	51.64 (23.43 - 84.7)	45.68 (23.68 - 70.34)	29.62 (14.37 - 47.63)	22.34 (11.93 - 35.03)	1451.21 (690.65 - 2317.24)	1243.62 (670.57 - 1879.89)	841.4 (443.22 - 1295.46)	622.02 (363.75 - 939.98)
Slovakia	167.39 (104.34 - 231.44)	117.03 (79.61 - 156.26)	93.53 (56.65 - 131.69)	63.65 (42.02 - 87.15)	4319.9 (2792.28 - 5780.33)	2678.96 (1920.9 - 3424.98)	2335.99 (1458.34 - 3197.5)	1504.57 (1050.58 - 1985.44)
Slovenia	94.46 (56.36 - 134.03)	63.13 (40.82 - 86.56)	51.38 (32.09 - 71.91)	31.87 (20.38 - 44.32)	2407.43 (1462.98 - 3332.05)	1530.55 (1031.77 - 2048.02)	1434.58 (923.28 - 1948.47)	920.06 (631.55 - 1241.86)
Solomon Islands	196.98 (98.75 - 323.31)	285.71 (156.26 - 445.35)	256.72 (144.8 - 414.13)	341.01 (196.52 - 532.37)	6333.47 (3392.78 - 10186.88)	8428.89 (5000.11 - 12657.53)	8051.09 (4640.01 - 12766.89)	9773.05 (6111.81 - 14909.51)
Somalia	45.32 (8.43 - 127.53)	107.76 (24.77 - 250.99)	43.5 (9.52 - 112.72)	101.03 (24.23 - 242.51)	1463.89 (330.51 - 3897.13)	2980.19 (780.19 - 6721.4)	1325.42 (330.32 - 3416.05)	2828.67 (834.67 - 6943.8)
South Africa	110.55 (73.08 - 152.04)	205.85 (160.28 - 252.46)	135.09 (90.13 - 183.2)	186.16 (146.11 - 231.02)	3088.65 (2112.21 - 4136.69)	5037.13 (4061.73 - 6045.85)	3757.31 (2665.85 - 4950.67)	4812.55 (3917.61 - 5808.79)
South Korea	49.96 (15.29 - 95.94)	45.32 (19.95 - 75.97)	26.96 (10.69 - 47.39)	18.34 (8.67 - 30.06)	1399.25 (464.79 - 2581.85)	1171.66 (528.83 - 1918.16)	830.33 (349.72 - 1420.98)	540.1 (259.26 - 880.8)
South Sudan	41.7 (9.51 - 123.68)	58.54 (14.08 - 151.13)	47.7 (12.36 - 137.22)	89.69 (22.36 - 238.05)	1352.46 (365.67 - 3711.3)	1649.27 (475.3 - 4235.65)	1459.06 (437.09 - 4085.72)	2501.64 (749.1 - 6533.05)
Spain	67.94 (39.88 - 97.4)	50.01 (33.77 - 67.69)	40.84 (25.2 - 56.98)	27.21 (18.37 - 36.82)	1770.79 (1086.87 - 2429.8)	1218.22 (865.48 - 1601.53)	1131.95 (729.71 - 1540.77)	741.22 (528.71 - 982.48)
Sri Lanka	28.54 (10.57 - 53.03)	30.72 (15.31 - 49.68)	40.63 (16.7 - 75.84)	42.81 (21.22 - 70.25)	899.87 (356.26 - 1602.55)	922.21 (486.84 - 1431.88)	1261.88 (576.94 - 2218.22)	1284.77 (691.45 - 2000.53)
Sudan	96.53 (47.88 - 163.09)	158.56 (95.88 - 242.41)	100.99 (48.21 - 171.25)	146.71 (80.24 - 230.76)	2883.85 (1512.99 - 4693.92)	4378.64 (2790.24 - 6417.82)	3057.86 (1567.55 - 4893.47)	4128.4 (2480.3 - 6203.91)
Suriname	83.56 (44.94 - 126.79)	110.69 (74.06 - 148.58)	116.09 (71.83 - 166.68)	116.9 (87.06 - 153.5)	2467.07 (1430.41 - 3580.21)	3031.19 (2166.12 - 3879.73)	3368.69 (2249.24 - 4552.05)	3225.5 (2537.79 - 4051.33)
Swaziland	144.69 (74.39 - 235.52)	220.03 (131.41 - 327.83)	199.55 (62.39 - 396.87)	284.44 (113.65 - 592.42)	3830.02 (2076.43 - 6161.11)	5361.43 (3416.04 - 7879.92)	5764.37 (2006.9 - 11548.57)	7401.55 (3370.24 - 15052.87)
Sweden	67.99 (34.43 - 103.74)	39.78 (23.36 - 57.86)	43.94 (25.69 - 64.56)	30.07 (18.99 - 41.83)	1693.56 (925.33 - 2466.68)	1028.82 (646.08 - 1435.78)	1190.52 (750.69 - 1651.92)	875.29 (599.06 - 1181.3)
Switzerland	59.13 (32.05 - 88.33)	31.84 (18.66 - 46.84)	36.07 (20.54 - 52.88)	23.23 (14.14 - 33.23)	1414.46 (791.44 - 2055.89)	765.56 (477.65 - 1095.87)	924.62 (546.54 - 1321.55)	590.21 (391.49 - 829.81)
Syria	141.07 (88.35 - 207.47)	145.8 (99.71 - 204.99)	112.72 (67.28 - 164.16)	99.3 (67.92 - 130.98)	4198.09 (2692.56 - 5980.17)	4090.55 (2917.9 - 5508.97)	3305.76 (2135.67 - 4629.67)	2889.24 (2118.53 - 3677)
Taiwan	59.18 (24.86 - 99.68)	55.85 (28.09 - 87.88)	43.36 (20.18 - 73.11)	29.73 (14.73 - 48.14)	1669.69 (739.4 - 2753.02)	1526.82 (815.08 - 2343.02)	1449.56 (733.11 - 2300.93)	1066.72 (618.25 - 1611.54)
Tajikistan	61.24 (24.83 - 109.96)	79.26 (45.53 - 118.36)	57.46 (25.22 - 99.13)	84.01 (52.11 - 119.92)	1622.06 (696.91 - 2769.72)	2075.45 (1256.05 - 2996.78)	1608.88 (756.59 - 2646.59)	2169.57 (1417.23 - 2961.65)
Tanzania	66.12 (32.28 - 107.76)	72.44 (38.47 - 117.78)	37.07 (12.28 - 91.8)	66 (22.22 - 148.43)	1886.71 (966.33 - 2960.75)	1792.24 (1008.8 - 2791.33)	1077.62 (383.06 - 2550.24)	1764.64 (693.79 - 3774.98)
Thailand	31.17 (11.52 - 57.35)	31.61 (14.94 - 52.58)	50.58 (22.88 - 85.63)	41.06 (23.51 - 63.1)	1020.71 (407.54 - 1816.47)	1000.34 (513.19 - 1604.02)	1566.79 (787.95 - 2542.35)	1312.36 (813.96 - 1892.11)
The Bahamas	125.02 (72.36 - 180.93)	137.01 (93.73 - 181.01)	104.35 (66.58 - 147.22)	116.64 (83.88 - 153.85)	3539.96 (2188.87 - 4932.26)	3636.93 (2682.57 - 4604.93)	2996.23 (2033.37 - 4035.95)	3106.05 (2410.42 - 3899.33)
The Gambia	35.71 (12.23 - 80.97)	68.41 (22.3 - 150.94)	60.16 (26.42 - 118.56)	77.2 (34.56 - 139.77)	1113.03 (411.12 - 2481.8)	1884.78 (684.12 - 4025.34)	1806.17 (840.73 - 3441.36)	2047.43 (1017.64 - 3552.2)
Timor-Leste	12.65 (2.14 - 33.63)	23.97 (7.8 - 50.44)	15.61 (4.04 - 35.84)	22.58 (8.73 - 44.83)	385.3 (72.98 - 982.72)	734.31 (256.69 - 1481.67)	514.44 (151.17 - 1122.25)	737.08 (309.79 - 1365.68)
Togo	18.34 (5.39 - 40.44)	36.95 (16.14 - 67.72)	45.17 (13.29 - 96.85)	70.32 (29.51 - 139.13)	569.56 (175.7 - 1211.04)	988.35 (452.99 - 1765.68)	1372.25 (445.33 - 2829.33)	1915.24 (860.86 - 3634.45)
Tonga	162.54 (114.29 - 221.56)	229.08 (172.41 - 296.48)	200 (140.38 - 266.63)	179.66 (129.9 - 241.84)	4870.11 (3631.07 - 6396.49)	6481.26 (5098.47 - 8144.99)	5995.45 (4434.41 - 7878.71)	5232.74 (3968.96 - 6676.82)
Trinidad and Tobago	178.88 (113.42 - 243.74)	189.08 (141.48 - 235.82)	167.14 (110.73 - 228.69)	151.48 (115.52 - 191.62)	5087.2 (3428.3 - 6713.95)	5235.77 (4044.23 - 6376.46)	4808.41 (3345.37 - 6328.65)	4220.04 (3382.55 - 5127.74)

Tunisia	72.56 (37.89 - 111.69)	75.38 (49.39 - 105.75)	71.87 (37.24 - 118.82)	58.44 (34.71 - 87.31)	2065.44 (1134.89 - 3084.82)	2248.31 (1594.15 - 2947.42)	2165.43 (1242.28 - 3352.41)	1889.11 (1269.76 - 2570.83)
Turkey	125.95 (76.89 - 178.24)	112.91 (81.8 - 145.35)	74.13 (48.86 - 99.74)	61.36 (46.16 - 77.26)	3654.52 (2339.69 - 5014.29)	3200.24 (2456.58 - 4043.27)	2315.21 (1604.18 - 3030.03)	2002.61 (1534.25 - 2490.24)
Turkmenistan	154.57 (83.8 - 228.41)	144.53 (96.9 - 193.14)	184.06 (114.97 - 254.7)	138.55 (97.63 - 180.79)	4082.67 (2307.25 - 5828.74)	3639.93 (2540.07 - 4711.78)	4981.55 (3260.37 - 6600.6)	3610.82 (2705.92 - 4523.01)
Uganda	29.69 (9.23 - 63.03)	70.57 (30.84 - 123.92)	39.79 (11.43 - 98.43)	73.83 (25.34 - 155.26)	833.96 (264.44 - 1722.03)	1614.18 (729.09 - 2750.8)	1173.48 (366.49 - 2762.14)	1877.33 (722.4 - 3730.45)
Ukraine	146.84 (88.11 - 208.21)	118.28 (83.21 - 154.9)	154.54 (92.98 - 217.21)	111.15 (77.21 - 145.26)	3608.87 (2217.54 - 4976.42)	2736.72 (2032.41 - 3451.02)	3837.2 (2375.28 - 5252.31)	2601.6 (1931.09 - 3290.83)
United Arab Emirates	128.62 (76.26 - 188.48)	153.14 (100.08 - 214.72)	103.93 (60.16 - 156.17)	103.84 (68.82 - 148.44)	4028.43 (2596.37 - 5648.56)	4675.86 (3270.41 - 6389.66)	3425.1 (2209.42 - 4864.85)	3491.93 (2522.12 - 4661.11)
United Kingdom	97.16 (60.2 - 134.76)	56.51 (37.4 - 77.06)	51.32 (35.1 - 68.1)	30.13 (20.69 - 40.41)	2360.41 (1521.05 - 3195.28)	1356.02 (947.04 - 1795.73)	1390.91 (984.53 - 1801.34)	860.44 (623.82 - 1121.81)
United States	76.45 (43.94 - 111.21)	52.25 (35.29 - 70.93)	72.67 (52.01 - 93.87)	50.08 (38.07 - 62.65)	2217.56 (1356.7 - 3073.57)	1552.09 (1113.42 - 2032.37)	2329.97 (1728.55 - 2936.51)	1686.65 (1332 - 2064.87)
Uruguay	103.98 (61.94 - 148.71)	75.69 (51.32 - 102.11)	77.51 (49.59 - 106.38)	53.42 (37.65 - 69.72)	2579.09 (1610.07 - 3559.15)	1848.5 (1324.12 - 2412.8)	1968.1 (1321.96 - 2605.24)	1317.3 (983.35 - 1675.01)
Uzbekistan	177.7 (108.6 - 252.5)	138.52 (89.23 - 191.56)	163.25 (99.87 - 229.99)	142.89 (100.99 - 186.14)	4413.69 (2849.78 - 5990.48)	3219.68 (2173.71 - 4277.73)	4222.8 (2766.68 - 5646.49)	3494.54 (2659.82 - 4328.77)
Vanuatu	151.06 (73.69 - 258.79)	229.1 (133.79 - 368.2)	186.88 (97.99 - 307.89)	227.7 (126.8 - 378.11)	4749.71 (2409.1 - 7977.85)	6649.91 (4099.57 - 10432.15)	5906.64 (3296.18 - 9536.35)	6640.34 (3957.73 - 10690.74)
Venezuela	85.85 (45.96 - 129.27)	96.5 (64.25 - 130.22)	104.26 (63.41 - 147.23)	85.8 (58.46 - 117.79)	2370.9 (1370.69 - 3392.64)	2596.6 (1833.77 - 3339.64)	2881.75 (1911.17 - 3895.68)	2305.1 (1708.48 - 2973.35)
Vietnam	9.55 (1.88 - 23.94)	13.74 (4.03 - 29.93)	26.07 (8.52 - 53.67)	21.81 (7.69 - 43.1)	301.58 (61.54 - 734.94)	398.16 (121.95 - 832.71)	741.42 (237.94 - 1526.04)	592.88 (230.57 - 1141.89)
Virgin Islands, U.S.	109.55 (66.26 - 156.33)	109.62 (76.96 - 143.06)	133.67 (87.95 - 180.36)	108.24 (81.95 - 135.52)	2986.91 (1919.01 - 4081.66)	2836.12 (2121.6 - 3544.58)	3702.94 (2611.34 - 4819.18)	2822.63 (2296.04 - 3449.01)
Yemen	74.5 (27.15 - 149.71)	106.81 (42.95 - 205.83)	78.14 (31.08 - 158.74)	124.35 (59.22 - 225.41)	2246.97 (881.47 - 4390.23)	3017.44 (1304.31 - 5529.79)	2373.03 (1016.22 - 4579.63)	3639.91 (1893.85 - 6356.37)
Zambia	42.18 (18.29 - 74.55)	98.65 (51.59 - 153.77)	89.21 (36.94 - 159.17)	113.19 (49.16 - 215.87)	1264.35 (585.62 - 2193.06)	2285.79 (1210.99 - 3487.17)	2670.71 (1132.41 - 4687.58)	2871.02 (1353.13 - 5295.06)
Zimbabwe	33.14 (13.4 - 63.72)	73.42 (43.12 - 108.45)	62.6 (22.79 - 124.12)	89.81 (37.97 - 183)	1050.45 (484.92 - 1891.77)	2055.23 (1302.8 - 2859.84)	1921.89 (752.42 - 3634.3)	2633.36 (1348.49 - 4809.23)

Table S7. Comparison of the root-mean-square error (RMSE) from models using only measured data wth the models using both self-reported and measured data

	Measured only	Measured and self-report
**Overweight**	0.063	0.07
**Obesity**	0.033	0.036

Table S8. Sources of epidemiological evidence used for assessment of causality between high body mass index and disease endpoints.		
**Risk**	**Outcome**	**Citation/Note**
High body-mass index	Oesophageal cancer	Renehan AG, Tyson M, Egger M, Heller RF, Zwahlen M. Body-mass index and incidence of cancer: a systematic review and meta-analysis of prospective observational studies. Lancet 2008; 371: 569–78.
High body-mass index	Colon and rectum cancer	Karahalios A, English DR, Simpson JA. Weight change and risk of colorectal cancer: a systematic review and meta-analysis. Am J Epidemiol 2015; 181: 832–45.
High body-mass index	Colon and rectum cancer	Renehan AG, Tyson M, Egger M, Heller RF, Zwahlen M. Body-mass index and incidence of cancer: a systematic review and meta-analysis of prospective observational studies. Lancet 2008; 371: 569–78.
High body-mass index	Colon and rectum cancer	Schlesinger S, Lieb W, Koch M, et al. Body weight gain and risk of colorectal cancer: a systematic review and meta-analysis of observational studies. Obes Rev 2015; 16: 607–19.
High body-mass index	Liver cancer	Chen Y, Wang X, Wang J, Yan Z, Luo J. Excess body weight and the risk of primary liver cancer: an updated meta-analysis of prospective studies. Eur J Cancer 2012; 48: 2137–45.
High body-mass index	Liver cancer	Renehan AG, Tyson M, Egger M, Heller RF, Zwahlen M. Body-mass index and incidence of cancer: a systematic review and meta-analysis of prospective observational studies. Lancet 2008; 371: 569–78.
High body-mass index	Liver cancer	Rui R, Lou J, Zou L, et al. Excess body mass index and risk of liver cancer: a nonlinear dose-response meta-analysis of prospective studies. PLoS ONE 2012; 7: e44522.
High body-mass index	Liver cancer	Tanaka K, Tsuji I, Tamakoshi A, et al. Obesity and liver cancer risk: an evaluation based on a systematic review of epidemiologic evidence among the Japanese population. Jpn J Clin Oncol 2012; 42: 212–21.
High body-mass index	Liver cancer	Wang Y, Wang B, Shen F, Fan J, Cao H. Body mass index and risk of primary liver cancer: a meta-analysis of prospective studies. Oncologist 2012; 17: 1461–8.
High body-mass index	Gallbladder and biliary tract cancer	Park M, Song DY, Je Y, Lee JE. Body mass index and biliary tract disease: a systematic review and meta-analysis of prospective studies. Prev Med 2014; 65: 13–22.
High body-mass index	Gallbladder and biliary tract cancer	Renehan AG, Tyson M, Egger M, Heller RF, Zwahlen M. Body-mass index and incidence of cancer: a systematic review and meta-analysis of prospective observational studies. Lancet 2008; 371: 569–78.
High body-mass index	Pancreatic cancer	Alsamarrai A, Das SLM, Windsor JA, Petrov MS. Factors that affect risk for pancreatic disease in the general population: a systematic review and meta-analysis of prospective cohort studies. Clin Gastroenterol Hepatol 2014; 12: 1635–1644.e5; quiz e103.
High body-mass index	Pancreatic cancer	Renehan AG, Tyson M, Egger M, Heller RF, Zwahlen M. Body-mass index and incidence of cancer: a systematic review and meta-analysis of prospective observational studies. Lancet 2008; 371: 569–78.
High body-mass index	Breast cancer (Pre-menopause)	Renehan AG, Tyson M, Egger M, Heller RF, Zwahlen M. Body-mass index and incidence of cancer: a systematic review and meta-analysis of prospective observational studies. Lancet 2008; 371: 569–78.
High body-mass index	Breast cancer (Pre-menopause)	Xia X, Chen W, Li J, et al. Body mass index and risk of breast cancer: a nonlinear dose-response meta-analysis of prospective studies. Sci Rep 2014; 4: 7480.
High body-mass index	Breast cancer (Post-menopause)	Renehan AG, Tyson M, Egger M, Heller RF, Zwahlen M. Body-mass index and incidence of cancer: a systematic review and meta-analysis of prospective observational studies. Lancet 2008; 371: 569–78.
High body-mass index	Breast cancer (Post-menopause)	Xia X, Chen W, Li J, et al. Body mass index and risk of breast cancer: a nonlinear dose-response meta-analysis of prospective studies. Sci Rep 2014; 4: 7480.
High body-mass index	Uterine cancer	Aune D, Greenwood DC, Chan DSM, et al. Body mass index, abdominal fatness and pancreatic cancer risk: a systematic review and non-linear dose-response meta-analysis of prospective studies. Ann Oncol 2012; 23: 843–52.
High body-mass index	Uterine cancer	Jenabi E, Poorolajal J. The effect of body mass index on endometrial cancer: a meta-analysis. Public Health 2015; 129: 872–80.
High body-mass index	Uterine cancer	Renehan AG, Tyson M, Egger M, Heller RF, Zwahlen M. Body-mass index and incidence of cancer: a systematic review and meta-analysis of prospective observational studies. Lancet 2008; 371: 569–78.
High body-mass index	Ovarian cancer	Aune D, Navarro Rosenblatt DA, Chan DSM, et al. Anthropometric factors and ovarian cancer risk: a systematic review and nonlinear dose-response meta-analysis of prospective studies. Int J Cancer 2015; 136: 1888–98.
High body-mass index	Ovarian cancer	Collaborative Group on Epidemiological Studies of Ovarian Cancer. Ovarian cancer and body size: individual participant meta-analysis including 25,157 women with ovarian cancer from 47 epidemiological studies. PLoS Med 2012; 9: e1001200.
High body-mass index	Ovarian cancer	Liu Z, Zhang T-T, Zhao J-J, et al. The association between overweight, obesity and ovarian cancer: a meta-analysis. Jpn J Clin Oncol 2015; 45: 1107–15.
High body-mass index	Ovarian cancer	Renehan AG, Tyson M, Egger M, Heller RF, Zwahlen M. Body-mass index and incidence of cancer: a systematic review and meta-analysis of prospective observational studies. Lancet 2008; 371: 569–78.
High body-mass index	Kidney cancer	Renehan AG, Tyson M, Egger M, Heller RF, Zwahlen M. Body-mass index and incidence of cancer: a systematic review and meta-analysis of prospective observational studies. Lancet 2008; 371: 569–78.
High body-mass index	Kidney cancer	Wang F, Xu Y. Body mass index and risk of renal cell cancer: a dose-response meta-analysis of published cohort studies. Int J Cancer 2014; 135: 1673–86.
High body-mass index	Thyroid cancer	Ma J, Huang M, Wang L, Ye W, Tong Y, Wang H. Obesity and risk of thyroid cancer: evidence from a meta-analysis of 21 observational studies. Med Sci Monit 2015; 21: 283–91.
High body-mass index	Thyroid cancer	Renehan AG, Tyson M, Egger M, Heller RF, Zwahlen M. Body-mass index and incidence of cancer: a systematic review and meta-analysis of prospective observational studies. Lancet. 2008; 371(9612): 569-78.
High body-mass index	Leukaemia	Castillo JJ, Reagan JL, Ingham RR, et al. Obesity but not overweight increases the incidence and mortality of leukemia in adults: a meta-analysis of prospective cohort studies. Leuk Res 2012; 36: 868–75.
High body-mass index	Leukaemia	Renehan AG, Tyson M, Egger M, Heller RF, Zwahlen M. Body-mass index and incidence of cancer: a systematic review and meta-analysis of prospective observational studies. Lancet. 2008; 371(9612): 569-78.
High body-mass index	Ischaemic heart disease	Singh GM, Danaei G, Farzadfar F, et al. The age-specific quantitative effects of metabolic risk factors on cardiovascular diseases and diabetes: a pooled analysis. PLoS ONE 2013; 8: e65174.
High body-mass index	Cerebrovascular disease	Singh GM, Danaei G, Farzadfar F, et al. The age-specific quantitative effects of metabolic risk factors on cardiovascular diseases and diabetes: a pooled analysis. PLoS ONE 2013; 8: e65174.
High body-mass index	Hypertensive heart disease	Singh GM, Danaei G, Farzadfar F, et al. The age-specific quantitative effects of metabolic risk factors on cardiovascular diseases and diabetes: a pooled analysis. PLoS ONE 2013; 8: e65174.
High body-mass index	Diabetes mellitus	Singh GM, Danaei G, Farzadfar F, et al. The age-specific quantitative effects of metabolic risk factors on cardiovascular diseases and diabetes: a pooled analysis. PLoS ONE 2013; 8: e65174.
High body-mass index	Chronic kidney disease	Emerging Risk Factors Collaboration, Wormser D, Kaptoge S, et al. Separate and combined associations of body-mass index and abdominal adiposity with cardiovascular disease: collaborative analysis of 58 prospective studies. Lancet 2011; 377: 1085–95.

Table S8. Sources of epidemiological evidence used for assessment of causality between high body mass index and disease endpoints.		
**Risk**	**Outcome**	**Citation/Note**
High body-mass index	Chronic kidney disease	Ni Mhurchu C, Rodgers A, Pan WH, Gu DF, Woodward M, Asia Pacific Cohort Studies Collaboration. Body mass index and cardiovascular disease in the Asia-Pacific Region: an overview of 33 cohorts involving 310 000 participants. Int J Epidemiol 2004; 33: 751–8.
High body-mass index	Chronic kidney disease	Prospective Studies Collaboration, Whitlock G, Lewington S, et al. Body-mass index and cause-specific mortality in 900 000 adults: collaborative analyses of 57 prospective studies. Lancet 2009; 373: 1083–96.
High body-mass index	Osteoarthritis	Jiang L, Rong J, Wang Y, et al. The relationship between body mass index and hip osteoarthritis: a systematic review and meta-analysis. Joint Bone Spine 2011; 78: 150–5.
High body-mass index	Osteoarthritis	Jiang L, Tian W, Wang Y, et al. Body mass index and susceptibility to knee osteoarthritis: a systematic review and meta-analysis. Joint Bone Spine 2012; 79: 291–7.
High body-mass index	Osteoarthritis	Silverwood V, Blagojevic-Bucknall M, Jinks C, Jordan JL, Protheroe J, Jordan KP. Current evidence on risk factors for knee osteoarthritis in older adults: a systematic review and meta-analysis. Osteoarthr Cartil 2015; 23: 507–15.
High body-mass index	Low back pain	Shiri R, Karppinen J, Leino-Arjas P, Solovieva S, Viikari-Juntura E. The association between obesity and low back pain: a meta-analysis. Am J Epidemiol 2010; 171: 135–54.

Table S9. Citations for all sources used for estimating body mass index, overweight, and obesity.
AA al-Nuaim; EA Bamgboye; KA al-Rubeaan; Y al-Mazrou. Overweight and obesity in Saudi Arabian adult population, role of socio-demographic variables. J Community Health. 1997; 22(3): 211-23.
Aarhus University, Addiction Switzerland Research Institute, Alcohol Research Group, Public Health Institute, Centre for Addiction and Mental Health (Canada), Centre for Alcohol Policy Research, Turning Point Alcohol and Drug Centre (Australia), Kettil Bruun Society for Social and Epidemiological Research on Alcohol, University of North Dakota. Belize Gender, Alcohol and Culture: An International Study (GENACIS) 2005 [Unpublished].
Aarhus University, Addiction Switzerland Research Institute, Alcohol Research Group, Public Health Institute, Centre for Addiction and Mental Health (Canada), Centre for Alcohol Policy Research, Turning Point Alcohol and Drug Centre (Australia), Kettil Bruun Society for Social and Epidemiological Research on Alcohol, University of North Dakota. Brazil - Botucatu Gender, Alcohol and Culture: An International Study (GENACIS) 2001-2002 [Unpublished].
Aarhus University, Addiction Switzerland Research Institute, Alcohol Research Group, Public Health Institute, Centre for Addiction and Mental Health (Canada), Centre for Alcohol Policy Research, Turning Point Alcohol and Drug Centre (Australia), Kettil Bruun Society for Social and Epidemiological Research on Alcohol, University of North Dakota. Canada Gender, Alcohol and Culture: An International Study (GENACIS) 2004 [Unpublished].
Aarhus University, Addiction Switzerland Research Institute, Alcohol Research Group, Public Health Institute, Centre for Addiction and Mental Health (Canada), Centre for Alcohol Policy Research, Turning Point Alcohol and Drug Centre (Australia), Kettil Bruun Society for Social and Epidemiological Research on Alcohol, University of North Dakota. Costa Rica Gender, Alcohol and Culture: An International Study (GENACIS) 2003 [Unpublished].
Aarhus University, Addiction Switzerland Research Institute, Alcohol Research Group, Public Health Institute, Centre for Addiction and Mental Health (Canada), Centre for Alcohol Policy Research, Turning Point Alcohol and Drug Centre (Australia), Kettil Bruun Society for Social and Epidemiological Research on Alcohol, University of North Dakota. Denmark Gender, Alcohol and Culture: An International Study (GENACIS) 2003 [Unpublished].
Aarhus University, Addiction Switzerland Research Institute, Alcohol Research Group, Public Health Institute, Centre for Addiction and Mental Health (Canada), Centre for Alcohol Policy Research, Turning Point Alcohol and Drug Centre (Australia), Kettil Bruun Society for Social and Epidemiological Research on Alcohol, University of North Dakota. France Gender, Alcohol and Culture: An International Study (GENACIS) 1999 [Unpublished].
Aarhus University, Addiction Switzerland Research Institute, Alcohol Research Group, Public Health Institute, Centre for Addiction and Mental Health (Canada), Centre for Alcohol Policy Research, Turning Point Alcohol and Drug Centre (Australia), Kettil Bruun Society for Social and Epidemiological Research on Alcohol, University of North Dakota. Germany Gender, Alcohol and Culture: An International Study (GENACIS) 2000 [Unpublished].
Aarhus University, Addiction Switzerland Research Institute, Alcohol Research Group, Public Health Institute, Centre for Addiction and Mental Health (Canada), Centre for Alcohol Policy Research, Turning Point Alcohol and Drug Centre (Australia), Kettil Bruun Society for Social and Epidemiological Research on Alcohol, University of North Dakota. Iceland Gender, Alcohol and Culture: An International Study (GENACIS) 2001 [Unpublished].

Aarhus University, Addiction Switzerland Research Institute, Alcohol Research Group, Public Health Institute, Centre for Addiction and Mental Health (Canada), Centre for Alcohol Policy Research, Turning Point Alcohol and Drug Centre (Australia), Kettil Bruun Society for Social and Epidemiological Research on Alcohol, University of North Dakota. India - Karnataka Gender, Alcohol and Culture: An International Study (GENACIS) 2003 [Unpublished].

Aarhus University, Addiction Switzerland Research Institute, Alcohol Research Group, Public Health Institute, Centre for Addiction and Mental Health (Canada), Centre for Alcohol Policy Research, Turning Point Alcohol and Drug Centre (Australia), Kettil Bruun Society for Social and Epidemiological Research on Alcohol, University of North Dakota. Japan Gender, Alcohol and Culture: An International Study (GENACIS) 2001 [Unpublished].

Aarhus University, Addiction Switzerland Research Institute, Alcohol Research Group, Public Health Institute, Centre for Addiction and Mental Health (Canada), Centre for Alcohol Policy Research, Turning Point Alcohol and Drug Centre (Australia), Kettil Bruun Society for Social and Epidemiological Research on Alcohol, University of North Dakota. Mexico Gender, Alcohol and Culture: An International Study (GENACIS) 1998 [Unpublished].

Aarhus University, Addiction Switzerland Research Institute, Alcohol Research Group, Public Health Institute, Centre for Addiction and Mental Health (Canada), Centre for Alcohol Policy Research, Turning Point Alcohol and Drug Centre (Australia), Kettil Bruun Society for Social and Epidemiological Research on Alcohol, University of North Dakota. New Zealand Gender, Alcohol and Culture: An International Study (GENACIS) 2007 [Unpublished].

Aarhus University, Addiction Switzerland Research Institute, Alcohol Research Group, Public Health Institute, Centre for Addiction and Mental Health (Canada), Centre for Alcohol Policy Research, Turning Point Alcohol and Drug Centre (Australia), Kettil Bruun Society for Social and Epidemiological Research on Alcohol, University of North Dakota. Sri Lanka Gender, Alcohol and Culture: An International Study (GENACIS) 2002 [Unpublished].

Aarhus University, Addiction Switzerland Research Institute, Alcohol Research Group, Public Health Institute, Centre for Addiction and Mental Health (Canada), Centre for Alcohol Policy Research, Turning Point Alcohol and Drug Centre (Australia), Kettil Bruun Society for Social and Epidemiological Research on Alcohol, University of North Dakota. Sweden Gender, Alcohol and Culture: An International Study (GENACIS) 2002 [Unpublished].

Aarhus University, Addiction Switzerland Research Institute, Alcohol Research Group, Public Health Institute, Centre for Addiction and Mental Health (Canada), Centre for Alcohol Policy Research, Turning Point Alcohol and Drug Centre (Australia), Kettil Bruun Society for Social and Epidemiological Research on Alcohol, University of North Dakota. Uganda Gender, Alcohol and Culture: An International Study (GENACIS) 2003 [Unpublished].

Aarhus University, Addiction Switzerland Research Institute, Alcohol Research Group, Public Health Institute, Centre for Addiction and Mental Health (Canada), Centre for Alcohol Policy Research, Turning Point Alcohol and Drug Centre (Australia), Kettil Bruun Society for Social and Epidemiological Research on Alcohol, University of North Dakota. United Kingdom Gender, Alcohol and Culture: An International Study (GENACIS) 2000 [Unpublished].

Aarhus University, Addiction Switzerland Research Institute, Alcohol Research Group, Public Health Institute, Centre for Addiction and Mental Health (Canada), Centre for Alcohol Policy Research, Turning Point Alcohol and Drug Centre (Australia), Kettil Bruun Society for Social and Epidemiological Research on Alcohol, University of North Dakota. Uruguay Gender, Alcohol and Culture: An International Study (GENACIS) 2004 [Unpublished].

Aarhus University, Addiction Switzerland Research Institute, Alcohol Research Group, Public Health Institute, Centre for Addiction and Mental Health (Canada), Centre for Alcohol Policy Research, Turning Point Alcohol and Drug Centre (Australia), Kettil Bruun Society for Social and Epidemiological Research on Alcohol, University of North Dakota. United States Gender, Alcohol and Culture: An International Study (GENACIS) 2000 [Unpublished].

Abdul-Rahim HF, Abu-Rmeileh NM, Husseini A, Holmboe-Ottesen G, Jervell J, Bjertness E. Obesity and selected co-morbidities in an urban Palestinian population. Int J Obes Relat Metab Disord. 2001; 25(11): 1736-40.

Abrantes MM, Lamounier JA, Colosimo EA. Prevalência de sobrepeso e obesidade nas regiões Nordeste e Sudeste do Brasil. Rev Assoc Med Bras. 2003; 49(2): 162-6.

Abu Dhabi General Authority for Health Services, Centers for Disease Control and Prevention (CDC), Dubai Health Authority, Ministry of Education (United Arab Emirates), Ministry of Health (United Arab Emirates), World Health Organization (WHO). United Arab Emirates Global School-Based Student Health Survey 2005. Geneva, Switzerland: World Health Organization (WHO).

Academy of Preventive Medicine (Kazakhstan), Macro International, Inc. Kazakhstan Demographic and Health Survey 1999. Calverton, United States: Macro International, Inc.

Achat HM, Stubbs JM. Socio-economic and ethnic differences in the prevalence of overweight and obesity among school children. J Paediatr Child Health. 2014; 50(10): E77-84.

ACIL Australia Pty Ltd., Australian National University, Ministry of Health (Timor-Leste), National Statistics Directorate (Timor-Leste), University of Newcastle (Australia). Timor-Leste Demographic and Health Survey 2003. Newcastle, Australia: University of Newcastle (Australia).

Ackerman IN, Osborne RH. Obesity and increased burden of hip and knee joint disease in Australia: results from a national survey. BMC Musculoskelet Disord. 2012; 13(1): 254.

Administrative Department of Science, Techonology, and Innovation (Colombia), Center for Development Projects, Pontifical Xavierian University, Ministry of Social Protection (Colombia), Specialized Information Systems. Colombia National Health Survey 2007-2008.

Adolescent Health Research Group, University of Auckland, Auckland UniServices, University of Auckland (New Zealand), Faculty of Medical and Health Sciences, University of Auckland. New Zealand Youth2012 Health and Wellbeing of Secondary School Students 2012.

Adventist Development and Relief Agency (ADRA), Azerbaijan Ministry of Health, State Statistical Committee of Azerbaijan, and Centers for Disease Control and Prevention (CDC). (2003) Azerbaijan Reproductive Health Survey 2001. Atlanta, United States: Centers for Disease Control and Prevention (CDC).

Aga Khan University, Central Statistics Organization (Afghanistan), Ministry of Public Health (Afghanistan), United Nations Children's Fund (UNICEF). Afghanistan National Nutrition Survey 2013.

Aga Khan University, Ministry of National Health Services, Regulations & Coordination (Pakistan), Ministry of Planning and Development (Pakistan), Pakistan Medical Research Council, United Nations Children's Fund (UNICEF). Pakistan National Nutrition Survey 2011.

AGB Attwood, Ministry of Agriculture, Nature Management, and Fisheries (Netherlands), Ministry of Welfare, Health, and Culture (Netherlands), TNO Nutrition. Netherlands Dutch National Food Consumption Survey 1987-1988.

AGB Fresh Foods, Ministry of Agriculture, Nature Management, and Fisheries (Netherlands), TNO Nutrition. Netherlands Dutch National Food Consumption Survey 1992.

Agency for Statistics (Bosnia and Herzegovina), Federal Ministry of Health (Bosnia and Herzegovina), Ministry of Health and Social Welfare (Republic of Srpska), Public Health Institute of Federation of Bosnia and Herzegovina, United Nations Children's Fund (UNICEF), United Nations Entity for Gender Equality and the Empowerment of Women (UN Women). Bosnia and Herzegovina Multiple Indicator Cluster Survey 2011-2012.

Agency for Statistics (Bosnia and Herzegovina), Ministry of Health (Federation of Bosnia and Herzegovina), Ministry of Health and Social Welfare (Republic of Srpska), United Nations Children's Fund (UNICEF). Bosnia and Herzegovina Multiple Indicator Cluster Survey 2000. New York, United States: United Nations Children's Fund (UNICEF).

Agency of the Republic of Kazakhstan on Statistics and United Nations Children's Fund (UNICEF). Kazakhstan Multiple Indicator Cluster Survey 2006. New York, United States: United Nations Children's Fund (UNICEF).

Agency of the Republic of Kazakhstan on Statistics, United Nations Children's Fund (UNICEF). Kazakhstan Multiple Indicator Cluster Survey 2010-2011. New York, United States: United Nations Children's Fund (UNICEF), 2013.

Agirbasli M, Cakir S, Ozme S, Ciliv G. Metabolic syndrome in Turkish children and adolescents. Metab Clin Exp. 2006; 55(8): 1002-6.

Ahti TM, Mäkivaara LA, Luukkaala T, Hakama M, Laurikka JO. Lifestyle factors and varicose veins: does cross-sectional design result in underestimate of the risk?. Phlebology. 2010; 25(4): 201-6.

Al Zenki S, Al Omirah H, Al Hooti S, Al Hamad N, Jackson RT, Rao A, Al Jahmah N, Al Obaid I, Al Ghanim J, Al Somaie M, Zaghloul S, Al Othman A. High prevalence of metabolic syndrome among Kuwaiti adults--a wake-up call for public health intervention. Int J Environ Res Public Health. 2012; 9(5): 1984-96.

Al-Almaie SM. Prevalence of obesity and overweight among Saudi adolescents in Eastern Saudi Arabia. Saudi Med J. 2005; 26(4): 607-11.

Al-Baghli NA, Al-Ghamdi AJ, Al-Turki KA, El-Zubaier AG, Al-Ameer MM, Al-Baghli FA. Overweight and obesity in the eastern province of Saudi Arabia. Saudi Med J. 2008; 29(9): 1319-25.

Albertini A, Tripodi A, Fabbri A, Mattioli M, Cavrini G, Cecchetti R, Dalle Donne E, Cortesi C, De Giorgi S, Contarini V, Andreotti L, Veronesi B, Stefanelli I, Di Martino E. Prevalence of obesity in 6- and 9-year-old children living in Central-North Italy. Analysis of determinants and indicators of risk of overweight. Obes Rev. 2008; 9(1): 4-10.

Alcohol Research Group, Public Health Institute. India - Goa Alcohol Use Study 2004-2008.

Al-Daghri NM, Al-Attas OS, Alokail MS, Alkharfy KM, Yousef M, Sabico SL, Chrousos GP. Diabetes mellitus type 2 and other chronic non-communicable diseases in the central region, Saudi Arabia (Riyadh cohort 2): a decade of an epidemic. BMC Med. 2011; 76.

Al-Habori M, Al-Mamari M, Al-Meeri A. Type II diabetes mellitus and impaired glucose tolerance in Yemen: prevalence, associated metabolic changes and risk factors. Diabetes Res Clin Pract. 2004; 65(3): 275-81.

Al-Haddad FH, Little BB, Abdul Ghafoor AGM. Childhood obesity in United Arab Emirates schoolchildren: a national study. Ann Hum Biol. 2005; 32(1): 72-9.

Alikor CA, Emem-Chioma PC. EPIDEMIOLOGY OF DIABETES AND IMPAIRED FASTING GLUCOSE IN A RURAL COMMUNITY OF NIGERIAN NIGER DELTA REGION. Niger J Med. 2015; 24(2): 114–24.

Allafi A, Al-Haifi AR, Al-Fayez MA, Al-Athari BI, Al-Ajmi FA, Al-Hazzaa HM, Musaiger AO, Ahmed F. Physical activity, sedentary behaviours and dietary habits among Kuwaiti adolescents: gender differences. Public Health Nutr. 2014; 17(9): 2045–52.

Al-Lawati JA, Jousilahti PJ. Prevalence and 10-year secular trend of obesity in Oman. Saudi Med J. 2004; 25(3): 346-51.

Alm A, Fåhraeus C, Wendt L-K, Koch G, Andersson-Gäre B, Birkhed D. Body adiposity status in teenagers and snacking habits in early childhood in relation to approximal caries at 15 years of age. Int J Paediatr Dent. 2008; 18(3): 189-96.

Al-Muhaimeed AA, Dandash K, Ismail MS, Saquib N. Prevalence and correlates of overweight status among Saudi school children. Ann Saudi Med. 2015; 35(4): 275–81.

Al-Nozha MM, Al-Mazrou YY, Al-Maatouq MA, Arafah MR, Khalil MZ, Khan NB, Al-Marzouki K, Abdullah MA, Al-Khadra AH, Al-Harthi SS, Al-Shahid MS, Al-Mobeireek A, Nouh MS. Obesity in Saudi Arabia. Saudi Med J. 2005; 26(5): 824-9.

al-Nuaim AR, al-Rubeaan K, al-Mazrou Y, al-Attas O, al-Daghari N, Khoja T. High prevalence of overweight and obesity in Saudi Arabia. Int J Obes Relat Metab Disord. 1996; 20(6): 547-52.

Al-Othaimeen AI, Al-Nozha M, Osman AK. Obesity: an emerging problem in Saudi Arabia. Analysis of data from the National Nutrition Survey. East Mediterr Health J. 2007; 13(2): 441-8.

Al-Raees GY, Al-Amer MA, Musaiger AO, D'Souza R. Prevalence of overweight and obesity among children aged 2-5 years in Bahrain: a comparison between two reference standards. Int J Pediatr Obes. 2009; 4(4): 414-6.

Al-Sendi AM, Shetty P, Musaiger AO. Prevalence of overweight and obesity among Bahraini adolescents: a comparison between three different sets of criteria. Eur J Clin Nutr. 2003; 57(3): 471-4.

Amaral C de A, Portela MC, Muniz PT, Farias E dos S, Araujo TS de, Souza OF de. Association of handgrip strength with self-reported diseases in adults in Rio Branco, Acre State, Brazil: a population-based study. Cad Saude Publica. 2015; 31(6): 1313–25.

AMATEM (Turkey), Plaza Ltd. Research, World Health Organization (WHO). Turkey WHO Multi-country Survey Study on Health and Health System Responsiveness 2000-2001. Geneva, Switzerland: World Health Organization (WHO).

American University of Beirut, World Health Organization (WHO). Lebanon STEPS Noncommunicable Disease Risk Factors Survey 2008-2009.

Amin TT, Al-Sultan AI, Ali A. Overweight and obesity and their relation to dietary habits and socio-demographic characteristics among male primary school children in Al-Hassa, Kingdom of Saudi Arabia. Eur J Nutr. 2008; 47(6): 310-8.

Andrews, Gary R., and George C. Myers. Australian [Adelaide] Longitudinal Study of Aging, Waves 1-5 [1992-1997]. ICPSR06707-v3. Ann Arbor, MI: Inter-university Consortium for Political and Social Research [distributor], 2000. doi:10.3886/ICPSR06707.v3

Androutsos O, Grammatikaki E, Moschonis G, Roma-Giannikou E, Chrousos GP, Manios Y, Kanaka-Gantenbein C. Neck circumference: a useful screening tool of cardiovascular risk in children. Pediatr Obes. 2012; 7(3): 187-95.

Angbratt M, Ekberg J, Walter L, Timpka T. Prediction of obesity from infancy to adolescence. Acta Paediatr. 2011; 100(9): 1249-52.

Aounallah-Skhiri H, Romdhane HB, Traissac P, Eymard-Duvernay S, Delpeuch F, Achour N, Maire B. Nutritional status of Tunisian adolescents: associated gender, environmental and socio-economic factors. Public Health Nutr. 2008; 11(12): 1306-17.

Aoyagi K, Kusano Y, Takamura N, Abe Y, Osaki M, Une H. Obesity and cardiovascular risk factors among men and women aged 40 years and older in a rural area of Japan. J Physiol Anthropol. 2006; 25(6): 371-5.

Aradillas-García C, Malacara JM, Garay-Sevilla ME, Guízar JM, Camacho N, De la Cruz-Mendoza E, Quemada L, Sierra JFH. Prediabetes in rural and urban children in 3 states in Mexico. J Cardiometab Syndr. 2007; 2(1): 35-9.

Armstrong MEG, Lambert MI, Sharwood KA, Lambert EV. Obesity and overweight in South African primary school children -- the Health of the Nation Study. S Afr Med J. 2006; 96(5): 439-44.

Arroyo P, Loria A, Fernández V, Flegal KM, Kuri-Morales P, Olaiz G, Tapia-Conyer R. Prevalence of pre-obesity and obesity in urban adult Mexicans in comparison with other large surveys. Obes Res. 2000; 8(2): 179-85.

Asfour MG, Lambourne A, Soliman A, Al-Behlani S, Al-Asfoor D, Bold A, Mahtab H, King H. High prevalence of diabetes mellitus and impaired glucose tolerance in the Sultanate of Oman: results of the 1991 national survey. Diabet Med. 1995; 12(12): 1122-5.

Asmar R, Vol S, Pannier B, Brisac AM, Tichet J, El Hasnaoui A. High blood pressure and associated cardiovascular risk factors in France. J Hypertens. 2001; 19(10): 1727-32.

Asociación Demográfica Salvadoreña (ADS), Division of Reproductive Health-Centers for Disease Control and Prevention (CDC). (2004) El Salvador Reproductive Health Survey 2002-2003. San Salvador, El Salvador: ADS.

Asociación Demográfica Salvadoreña (ADS), Division of Reproductive Health-Centers for Disease Control and Prevention (CDC). (2009) El Salvador Reproductive Health Survey 2008. San Salvador, El Salvador: ADS.

Aspray TJ, Mugusi F, Rashid S, Whiting D, Edwards R, Alberti KG, Unwin NC, Essential Non-Communicable Disease Health Intervention Project. Rural and urban differences in diabetes prevalence in Tanzania: the role of obesity, physical inactivity and urban living. Trans R Soc Trop Med Hyg. 2000; 94(6): 637-44.

Associates for Community and Population Research (ACPR), International Centre for Diarrhoeal Disease Research (Bangladesh), MEASURE Evaluation Project, Carolina Population Center, University of North Carolina, National Institute of Population Research and Training (NIPORT). Bangladesh Urban Health Survey 2006.

Australian Agency for International Development (AusAID), Fiji School of Medicine, Menzies Center for Population Health Research, University of Tasmania (Australia), Ministry of Health (Fiji), World Health Organization (WHO). Fiji STEPS Noncommunicable Disease Risk Factors Survey 2002.

Australian Bureau of Statistics, Australian Institute of Family Studies, Department of Families, Housing, Community Services and Indigenous Affairs (Australia). Australia Longitudinal Study of Children, Wave 4 2009-2010. Melbourne, Australia: Australian Institute of Family Studies.

Australian Bureau of Statistics, Department of Health and Family Services (Australia). Australia National Nutrition Survey 1995-1996. Canberra, Australia: Australian Bureau of Statistics.

Australian Bureau of Statistics. Australia National Health Survey 2001. Canberra, Australia: Australian Bureau of Statistics.

Australian Bureau of Statistics. Australia National Health Survey 2004-2005.

Australian Bureau of Statistics. Australia National Health Survey 2007-2008. Canberra, Australia: Australian Bureau of Statistics.

Australian Bureau of Statistics. Australia National Health Survey 2011-2013.

Australian Bureau of Statistics. Australia National Health Survey 1995. Canberra, Australia: Australian Bureau of Statistics.

Australian Institute of Health and Welfare, National Heart Foundation (Australia). Australia Risk Factor Prevalence Survey 1989.

Australian Institute of Health and Welfare, National Heart Foundation (Australia). Australia Risk Factor Prevalence Survey 1994.

Azerbaijan Ministry of Health, GroundWork LLC, State Statistical Committee of Azerbaijan, United Nations Children's Fund (UNICEF). Azerbaijan Nutrition Survey 2013.

Baker IDI Heart and Diabetes Institute, Imperial College London, Ministry of Health and Quality of Life (Mauritius), National Public Health Institute (Finland), Umeå University. Mauritius Noncommunicable Disease Survey 2009.

Baker IDI Heart and Diabetes Institute, International Diabetes Institute (IDI), Monash University (Australia), University of Melbourne. Australia Diabetes, Obesity and Lifestyle Study 2011-2012.

Baltazar JC, Ancheta CA, Aban IB, Fernando RE, Baquilod MM. Prevalence and correlates of diabetes mellitus and impaired glucose tolerance among adults in Luzon, Philippines. Diabetes Res Clin Pract. 2004; 64(2): 107-15.

Bangladesh Bureau of Statistics, Government of Bangladesh, Ministry of Planning (Bangladesh), United Nations Children's Fund (UNICEF). Bangladesh Multiple Indicator Cluster Survey 2012-2013. New York, United States: United Nations Children's Fund (UNICEF), 2015.

Bangladesh Society of Medicine, Directorate General of Health Services, Ministry of Health and Family Welfare (Bangladesh), Ministry of Health and Family Welfare (Bangladesh), World Health Organization (WHO). Bangladesh STEPS Noncommunicable Disease Risk Factors Survey 2009-2010.

Barbados Statistical Service, Caribbean Food and Nutrition Institute, Pan American Health Organization, Ministry of Health (Barbados). Barbados Food Consumption and Anthropometric Survey 2000-2001.

Barbados Statistical Service, United Nations Children's Fund (UNICEF). Barbados Multiple Indicator Cluster Survey 2012. New York, United States: United Nations Children's Fund (UNICEF).

Barbu CG, Teleman MD, Albu AI, Sirbu AE, Martin SC, Bancescu A, Fica SV. Obesity and eating behaviors in school children and adolescents -data from a cross sectional study from Bucharest, Romania. BMC Public Health. 2015; 15: 206.

Bayer O, Rosario AS, Wabitsch M, von Kries R. Sleep duration and obesity in children: is the association dependent on age and choice of the outcome parameter?. Sleep. 2009; 32(9): 1183-9.

Bener A, Tewfik I. Prevalence of overweight, obesity, and associated psychological problems in Qatari's female population. Obes Rev. 2006; 7(2): 139-45.

Bener A, Zirie M, Musallam M, Khader YS, Al-Hamaq AOAA. Prevalence of metabolic syndrome according to Adult Treatment Panel III and International Diabetes Federation criteria: a population-based study. Metab Syndr Relat Disord. 2009; 7(3): 221-9.

Berdasco A. Body mass index values in the Cuban adult population. Eur J Clin Nutr. 1994; S155-164.

Bere E, Westersjo JH. Nature trips and traditional methods for food procurement in relation to weight status. Scand J Public Health. 2013; 41(2): 180-4.

Berentzen NE, van Stokkom VL, Gehring U, Koppelman GH, Schaap LA, Smit HA, Wijga AH. Associations of sugar-containing beverages with asthma prevalence in 11-year-old children: the PIAMA birth cohort. Eur J Clin Nutr. 2015; 69(3): 303–8.

Berraho M, Nejjari C, Raherison C, El Achhab Y, Tachfouti N, Serhier Z, Dartigues JF, Barberger-Gateau P. Body mass index, disability, and 13-year mortality in older French adults. J Aging Health. 2010; 22(1): 68-83.

Berta Vanrullen I, Volatier J-L, Bertaut A, Dufour A, Dallongeville J. Characteristics of energy intake under-reporting in French adults. Br J Nutr. 2014; 111(7): 1292–302.

Bertoncello C, Cazzaro R, Ferraresso A, Mazzer R, Moretti G. Prevalence of overweight and obesity among school-aged children in urban, rural and mountain areas of the Veneto Region, Italy. Public Health Nutr. 2008; 11(9): 887-90.

Bhagyalaxmi A, Atul T, Shikha J. Prevalence of risk factors of non-communicable diseases in a District of Gujarat, India. J Health Popul Nutr. 2013; 31(1): 78-85.

Bharati S, Pal M, Bhattacharya BN, Bharati P. Prevalence and causes of chronic energy deficiency and obesity in Indian women. Hum Biol. 2007; 79(4): 395-412.

Bhowmik B, Munir SB, Ahmed KR, Siddiquee T, Diep LM, Wright E, Hassan Z, Debnath PR, Mahtab H, Azad Khan AK, Hussain A. Anthropometric indices of obesity and type 2 diabetes in Bangladeshi population: Chandra Rural Diabetes Study (CRDS). Obes Res Clin Pract. 2014; 8(3): e201–98.

Bibiloni MM, Salas R, Pons A, Tur JA. Prevalence of dyslipidaemia and associated risk factors among Balearic Islands adolescents, a Mediterranean region. Eur J Clin Nutr. 2015; 69(6): 722–8.

Bigornia SJ, LaValley MP, Noel SE, Moore LL, Ness AR, Newby PK. Sugar-sweetened beverage consumption and central and total adiposity in older children a prospective study accounting for dietary reporting errors. Public Health Nutr. 2015; 18(7): 1155–63.

Binkin N, Fontana G, Lamberti A, Cattaneo C, Baglio G, Perra A, Spinelli A. A national survey of the prevalence of childhood overweight and obesity in Italy. Obes Rev. 2010; 11(1): 2-10.

Binkowska-Bury M, Zal M, Wolan M, Sobolewski M, Januszewicz P, Bolanowski M, Mazur A. Secular trends in BMI changes among the military population between 2000 and 2010 in Poland - a retrospective study. Neuro Endocrinol Lett. 2013; 34(8): 814–20.

Birzeit University, Palestinian Central Bureau of Statistics. Palestine Nutrition Survey 2002. Ramallah, Palestine: Palestinian Central Bureau of Statistics.

Bjelland M, Bergh IH, Grydeland M, Klepp K-I, Andersen LF, Anderssen SA, Ommundsen Y, Lien N. Changes in adolescents' intake of sugar-sweetened beverages and sedentary behaviour: results at 8 month mid-way assessment of the HEIA study--a comprehensive, multi-component school-based randomized trial. Int J Behav Nutr Phys Act. 2011; 63.

Boneberger A, von Kries R, Milde-Busch A, Bolte G, Rochat MK, Rückinger S, GME Study Group. Association between peer relationship problems and childhood overweight/obesity. Acta Paediatr. 2009; 98(12): 1950-5.

Booth ML, Dobbins T, Okely AD, Denney-Wilson E, Hardy LL. Trends in the prevalence of overweight and obesity among young Australians, 1985, 1997, and 2004. Obesity (Silver Spring). 2007; 15(5): 1089-95.

Booth ML, Okely AD, Denney-Wilson E. Validation and application of a novel method of measuring non-response bias in school-based surveys of paediatric overweight and obesity. Int J Pediatr Obes. 2011; 6(2-2): e87-93.

Booth ML, Wake M, Armstrong T, Chey T, Hesketh K, Mathur S. The epidemiology of overweight and obesity among Australian children and adolescents, 1995-97. Aust N Z J Public Health. 2001; 25(2): 162-9.

Borici S, Agaoglu NB, Baykan OA, Agirbasli M. Blood pressure and anthropometric measurements in Albanian versus Turkish children and adolescents. Acta Cardiol. 2009; 64(6): 747-54.

Börsch-Supan, A. (2013). Survey of Health, Ageing and Retirement in Europe (SHARE) Wave 1. Release version: 2.6.0. SHARE-ERIC. Data set. DOI: 10.6103/SHARE.w1.260

Börsch-Supan, A. (2013). Survey of Health, Ageing and Retirement in Europe (SHARE) Wave 2. Release version: 2.6.0. SHARE-ERIC. Data set. DOI: 10.6103/SHARE.w2.260

Börsch-Supan, A. (2013). Survey of Health, Ageing and Retirement in Europe (SHARE) Wave 4. Release version: 1.1.1. SHARE-ERIC. Data set. DOI: 10.6103/SHARE.w4.111

Bovet P, Chiolero A, Shamlaye C, Paccaud F. Prevalence of overweight in the Seychelles: 15 year trends and association with socio-economic status. Obes Rev. 2008; 9(6): 511-7.

Bovet P, Kizirian N, Madeleine G, Blössner M, Chiolero A. Prevalence of thinness in children and adolescents in the Seychelles: comparison of two international growth references. Open Nutr J. 2011; 65.

Boylan EA, McNulty BA, Walton J, Flynn A, Nugent AP, Gibney MJ. The prevalence and trends in overweight and obesity in Irish adults between 1990 and 2011. Public Health Nutr. 2014; 17(11): 2389–97.

Branca F, Pastore G, Rossi L, Sette S, Stojanovska Ancevska B, Janeva N, Kolevska L, Peova S, Muratovska O, Venovska K. Multiple Indicators Cluster Survey in FYR Macedonia With Micronutrient Component. Rome, Italy: National Institute of Nutrition, 2000.

Brannsether B, Eide GE, Roelants M, Bjerknes R, Júlíusson PB. Interrelationships between anthropometric variables and overweight in childhood and adolescence. Am J Hum Biol. 2014; 26(4): 502–10.

Brazil - Hearts of Brazil Survey 2004

Brazilian Institute of Geography and Statistics (IBGE), World Bank (WB). Brazil Living Standards Measurement Survey 1996-1997. Washington DC, United States: World Bank (WB).

Brazilian Institute of Geography and Statistics (IBGE). Brazil Consumer Expenditure Survey 2002-2003. Rio de Janeiro, Brazil: Brazilian Institute of Geography and Statistics (IBGE).

Brazilian Institute of Geography and Statistics (IBGE). Brazil National Household Sample Survey 2006. Rio de Janeiro, Brazil: Brazilian Institute of Geography and Statistics (IBGE).

Brazilian Society for Family Welfare (BEMFAM), Macro International, Inc. Brazil Demographic and Health Survey 1996. Calverton, United States: Macro International, Inc.

Brazilian Society for Family Welfare (BEMFAM), Westinghouse; Institute for Resource Development. Brazil Demographic and Health Survey 1986. Columbia, United States: Westinghouse; Institute for Resource Development.

Brug J, van Stralen MM, Te Velde SJ, Chinapaw MJM, De Bourdeaudhuij I, Lien N, Bere E, Maskini V, Singh AS, Maes L, Moreno L, Jan N, Kovacs E, Lobstein T, Manios Y. Differences in weight status and energy-balance related behaviors among schoolchildren across Europe: the ENERGY-project. PLoS One. 2012; 7(4): e34742.

Buchmueller TC, Johar M. Obesity and health expenditures: evidence from Australia. Econ Hum Biol. 2015; 17: 42–58.

Buckland G, Salas-Salvadó J, Roure E, Bulló M, Serra-Majem L. Sociodemographic risk factors associated with metabolic syndrome in a Mediterranean population. Public Health Nutr. 2008; 11(12): 1372-8.

Buckland GG, Salas-Salvadó J, Serra-Majem L, Castell C, Cabré J, Salleras-Sanmartí L. Increase in metabolic syndrome as defined by ATPIII from 1992-1993 to 2002-2003 in a Mediterranean population. Nutr Rev. 2009; S117-125.

Bureau of Labor Statistics (United States). United States National Longitudinal Survey of Youth 1997. Washington, D.C., United States: Bureau of Labor Statistics (United States).

Bureau of Labor Statistics (USA). United States National Longitudinal Survey of Youth 1979 Children and Young Adults 1986. Washington, D.C., United States: Bureau of Labor Statistics (USA).

Bureau of Labor Statistics (USA). United States National Longitudinal Survey of Youth 1979 Children and Young Adults 1988. Washington, D.C., United States: Bureau of Labor Statistics (USA).

Bureau of Labor Statistics (USA). United States National Longitudinal Survey of Youth 1979 Children and Young Adults 1990. Washington, D.C., United States: Bureau of Labor Statistics (USA).

Bureau of Labor Statistics (USA). United States National Longitudinal Survey of Youth 1979 Children and Young Adults 1992. Washington, D.C., United States: Bureau of Labor Statistics (USA).

Bureau of Labor Statistics (USA). United States National Longitudinal Survey of Youth 1979 Children and Young Adults 1994. Washington, D.C., United States: Bureau of Labor Statistics (USA).

Bureau of Labor Statistics (USA). United States National Longitudinal Survey of Youth 1979 Children and Young Adults 1996. Washington, D.C., United States: Bureau of Labor Statistics (USA).

Bureau of Labor Statistics (USA). United States National Longitudinal Survey of Youth 1979 Children and Young Adults 1998. Washington, D.C., United States: Bureau of Labor Statistics (USA).

Bureau of Labor Statistics (USA). United States National Longitudinal Survey of Youth 1979 Children and Young Adults 2000. Washington, D.C., United States: Bureau of Labor Statistics (USA).

Bureau of Labor Statistics (USA). United States National Longitudinal Survey of Youth 1979 Children and Young Adults 2002. Washington, D.C., United States: Bureau of Labor Statistics (USA).

Bureau of Labor Statistics (USA). United States National Longitudinal Survey of Youth 1979 Children and Young Adults 2004. Washington, D.C., United States: Bureau of Labor Statistics (USA).

Bureau of Labor Statistics (USA). United States National Longitudinal Survey of Youth 1979 Children and Young Adults 2006. Washington, D.C., United States: Bureau of Labor Statistics (USA).

Bureau of Labor Statistics (USA). United States National Longitudinal Survey of Youth 1979 Children and Young Adults 2008. Washington, D.C., United States: Bureau of Labor Statistics (USA).

Bureau of Labor Statistics (USA). United States National Longitudinal Survey of Youth 1979 Children and Young Adults 2010. Washington, D.C., United States: Bureau of Labor Statistics (USA).

Bureau of Policy and Strategy, Ministry of Public Health (Thailand). Thailand Health Profile 2005-2007. Bangkok, Thailand: Ministry of Public Health (Thailand).

Bureau of Statistics (Guyana), ICF Macro, Ministry of Health (Guyana). Guyana Demographic and Health Survey 2009. Calverton, United States: ICF Macro, 2011.

Bureau of Statistics (Guyana), United Nations Children's Fund (UNICEF). Guyana Multiple Indicator Cluster Survey 2000. New York, United States: United Nations Children's Fund (UNICEF)

Bureau of Statistics (Lesotho), Macro International, Inc, Ministry of Health and Social Welfare (Lesotho). Lesotho Demographic and Health Survey 2004-2005. Calverton, United States: Macro International, Inc.

Bureau of Statistics (Lesotho), United Nations Children's Fund (UNICEF). Lesotho Multiple Indicator Cluster Survey 2000. New York, United States: United Nations Children's Fund (UNICEF).

Bureau of Statistics (Punjab), United Nations Children's Fund (UNICEF). Pakistan - Punjab Multiple Indicator Cluster Survey 2014. New York, United States: United Nations Children's Fund (UNICEF), 2015.

Bureau of Statistics (Tanzania), Macro International, Inc, Ministry of Health (Tanzania). Tanzania Demographic and Health Survey 1991-1992. Calverton, United States: Macro International, Inc.

Bureau of Statistics (Tanzania), Macro International, Inc, Planning Commission (Tanzania). Tanzania Demographic and Health Survey 1996. Calverton, United States: Macro International, Inc.

Bureau of Statistics (Tanzania), Macro International, Inc. Tanzania Demographic and Health Survey 1999. Calverton, United States: Macro International, Inc.

Burundi Institute of Statistics and Economic Studies, ICF International, Ministry of Public Health and the Fight against AIDS (Burundi). Burundi Demographic and Health Survey 2010-2011. Fairfax, United States: ICF International, 2012.

Burundi Institute of Statistics and Economic Studies, United Nations Children's Fund (UNICEF). Burundi Multiple Indicator Cluster Survey 2000. New York, United States: United Nations Children's Fund (UNICEF).

Bustos P, da Silva AAM, Amigo H, Bettiol H, Barbieri MA. Metabolic syndrome in young adults from two socioeconomic Latin American settings. Nutr Metab Cardiovasc Dis. 2007; 17(8): 581-9.

Cadogan SL, Keane E, Kearney PM. The effects of individual, family and environmental factors on physical activity levels in children: a cross-sectional study. BMC Pediatr. 2014; 14: 107.

Caleyachetty R, Rudnicka AR, Echouffo-Tcheugui JB, Siegel KR, Richards N, Whincup PH. Prevalence of overweight, obesity and thinness in 9-10 year old children in Mauritius. Global Health. 2012; 28.

California Center for Population Research (CCPR), University of California Los Angeles (UCLA), Center for Research and Teaching in Economics (CIDE) (Mexico), National Institute of Public Health (Mexico), Universidad Iberoamericana. Mexico Family Life Survey 2005-2006.

Canadian Society for International Health, Ministry of Health (Croatia). Croatia Adult Health Survey 2003.

Canning PM, Courage ML, Frizzell LM. Prevalence of overweight and obesity in a provincial population of Canadian preschool children. CMAJ. 2004; 171(3): 240-2.

Carba DB, Bas IN, Gultiano SA, Lee NR, Adair LS. Waist circumference and the risk of hypertension and prediabetes among Filipino women. Eur J Nutr. 2013; 52(2): 825-32.

CARE International, Macro International, Inc. Niger Demographic and Health Survey 1998. Calverton, United States: Macro International, Inc.

Caribbean Epidemiology Centre (CAREC), Central Statistical Office (Trinidad and Tobago), Ministry of Health (Trinidad and Tobago), Pan American Health Organization (PAHO), University of the West Indies. Trinidad and Tobago STEPS Noncommunicable Disease Risk Factors Survey 2011.

Carolina Population Center, University of North Carolina at Chapel Hill, Chinese Center for Disease Control and Prevention (CCDC). China Health and Nutrition Survey 1989. Chapel Hill, United States: Carolina Population Center, University of North Carolina at Chapel Hill.

Carolina Population Center, University of North Carolina at Chapel Hill, Chinese Center for Disease Control and Prevention (CCDC). China Health and Nutrition Survey 1989-2011. Chapel Hill, United States: Carolina Population Center, University of North Carolina at Chapel Hill.

Carolina Population Center, University of North Carolina at Chapel Hill, Chinese Center for Disease Control and Prevention (CCDC). China Health and Nutrition Survey 1991.

Carolina Population Center, University of North Carolina at Chapel Hill, Chinese Center for Disease Control and Prevention (CCDC). China Health and Nutrition Survey. Chapel Hill, United States: Carolina Population Center, University of North Carolina at Chapel Hill.

Carolina Population Center, University of North Carolina at Chapel Hill, Institute of Sociology, Russian Academy of Sciences, National Research University Higher School of Economics (Russia), ZAO Demoscope. Russia Longitudinal Monitoring Survey of HSE, Round II 1992-1993.

Carolina Population Center, University of North Carolina at Chapel Hill, Institute of Sociology, Russian Academy of Sciences, National Research University Higher School of Economics (Russia), ZAO Demoscope. Russia Longitudinal Monitoring Survey of HSE, Round III 1993.

Carolina Population Center, University of North Carolina at Chapel Hill, Institute of Sociology, Russian Academy of Sciences, National Research University Higher School of Economics (Russia), ZAO Demoscope. Russia Longitudinal Monitoring Survey of HSE, Round IV 1993-1994.

Carolina Population Center, University of North Carolina at Chapel Hill, Institute of Sociology, Russian Academy of Sciences, National Research University Higher School of Economics (Russia), ZAO Demoscope. Russia Longitudinal Monitoring Survey of HSE, Round I 1992.

Carolina Population Center, University of North Carolina at Chapel Hill, Office of Population Studies, University of San Carlos (Philippines). Philippines - Cebu Longitudinal Health and Nutrition Survey 2002-2003. Chapel Hill, United States: Carolina Population Center, University of North Carolina at Chapel Hill.

Carolina Population Center, University of North Carolina at Chapel Hill, Office of Population Studies, University of San Carlos (Philippines). Philippines - Cebu Longitudinal Health and Nutrition Survey 2004-2006. Chapel Hill, United States: Carolina Population Center, University of North Carolina at Chapel Hill.

Carriere C, Langevin C, Deti EK, Barberger-Gateau P, Maurice S, Thibault H. Stabilization of overweight prevalence and improvement of dietary habits in French children between 2004 and 2008. Public Health Nutr. 2015; 18(10): 1883–9.

Carter AO, Saadi HF, Reed RL, Dunn EV. Assessment of obesity, lifestyle, and reproductive health needs of female citizens of Al Ain, United Arab Emirates. J Health Popul Nutr. 2004; 22(1): 75-83.

CBG Health Research Ltd., Ministry of Health (New Zealand), University of Otago (New Zealand). New Zealand Adult Nutrition Survey 2008-2009.

CBG Health Research Ltd., Ministry of Health (New Zealand), University of Wollongong. New Zealand Health Survey 2011-2012. Wellington, New Zealand: Ministry of Health (New Zealand).

Celi F, Bini V, De Giorgi G, Molinari D, Faraoni F, Di Stefano G, Bacosi ML, Berioli MG, Contessa G, Falorni A. Epidemiology of overweight and obesity among school children and adolescents in three provinces of central Italy, 1993-2001: study of potential influencing variables. Eur J Clin Nutr. 2003; 57(9): 1045-51.

Census Bureau (Chad), Macro International, Inc, National Institute of Statistical, Economic and Demographic Studies (Chad). Chad Demographic and Health Survey 1996-1997. Calverton, United States: Macro International, Inc.

Center for Human Resource Research, Ohio State University, Korea Labor Institute (South Korea), RAND Corporation, Statistics Netherlands. Korea, South Longitudinal Study of Ageing 2006. Seoul, South Korea: Korea Labor Institute (South Korea).

Center for Population and Policy Studies, Gadjah Mada University (Indonesia), RAND Corporation, SurveyMETER. Indonesia Family Life Survey 2007-2008. Santa Monica, United States: RAND Corporation.

Center for Population and Policy Studies, Gadjah Mada University (Indonesia), RAND Corporation. Indonesia Family Life Survey 2000. Santa Monica, United States: RAND Corporation.

Center for Research and Teaching in Economics (CIDE) (Mexico), National Institute of Perinatology (Mexico), National Institute of Statistics and Geography (Mexico), Universidad Iberoamericana. Mexico Family Life Survey 2002.

Center for Research in Human Development (CRDH), Cheikh Anta Diop University, Hospital Aristide Le Dantec, ICF Macro, National Agency of Statistics and Demography (Senegal). Senegal Demographic and Health Survey 2010-2011. Calverton, United States: ICF Macro.

Center for Scientific and Technological Information, Oswaldo Cruz Foundation and World Health Organization (WHO). Brazil World Health Survey 2003. Geneva, Switzerland: World Health Organization (WHO), 2005.

Center for Social and Demographic Studies (CESDEM), ICF International, Ministry of Public Health and Social Assistance (Dominican Republic). Dominican Republic Demographic and Health Survey 2013. Fairfax, United States: ICF International, 2014.

Center for Social and Demographic Studies (CESDEM), Macro International, Inc, National Planning Office (Dominican Republic), Profamilia. Dominican Republic Demographic and Health Survey 1996. Calverton, United States: Macro International, Inc.

Center for Social and Demographic Studies (CESDEM), Macro International, Inc. Dominican Republic Demographic and Health Survey 2002. Calverton, United States: Macro International, Inc.

Center for Social and Demographic Studies (CESDEM), Macro International, Inc. Dominican Republic Demographic and Health Survey 2007. Calverton, United States: Macro International, Inc.

Center for Social and Demographic Studies (CESDEM), United Nations Children's Fund (UNICEF). Dominican Republic Multiple Indicator Cluster Survey 2000. New York, United States: United Nations Children's Fund (UNICEF).

Center for Studies of Population and Social Development (CEPAR) (Ecuador) and Division of Reproductive Health-Centers for Disease Control and Prevention (CDC). (2005) Ecuador Reproductive Health Survey 2004. Quito, Ecuador: CEPAR.

Center for the Study of State and Society (CEDES), Ministry of Health and Environment (Argentina), National Institute of Statistics and Censuses (Argentina). Argentina National Survey of Risk Factors 2005.

CentERdata. Netherlands Dutch National Bank Household Survey 1995. Tillburg, Netherlands: CentERdata.

CentERdata. Netherlands Dutch National Bank Household Survey 1996. Tillburg, Netherlands: CentERdata.

CentERdata. Netherlands Dutch National Bank Household Survey 1997. Tillburg, Netherlands: CentERdata.

CentERdata. Netherlands Dutch National Bank Household Survey 1998. Tillburg, Netherlands: CentERdata.

CentERdata. Netherlands Dutch National Bank Household Survey 1999. Tillburg, Netherlands: CentERdata.

CentERdata. Netherlands Dutch National Bank Household Survey 2000. Tillburg, Netherlands: CentERdata.

CentERdata. Netherlands Dutch National Bank Household Survey 2001. Tillburg, Netherlands: CentERdata.

CentERdata. Netherlands Dutch National Bank Household Survey 2002. Tillburg, Netherlands: CentERdata.

CentERdata. Netherlands Dutch National Bank Household Survey 2003. Tillburg, Netherlands: CentERdata.

CentERdata. Netherlands Dutch National Bank Household Survey 2004. Tillburg, Netherlands: CentERdata.

CentERdata. Netherlands Dutch National Bank Household Survey 2005. Tillburg, Netherlands: CentERdata.

CentERdata. Netherlands Dutch National Bank Household Survey 2006. Tillburg, Netherlands: CentERdata.

CentERdata. Netherlands Dutch National Bank Household Survey 2007. Tillburg, Netherlands: CentERdata.

CentERdata. Netherlands Dutch National Bank Household Survey 2008. Tillburg, Netherlands: CentERdata.

CentERdata. Netherlands Dutch National Bank Household Survey 2009. Tillburg, Netherlands: CentERdata.

CentERdata. Netherlands Dutch National Bank Household Survey 2010. Tilburg, Netherlands: CentERdata.

CentERdata. Netherlands Dutch National Bank Household Survey 2011. Tilburg, Netherlands: CentERdata.

CentERdata. Netherlands Dutch National Bank Household Survey 2012. Tilburg, Netherlands: CentERdata.

CentERdata. Netherlands Dutch National Bank Household Survey 2013. Tilburg, Netherlands: CentERdata.

CentERdata. Netherlands Dutch National Bank Household Survey 2014.

Centers for Disease Control and Prevention (CDC), Central Bureau of Statistics (Kenya), Macro International, Inc, Ministry of Health (Kenya), National Council for Population and Development (Kenya). Kenya Demographic and Health Survey 2003. Calverton, United States: Macro International, Inc.

Centers for Disease Control and Prevention (CDC), Department of Health (Papua New Guinea), United Nations Children's Fund (UNICEF), University of Papua New Guinea. Papua New Guinea National Nutrition Survey 2005.

Centers for Disease Control and Prevention (CDC), Ghana Statistical Service, Government of Japan, Ministry of Health (Ghana), Navrongo Health Research Centre, USAID, United Nations Children's Fund (UNICEF), United Nations Population Fund (UNFPA). Ghana Multiple Indicator Cluster Survey 2011. New York, United States: United Nations Children's Fund (UNICEF), 2013.

Centers for Disease Control and Prevention (CDC), Haiti Child Health Institute (CHI), Haitian Institute of Statistics and Informatics, Macro International, Inc. Haiti Demographic and Health Survey 2012. Fairfax, United States: ICF International, 2013.

Centers for Disease Control and Prevention (CDC), Institute of Nutrition of Central America and Panama, Pan American Health Organization (PAHO). El Salvador - Santa Tecla Diabetes, Hypertension, and Chronic Disease Risk Factors Survey 2004.

Centers for Disease Control and Prevention (CDC), Institute of Public Health (Macedonia), Joint United Nations Program on HIV/AIDS (UNAIDS), United Nations Children's Fund (UNICEF), United Nations Educational, Scientific and Cultural Organization (UNESCO), World Health Organization (WHO). Macedonia Global School-Based Student Health Survey 2007. Geneva, Switzerland: World Health Organization (WHO).

Centers for Disease Control and Prevention (CDC), Joint United Nations Program on HIV/AIDS (UNAIDS), Ministry of Education (Thailand), Ministry of Public Health (Thailand), United Nations Children's Fund (UNICEF), United Nations Educational, Scientific and Cultural Organization (UNESCO), World Health Organization (WHO). Thailand Global School-Based Student Health Survey 2008. Geneva, Switzerland: World Health Organization (WHO).

Centers for Disease Control and Prevention (CDC), Joint United Nations Program on HIV/AIDS (UNAIDS), Ministry of Education and Sports (Uganda), Ministry of Health (Uganda), United Nations Children's Fund (UNICEF), United Nations Educational, Scientific and Cultural Organization (UNESCO), World Health Organization (WHO). Uganda Global School-Based Student Health Survey 2003 . Geneva, Switzerland: World Health Organization (WHO).

Centers for Disease Control and Prevention (CDC), Joint United Nations Program on HIV/AIDS (UNAIDS), Ministry of Education, Women Affairs, and Culture (Tonga), Ministry of Health (Tonga), United Nations Children's Fund (UNICEF), United Nations Educational, Scientific and Cultural Organization (UNESCO), World Health Organization (WHO). Tonga Global School-Based Student Health Survey 2010. Geneva, Switzerland: World Health Organization (WHO).

Centers for Disease Control and Prevention (CDC), Joint United Nations Program on HIV/AIDS (UNAIDS), Ministry of Health (Djibouti), Ministry of National and Higher Education (Djibouti), United Nations Children's Fund (UNICEF), United Nations Educational, Scientific and Cultural Organization (UNESCO), World Health Organization (WHO). Djibouti Global School-Based Student Health Survey 2007. Geneva, Switzerland: World Health Organization (WHO).

Centers for Disease Control and Prevention (CDC), Joint United Nations Program on HIV/AIDS (UNAIDS), Ministry of Health (Fiji), United Nations Children's Fund (UNICEF), United Nations Educational, Scientific and Cultural Organization (UNESCO), World Health Organization (WHO). Fiji Global School-Based Student Health Survey 2010. Geneva, Switzerland: World Health Organization (WHO).

Centers for Disease Control and Prevention (CDC), Joint United Nations Program on HIV/AIDS (UNAIDS), Ministry of Health (Morocco), United Nations Children's Fund (UNICEF), United Nations Educational, Scientific and Cultural Organization (UNESCO), World Health Organization (WHO). Morocco Global School-Based Student Health Survey 2010-2011. Geneva, Switzerland: World Health Organization (WHO), 2013.

Centers for Disease Control and Prevention (CDC), Joint United Nations Program on HIV/AIDS (UNAIDS), Ministry of Health (Sri Lanka), United Nations Children's Fund (UNICEF), United Nations Educational, Scientific and Cultural Organization (UNESCO), World Health Organization (WHO). Sri Lanka Global School-Based Student Health Survey 2008 . Geneva, Switzerland: World Health Organization (WHO).

Centers for Disease Control and Prevention (CDC), Joint United Nations Program on HIV/AIDS (UNAIDS), Ministry of Public Health (Benin), United Nations Children's Fund (UNICEF), United Nations Educational, Scientific and Cultural Organization (UNESCO), World Health Organization (WHO). Benin Global School-Based Student Health Survey 2009. Geneva, Switzerland: World Health Organization (WHO).

Centers for Disease Control and Prevention (CDC), Joint United Nations Program on HIV/AIDS (UNAIDS), Pakistan Medical Research Council, United Nations Children's Fund (UNICEF), United Nations Educational, Scientific and Cultural Organization (UNESCO), World Health Organization (WHO). Pakistan Global School-Based Student Health Survey 2009. Geneva, Switzerland: World Health Organization (WHO).

Centers for Disease Control and Prevention (CDC), Joint United Nations Program on HIV/AIDS (UNAIDS), United Nations Children's Fund (UNICEF), United Nations Educational, Scientific and Cultural Organization (UNESCO), World Health Organization (WHO). Algeria Global School-Based Student Health Survey 2010. Geneva, Switzerland: World Health Organization (WHO).

Centers for Disease Control and Prevention (CDC), Joint United Nations Program on HIV/AIDS (UNAIDS), United Nations Children's Fund (UNICEF), United Nations Educational, Scientific and Cultural Organization (UNESCO), World Health Organization (WHO). Costa Rica Global School-Based Student Health Survey 2009. Geneva, Switzerland: World Health Organization (WHO).

Centers for Disease Control and Prevention (CDC), Joint United Nations Program on HIV/AIDS (UNAIDS), United Nations Children's Fund (UNICEF), United Nations Educational, Scientific and Cultural Organization (UNESCO), World Health Organization (WHO). Myanmar Global School-Based Student Health Survey 2007. Geneva, Switzerland: World Health Organization (WHO).

Centers for Disease Control and Prevention (CDC), Joint United Nations Program on HIV/AIDS (UNAIDS), United Nations Children's Fund (UNICEF), United Nations Educational, Scientific and Cultural Organization (UNESCO), World Health Organization (WHO). Suriname Global School-Based Student Health Survey 2009. Geneva, Switzerland: World Health Organization (WHO).

Centers for Disease Control and Prevention (CDC), Ministry of Education (Argentina), Ministry of Health (Argentina), Pan American Health Organization (PAHO), Public Opinion, Services, and Markets (OPSM), World Health Organization (WHO). Argentina Global School-Based Student Health Survey 2007.

Centers for Disease Control and Prevention (CDC), Ministry of Education (Jordan), Ministry of Health (Jordan), World Health Organization (WHO). Jordan Global School-Based Student Health Survey 2004. Geneva, Switzerland: World Health Organization (WHO).

Centers for Disease Control and Prevention (CDC), Ministry of Education (Syria), Ministry of Health (Syria), World Health Organization (WHO). Syria Global School-Based Student Health Survey 2010-2011. Geneva, Switzerland: World Health Organization (WHO), 2013.

Centers for Disease Control and Prevention (CDC), Ministry of Health (Belize), Pan American Health Organization (PAHO). Belize Diabetes, Hypertension, and Noncommunicable Disease Risk Factors Survey 2005-2006.

Centers for Disease Control and Prevention (CDC), Ministry of Health (Guyana), World Health Organization (WHO). Guyana Global School-Based Student Health Survey 2010.

Centers for Disease Control and Prevention (CDC), Ministry of Health (Jordan), World Health Organization (WHO). Jordan STEPS Noncommunicable Disease Risk Factors Survey 2007.

Centers for Disease Control and Prevention (CDC), Ministry of Health (Nicaragua), Pan American Health Organization (PAHO). Nicaragua - Managua Diabetes, Hypertension, and Non-Communicable Disease Risk Factors Survey 2003.

Centers for Disease Control and Prevention (CDC), Ministry of Health (Peru), Pan American Health Organization (PAHO). Peru Global School Based Student Health Survey 2010. Geneva, Switzerland: World Health Organization (WHO).

Centers for Disease Control and Prevention (CDC), Ministry of Health (Rwanda), National Institute of Statistics of Rwanda, Rwanda Biomedical Center, World Health Organization (WHO). Rwanda STEPS Noncommunicable Disease Risk Factor Survey 2012-2013.

Centers for Disease Control and Prevention (CDC), Ministry of Higher Education (Egypt), World Health Organization (WHO). Egypt Global School-Based Student Health Survey 2006. Geneva, Switzerland: World Health Organization (WHO).

Centers for Disease Control and Prevention (CDC), Secretariat of Health and Environment (Libya), World Health Organization (WHO). Libya Global School-Based Student Health Survey 2007. Geneva, Switzerland: World Health Organization (WHO).

Centers for Disease Control and Prevention (CDC), World Health Organization (WHO). Ghana Global School-Based Student Health Survey 2007. Geneva, Switzerland: World Health Organization (WHO).

Centers for Disease Control and Prevention (CDC), World Health Organization (WHO). Jamaica Global School-Based Student Health Survey 2010. Geneva, Switzerland: World Health Organization (WHO).

Centers for Disease Control and Prevention (CDC), World Health Organization (WHO). Philippines Global School-Based Student Health Survey 2007. Geneva, Switzerland: World Health Organization (WHO).

Centers for Disease Control and Prevention (CDC). United States Behavioral Risk Factor Surveillance System 1984. Atlanta, Georgia: CDC, US Department of Health and Human Services

Centers for Disease Control and Prevention (CDC). United States Behavioral Risk Factor Surveillance System 1985. Atlanta, Georgia: CDC, US Department of Health and Human Services.

Centers for Disease Control and Prevention (CDC). United States Behavioral Risk Factor Surveillance System 1989. Atlanta, Georgia: CDC, US Department of Health and Human Services.

Centers for Disease Control and Prevention (CDC). United States Behavioral Risk Factor Surveillance System 1990. Atlanta, Georgia: CDC, US Department of Health and Human Services.

Centers for Disease Control and Prevention (CDC). United States Behavioral Risk Factor Surveillance System 1991. Atlanta, Georgia: CDC, US Department of Health and Human Services.

Centers for Disease Control and Prevention (CDC). United States Behavioral Risk Factor Surveillance System 1992. Atlanta, Georgia: CDC, US Department of Health and Human Services.

Centers for Disease Control and Prevention (CDC). United States Behavioral Risk Factor Surveillance System 1993. Atlanta, Georgia: CDC, US Department of Health and Human Services.

Centers for Disease Control and Prevention (CDC). United States Behavioral Risk Factor Surveillance System 1994. Atlanta, Georgia: CDC, US Department of Health and Human Services.

Centers for Disease Control and Prevention (CDC). United States Behavioral Risk Factor Surveillance System 1995. Atlanta, Georgia: CDC, US Department of Health and Human Services.

Centers for Disease Control and Prevention (CDC). United States Behavioral Risk Factor Surveillance System 1996. Atlanta, Georgia: CDC, US Department of Health and Human Services.

Centers for Disease Control and Prevention (CDC). United States Behavioral Risk Factor Surveillance System 1997. Atlanta, Georgia: CDC, US Department of Health and Human Services.

Centers for Disease Control and Prevention (CDC). United States Behavioral Risk Factor Surveillance System 1998. Atlanta, Georgia: CDC, US Department of Health and Human Services.

Centers for Disease Control and Prevention (CDC). United States Behavioral Risk Factor Surveillance System 2000. Atlanta, Georgia: CDC, US Department of Health and Human Services.

Centers for Disease Control and Prevention (CDC). United States Behavioral Risk Factor Surveillance System 2001. Atlanta, Georgia: CDC, US Department of Health and Human Services.

Centers for Disease Control and Prevention (CDC). United States Behavioral Risk Factor Surveillance System 2002. Atlanta, Georgia: CDC, US Department of Health and Human Services.

Centers for Disease Control and Prevention (CDC). United States Behavioral Risk Factor Surveillance System 2003. Atlanta, Georgia: CDC, US Department of Health and Human Services.

Centers for Disease Control and Prevention (CDC). United States Behavioral Risk Factor Surveillance System 2004. Atlanta, Georgia: CDC, US Department of Health and Human Services.

Centers for Disease Control and Prevention (CDC). United States Behavioral Risk Factor Surveillance System 2005. Atlanta, Georgia: CDC, US Department of Health and Human Services.

Centers for Disease Control and Prevention (CDC). United States Behavioral Risk Factor Surveillance System 2006. Atlanta, Georgia: CDC, US Department of Health and Human Services.

Centers for Disease Control and Prevention (CDC). United States Behavioral Risk Factor Surveillance System 2007. Atlanta, Georgia: CDC, US Department of Health and Human Services.

Centers for Disease Control and Prevention (CDC). United States Behavioral Risk Factor Surveillance System 2008. Atlanta, Georgia: CDC, US Department of Health and Human Services.

Centers for Disease Control and Prevention (CDC). United States Behavioral Risk Factor Surveillance System 2009. Atlanta, Georgia: CDC, US Department of Health and Human Services.

Centers for Disease Control and Prevention (CDC). United States Behavioral Risk Factor Surveillance System 2010. Atlanta, United States: Centers for Disease Control and Prevention (CDC).

Centers for Disease Control and Prevention (CDC). United States Behavioral Risk Factor Surveillance System 2011. Atlanta, Georgia: CDC, US Department of Health and Human Services.

Centers for Disease Control and Prevention (CDC). United States Behavioral Risk Factor Surveillance System 2012. Atlanta, Georgia: CDC, US Department of Health and Human Services, 2013.

Centers for Disease Control and Prevention (CDC). United States Behavioral Risk Factor Surveillance System 2013. Atlanta, United States: Centers for Disease Control and Prevention (CDC), 2014.

Centers for Disease Control and Prevention (CDC). United States Behavioral Risk Factor Surveillance System 2014. Atlanta, United States: Centers for Disease Control and Prevention (CDC), 2015.

Centers for Disease Control and Prevention (CDC). United States Behavioral Risk Factor Surveillance System 1986. Atlanta, Georgia: CDC, US Department of Health and Human Services.

Centers for Disease Control and Prevention (CDC). United States Behavioral Risk Factor Surveillance System 1987. Atlanta, Georgia: CDC, US Department of Health and Human Services.

Centers for Disease Control and Prevention (CDC). United States Behavioral Risk Factor Surveillance System 1999. Atlanta, Georgia: CDC, US Department of Health and Human Services.

Centers for Disease Control and Prevention (CDC). United States Behavioral Risk Factor Surveillance System; 1988. Atlanta, Georgia: CDC, US Department of Health and Human Services.

Centers for Disease Control and Prevention (CDC). United States National Youth Risk Behavior Survey 1999. Atlanta, United States: Centers for Disease Control and Prevention (CDC), 2000.

Centers for Disease Control and Prevention (CDC). United States National Youth Risk Behavior Survey 2001. Atlanta, United States: Centers for Disease Control and Prevention (CDC), 2002.

Centers for Disease Control and Prevention (CDC). United States National Youth Risk Behavior Survey 2003. Atlanta, United States: Centers for Disease Control and Prevention (CDC), 2004.

Centers for Disease Control and Prevention (CDC). United States National Youth Risk Behavior Survey 2005. Atlanta, United States: Centers for Disease Control and Prevention (CDC), 2006.

Centers for Disease Control and Prevention (CDC). United States National Youth Risk Behavior Survey 2007. Atlanta, United States: Centers for Disease Control and Prevention (CDC), 2008.

Centers for Disease Control and Prevention (CDC). United States National Youth Risk Behavior Survey 2009. Atlanta, United States: Centers for Disease Control and Prevention (CDC), 2010.

Centers for Disease Control and Prevention (CDC). United States National Youth Risk Behavior Survey 2011. Atlanta, United States: Centers for Disease Control and Prevention (CDC), 2012.

Centers for Disease Control and Prevention (CDC). United States National Youth Risk Behavior Survey 2013. Atlanta, United States: Centers for Disease Control and Prevention (CDC), 2014.

Centers for Disease Control and Prevention (CDC). United States Pediatric Nutrition Surveillance System Table 20D 2002-2011.

Central Administration of Statistics (Lebanon), League of Arab States, Ministry of Social Affairs (Lebanon), Pan Arab Project for Family Health (PAPFAM). Lebanon Family Health Survey 2004.

Central African Institute of Statistics, Economic and Social Studies (ICASEES) (Central African Republic), ICF International. Central African Republic Multiple Indicator Cluster Survey 2010-2011. Fairfax, United States: ICF International, 2013.

Central Agency for Public Mobilization and Statistics (Egypt), Macro Systems, Inc.; Institute for Resource Development, National Population Council (Egypt). Egypt Demographic and Health Survey 1988-1989. Columbia, United States: Macro Systems, Inc.

Central Board of Health (Zambia), Central Statistical Office (Zambia), Macro International, Inc. Zambia Demographic and Health Survey 2001-2002. Calverton, United States: Macro International, Inc.

Central Bureau of Statistics (Kenya), Macro International, Inc, National Council for Population Development (NCPD). Kenya Demographic and Health Survey 1993. Calverton, United States: Macro International, Inc.

Central Bureau of Statistics (Kenya), Macro International, Inc, National Council for Population Development (NCPD). Kenya Demographic and Health Survey 1998. Calverton, United States: Macro International, Inc.

Central Bureau of Statistics (Kenya), United Nations Children's Fund (UNICEF). Kenya Multiple Indicator Cluster Survey 2000. New York, United States: United Nations Children's Fund (UNICEF).

Central Bureau of Statistics (Nepal), United Nations Children's Fund (UNICEF). Nepal Multiple Indicator Cluster Survey 2014. New York, United States: United Nations Children's Fund (UNICEF), 2015.

Central Bureau of Statistics (Sudan), Ministry of Health (South Sudan). Sudan - North Multiple Indicator Cluster Survey 2010. New York, United States: United Nations Children's Fund (UNICEF), 2015.

Central Bureau of the Census and Population Studies (Cameroon), Macro International, Inc. Cameroon Demographic and Health Survey 1998. Calverton, United States: Macro International, Inc.

Central Organization for Statistics and Information Technology (Iraq), Kurdistan Regional Statistics Office, Ministry of Health (Iraq), United Nations Children's Fund (UNICEF). Iraq Multiple Indicator Cluster Survey 2011. New York, United States: United Nations Children's Fund (UNICEF), 2013.

Central Organization for Statistics and Information Technology (Iraq), Ministry of Health (Iraq), World Health Organization (WHO). Iraq STEPS Noncommunicable Disease Risk Factors Survey 2006.

Central Statistical Agency (Ethiopia), ICF Macro, Ministry of Health (Ethiopia). Ethiopia Demographic and Health Survey 2010-2011. Calverton, United States: ICF Macro.

Central Statistical Agency (Ethiopia), ORC Macro. Ethiopia Demographic and Health Survey 2000. Calverton, United States: ORC Macro, 2001.

Central Statistical Office (Swaziland), Macro International, Inc. Swaziland Demographic and Health Survey 2006-2007. Calverton, United States: Macro International, Inc.

Central Statistical Office (Swaziland), United Nations Children's Fund (UNICEF). Swaziland Multiple Indicator Cluster Survey 2000. New York, United States: United Nations Children's Fund (UNICEF).

Central Statistical Office (Swaziland), United Nations Children's Fund (UNICEF). Swaziland Multiple Indicator Cluster Survey 2010. New York, United States: United Nations Children's Fund (UNICEF).

Central Statistical Office (Trinidad and Tobago), United Nations Children's Fund (UNICEF). Trinidad and Tobago Multiple Indicator Cluster Survey 2000. New York, United States: United Nations Children's Fund (UNICEF).

Central Statistical Office (Zambia), Food Security, Health and Nutrition Information System (Zambia), United Nations Children's Fund (UNICEF). Zambia Multiple Indicator Cluster Survey 1999. New York, United States: United Nations Children's Fund (UNICEF).

Central Statistical Office (Zambia), ICF International, Ministry of Health (Zambia), Tropical Diseases Research Centre, University Teaching Hospital (Zambia), University of Zambia. Zambia Demographic and Health Survey 2013-2014. Fairfax, United States: ICF International, 2015.

Central Statistical Office (Zambia), London School of Hygiene and Tropical Medicine. Zambia Living Conditions Monitoring Survey 1998. Lusaka, Zambia: Central Statistical Office (Zambia).

Central Statistical Office (Zambia), Macro International, Inc, Ministry of Health (Zambia). Zambia Demographic and Health Survey 1996-1997. Calverton, United States: Macro International, Inc.

Central Statistical Office (Zambia), Macro International, Inc, University of Zambia. Zambia Demographic and Health Survey 1992. Calverton, United States: Macro International, Inc.

Central Statistical Office (Zambia), Macro International, Inc. Zambia Demographic and Health Survey 2007. Calverton, United States: Macro International, Inc.

Central Statistical Office (Zambia). Zambia Living Conditions Monitoring Survey 1996. Lusaka, Zambia: Central Statistical Office (Zambia).

Central Statistical Office (Zambia). Zambia Living Conditions Monitoring Survey 2002-2003. Lusaka, Zambia: Central Statistical Office (Zambia).

Central Statistical Office (Zambia). Zambia Living Conditions Monitoring Survey 2004-2005. Lusaka, Zambia: Central Statistical Office (Zambia).

Central Statistical Office (Zambia). Zambia Living Conditions Monitoring Survey 2006.

Central Statistical Office (Zambia). Zambia Living Conditions Monitoring Survey 2010.

Central Statistical Office (Zimbabwe), Macro International, Inc. Zimbabwe Demographic and Health Survey 1994. Calverton, United States: Macro International, Inc.

Central Statistical Office (Zimbabwe), Macro International, Inc. Zimbabwe Demographic and Health Survey 1999. Calverton, United States: Macro International, Inc.

Central Statistical Office (Zimbabwe), Macro International, Inc. Zimbabwe Demographic and Health Survey 2005-2006. Calverton, United States: Macro International, Inc.

Central Statistical Office (Zimbabwe), Macro Systems, Inc.; Institute for Resource Development. Zimbabwe Demographic and Health Survey 1988-1989. Columbia, United States: Macro Systems, Inc.

Central Statistical Office (Zimbabwe). Zimbabwe Multiple Indicator Monitoring Survey 2009. New York, United States: United Nations Children's Fund (UNICEF).

Central Statistical Office of Saint Lucia, Government of Saint Lucia, Ministry of Social Transformation, Local Government and Community Empowerment (Saint Lucia), United Nations Children's Fund (UNICEF). Saint Lucia Multiple Indicator Cluster Survey 2012. New York, United States: United Nations Children's Fund (UNICEF), 2014.

Central Statistical Organization (Iraq), United Nations Children's Fund (UNICEF). Iraq Multiple Indicator Cluster Survey 2000. New York, United States: United Nations Children's Fund (UNICEF).

Central Statistical Organization (Yemen), ICF International, Ministry of Public Health and Population (Yemen). Yemen Demographic and Health Survey 2013. Fairfax, United States: ICF International, 2015.

Central Statistical Organization (Yemen), League of Arab States, Macro International, Inc. Yemen Demographic and Health Survey 1991-1992. Calverton, United States: Macro International, Inc.

Central Statistics Department (Gambia), United Nations Children's Fund (UNICEF). Gambia Multiple Indicator Cluster Survey 2000. New York, United States: United Nations Children's Fund (UNICEF).

Central Statistics Office (Botswana), United Nations Children's Fund (UNICEF). Botswana Multiple Indicator Cluster Survey 2000. New York, United States: United Nations Children's Fund (UNICEF), 2015.

Central Statistics Office (Namibia), Macro International, Inc, Ministry of Health and Social Services (Namibia). Namibia Demographic and Health Survey 1992. Calverton, United States: Macro International, Inc.

Central Statistics Office (Sierra Leone), United Nations Children's Fund (UNICEF). Sierra Leone Multiple Indicator Cluster Survey 2000. New York, United States: United Nations Children's Fund (UNICEF).

Central Statistics Organization (Afghanistan), United Nations Children's Fund (UNICEF). Afghanistan Multiple Indicator Cluster Survey 2010-2011. New York, United States: United Nations Children's Fund (UNICEF), 2013.

Centre for Disease Prevention and Control (Latvia), National Institute for Health and Welfare (Finland), Riga Stradiņš University. Latvia Health Behavior Among the Adult Population 2012.

Centre for Economic Reform and Transformation (CERT), University of Edinburgh, Economics Education and Research Consortium (EERC) (Ukraine), Institute for the Study of Labor (IZA), Kiev International Institute of Sociology, Rhine-Westphalia Institute for Economic Research (RWI). Ukraine Longitudinal Monitoring Survey 2003. Bonn, Germany: Institute for the Study of Labor (IZA).

Centre for Economic Reform and Transformation (CERT), University of Edinburgh, Economics Education and Research Consortium (EERC) (Ukraine), Institute for the Study of Labor (IZA), Kiev International Institute of Sociology, Rhine-Westphalia Institute for Economic Research (RWI). Ukraine Longitudinal Monitoring Survey 2004. Bonn, Germany: Institute for the Study of Labor (IZA).

Centre for Economic Reform and Transformation (CERT), University of Edinburgh, Economics Education and Research Consortium (EERC) (Ukraine), Institute for the Study of Labor (IZA), Kiev International Institute of Sociology, Rhine-Westphalia Institute for Economic Research (RWI). Ukraine Longitudinal Monitoring Survey 2007. Bonn, Germany: Institute for the Study of Labor (IZA).

Centre for Health Promotion Studies, National University of Ireland, Galway, Department of Public Health Medicine and Epidemiology, University College Dublin, Health Promotion Unit, Department of Health and Children (Ireland). Ireland Survey of Lifestyle Attitudes and Nutrition 2002. Dublin, Ireland: Health Promotion Unit, Department of Health and Children (Ireland).

Centre for Health Promotion Studies, National University of Ireland, Galway, Health Promotion Unit, Department of Health and Children (Ireland). Ireland Survey of Lifestyle Attitudes and Nutrition 1998. Dublin, Ireland: Health Promotion Unit, Department of Health and Children (Ireland).

Centre for International Health and Development, Institute of Child Health (CIHD), Food and Nutrition Security Analysis Unit (Somalia), Ministry of Health (Somalia), Ministry of Health and Labor (Somaliland), Puntland Ministry of Health (Somalia), United Nations Children's Fund (UNICEF), World Food Programme (WFP), World Health Organization (WHO). Somalia National Micronutrient and Anthropometric Survey 2009.

Centre for Physical Activity and Health, University of Sydney (Australia), Department of Health and Social Affairs (Micronesia), Fiji School of Medicine, Micronesia Human Resources Development Center, Pohnpei State Department of Health Services, World Health Organization (WHO). Micronesia - Pohnpei STEPS Noncommunicable Disease Risk Factors Survey 2002.

Centre for Research and Documentation in Health Economics (CREDES) (France). France Survey of Health and Welfare 2002.

Centre for the Study of African Economies (CSAE), Ghana Statistical Service. Ghana Urban Household Panel Survey 2006. Oxford, United Kingdom: Centre for the Study of African Economies (CSAE).

Centre for the Study of African Economies (CSAE), National Bureau of Statistics (Tanzania). Tanzania Urban Household Panel Survey 2003-2004. Oxford, United Kingdom: Centre for the Study of African Economies (CSAE).

Centre of Health Economics (Latvia), Lithuanian University of Health Sciences, National Institute for Health Development (Estonia), National Institute for Health and Welfare (Finland). Social Determinants of Health Behaviors Finbalt Health Monitor 1998-2008. Helsinki, Finland: National Institute for Health and Welfare (Finland), 2011.

Centre of Health Economics (Latvia), National Institute for Health and Welfare (Finland). Latvia Health Behavior Among the Adult Population 2010.

Centre of Health Economics (Latvia). Latvia Health Behavior Among the Adult Population 2008.

Chacar HR, Salameh P. Public schools adolescents' obesity and growth curves in Lebanon. J Med Liban. 2011; 59(2): 80-8.

Chamay Weber C, Haller DM, Narring F. Is there a role for primary care physicians' screening of excessive weight and eating concerns in adolescence?. J Pediatr. 2010; 157(1): 32-5.

Chang Y, Woo H-Y, Sung E, Kim CH, Kang H, Ju YS, Park KH. Prevalence of acanthosis nigricans in relation to anthropometric measures: community-based cross-sectional study in Korean pre-adolescent school children. Pediatr Int. 2008; 50(5): 667-73.

Chen LJ, Fox KR, Haase A, Wang JM. Obesity, fitness and health in Taiwanese children and adolescents. Eur J Clin Nutr. 2006; 60(12): 1367-75.

Chen L-J, Haase AM, Fox KR. Physical activity among adolescents in Taiwan. Asia Pac J Clin Nutr. 2007; 16(2): 354-61.

Chen Z, Yang G, Zhou M, Smith M, Offer A, Ma J, Wang L, Pan H, Whitlock G, Collins R, Niu S, Peto R. Body mass index and mortality from ischaemic heart disease in a lean population: 10 year prospective study of 220,000 adult men. Int J Epidemiol. 2006; 35(1): 141-50.

Chilima DM, Ismail SJ. Anthropometric characteristics of older people in rural Malawi. Eur J Clin Nutr. 1998; 52(9): 643-9.

China Center for Economic Research, Peking University. China Health and Retirement Longitudinal Study 2008. Beijing, China: China Center for Economic Research, Peking University.

China Center for Economic Research, Peking University. China Health and Retirement Longitudinal Study 2011-2012. Beijing, China: China Center for Economic Research, Peking University.

China Center for Economic Research, Peking University. China Health and Retirement Longitudinal Study Pilot Resurvey 2012. Beijing, China: China Center for Economic Research, Peking University.

Chinese Center for Disease Control and Prevention (CCDC). China Chronic Disease and Risk Factor Surveillance 2004 [Unpublished].

Chinese Center for Disease Control and Prevention (CCDC). China Chronic Disease and Risk Factor Surveillance 2007 [Unpublished].

Chinese Center for Disease Control and Prevention (CCDC). China Chronic Disease and Risk Factor Surveillance 2010 [Unpublished].

Chinese Center for Disease Control and Prevention (CCDC). China Chronic Disease and Risk Factor Surveillance 2013 [Unpublished].

Chiolero A, Madeleine G, Gabriel A, Burnier M, Paccaud F, Bovet P. Prevalence of elevated blood pressure and association with overweight in children of a rapidly developing country. J Hum Hypertens. 2007; 21(2): 120-7.

Chiu HC, Chang HY, Mau LW, Lee TK, Liu HW. Height, weight, and body mass index of elderly persons in Taiwan. J Gerontol A Biol Sci Med Sci. 2000; 55(11): M684-690.

Chronic Disease Research Centre, University of the West Indies, Ministry of Health (Barbados). Barbados Health of the Nation Survey 2011.

Chrzanowska M, Koziel S, Ulijaszek SJ. Changes in BMI and the prevalence of overweight and obesity in children and adolescents in Cracow, Poland, 1971-2000. Econ Hum Biol. 2007; 5(3): 370-8.

Chulalongkorn University, Institute of Population Studies (Thailand), Westinghouse; Institute for Resource Development. Thailand Demographic and Health Survey 1987. Columbia, United States: Westinghouse; Institute for Resource Development.

Chuuk Department of Health Services (Micronesia), Department of Health and Social Affairs (Micronesia), World Health Organization (WHO). Micronesia - Chuuk STEPS Noncommunicable Disease Risk Factors Survey 2006.

Cochrane T, Davey RC, de Castella FR. Influence of school community and fitness on prevalence of overweight in Australian school children. Prev Med. 2015; 81: 433–7.

College of Population Studies, Chulalongkorn University (Thailand), Institute for Population and Social Research, Mahidol University (Thailand), International Health Policy Program (Thailand), Ministry of Education (Thailand), Ministry of Public Health (Thailand), Ministry of Social Development and Human Security (MSDHS) (Thailand), National Health Security Office (Thailand), National Statistical Office (Thailand), Thai Health Promotion Foundation, United Nations Children's Fund (UNICEF). Thailand Multiple Indicator Cluster Survey 2012. New York, United States: United Nations Children's Fund (UNICEF), 2016.

Colombian Family Welfare Institute, Ministry of Social Protection (Colombia), National Institute of Health (Colombia), Profamilia. Colombia National Survey of the Nutritional Situation 2010.

Colombian Family Welfare Institute, National Institute of Health (Colombia), Pan American Health Organization (PAHO), Profamilia, Universidad de Antioquia (Colombia). Colombia National Survey of the Nutritional Situation 2004-2005.

Commission of the European Communities (2012): Eurobarometer 32 (Oct-Nov 1989). INRA, Brussels. GESIS Data Archive, Cologne. ZA1752 Data file Version 1.1.0, doi:10.4232/1.10890

Commonwealth Scientific and Industrial Research Organisation (CSIRO) National Research Preventative Health Flagship (Australia), Department of Health and Ageing (Australia), I-view Pty Ltd, University of South Australia. Australia National Children's Nutrition and Physical Activity Survey 2007.

Council of Health Ministers of GCC States, Ministry of Health (Kuwait), World Health Organization (WHO). Kuwait World Health Survey 2008-2010.

Cox, B.D., Health and Lifestyle Survey, 1984-1985 [computer file]. Colchester, Essex: UK Data Archive [distributor], October 1988. SN: 2218, http://dx.doi.org/10.5255/UKDA-SN-2218-1.

Cox, B.D., Health and Lifestyle Survey: Seven-Year Follow-Up, 1991-1992 [computer file]. Colchester, Essex: UK Data Archive [distributor], January 1995. SN: 3279 , http://dx.doi.org/10.5255/UKDA-SN-3279-1

Czajka K, Kołodziej M. PARENTAL PERCEPTION OF BODY WEIGHT IN PRESCHOOL CHILDREN AND AN ANALYSIS OF THE CONNECTION BETWEEN SELECTED PARENT-RELATED FACTORS AND THE ASSESSMENT OF THEIR CHILDREN'S WEIGHT. Dev Period Med. 2015; 19(3 Pt 2): 375–82.

Czech Republic Statistical Office, World Health Organization (WHO) Collaborating Center for Perinatal Medicine/Institute for the Care of Mother and Child, Prague, Centers for Disease Control and Prevention (CDC). Czech Republic Reproductive Health Survey 1993. Atlanta, United States: Centers for Disease Control and Prevention (CDC), 1995.

Dalla Vecchia CF, Susin C, Rösing CK, Oppermann RV, Albandar JM. Overweight and obesity as risk indicators for periodontitis in adults. J Periodontol. 2005; 76(10): 1721-8.

Daniel CR, Prabhakaran D, Kapur K, Graubard BI, Devasenapathy N, Ramakrishnan L, George PS, Shetty H, Ferrucci LM, Yurgalevitch S, Chatterjee N, Reddy KS, Rastogi T, Gupta PC, Mathew A, Sinha R. A cross-sectional investigation of regional patterns of diet and cardio-metabolic risk in India. Open Nutr J. 2011; 12.

Danish National Institute of Public Health. Denmark Health Interview Survey 1994.

Danish National Institute of Public Health. Denmark Health Interview Survey 2005.

Danish National Institute of Public Health. Denmark Health Interview Survey 2010.

Danish National Institute of Public Health. Denmark Health Interview Survey Database - Overweight and Underweight.

Dassanayake AS, Kasturiratne A, Rajindrajith S, Kalubowila U, Chakrawarthi S, De Silva AP, Makaya M, Mizoue T, Kato N, Wickremasinghe AR, de Silva HJ. Prevalence and risk factors for non-alcoholic fatty liver disease among adults in an urban Sri Lankan population. J Gastroenterol Hepatol. 2009; 24(7): 1284-8.

Data Analysis and Technical Assistance Limited (Bangladesh), International Food Policy Research Institute (IFPRI), USAID. Bangladesh Integrated Household Survey 2011-2012.

Davies CA, Vandelanotte C, Duncan MJ, van Uffelen JGZ. Associations of physical activity and screen-time on health related quality of life in adults. Prev Med. 2012; 55(1): 46-9.

de Abreu D, Guessous I, Gaspoz J-M, Marques-Vidal P. Compliance with the Swiss Society for Nutrition's dietary recommendations in the population of Geneva, Switzerland: a 10-year trend study (1999-2009). J Acad Nutr Diet. 2014; 114(5): 774–80.

De Andrade FB, de França Caldas A Jr, Kitoko PM. Relationship between oral health, nutrient intake and nutritional status in a sample of Brazilian elderly people. Gerodontology. 2009; 26(1): 40-5.

De Assis MAA, Rolland-Cachera MF, de Vasconcelos FAG, Bellisle F, Conde W, Calvo MCM, Luna MEP, Ireton MJ, Grosseman S. Central adiposity in Brazilian schoolchildren aged 7-10 years. Br J Nutr. 2007; 97(4): 799-805.

De Assis MAA, Rolland-Cachera MF, Grosseman S, de Vasconcelos FAG, Luna MEP, Calvo MCM, Barros MVG, Pires MMS, Bellisle F. Obesity, overweight and thinness in schoolchildren of the city of Florianópolis, Southern Brazil. Eur J Clin Nutr. 2005; 59(9): 1015-21.

de Freitas SN, Caiaffa WT, César CC, Faria VA, do Nascimento RM, Coelho GL. Nutritional Risk in the Urban Population of Ouro Preto, Southeastern Region of Brazil: The Ouro Preto Heart Study. Arq Bras Cardiol. 2007; 88(2): 191-9.

De Wit LM, van Straten A, van Herten M, Penninx BWJH, Cuijpers P. Depression and body mass index, a u-shaped association. BMC Public Health. 2009; 14.

De Zwaan M, Gruss B, Müller A, Philipsen A, Graap H, Martin A, Glaesmer H, Hilbert A. Association between obesity and adult attention-deficit/hyperactivity disorder in a German community-based sample. Obes Facts. 2011; 4(3): 204-11.

Decelis A, Jago R, Fox KR. Physical activity, screen time and obesity status in a nationally representative sample of Maltese youth with international comparisons. BMC Public Health. 2014; 14: 664.

Deepa M, Farooq S, Deepa R, Manjula D, Mohan V. Prevalence and significance of generalized and central body obesity in an urban Asian Indian population in Chennai, India (CURES: 47). Eur J Clin Nutr. 2009; 63(2): 259-67.

Demographic Research Unit (Togo), Department of Statistics (Togo), Macro Systems, Inc.; Institute for Resource Development, Ministry of Public Health, Social Affairs and the Status of Women (Togo). Togo Demographic and Health Survey 1988. Columbia, United States: Macro Systems, Inc.

Department of Applied Research for Development (Madagascar), Macro International, Inc. Madagascar Demographic and Health Survey 1992. Calverton, United States: Macro International, Inc.

Department of Biological Anthropology, University of Cambridge (UK), Institute of Epidemiology, Disease Control & Research (Bangladesh), National Centre for Control of Rheumatic Fever & Heart Diseases (Bangladesh), National Institute of Preventive and Social Medicine, University of Dhaka (Bangladesh), World Health Organization (WHO). Bangladesh STEPS Noncommunicable Disease Risk Factors Survey 2002.

Department of Census and Statistics (Sri Lanka), Westinghouse; Institute for Resource Development. Sri Lanka Demographic and Health Survey 1987. Columbia, United States: Westinghouse; Institute for Resource Development.

Department of Census and Statistics (Sri Lanka). Sri Lanka Demographic and Health Survey 2006-2007.

Department of Epidemiology and Public Health, University College London, MRC Social and Public Health Sciences Unit, University of Glasgow, Scottish Centre for Social Research (ScotCen). United Kingdom - Scottish Health Survey 2009 - Scottish Government.

Department of Epidemiology and Public Health, University College London, MRC Social and Public Health Sciences Unit, University of Glasgow, Scottish Centre for Social Research (ScotCen). United Kingdom - Scottish Health Survey 2010 - Scottish Government.

Department of Epidemiology and Public Health, University College London, MRC Social and Public Health Sciences Unit, University of Glasgow, Scottish Centre for Social Research (ScotCen). United Kingdom - Scottish Health Survey 2011 - Scottish Government.

Department of Epidemiology and Public Health, University College London, National Centre for Social Research (NatCen). United Kingdom Health Survey for England 2012-2013 - HSCIC.

Department of Epidemiology and Public Health, University College London, National Centre for Social Research (NatCen). United Kingdom Health Survey for England 2013-2014 - HSCIC.

Department of Epidemiology and Public Health, University College London, Scottish Centre for Social Research (ScotCen), University of Glasgow. United Kingdom - Scottish Health Survey 2012 - Scottish Government.

Department of Health (American Samoa), Monash University (Australia), World Health Organization (WHO). American Samoa STEPS Noncommunicable Disease Risk Factors Survey 2004.

Department of Health (Philippines), Department of Science and Technology (Philippines), Food and Nutrition Research Institute, Department of Science and Technology (Philippines), National Statistics Office (Philippines). Philippines Updating of Nutritional Status of Filipino Children and Other Population Groups 2011.

Department of Health (South Africa), Human Sciences Research Council, World Health Organization (WHO). South Africa WHO Study on Global AGEing and Adult Health 2007-2008. Geneva, Switzerland: World Health Organization (WHO).

Department of Health (South Africa), Macro International, Inc, South African Medical Research Council. South Africa Demographic and Health Survey 1998. Calverton, United States: Macro International, Inc.

Department of Health (South Africa), Macro International, Inc, South African Medical Research Council. South Africa Demographic and Health Survey 2003-2004.

Department of Health and Ageing (Australia), World Health Organization (WHO). Australia World Health Survey 2003. Geneva, Switzerland: World Health Organization (WHO), 2005.

Department of Public Health, Pontifical Catholic University of Chile, Institute of Nutrition and Food Technology (INTA), University of Chile, Ministry of Health (Chile), Pan American Health Organization (PAHO). Chile National Health Survey 2003.

Department of Statistics (Côte d'Ivoire), World Bank (WB). Côte d'Ivoire Living Standards Measurement Survey 1986-1987. Washington, DC, United States: World Bank (WB)

Department of Statistics (Côte d'Ivoire). Côte d'Ivoire Living Standards Measurement Survey 1987-1988. Washington, DC, United States: World Bank (WB).

Department of Statistics (Côte d'Ivoire). Côte d'Ivoire Living Standards Measurement Survey 1988-1989. Washington, DC, United States: World Bank (WB).

Department of Statistics (Jordan), ICF International. Jordan Demographic and Health Survey 2012. Fairfax, United States: ICF International, 2013.

Department of Statistics (Jordan), ICF Macro. Jordan Interim Demographic and Health Survey 2009. Calverton, United States: ICF Macro, 2010.

Department of Statistics (Jordan), Macro International, Inc. Jordan Demographic and Health Survey 1997. Calverton, United States: Macro International, Inc.

Department of Statistics (Jordan), Macro International, Inc. Jordan Demographic and Health Survey 2002. Calverton, United States: Macro International, Inc.

Department of Statistics (Jordan), Macro International, Inc. Jordan Demographic and Health Survey 2007. Calverton, United States: Macro International, Inc.

Department of Statistics (Jordan), Macro International, Inc.; Institute for Resource Development, Ministry of Planning (Jordan). Jordan Demographic and Health Survey 1990. Calverton, United States: Macro International, Inc.

Department of Statistics (Rwanda), United Nations Children's Fund (UNICEF). Rwanda Multiple Indicator Cluster Survey 2000. New York, United States: United Nations Children's Fund (UNICEF).

Department of Statistics (Senegal), Westinghouse; Institute for Resource Development. Senegal Demographic and Health Survey 1986. Columbia, United States: Westinghouse; Institute for Resource Development.

Department of Statistics (Togo), Macro International, Inc. Togo Demographic and Health Survey 1998. Calverton, United States: Macro International, Inc.

Department of Statistics and National Accounts (Niger), Macro International, Inc. Niger Demographic and Health Survey 1992. Calverton, United States: Macro International, Inc.

Department of Statistics and National Accounts (Niger), Macro International, Inc. Niger Demographic and Health Survey 2006. Calverton, United States: Macro International, Inc.

DeRoo LA, Vlastos AT, Mock P, Vlastos G, Morabia A. Comparison of women's breast cancer risk factors in Geneva, Switzerland and Shanghai, China. Prev Med. 2010; 51(6): 497-501.

Desai, Sonalde, Reeve Vanneman, and National Council of Applied Economic Research, New Delhi. India Human Development Survey (IHDS), 2005. ICPSR22626-v8. Ann Arbor, MI: Inter-university Consortium for Political and Social Research [distributor], 2010-06-29. doi:10.3886/ICPSR22626.v8.

Dhungana RR, Devkota S, Khanal MK, Gurung Y, Giri RK, Parajuli RK, Adhikari A, Joshi S, Hada B, Shayami A. Prevalence of cardiovascular health risk behaviors in a remote rural community of Sindhuli district, Nepal. BMC Cardiovasc Disord. 2014; 14: 92.

Diabetic and Cancer Society of Maldives (DCSM), Indhira Gandhi Memorial Hospital (Maldives), Ministry of Health (Maldives), World Health Organization (WHO). Maldives - Malé STEPS Noncommunicable Disease Risk Factors Survey 2004.

Dieu HTT, Dibley MJ, Sibbritt D, Hanh TTM. Prevalence of overweight and obesity in preschool children and associated socio-demographic factors in Ho Chi Minh City, Vietnam. Int J Pediatr Obes. 2007; 2(1): 40-50.

Dieu HTT, Dibley MJ, Sibbritt DW, Hanh TTM. Trends in overweight and obesity in pre-school children in urban areas of Ho Chi Minh City, Vietnam, from 2002 to 2005. Public Health Nutr. 2009; 12(5): 702-9.

Directorate for Economic Planning (Bosnia and Herzegovina), Federal Office of Statistics (Federation of Bosnia and Herzegovina), Institute of Statistics (Republic of Srpska), Ministry of Health (Federation of Bosnia and Herzegovina), Ministry of Health and Social Welfare (Republic of Srpska), Public Health Institute of Federation of Bosnia and Herzegovina, United Nations Children's Fund (UNICEF). Bosnia and Herzegovina Multiple Indicator Cluster Survey 2006. New York, United States: United Nations Children's Fund (UNICEF).

Directorate for Research, Studies, Evaluation, and Statistics (France), French Institute for Public Health Surveillance (INVS), Ministry of National Education, Higher Education, and Research (France), Ministry of Social Affairs and Health (France). France Health of Kindergarten Children 2005-2006.

Directorate for Research, Studies, Evaluation, and Statistics (France), French Institute for Public Health Surveillance (INVS), Ministry of National Education, Higher Education, and Research (France), Ministry of Social Affairs and Health (France). France Health of CM2 Students 2007-2008.

Directorate General of Statistics and National Accounting (Togo), United Nations Children's Fund (UNICEF). Togo Multiple Indicator Cluster Survey 2006. New York, United States: United Nations Children's Fund (UNICEF).

Directorate General of Statistics and National Accounting (Togo), United Nations Children's Fund (UNICEF). Togo Multiple Indicator Cluster Survey 2010. New York, United States: United Nations Children's Fund (UNICEF).

Directorate of Forecasting and Statistics, Ministry of the Economy, Finance and Planning (Senegal), Macro International, Inc. Senegal Demographic and Health Survey 1992-1993. Calverton, United States: Macro International, Inc.

Directorate of Forecasting and Statistics, Ministry of the Economy, Finance and Planning (Senegal), Ministry of Economics and Finance (Senegal), United Nations Children's Fund (UNICEF). Senegal Multiple Indicator Cluster Survey 2000. New York, United States: United Nations Children's Fund (UNICEF).

Directorate of Statistics and Census (Panama), Ministry of Economy and Finance (Panama), World Bank. Panama Living Standard Measurement Survey 2003. Washington DC, United States: World Bank.

Division of Reproductive Health, Centers for Disease Control and Prevention (CDC), National Institute for Development Information (Nicaragua). Nicaragua Reproductive Health Survey 2006-2007. Managua, Nicaragua: National Institute for Development Information (Nicaragua).

Division of Statistics and Economic Studies (Central African Republic), Macro International, Inc. Central African Republic Demographic and Health Survey 1994-1995. Calverton, United States: Macro International, Inc.

Division of Statistics and Economic Studies (Central African Republic), Ministry of Economy, Planning and International Cooperation (Central African Republic), United Nations Children's Fund (UNICEF). Central African Republic Multiple Indicator Cluster Survey 2000. New York, United States: United Nations Children's Fund (UNICEF).

Djärv T, Wikman A, Nordenstedt H, Johar A, Lagergren J, Lagergren P. Physical activity, obesity and gastroesophageal reflux disease in the general population. World J Gastroenterol. 2012; 18(28): 3710-4.

Do HTP, Geleijnse JM, Le MB, Kok FJ, Feskens EJM. National prevalence and associated risk factors of hypertension and prehypertension among Vietnamese adults. Am J Hypertens. 2015; 28(1): 89–97.

Do LM, Tran TK, Eriksson B, Petzold M, Nguyen CTK, Ascher H. Preschool overweight and obesity in urban and rural Vietnam: differences in prevalence and associated factors. Glob Health Action. 2015; 8..

Dong B, Wang Z, Song Y, Wang H-J, Ma J. Understanding trends in blood pressure and their associations with body mass index in Chinese children, from 1985 to 2010: a cross-sectional observational study. BMJ Open. 2015; 5(9).

Dorosty AR, Siassi F, Reilly JJ. Obesity in Iranian children. Arch Dis Child. 2002; 87(5): 388-391.

Doulougou B, Kouanda S, Rossier C, Soura A, Zunzunegui MV. Differences in hypertension between informal and formal areas of Ouagadougou, a sub-Saharan African city. BMC Public Health. 2014; 14: 893.

Dowse GK, Zimmet PZ, Gareeboo H, George K, Alberti MM, Tuomilehto J, Finch CF, Chitson P, Tulsidas H. Abdominal obesity and physical inactivity as risk factors for NIDDM and impaired glucose tolerance in Indian, Creole, and Chinese Mauritians. Diabetes Care. 1991; 14(4): 271-82.

Duboz P, Boetsch G, Gueye L, Macia E. Hypertension prevalence, awareness, treatment and control in Dakar (Senegal). J Hum Hypertens. 2014; 28(8): 489–93.

Duda RB, Anarfi JK, Adanu RMK, Seffah J, Darko R, Hill AG. The health of the “older women” in Accra, Ghana: results of the Women’s Health Study of Accra. J Cross Cult Gerontol. 2011; 26(3): 299–314.

Dugee O, Khor GL, Lye M-S, Luvsannyam L, Janchiv O, Jamyan B, Esa N. Association of major dietary patterns with obesity risk among Mongolian men and women. Asia Pac J Clin Nutr. 2009; 18(3): 433-40.

Dulskiene V, Kuciene R, Medzioniene J, Benetis R. Association between obesity and high blood pressure among Lithuanian adolescents: a cross-sectional study. Ital J Pediatr. 2014; 40: 102.

Duvigneaud N, Wijndaele K, Matton L, Deriemaeker P, Philippaerts R, Lefevre J, Thomis M, Duquet W. Socio-economic and lifestyle factors associated with overweight in Flemish adult men and women. BMC Public Health. 2007; 23.

Dyson PA, Anthony D, Fenton B, Matthews DR, Stevens DE, Community Interventions for Health Collaboration. High rates of child hypertension associated with obesity: a community survey in China, India and Mexico. Paediatr Int Child Health. 2014; 34(1): 43–9.

Eckhardt CL, Torheim LE, Monterrubio E, Barquera S, Ruel MT. The overlap of overweight and anaemia among women in three countries undergoing the nutrition transition. Eur J Clin Nutr. 2008; 62(2): 238-46.

Economic and Social Research Institute (ESRI) (Ireland), Health Promotion Unit, Department of Health and Children (Ireland), National University of Ireland, Galway, Royal College of Surgeons in Ireland (RCSI), University College Cork. Ireland Survey of Lifestyle Attitudes and Nutrition 2007. Dublin, Ireland: Health Promotion Unit, Department of Health and Children (Ireland).

Economic Development Initiatives (EDI), Muhimbili University of Health and Allied Sciences (Tanzania), Rockwool Foundation Research Unit. Tanzania - Kagera Living Standards Measurement Study 2010. Washington DC, United States: World Bank.

Economic Development Initiatives (EDI), World Bank (WB). Tanzania Core Welfare Indicators Questionnaire Survey 2006-2007. Bukoba, Tanzania: Economic Development Initiatives (EDI).

Egan KB, Ettinger AS, DeWan AT, Holford TR, Holmen TL, Bracken MB. General, but not abdominal, overweight increases odds of asthma among Norwegian adolescents: the Young-HUNT study. Acta Paediatr. 2014; 103(12): 1270–6.

Eiõsdóttir SP, Kristjánsson AL, Sigfúsdóttir ID, Garber CE, Allegrante JP. Trends in body mass index among Icelandic adolescents and young adults from 1992 to 2007. Int J Environ Res Public Health. 2010; 7(5): 2191-207.

Ekbäck G, Näslund I, Montgomery SM, Ordell S. Self-perceived oral health and obesity among 65 years old in two Swedish counties. Swed Dent J. 2010; 34(4): 207-15.

Ekblom O, Oddsson K, Ekblom B. Prevalence and regional differences in overweight in 2001 and trends in BMI distribution in Swedish children from 1987 to 2001. Scand J Public Health. 2004; 32(4): 257-63.

Ekelund U, Neovius M, Linné Y, Brage S, Wareham NJ, Rössner S. Associations between physical activity and fat mass in adolescents: the Stockholm Weight Development Study. Am J Clin Nutr. 2005; 81(2): 355-60.

El Salvador Demographic Association (ADS), Division of Reproductive Health-Centers for Disease Control and Prevention (CDC). El Salvador Family Planning/Maternal and Child Survey 1993. Atlanta, United States: Centers for Disease Control and Prevention (CDC).

El Salvador Demographic Association (ADS), Division of Reproductive Health-Centers for Disease Control and Prevention (CDC). El Salvador Reproductive Health Survey 1998. Atlanta, United States: Centers for Disease Control and Prevention (CDC).

El-Hazmi MAF, Warsy AS. A comparative study of prevalence of overweight and obesity in children in different provinces of Saudi Arabia. J Trop Pediatr. 2002; 48(3): 172-7.

El-Zanaty and Associates, Macro International, Inc, Ministry of Health and Population (Egypt), National Population Council (Egypt). Egypt Demographic and Health Survey 2005. Calverton, United States: Macro International, Inc.

El-Zanaty and Associates, Macro International, Inc, Ministry of Health and Population (Egypt), National Population Council (Egypt). Egypt Interim Demographic and Health Survey 2003. Calverton, United States: Macro International, Inc.

El-Zanaty and Associates, Macro International, Inc, Ministry of Health and Population (Egypt). Egypt Demographic and Health Survey 2008. Calverton, United States: Macro International, Inc, 2009.

Erem C, Yildiz R, Kavgaci H, Karahan C, Deger O, Can G, Telatar M. Prevalence of diabetes, obesity and hypertension in a Turkish population (Trabzon city). Diabetes Res Clin Pract. 2001; 54(3): 203-8.

Eschwege E, Basdevant A, Crine A, Moisan C, Charles M-A. Type 2 diabetes mellitus in France in 2012: results from the ObEpi survey. Diabetes Metab. 2015; 41(1): 55–61.

Esmaillzadeh A, Mirmiran P, Azizi F. Comparative evaluation of anthropometric measures to predict cardiovascular risk factors in Tehranian adult women. Public Health Nutr. 2006; 9(1): 61-9.

Esteghamati A, Meysamie A, Khalilzadeh O, Rashidi A, Haghazali M, Asgari F, Kamgar M, Gouya MM, Abbasi M. Third national Surveillance of Risk Factors of Non-Communicable Diseases (SuRFNCD-2007) in Iran: methods and results on prevalence of diabetes, hypertension, obesity, central obesity, and dyslipidemia. BMC Public Health. 2009; 167.

Estonian Centre for Health Education and Promotion. Estonia Health Behaviour Among the Estonian Adult Population Obesity Estimates 1990, 1992, 1994, 1996 [Unpublished].

European Commission (2012): Eurobarometer 44.3 (Feb-Apr 1996). INRA, Brussels. GESIS Data Archive, Cologne. ZA2829 Data file Version 1.0.1, doi:10.4232/1.10920

European Commission (2012): Eurobarometer 59.0 (Jan-Feb 2003). European Opinion Research Group (EORG), Brussels. GESIS Data Archive, Cologne. ZA3903 Data file Version 1.0.1, doi:10.4232/1.11352

European Commission (2012): Eurobarometer 64.3 (Nov-Dec 2005). TNS OPINION & SOCIAL, Brussels [Producer]. GESIS Data Archive, Cologne. ZA4415 Data file Version 1.0.1, doi:10.4232/1.10971

Eurostat, Ministry of Health (Bulgaria), National Statistical Institute of Bulgaria. Bulgaria European Health Interview Survey 2008.

Eurostat. Slovenia European Health Interview Survey 2007.

Evans DS, Glacken M, Goggin D. Childhood obesity: the extent of the problem among 6-year-old Irish national school children. Child Care Health Dev. 2011; 37(3): 352-9.

Ezeoma IT, Abioye-Kuteyi EA, Oladeji AO. Body build and blood pressure in a rural Nigerian community. Niger Postgrad Med J. 2001; 8(3): 140-4.

Fall CHD, Sachdev HS, Osmond C, Lakshmy R, Biswas SD, Prabhakaran D, Tandon N, Ramji S, Reddy KS, Barker DJP, Bhargava SK. Adult metabolic syndrome and impaired glucose tolerance are associated with different patterns of BMI gain during infancy: Data from the New Delhi Birth Cohort. Diabetes Care. 2008; 31(12): 2349-56.

Family Health Development Division, Ministry of Health (Malaysia). Malaysia Adult Nutrition Survey 2002-2003.

Family Planning Association (Trinidad and Tobago), Westinghouse; Institute for Resource Development. Trinidad and Tobago Demographic and Health Survey 1987. Columbia, United States: Westinghouse; Institute for Resource Development.

Farajian P, Panagiotakos DB, Risvas G, Malisova O, Zampelas A. Hierarchical analysis of dietary, lifestyle and family environment risk factors for childhood obesity: the GRECO study. Eur J Clin Nutr. 2014; 68(10): 1107–12.

Fazizi F, Esmaillzadeh A, Mirmiran FP. Obesity and cardiovascular disease risk factors in Tehran adults: a population-based study. East Mediterr Health J. 2004; 10(6): 887-97.

Federal Environment Agency (Germany), Federal Institute for Drugs and Medical Devices (Germany), Max Planck Institute of Psychiatry, Robert Koch Institute. Germany National Health Interview and Examination Survey 1997-1999. Berlin, Germany: Robert Koch Institute, 2000.

Federal Ministry of Food, Agriculture, and Consumer Protection (Germany), Max Rubner Institute. Germany National Nutrition Survey II 2005-2007. Max Rubner Institute.

Federal Ministry of Health (Austria). Austria Nutrition Report 2008.

Federal Ministry of Health (Nigeria), Macro Systems, Inc.; Institute for Resource Development, National Population Bureau (Nigeria). Nigeria - Ondo Special Demographic and Health Survey 1986-1987. Columbia, United States: Macro Systems, Inc.

Federal Ministry of Health (Sudan), Central Bureau of Statistics (Sudan), United Nations Children's Fund (UNICEF). Sudan Multiple Indicator Cluster Survey, North Sudan 2000. New York, United States: United Nations Children's Fund (UNICEF).

Federal Ministry of Health (Sudan), World Health Organization (WHO). Sudan - Khartoum STEPS Noncommunicable Disease Risk Factors Survey 2005-2006.

Federal Office of Statistics (Nigeria), Macro International, Inc.; Institute for Resource Development. Nigeria Demographic and Health Survey 1990. Calverton, United States: Macro International, Inc.

Federal Statistical Office (Switzerland), MIS Trend SA. Switzerland Health Survey 2007.

Federal Statistical Office (Switzerland). Switzerland Body Mass Index by Age, Sex, and Language Region. Neuchâtel, Switzerland: Federal Statistical Office (Switzerland).

Federal Statistical Office (Switzerland). Switzerland Health Survey 1993.

Federal Statistical Office (Switzerland). Switzerland Health Survey 1997.

Federal Statistical Office (Switzerland). Switzerland Health Survey 2002.

Ferreira RJ, Marques-Vidal PM. Prevalence and determinants of obesity in children in public schools of Sintra, Portugal. Obesity (Silver Spring). 2008; 16(2): 497-500.

Fezeu L, Minkoulou E, Balkau B, Kengne A-P, Awah P, Unwin N, Alberti GKMM, Mbanya J-C. Association between socioeconomic status and adiposity in urban Cameroon. Int J Epidemiol. 2006; 35(1): 105-11.

Field, J. et al. , National Survey of Sexual Attitudes and Lifestyles, 1990 [computer file]. Colchester, Essex: UK Data Archive [distributor], August 1995. SN: 3434, http://dx.doi.org/10.5255/UKDA-SN-3434-1

Fiji School of Medicine, Menzies Center for Population Health Research, University of Tasmania (Australia), Ministry of Health (Marshall Islands), World Health Organization (WHO). Marshall Islands STEPS Noncommunicable Disease Risk Factors Survey 2002.

Fiji School of Medicine, Ministry of Health and Medical Services (Solomon Islands), World Health Organization (WHO). Solomon Islands STEPS Noncommunicable Disease Risk Factors Survey 2005-2006.

Fisher L, Fraser J, Alexander C. Caregivers' inability to identify childhood adiposity: a cross-sectional survey of rural children and their caregivers' attitudes. Aust J Rural Health. 2006; 14(2): 56-61.

Flicker L, McCaul KA, Hankey GJ, Jamrozik K, Brown WJ, Byles JE, Almeida OP. Body mass index and survival in men and women aged 70 to 75. J Am Geriatr Soc. 2010; 58(2): 234-41.

Food and Nutrition Research Institute, Department of Science and Technology (Philippines). Philippines National Nutrition Survey 1998.

Food and Nutrition Research Institute, Department of Science and Technology (Philippines). Philippines National Nutrition Survey 2003.

Food and Nutrition Research Institute, Department of Science and Technology (Philippines). Philippines National Nutrition Survey 2013-2014.

Food and Nutrition Services, Ministry of Health (Israel), Israel Center for Disease Control (ICDC). Israel National Health and Nutrition Survey 1999-2001.

Fox K, Gordon-Strachan G, Johnson A, Ashley D. Jamaican youth health status 2005. West Indian Med J. 2009; 58(6): 533-8.

Frederiksen P, Jensen KE, Kjaer SK. Sociodemographic factors and risk-taking behaviour during adolescence and obesity among more than 40 000 Danes. Public Health Nutr. 2014; 17(1): 162–9.

Free University of Brussels (ULB), Ghent University, International Agency for Research on Cancer (IARC), Limburg University Center, Scientific Institute of Public Health (IPH) (Belgium), Statistics Belgium. Belgium Food Consumption Survey 2004.

French Institute for Public Health Surveillance (INVS), French National Health Insurance Fund for Salaried Workers (CNAMTS), National Conservatory of Arts and Trades (CNAM) (France), University of Paris 13. France National Nutrition and Health Survey 2006-2007.

French Institute for Public Health Surveillance (INVS), Ministry of National Education, Higher Education, and Research (France). France Health of Adolescents in Third Grade 2003-2004.

French Institute for Public Health Surveillance (INVS), Ministry of National Education, Higher Education, and Research (France). France Health of Children Aged Six through School Health Assessments 2000-2001.

French Institute for Public Health Surveillance (INVS), Ministry of National Education, Higher Education, and Research (France). France Health of Schoolchildren in CM2 2004-2005.

French Institute of Health and Medical Research (INSERM), Kantar Health, Roche (France). France National Survey of Overweight and Obesity 2009.

French Institute of Health and Medical Research (INSERM), Kantar Health, Roche (France). France National Survey of Overweight and Obesity 2012.

French Institute of Health and Medical Research (INSERM), Roche (France), TNS Sofres (France). France National Survey of Overweight and Obesity 2006.

French Institute of Health and Medical Research (INSERM), University of Victor Segalen Bordeaux (France). France Three-City Cohort Study 1999-2001.

Friis K, Lyng JI, Lasgaard M, Larsen FB. Energy drink consumption and the relation to socio-demographic factors and health behaviour among young adults in Denmark. A population-based study. Eur J Public Health. 2014; 24(5): 840–4.

Frye C, Heinrich J. Trends and predictors of overweight and obesity in East German children. Int J Obes Relat Metab Disord. 2003; 27(8): 963-9.

Fu Q, George LK. Socioeconomic determinants of childhood overweight and obesity in China: the long arm of institutional power. Sociol Health Illn. 2015; 37(6): 805–22.

Gajalakshmi V, Peto R, Kanimozhi VC, Whitlock G, Veeramani D. Cohort Profile: the Chennai prospective study of mortality among 500,000 adults in Tamil Nadu, South India. Int J Epidemiol. 2007; 36(6): 1190-5.

Gallus S, Odone A, Lugo A, Bosetti C, Colombo P, Zuccaro P, La Vecchia C. Overweight and obesity prevalence and determinants in Italy: an update to 2010. Eur J Nutr. 2013; 52(2): 677-85.

Gambia Bureau of Statistics (GBOS), ICF International, Ministry of Health and Social Welfare (Gambia). Gambia Demographic and Health Survey 2013. Fairfax, United States: ICF International, 2015.

Gambia Bureau of Statistics (GBOS), United Nations Children's Fund (UNICEF). Gambia Multiple Indicator Cluster Survey 2005-2006. New York, United States: United Nations Children's Fund (UNICEF).

Garcez MR, Pereira JL, Fontanelli M de M, Marchioni DML, Fisberg RM. Prevalence of dyslipidemia according to the nutritional status in a representative sample of Sao Paulo. Arq Bras Cardiol. 2014; 103(6): 476–84.

Gardner K, Bird J, Canning PM, Frizzell LM, Smith LM. Prevalence of overweight, obesity and underweight among 5-year-old children in Saint Lucia by three methods of classification and a comparison with historical rates. Child Care Health Dev. 2011; 37(1): 143-9.

Garmendia ML, Alonso FT, Kain J, Uauy R, Corvalan C. Alarming weight gain in women of a post-transitional country. Public Health Nutr. 2014; 17(3): 667–73.

Garmy P, Clausson EK, Nyberg P, Jakobsson U. Overweight and television and computer habits in Swedish school-age children and adolescents: a cross-sectional study. Nurs Health Sci. 2014; 16(2): 143–8.

Gaskin PS, Broome H, Alert C, Fraser H. Misperceptions, inactivity and maternal factors may drive obesity among Barbadian adolescents. Public Health Nutr. 2008; 11(1): 41-8.

General Bureau of Statistics (Suriname), Ministry of Planning and Development Cooperation (Suriname), Ministry of Social Affairs and Housing (Suriname), United Nations Children's Fund (UNICEF). Suriname Multiple Indicator Cluster Survey 2010. New York, United States: United Nations Children's Fund (UNICEF), 2013.

General Directorate of Statistics (Gabon), ICF International, Ministry of Economy, Employment and Sustainable Development (Gabon), Ministry of Health (Gabon). Gabon Demographic and Health Survey 2012. Fairfax, United States: ICF International, 2013.

General Directorate of Statistics and Economic Studies (Gabon), Macro International, Inc. Gabon Demographic and Health Survey 2000-2001. Calverton, United States: Macro International, Inc.

General Statistical Office (Suriname), United Nations Children's Fund (UNICEF). Suriname Multiple Indicator Cluster Survey 2006. New York, United States: United Nations Children's Fund (UNICEF).

General Statistics Office (Viet Nam), Ministry of Health (Viet Nam). Vietnam National Health Survey 2001-2002.

General Statistics Office (Viet Nam), Ministry of Health (Vietnam), National Institute of Nutrition (Vietnam), United Nations Children's Fund (UNICEF). Vietnam General Nutrition Survey 2009-2010.

General Statistics Office (Viet Nam), United Nations Children's Fund (UNICEF). Vietnam Multiple Indicator Cluster Survey 2000. New York, United States: United Nations Children's Fund (UNICEF).

General Statistics Office (Viet Nam), United Nations Children's Fund (UNICEF). Vietnam Multiple Indicator Cluster Survey 2010-2011. New York, United States: United Nations Children's Fund (UNICEF).

Georgiadis G, Nassis GP. Prevalence of overweight and obesity in a national representative sample of Greek children and adolescents. Eur J Clin Nutr. 2007; 61(9): 1072-4.

Gerdin EW, Angbratt M, Aronsson K, Eriksson E, Johansson I. Dental caries and body mass index by socio-economic status in Swedish children. Community Dent Oral Epidemiol. 2008; 36(5): 459-65.

GfK, National Food Agency (Sweden). Sweden National Food Consumption Survey 2003 [Unpublished].

GfK, Netherlands Nutrition Centre Foundation, TNO Nutrition. Netherlands National Food Consumption Survey 1997-1998.

Ghaem Maralani H, Tai BC, Wong TY, Tai ES, Li J, Wang JJ, Mitchell P. The prognostic role of body mass index on mortality amongst the middle-aged and elderly: a competing risk analysis. Diabetes Res Clin Pract. 2014; 103(1): 42–50.

Ghana Health Service, Ghana Statistical Service, ICF International. Ghana Demographic and Health Survey 2014. Fairfax, United States: ICF International, 2015.

Ghana Health Service, World Health Organization (WHO). Ghana - Greater Accra Region STEPS Noncommunicable Disease Risk Factors Survey 2006.

Ghana Statistical Service, Macro International, Inc, Ministry of Health (Ghana). Ghana Demographic and Health Survey 2008. Calverton, United States: Macro International, Inc.

Ghana Statistical Service, Macro International, Inc. Ghana Demographic and Health Survey 1993-1994. Calverton, United States: Macro International, Inc.

Ghana Statistical Service, Macro International, Inc. Ghana Demographic and Health Survey 1998-1999. Calverton, United States: Macro International, Inc.

Ghana Statistical Service, Macro International, Inc. Ghana Demographic and Health Survey 2003. Calverton, United States: Macro International, Inc.

Ghana Statistical Service, Macro Systems, Inc.; Institute for Resource Development. Ghana Demographic and Health Survey 1988. Columbia, United States: Macro Systems, Inc.

Ghannem H, Khlifa K, Harrabi I, Ben Abdelaziz A, Gaha R. Study of cardiovascular disease risk factors among urban schoolchildren in Sousse, Tunisia. East Mediterr Health J. 2000; 6(5-6): 1046-54.

Ghannem H, Trabelsi L, Gaha R, Harrabi I, Essoussi AS. Study of cardiovascular disease risk factors among rural schoolchildren in Sousse, Tunisia. East Mediterr Health J. 2001; 7(4-5): 617-24.

Giannouli P, Zervas I, Armeni E, Koundi K, Spyropoulou A, Alexandrou A, Kazani A, Areti A, Creatsa M, Lambrinoudaki I. Determinants of quality of life in Greek middle-age women: a population survey. Maturitas. 2012; 71(2): 154-61.

Gill TK, Hill CL, Shanahan EM, Taylor AW, Appleton SL, Grant JF, Shi Z, Dal Grande E, Price K, Adams RJ. Vitamin D levels in an Australian population. BMC Public Health. 2014; 14: 1001.

Global Fund to Fight Aids Tuberculosis and Malaria (GFATM), ICF International, National Center for Endemic Diseases (CNE) (Sao Tome and Principe), National Institute of Statistics (Sao Tome and Principe), United Nations Children's Fund (UNICEF), United Nations Development Programme (UNDP). Sao Tome and Principe Multiple Indicator Cluster Survey 2014. New York, United States: United Nations Children's Fund (UNICEF), 2016.

Goldney RD, Dunn KI, Air TM, Dal Grande E, Taylor AW. Relationships between body mass index, mental health, and suicidal ideation: population perspective using two methods. Aust N Z J Psychiatry. 2009; 43(7): 652-8.

Gong Y, Zhang X, Zou H, Tian D, Qu Z, Zhang W, Chu Y. Debt and depression among the Dibao population in northwestern China. Int J Soc Psychiatry. 2014; 60(7): 637–45.

Goon DT, Toriola AL, Shaw BS. Screening for body-weight disorders in Nigerian children using contrasting definitions. Obes Rev. 2010; 11(7): 508-15.

Gordon-Larsen P, Wang H, Popkin BM. Overweight dynamics in Chinese children and adults. Obes Rev. 2014; 15 Suppl 1: 37–48.

Government of Malta. Malta Annual Reports of Government Departments 2009. 2010.

Government of Mongolia, National Statistical Office of Mongolia, United Nations Children's Fund (UNICEF), United Nations Population Fund (UNFPA). Mongolia Multiple Indicator Cluster Survey 2013. New York, United States: United Nations Children's Fund (UNICEF), 2016.

Government of Niger, Macro International, Inc, United Nations Children's Fund (UNICEF). Niger Multiple Indicator Cluster Survey 2000. New York, United States: United Nations Children's Fund (UNICEF).

Government of Portugal, World Health Organization Regional Office for Europe (EURO-WHO). Portugal Childhood Obesity Surveillance Initiative 2008.

Grammatikopoulou MG, Poulimeneas D, Gounitsioti IS, Gerothanasi K, Tsigga M, Kiranas E, ADONUT Study Group. Prevalence of simple and abdominal obesity in Greek adolescents: the ADONUT study. Clin Obes. 2014; 4(6): 303–8.

Grech V, Farrugia Sant'Angelo V. Body mass index estimation in a school-entry aged cohort in Malta. Int J Pediatr Obes. 2009; 4(2): 126-8.

Grøholt E-K, Stigum H, Nordhagen R. Overweight and obesity among adolescents in Norway: cultural and socio-economic differences. J Public Health (Oxf). 2008; 30(3): 258-65.

Gu D, Reynolds K, Wu X, Chen J, Duan X, Reynolds RF, Whelton PK, He J, InterASIA Collaborative Group. Prevalence of the metabolic syndrome and overweight among adults in China. Lancet. 2005; 365(9468): 1398-405.

Gualdi-Russo E, Albertini A, Argnani L, Celenza F, Nicolucci M, Toselli S. Weight status and body image perception in Italian children. J Hum Nutr Diet. 2008; 21(1): 39-45.

Guatemala Ministry of Health and Social Assistance, University of Valle and Division of Reproductive Health-Centers for Disease Control and Prevention (CDC). Guatemala Reproductive Health Survey 2008-2009. Atlanta, United States: Centers for Disease Control and Prevention (CDC).

Guatemala Ministry of Health and Social Assistance, University of Valle, Division of Reproductive Health-Centers for Disease Control and Prevention (CDC). (2003) Guatemala Reproductive Health Survey 2002. Atlanta, United States: Centers for Disease Control and Prevention (CDC).

Guimarães JMN, de Souza Lopes C, Baima J, Sichieri R. Depression symptoms and hypothyroidism in a population-based study of middle-aged Brazilian women. J Affect Disord. 2009; 117(1-2): 120-3.

Guo X, Li Z, Guo L, Zheng L, Yu S, Yang H, Zou L, Zhou Y, Zhang Y, Zhu L, Zhang Y, Sun Y. An update on overweight and obesity in rural Northeast China: from lifestyle risk factors to cardiometabolic comorbidities. BMC Public Health. 2014; 14: 1046.

Gupta A, Gupta R, Sarna M, Rastogi S, Gupta VP, Kothari K. Prevalence of diabetes, impaired fasting glucose and insulin resistance syndrome in an urban Indian population. Diabetes Res Clin Pract. 2003; 61(1): 69-76.

Gupta R, Deedwania PC, Sharma K, Gupta A, Guptha S, Achari V, Asirvatham AJ, Bhansali A, Gupta B, Gupta S, Jali MV, Mahanta TG, Maheshwari A, Saboo B, Singh J, Gupta R. Association of educational, occupational and socioeconomic status with cardiovascular risk factors in Asian Indians: a cross-sectional study. PLoS One. 2012; 7(8): e44098.

Gurzkowska B, Grajda A, Kulaga Z, Napieralska E, Litwin M. Distribution of body mass index categories among Polish children and adolescents from rural and urban areas. Med Wieku Rozwoj. 2011; 15(3): 250–7.

Gutiérrez-Fisac JL, López E, Banegas JR, Graciani A, Rodríguez-Artalejo F. Prevalence of Overweight and Obesity in Elderly People in Spain. Obes Res. 2004; 12(4): 710-5.

Haba-Rubio J, Marques-Vidal P, Andries D, Tobback N, Preisig M, Vollenweider P, Waeber G, Luca G, Tafti M, Heinzer R. Objective sleep structure and cardiovascular risk factors in the general population: the HypnoLaus Study. Sleep. 2015; 38(3): 391–400.

Haenle MM, Brockmann SO, Kron M, Bertling U, Mason RA, Steinbach G, Boehm BO, Koenig W, Kern P, Piechotowski I, Kratzer W, EMIL-Study group. Overweight, physical activity, tobacco and alcohol consumption in a cross-sectional random sample of German adults. BMC Public Health. 2006; 233.

Haiti Child Health Institute (CHI), Haitian Institute of Statistics and Informatics, Macro International, Inc. Haiti Demographic and Health Survey 2005-2006. Calverton, United States: Macro International, Inc.

Haiti Child Health Institute (CHI), Macro International, Inc. Haiti Demographic and Health Survey 1994-1995. Calverton, United States: Macro International, Inc.

Haiti Child Health Institute (CHI), Macro International, Inc. Haiti Demographic and Health Survey 2000. Calverton, United States: Macro International, Inc.

Haitian Institute of Statistics and Informatics, The Fafo Research Foundation. Haiti Living Condition Survey 2001, Haiti: Haitian Institute of Statistics and Informatics.

Hajian-Tilaki KO, Heidari B. Prevalence of obesity, central obesity and the associated factors in urban population aged 20-70 years, in the north of Iran: a population-based study and regression approach. Obes Rev. 2007; 8(1): 3-10.

Hamaideh SH, Al-Khateeb RY, Al-Rawashdeh AB. Overweight and obesity and their correlates among Jordanian adolescents. J Nurs Scholarsh. 2010; 42(4): 387-94.

Hansson LM, Rasmussen F. Predictors of 10-year-olds' obesity stereotypes: A population-based study. Int J Pediatr Obes. 2010; 5(1): 25-33.

Harris, Kathleen Mullan, and J. Richard Udry. National Longitudinal Study of Adolescent to Adult Health (Add Health), 1994-2008. ICPSR21600-v12. Ann Arbor, MI: Inter-university Consortium for Political and Social Research [distributor], 2013-03-08. doi:10.3886/ICPSR21600.v12

Hassing LB, Dahl AK, Pedersen NL, Johansson B. Overweight in midlife is related to lower cognitive function 30 years later: a prospective study with longitudinal assessments. Dement Geriatr Cogn Disord. 2010; 29(6): 543-52.

Hayashi R, Iwasaki M, Otani T, Wang N, Miyazaki H, Yoshiaki S, Aoki S, Koyama H, Suzuki S. Body mass index and mortality in a middle-aged Japanese cohort. J Epidemiol. 2005; 15(3): 70-7.

Health Canada, Heart and Stroke Foundation of Canada. Canada Heart Health Database 1986-1992.

Health Canada, Public Health Agency of Canada, Statistics Canada. Canada Health Measures Survey 2007-2009. Ottawa, Canada: Statistics Canada.

Health Canada, Public Health Agency of Canada, Statistics Canada. Canada Health Measures Survey 2009-2011. Ottawa, Canada: Statistics Canada.

Health Care International, World Health Organization (WHO). Egypt WHO Multi-country Survey Study on Health and Health System Responsiveness 2000-2001. Geneva, Switzerland: World Health Organization (WHO).

Health Measurement Research Group, University of Wisconsin. United States National Health Measurement Study 2005-2006. Data and Information Sciences Center, University of Wisconsin-Madison [distributor].

Health of Populations in Transition (HoPiT) Research Group (Cameroon), Ministry of Public Health (Cameroon), World Diabetes Foundation (WDF), World Health Organization (WHO). Cameroon STEPS Noncommunicable Disease Risk Factors Survey 2003.

Health Promotion Centre (Latvia). Latvia Health Behavior Among the Adult Population 2004.

Health Promotion Research Institute (Hungary), Hungarian Gallup Institute. Hungary National Population Health Survey 2000.

Health Protection Agency (Maldives), World Health Organization (WHO). Maldives STEPS Noncommunicable Disease Risk Factors Survey 2011 .

Heartfile, Ministry of Health (Pakistan), Statistics Division (Pakistan), World Health Organization (WHO). Pakistan Health Indicators 2007. Islamabad, Pakistan: Heartfile, 2007.

Heitzinger K, Montano SM, Hawes SE, Alarcon JO, Zunt JR. A community-based cluster randomized survey of noncommunicable disease and risk factors in a peri-urban shantytown in Lima, Peru. BMC Int Health Hum Rights. 2014; 14: 19.

Helseth S, Haraldstad K, Christophersen K-A. A cross-sectional study of Health Related Quality of Life and body mass index in a Norwegian school sample (8-18 years): a comparison of child and parent perspectives. Health Qual Life Outcomes. 2015; 13: 47.

Herman KM, Craig CL, Gauvin L, Katzmarzyk PT. Tracking of obesity and physical activity from childhood to adulthood: the Physical Activity Longitudinal Study. Int J Pediatr Obes. 2009; 4(4): 281-8.

Herman KM, Hopman WM, Craig CL. Are youth BMI and physical activity associated with better or worse than expected health-related quality of life in adulthood? The Physical Activity Longitudinal Study. Qual Life Res. 2010; 19(3): 339-49.

Hesketh K, Wake M, Waters E, Carlin J, Crawford D. Stability of body mass index in Australian children: a prospective cohort study across the middle childhood years. Public Health Nutr. 2004; 7(2): 303-9.

Hestetun I, Svendsen MV, Oellingrath IM. Associations between overweight, peer problems, and mental health in 12-13-year-old Norwegian children. Eur Child Adolesc Psychiatry. 2015; 24(3): 319–26.

Heude B, Lafay L, Borys JM, Thibult N, Lommez A, Romon M, Ducimetière P, Charles MA. Time trend in height, weight, and obesity prevalence in school children from Northern France, 1992-2000. Diabetes Metab. 2003; 29(3): 235-40.

Ho SC, Chen YM, Woo JL, Leung SS, Lam TH, Janus ED. Association between simple anthropometric indices and cardiovascular risk factors. Int J Obes Relat Metab Disord. 2001; 25(11): 1689-97.

Holden CA, McLachlan RI, Pitts M, Cumming R, Wittert G, Ehsani JP, de Kretser DM, Handelsman DJ. Determinants of male reproductive health disorders: the Men in Australia Telephone Survey (MATeS). BMC Public Health. 2010; 96.

Holdsworth M, Gartner A, Landais E, Maire B, Delpeuch F. Perceptions of healthy and desirable body size in urban Senegalese women. Int J Obes Relat Metab Disord. 2004; 28(12): 1561-8.

Honduras Family Planning Association (ASHONPLAFA), Ministry of Health (Honduras), and Division of Reproductive Health-Centers for Disease Control and Prevention (CDC). Honduras Reproductive Health Survey 2001. Tegucigalpa, Honduras: Honduras Family Planning Association (ASHONPLAFA).

Hong TK, Dibley MJ, Sibbritt D, Binh PNT, Trang NHHD, Hanh TTM. Overweight and obesity are rapidly emerging among adolescents in Ho Chi Minh City, Vietnam, 2002-2004. Int J Pediatr Obes. 2007; 2(4): 194-201.

Hopman WM, Berger C, Joseph L, Barr SI, Gao Y, Prior JC, Poliquin S, Towheed T, Anastassiades T. The association between body mass index and health-related quality of life: data from CaMos, a stratified population study. Qual Life Res. 2007; 16(10): 1595-603.

Hou X, Jia W, Bao Y, Lu H, Jiang S, Zuo Y, Gu H, Xiang K. Risk factors for overweight and obesity, and changes in body mass index of Chinese adults in Shanghai. BMC Public Health. 2008; 389.

Howe AS, Mandic S, Parnell WR, Skidmore PML. Attitudes to food differ between adolescent dieters and non-dieters from Otago, New Zealand, but overall food intake does not. Public Health Nutr. 2013; 16(1): 36-45.

Hu H-Y, Chou Y-J, Chou P, Chen L-K, Huang N. Association between obesity and injury among Taiwanese adults. Int J Obes (Lond). 2009; 33(8): 878-84.

Huerta JM, Tormo MJ, Egea-Caparrós JM, Ortolá-Devesa JB, Navarro C. Accuracy of self-reported diabetes, hypertension and hyperlipidemia in the adult Spanish population. DINO study findings. Rev Esp Cardiol. 2009; 62(2): 143-52.

Hulsegge G, Picavet HSJ, Blokstra A, Nooyens ACJ, Spijkerman AMW, van der Schouw YT, Smit HA, Verschuren WMM. Today's adult generations are less healthy than their predecessors: generation shifts in metabolic risk factors: the Doetinchem Cohort Study. Eur J Prev Cardiol. 2014; 21(9): 1134–44.

Human Nutrition Research, Medical Research Council, National Centre for Social Research (NatCen), Northern Ireland Statistics and Research Agency (NISRA), University College London Medical School. United Kingdom National Diet and Nutrition Survey 2008-2011 - Public Health England.

Human Sciences Research Council, South African Medical Research Council. South Africa National Health and Nutrition Examination Survey 2012.

Hurjui DM, Nita O, Graur LI, Mihalache L, Popescu DS, Hutanasu IC, Ungureanu D, Graur M. Non-alcoholic fatty liver disease is associated with cardiovascular risk factors of metabolic syndrome. Med Surg J Soc Phys Nat Iasi. 2012; 116(3): 692-9.

Hussain A, Vaaler S, Sayeed MA, Mahtab H, Ali SM, Khan AK. Type 2 diabetes and impaired fasting blood glucose in rural Bangladesh: a population-based study. Eur J Public Health. 2007; 17(3): 291-6.

Husseini A, Abdul-Rahim H, Awartani F, Giacaman R, Jervell J, Bjertness E. Type 2 diabetes mellitus, impaired glucose tolerance and associated factors in a rural Palestinian village. Diabet Med. 2000; 17(10): 746-8.

Hyska J, Mersini E, Mone I, Burazeri G. Prevalence and demographic correlates of overweight and obesity among children in a transitional southeastern European population. J Community Health. 2014; 39(5): 828–34.

Icelandic Centre for Social Research and Analysis (ICSRA). Iceland Youth Survey 1992.

Icelandic Centre for Social Research and Analysis (ICSRA). Iceland Youth Survey 2000.

Icelandic Nutrition Council. Iceland Dietary Survey 2002.

ICF International, Institute of Population Studies, Hacettepe University, Ministry of Development (Turkey), Ministry of Health (Turkey). Turkey Demographic and Health Survey 2013-2014.

ICF International, Kenya Medical Research Institute (KEMRI), Kenya National Bureau of Statistics, Ministry of Health (Kenya), National AIDS Control Council (Kenya), National Council for Population and Development (Kenya). Kenya Demographic and Health Survey 2014. Fairfax, United States: ICF International, 2015.

ICF International, Macro International, Inc, National Institute of Statistics and Informatics (INEI) (Peru). Peru Continuous Demographic and Health Survey. Calverton, United States: Macro International, Inc.

ICF International, Ministry of Economy, Planning and Regional Development (Cameroon), Ministry of Public Health (Cameroon), National Institute of Statistics (Cameroon), Pasteur Center of Cameroon. Cameroon Demographic and Health Survey 2011. Fairfax, United States: ICF International.

ICF International, Ministry of Health (Congo, Rep.), National Center for Statistics and Economic Studies (Congo, Rep.). Congo, Rep. Demographic and Health Survey 2011-2012. Fairfax, United States: ICF International, 2013.

ICF International, Ministry of Health (Kyrgyzstan), National Statistical Committee of the Kyrgyz Republic. Kyrgyzstan Demographic and Health Survey 2012. Fairfax, United States: ICF International, 2014.

ICF International, Ministry of Health (Peru), National Institute of Statistics and Informatics (INEI) (Peru), National Police of Peru (PNP). Peru Continuous Demographic and Health Survey 2014 - INEI. Lima, Peru: National Institute of Statistics and Informatics (INEI) (Peru), 2015.

ICF International, Ministry of Health (Tajikistan), Statistical Agency under the President of the Republic of Tajikistan. Tajikistan Demographic and Health Survey 2012. Fairfax, United States: ICF International, 2013.

ICF International, Ministry of Health and Sanitation (Sierra Leone), Statistics Sierra Leone. Sierra Leone Demographic and Health Survey 2013. Fairfax, United States: ICF International, 2014.

ICF International, Ministry of Health and Social Services (Namibia), Namibia Institute of Pathology, Namibia Statistics Agency. Namibia Demographic and Health Survey 2013. Fairfax, United States: ICF International, 2015.

ICF International, Ministry of Public Health (Niger), National Institute of Statistics (Niger). Niger Demographic and Health Survey 2012. Fairfax, United States: ICF International, 2014.

ICF International, Ministry of the Fight Against AIDS (Côte d'Ivoire), National Institute of Statistics (Côte d'Ivoire). Côte d'Ivoire Demographic and Health Survey 2011-2012. Fairfax, United States: ICF International, 2013.

ICF International, National Institute of Population Studies (Pakistan), Pakistan Bureau of Statistics. Pakistan Demographic and Health Survey 2012-2013. Fairfax, United States: ICF International, 2014.

ICF International, National Institute of Statistics (Honduras). Honduras Demographic and Health Survey 2011-2012. Calverton, United States: ICF Macro, 2013.

ICF International, National Institute of Statistics and Economic Analysis (INSAE) (Benin), National Program Against AIDS (PNLS) (Benin). Benin Demographic and Health Survey 2011-2012. Fairfax, United States: ICF International, 2014.

ICF International, National Institute of Statistics and Informatics (INEI) (Peru). Peru Continuous Demographic and Health Survey 2013 - INEI. Lima, Peru: National Institute of Statistics and Informatics (INEI) (Peru), 2014.

ICF International, National Population Commission of Nigeria. Nigeria Demographic and Health Survey 2013. Fairfax, United States: ICF International, 2014.

ICF Macro, Institute of Public Health (Albania), Institute of Statistics (Albania). Albania Demographic and Health Survey 2008-2009. Calverton, United States: ICF Macro, 2009.

ICF Macro, Kenya Medical Research Institute (KEMRI), Kenya National Bureau of Statistics, Ministry of Public Health and Sanitation (Kenya), National AIDS and STI Control Program (Kenya), National Aids Control Council (NACC), National Coordinating Agency for Population and Development (Kenya). Kenya Demographic and Health Survey 2008-2009. Calverton, United States: ICF Macro.

ICF Macro, Manhica Health Research Center (CISM), Ministry of Health (Mozambique), National Institute of Statistics (INE) (Mozambique). Mozambique Demographic and Health Survey 2011. Calverton, United States: ICF Macro, 2013.

ICF Macro, Ministry of Finance (Timor-Leste), National Statistics Directorate (Timor-Leste). Timor-Leste Demographic and Health Survey 2009-2010. Calverton, United States: ICF Macro.

ICF Macro, Ministry of Health (Armenia), National Statistical Service of the Republic of Armenia. Armenia Demographic and Health Survey 2010. Calverton, United States: ICF Macro, 2015.

ICF Macro, Ministry of Health (Burkina Faso), National Institute of Statistics and Demography (Burkina Faso). Burkina Faso Demographic and Health Survey 2010-2011. Calverton, United States: ICF Macro.

ICF Macro, Ministry of Health (Cambodia), National Institute of Statistics (Cambodia). Cambodia Demographic and Health Survey 2010-2011. Calverton, United States: ICF Macro, 2011.

ICF Macro, Ministry of Health (Maldives). Maldives Demographic and Health Survey 2009. Calverton, United States: ICF Macro, 2010.

ICF Macro, Ministry of Health (Rwanda), National Institute of Statistics of Rwanda. Rwanda Demographic and Health Survey 2010-2011. Calverton, United States: ICF Macro.

ICF Macro, Ministry of Health (Sao Tome and Principe), National Institute of Statistics (Sao Tome and Principe). Sao Tome and Principe Demographic and Health Survey 2008-2009. Calverton, United States: ICF Macro.

ICF Macro, Ministry of Health and Population (Nepal), New ERA. Nepal Demographic and Health Survey 2011. Calverton, United States: ICF Macro.

ICF Macro, Ministry of Health and Social Welfare (Lesotho). Lesotho Demographic and Health Survey 2009-2010. Calverton, United States: ICF Macro.

ICF Macro, Mitra and Associates, National Institute of Population Research and Training (NIPORT). Bangladesh Demographic and Health Survey 2011-2012. Calverton, United States: ICF Macro.

ICF Macro, National Bureau of Statistics (Tanzania). Tanzania Demographic and Health Survey 2009-2010. Calverton, United States: ICF Macro.

ICF Macro, National Institute of Statistics (Madagascar). Madagascar Demographic and Health Survey 2008-2009. Calverton, United States: ICF Macro, 2010.

ICF Macro, National Statistical Office of Malawi. Malawi Demographic and Health Survey 2010. Calverton, United States: ICF Macro.

ICF Macro, Profamilia. Colombia Demographic and Health Survey 2009-2010. Calverton, United States: ICF Macro, 2011.

ICF Macro, Uganda Bureau of Statistics. Uganda Demographic and Health Survey 2011. Calverton, United States: ICF Macro.

ICF Macro, Zimbabwe National Statistics Agency. Zimbabwe Demographic and Health Survey 2010-2011. Calverton, United States: ICF Macro, 2012.

Ichinohe M, Mita R, Saito K, Shinkawa H, Nakaji S, Coombs M, Carney A, Wright B, Fuller EL. The prevalence of obesity and its relationship with lifestyle factors in Jamaica. Tohoku J Exp Med. 2005; 207(1): 21-32.

Iliyasu Z, Abubakar IS, Abubakar S, Lawan UM, Gajida AU, Jibo AM. A survey of weight perception and social desirability of obesity among adults in Kano Metropolis, Northern Nigeria. Niger J Med. 2013; 22(2): 101-8.

INCLEN Multicentre Collaborative Group. Risk factors for cardiovascular disease in the developing world. A multicentre collaborative study in the International Clinical Epidemiology Network (INCLEN). J Clin Epidemiol. 1992; 45(8).

Indian Council of Medical Research (ICMR), National Institute of Medical Statistics (India). India Noncommunicable Disease Risk Factors Survey 2007-2008.

Indian Council of Medical Research (ICMR), World Health Organization (WHO). India STEPS Noncommunicable Disease Risk Factors Survey 2003-2005.

Institute for Health Metrics and Evaluation (IHME), Ministry of Health (Saudi Arabia). Saudi Arabia Health Interview Survey 2013.

Institute for Mother and Child Health, Health Home Skopje (Macedonia), National Nutrition Institute (Italy), United Nations Children's Fund (UNICEF), World Health Organization Regional Office for Europe (EURO-WHO). Macedonia Health and Nutritional Status of the Elderly Survey 1999.

Institute for Polling and Marketing (Georgia), World Health Organization (WHO). Georgia WHO Multi-country Survey Study on Health and Health System Responsiveness 2000-2001.

Institute for Public Health, Ministry of Health (Malaysia). Malaysia National Health and Morbidity Survey 2011.

Institute for Research and Documentation in Health Economics (IRDES) (France). France Survey of Health and Welfare 2004.

Institute for Research and Documentation in Health Economics (IRDES) (France). France Survey of Health and Welfare 2006.

Institute for Research and Documentation in Health Economics (IRDES) (France). France Survey of Health and Welfare 2008.

Institute for Research and Documentation in Health Economics (IRDES) (France). France Survey of Health and Welfare 2010.

Institute for Social and Economic Research, University of Essex, Gfk NOP, Office for National Statistics (ONS) (United Kingdom), Northern Ireland Statistics and Research Agency (NISRA), Economic and Social Research Council (ESRC), Health Development Agency (United Kingdom). United Kingdom British Household Panel Survey 2004-2005. Essex, United Kingdom: Institute for Social and Economic Research, University of Essex.

Institute for Social and Economic Research, University of Essex, Gfk NOP, Office for National Statistics (ONS) (United Kingdom), Northern Ireland Statistics and Research Agency (NISRA), Economic and Social Research Council (ESRC), National Institute for Health and Clinical Excellence (NICE). United Kingdom British Household Panel Survey 2006-2007. Essex, United Kingdom: Institute for Social and Economic Research, University of Essex.

Institute of Experimental and Clinical Medicine (Estonia). Estonia Health Interview Survey 1996-1997.

Institute of Health Systems (India), World Health Organization (WHO). India - Andhra Pradesh WHO Multi-country Survey Study on Health and Health System Responsiveness 2000-2001.

Institute of Nutrition of Central America and Panama, Westinghouse; Institute for Resource Development. Guatemala Demographic and Health Survey 1987. Columbia, United States: Westinghouse; Institute for Resource Development.

Institute of Obstetrics and Gynecology, Ministry of Health (Uzbekistan), Macro International, Inc, Ministry of Health (Uzbekistan). Uzbekistan Demographic and Health Survey 1996. Calverton, United States: Macro International, Inc.

Institute of Population Studies, Hacettepe University, Macro International, Inc, Ministry of Health (Turkey). Turkey Demographic and Health Survey 1993. Calverton, United States: Macro International, Inc.

Institute of Population Studies, Hacettepe University, Macro International, Inc. Turkey Demographic and Health Survey 1998. Calverton, United States: Macro International, Inc.

Institute of Population Studies, Hacettepe University, Ministry of Health (Turkey). Turkey Demographic and Health Survey 2003-2004. Ankara, Turkey: Institute of Population Studies, Hacettepe University.

Institute of Public Health (Macedonia), Ipsos Strategic Puls, Ministry of Education and Science (Macedonia), Ministry of Labor and Social Policy (Macedonia), United Nations Children's Fund (UNICEF). Macedonia Multiple Indicator Cluster Survey 2011. New York, United States: United Nations Children's Fund (UNICEF), 2013.

Institute of Public Health of Serbia, Ministry of Health (Serbia). Serbia National Health Survey 2006.

Institute of Public Health of Serbia, Ministry of Health (Serbia). Serbia National Health Survey 2013.

Institute of Social Medicine and Health Policy, Shandong University, Shandong University School of Medicine, World Health Organization (WHO). China WHO Multi-country Survey Study on Health and Health System Responsiveness 2000-2001.

Instituto Cuánto. Peru National Living Standards Measurement Survey 2000. Lima, Peru: Instituto Cuánto.

Inter-American Development Bank (IDB), National Statistics Institute (Guatemala), World Bank. Guatemala Living Standards Measurement Survey 2000. Washington DC, United States: World Bank.

International Centre for Diarrhoeal Disease Research, Bangladesh (ICDDR,B), National Institute of Cardiovascular Disease (Bangladesh). Bangladesh Risk of Acute Vascular Events (BRAVE) Tabulation on BMI by Age and Sex [Unpublished].

International Diabetes Institute (IDI). Australia Diabetes, Obesity and Lifestyle Study 1999-2000. Melbourne, Australia: International Diabetes Institute (IDI).

International Diabetes Institute (IDI). Australia Diabetes, Obesity and Lifestyle Study 2004-2005.

International Food Policy Research Institute (IFPRI), University of Natal, University of Wisconsin, Data Research Africa (DRA), Policy and Praxis, Southern Africa Labour Development Research Unit (SALDRU), School of Economics, University of Cape Town. South Africa KwaZulu-Natal Income Dynamics Study 1998. Durban, South Africa: University of Natal.

International Food Policy Research Institute (IFPRI), University of Natal, University of Wisconsin, Southern Africa Labour Development Research Unit (SALDRU), School of Economics, University of Cape Town. South Africa KwaZulu-Natal Income Dynamics Study 1993. Durban, South Africa: University of Natal.

International Institute for Population Sciences (India), Macro International, Inc. India Demographic and Health Survey 1998-1999. Calverton, United States: Macro International, Inc.

International Institute for Population Sciences (India), Macro International, Inc. India Demographic and Health Survey 2005-2006. Calverton, United States: Macro International, Inc.

International Institute for Population Sciences (India), World Health Organization (WHO). India WHO Study on Global Ageing and Adult Health 2007. Geneva, Switzerland: World Health Organization (WHO), 2007.

International Institute for Population Sciences (India), World Health Organization (WHO). India World Health Survey 2003. Geneva, Switzerland: World Health Organization (WHO), 2005.

International Institute for Population Sciences (India). India Demographic and Health Survey 1992-1993. Mumbai, India: International Institute for Population Sciences (India).

International Institute for Population Sciences (India). India District Level Household Survey 2012-2014. New Delhi, India: Ministry of Health and Family Welfare (India).

Irish Universities Nutrition Alliance (IUNA), University College Cork, University College Dublin. Ireland National Adult Nutrition Survey 2008-2010.

Isomaa R, Isomaa A-L, Marttunen M, Kaltiala-Heino R, Björkqvist K. Longitudinal concomitants of incorrect weight perception in female and male adolescents. Body Image. 2011; 8(1): 58-63.

Israel Center for Disease Control (ICDC), Nutrition Department, Ministry of Health (Israel). Israel National Health and Nutrition Survey of the Elderly Aged 65 And Over 2005-2006.

ISSP Research Group (2009): International Social Survey Programme: Health and Health Care - ISSP 2011. GESIS Data Archive, Cologne. ZA5800 Data file version 3.0.0, doi:10.4232/1.12252.

ISSP Research Group (2009): International Social Survey Programme: Leisure Time and Sports - ISSP 2007. GESIS Data Archive, Cologne. ZA4850 Data file version 2.0.0, doi:10.4231/1.10079.

Iwasaki M, Otani T, Inoue M, Sasazuki S, Tsugane S, Japan Public Health Center-Based Prospective Study Group. Body size and risk for breast cancer in relation to estrogen and progesterone receptor status in Japan. Ann Epidemiol. 2007; 17(4): 304–12.

Jääskeläinen A, Pussinen J, Nuutinen O, Schwab U, Pirkola J, Kolehmainen M, Järvelin M-R, Laitinen J. Intergenerational transmission of overweight among Finnish adolescents and their parents: a 16-year follow-up study. Int J Obes (Lond). 2011; 35(10): 1289-94.

Jabre P, Sikias P, Khater-Menassa B, Baddoura R, Awada H. Overweight children in Beirut: prevalence estimates and characteristics. Child Care Health Dev. 2005; 31(2): 159-65.

Jahns L, Adair L, Mroz T, Popkin BM. The declining prevalence of overweight among Russian children: income, diet, and physical activity behavior changes. Econ Hum Biol. 2012; 10(2): 139-46.

Jaime PC, Duran AC, Sarti FM, Lock K. Investigating environmental determinants of diet, physical activity, and overweight among adults in Sao Paulo, Brazil. J Urban Health. 2011; 88(3): 567-81.

Jakimaviciene EM, Tutkuviene J. Trends in body mass index, prevalence of overweight and obesity in preschool Lithuanian children, 1986-2006. Coll Antropol. 2007; 31(1): 79-88.

Jan Mohamed HJB, Mitra AK, Zainuddin LRM, Leng SK, Wan Muda WM. Women are at a higher risk of metabolic syndrome in rural Malaysia. Women Health. 2013; 53(4): 335-48.

Janghorbani M, Amini M, Rezvanian H, Gouya M-M, Delavari A, Alikhani S, Mahdavi A. Association of body mass index and abdominal obesity with marital status in adults. Arch Iran Med. 2008; 11(3): 274-81.

Janus ED, Laatikainen T, Dunbar JA, Kilkkinen A, Bunker SJ, Philpot B, Tideman PA, Tirimacco R, Heistaro S. Overweight, obesity and metabolic syndrome in rural southeastern Australia. Med J Aust. 2007; 187(3): 147-52.

Jayawardena R, Byrne NM, Soares MJ, Katulanda P, Hills AP. Body weight perception and weight loss practices among Sri Lankan adults. Obes Res Clin Pract. 2014; 8(2): e192–200.

Jebb SA, Rennie KL, Cole TJ. Prevalence of overweight and obesity among young people in Great Britain. Public Health Nutr. 2004; 7(3): 461-5.

Jiang J, Rosenqvist U, Wang H, Greiner T, Ma Y, Toschke AM. Risk factors for overweight in 2- to 6-year-old children in Beijing, China. Int J Pediatr Obes. 2006; 1(2): 103-8.

Jiang X-X, Hardy LL, Baur LA, Ding D, Wang L, Shi H-J. High prevalence of overweight and obesity among inner city Chinese children in Shanghai, 2011. Ann Hum Biol. 2014; 41(5): 469–72.

Jinabhai CC, Taylor M, Sullivan KR. Changing patterns of under- and over-nutrition in South African children-future risks of non-communicable diseases. Ann Trop Paediatr. 2005; 25(1): 3-15.

Jodkowska M, Oblacinska A, Tabak I. Overweight and obesity among adolescents in Poland: gender and regional differences. Public Health Nutr. 2010; 13(10A): 1688-92.

Joffres MR, Hamet P, Rabkin SW, Gelskey D, Hogan K, Fodor G. Prevalence, control and awareness of high blood pressure among Canadian adults. Canadian Heart Health Surveys Research Group. CMAJ. 1992; 146(11): 1997-2005.

Joint Health Surveys Unit of Social and Community Planning Research and University College London, Scottish Health Survey, 1998 [computer file]. Colchester, Essex: UK Data Archive [distributor], July 2001. SN: 4379 , http://dx.doi.org/10.5255/UKDA-SN-4379-1.

Joint Health Surveys Unit of Social and Community Planning Research and University College London, Health Survey for England, 1995 [computer file]. 3rd ed. Colchester, Essex: UK Data Archive [distributor], 26 March 2001. SN: 3796.

Joint Health Surveys Unit of Social and Community Planning Research and University College London, Health Survey for England, 1996 [computer file]. 3rd ed. Colchester, Essex: UK Data Archive [distributor], March 2001. SN: 3886.

Joint Health Surveys Unit of Social and Community Planning Research and University College London, Health Survey for England, 1997 [computer file]. 2nd ed. Colchester, Essex: UK Data Archive [distributor], 4 December 2000. SN: 3979.

Joint Health Surveys Unit of Social and Community Planning Research and University College London, Scottish Health Survey, 1995 [computer file]. 3rd ed. Colchester, Essex: UK Data Archive [distributor], 11 February 1999. SN: 3807.

Joint Health Surveys Unit, University College London and Medical Research Council. Social and Public Health Sciences Unit, Scottish Health Survey, 2003 [computer file]. Colchester, Essex: UK Data Archive [distributor], February 2006. SN: 5318.

Jones DW, Kim JS, Andrew ME, Kim SJ, Hong YP. Body mass index and blood pressure in Korean men and women: the Korean National Blood Pressure Survey. J Hypertens. 1994; 12(12): 1433-7.

Joshi MD, Ayah R, Njau EK, Wanjiru R, Kayima JK, Njeru EK, Mutai KK. Prevalence of hypertension and associated cardiovascular risk factors in an urban slum in Nairobi, Kenya: a population-based survey. BMC Public Health. 2014; 14: 1177.

Júlíusson PB, Eide GE, Roelants M, Waaler PE, Hauspie R, Bjerknes R. Overweight and obesity in Norwegian children: prevalence and socio-demographic risk factors. Acta Paediatr. 2010; 99(6): 900-5.

Kadota A, Hozawa A, Okamura T, Kadowak T, Nakmaura K, Murakami Y, Hayakawa T, Kita Y, Okayama A, Nakamura Y, Kashiwagi A, Ueshima H, NIPPON DATA Research Group. Relationship between metabolic risk factor clustering and cardiovascular mortality stratified by high blood glucose and obesity: NIPPON DATA90, 1990-2000. Diabetes Care. 2007; 30(6): 1533-8.

Kalies H, Lenz J, von Kries R. Prevalence of overweight and obesity and trends in body mass index in German pre-school children, 1982-1997. Int J Obes Relat Metab Disord. 2002; 26(9): 1211-7.

Kamadjeu RM, Edwards R, Atanga JS, Kiawi EC, Unwin N, Mbanya J-C. Anthropometry measures and prevalence of obesity in the urban adult population of Camerron: an update from the Cameroon Burden of Diabetes Baseline Survey. BMC Public Health. 2006; 228.

Kambas A, Venetsanou F, Avloniti A, Giannakidou DM, Gourgoulis V, Draganidis D, Chatzinikolaou A, Fatouros I, Michalopoulou M. Pedometer determined physical activity and obesity prevalence of Greek children aged 4-6 years. Ann Hum Biol. 2015; 42(3): 231–6.

Kapci N, Akcam M, Koca T, Dereci S, Kapci M. The nutritional status of hospitalized children: Has this subject been overlooked?. Turk J Gastroenterol. 2015; 26(4): 351–5.

Karayiannis D, Yannakoulia M, Terzidou M, Sidossis LS, Kokkevi A. Prevalence of overweight and obesity in Greek school-aged children and adolescents. Eur J Clin Nutr. 2003; 57(9): 1189-92.

Kaur S, Sachdev HPS, Dwivedi SN, Lakshmy R, Kapil U. Prevalence of overweight and obesity amongst school children in Delhi, India. Asia Pac J Clin Nutr. 2008; 17(4): 592-6.

Kautiainen S, Koivisto A-M, Koivusilta L, Lintonen T, Virtanen SM, Rimpelä A. Sociodemographic factors and a secular trend of adolescent overweight in Finland. Int J Pediatr Obes. 2009; 4(4): 360-70.

Kavishe B, Biraro S, Baisley K, Vanobberghen F, Kapiga S, Munderi P, Smeeth L, Peck R, Mghamba J, Mutungi G, Ikoona E, Levin J, Bou Monclús MA, Katende D, Kisanga E, Hayes R, Grosskurth H. High prevalence of hypertension and of risk factors for non-communicable diseases (NCDs): a population based cross-sectional survey of NCDS and HIV infection in Northwestern Tanzania and Southern Uganda. BMC Med. 2015; 13: 126.

Kayina KP, Sharma AK, Agrawal K. Implementation of ICD 10: a study on the doctors' knowledge and coding practices in Delhi. Indian J Public Health. 2015; 59(1): 68–9.

Kelishadi R, Ardalan G, Gheiratmand R, Majdzadeh R, Hosseini M, Gouya MM, Razaghi EM, Delavari A, Motaghian M, Barekati H, Mahmoud-Arabi MS, Lock K, Caspian Study Group. Thinness, overweight and obesity in a national sample of Iranian children and adolescents: CASPIAN Study. Child Care Health Dev. 2008; 34(1): 44-54.

Kelishadi R, Gharipour M, Sadri GH, Tavasoli AA, Amani A. Cardiovascular disease risk factors, metabolic syndrome and obesity in an Iranian population. East Mediterr Health J. 2008; 14(5): 1070-9.

Kenya National Bureau of Statistics, Population Studies and Research Institute, University of Nairobi (Kenya), United Nations Children's Fund (UNICEF). Kenya - Bungoma County Multiple Indicator Survey 2013-2014. New York, United States: United Nations Children's Fund (UNICEF), 2015.

Kenya National Bureau of Statistics, Population Studies and Research Institute, University of Nairobi (Kenya), United Nations Children's Fund (UNICEF). Kenya - Kakamega County Multiple Indicator Survey 2013-2014. New York, United States: United Nations Children's Fund (UNICEF), 2015.

Kenya National Bureau of Statistics, Population Studies and Research Institute, University of Nairobi (Kenya), United Nations Children's Fund (UNICEF). Kenya - Turkana County Multiple Indicator Survey 2013-2014. New York, United States: United Nations Children's Fund (UNICEF), 2015.

Kenya National Bureau of Statistics, United Nations Children's Fund (UNICEF). Kenya - North Eastern Province Multiple Indicator Cluster Survey 2007. Nairobi, Kenya: Kenya National Bureau of Statistics.

Kenya National Bureau of Statistics, United Nations Children's Fund (UNICEF). Kenya - Nyanza Province Multiple Indicator Cluster Survey 2011. Nairobi, Kenya: Kenya National Bureau of Statistics.

Khader Y, Batieha A, Ajlouni H, El-Khateeb M, Ajlouni K. Obesity in Jordan: prevalence, associated factors, comorbidities, and change in prevalence over ten years. Metab Syndr Relat Disord. 2008; 6(2): 113-20.

Khader YS, Batieha A, Jaddou H, Batieha Z, El-Khateeb M, Ajlouni K. Metabolic abnormalities associated with obesity in children and adolescents in Jordan. Int J Pediatr Obes. 2011; 6(3-4): 215-22.

Khan MKA, Chu YL, Kirk SFL, Veugelers PJ. Are sleep duration and sleep quality associated with diet quality, physical activity, and body weight status? A population-based study of Canadian children. Can J Public Health. 2015; 106(5): 277–82.

Khazaie H, Najafi F, Rezaie L, Tahmasian M, Sepehry AA, Herth FJF. Prevalence of symptoms and risk of obstructive sleep apnea syndrome in the general population. Arch Iran Med. 2011; 14(5): 335-8.

Kim YC, Koo HS, Kim S, Chin HJ. Estimation of daily salt intake through a 24-hour urine collection in Pohang, Korea. J Korean Med Sci. 2014; 29 Suppl 2: S87–90.

King H, Keuky L, Seng S, Khun T, Roglic G, Pinget M. Diabetes and associated disorders in Cambodia: two epidemiological surveys. Lancet. 2005; 366(9497): 1633-9.

Klein-Platat C, Wagner A, Haan MC, Arveiler D, Schlienger JL, Simon C. Prevalence and sociodemographic determinants of overweight in young French adolescents. Diabetes Metab Res Rev. 2003; 19(2): 153-8.

Knowles G, Ling FCM, Thomas GN, Adab P, McManus AM. Body size dissatisfaction among young Chinese children in Hong Kong: a cross-sectional study. Public Health Nutr. 2015; 18(6): 1067–74.

Ko GTC, Ozaki R, Wong GWK, Kong APS, So W-Y, Tong PCY, Chan MHM, Ho C-S, Lam CWK, Chan JCN. The problem of obesity among adolescents in Hong Kong: a comparison using various diagnostic criteria. BMC Pediatr. 2008; 10.

Kontogianni MD, Farmaki A-E, Vidra N, Sofrona S, Magkanari F, Yannakoulia M. Associations between lifestyle patterns and body mass index in a sample of Greek children and adolescents. J Am Diet Assoc. 2010; 110(2): 215-21.

Konttinen H, Kiviruusu O, Huurre T, Haukkala A, Aro H, Marttunen M. Longitudinal associations between depressive symptoms and body mass index in a 20-year follow-up. Int J Obes (Lond). 2014; 38(5): 668–74.

Korea Centers for Disease Control and Prevention. Korea, South National Health and Nutrition Examination Survey 1998.

Kortt MA, Dollery B. Association between body mass index and health-related quality of life among an Australian sample. Clin Ther. 2011; 33(10): 1466-74.

Kortt MA, Dollery B. Religion and BMI in Australia. J Relig Health. 2014; 53(1): 217–28.

Koskenvuo K, Hublin C, Partinen M, Paunio T, Koskenvuo M. Childhood adversities and quality of sleep in adulthood: A population-based study of 26,000 Finns. Sleep Med. 2010; 11(1): 17-22.

Kouda K, Nakamura H, Tokunaga R, Takeuchi H. Trends in levels of cholesterol in Japanese children from 1993 through 2001. J Epidemiol. 2004; 14(3): 78-82.

Koziel S, Welon Z, Bielicki T, Szklarska A, Ulijaszek S. The effect of the economic transition on the body mass index of conscripts in Poland. Econ Hum Biol. 2004; 2(1): 97–106.

Kruger R, Kruger HS, Macintyre UE. The determinants of overweight and obesity among 10- to 15-year-old schoolchildren in the North West Province, South Africa - the THUSA BANA (Transition and Health during Urbanisation of South Africans; BANA, children) study. Public Health Nutr. 2006; 9(3): 351-8.

Kull M, Matsi J, Raudsepp L. Relationship between various physical activity domains and self-perceived health and obesity in women. Women Health. 2010; 50(7): 639-51.

Kunešová M, Vignerová J, Parízková J, Procházka B, Braunerová R, Riedlová J, Zamrazilová H, Hill M, Bláha P Steflová A. Long-term changes in prevalence of overweight and obesity in Czech 7-year-old children: evaluation of different cut-off criteria of childhood obesity. Obes Rev. 2011; 12(7): 483-91.

Kuriyama S, Tsubono Y, Hozawa A, Shimazu T, Suzuki Y, Koizumi Y, Suzuki Y, Ohmori K, Nishino Y, Tsuji I. Obesity and risk of cancer in Japan. Int J Cancer. 2005; 113(1): 148–57.

Kvaavik E, Tell GS, Klepp K-I. Predictors and tracking of body mass index from adolescence into adulthood: follow-up of 18 to 20 years in the Oslo Youth Study. Arch Pediatr Adolesc Med. 2003; 157(12): 1212-8.

Lager ACJ, Fossum B, Rörvall G, Bremberg SG. Children's overweight and obesity: local and national monitoring using electronic health records. Scand J Public Health. 2009; 37(2): 201-5.

Laitinen J, Ek E, Sovio U. Stress-related eating and drinking behavior and body mass index and predictors of this behavior. Prev Med. 2002; 34(1): 29-39.

Lao XQ, Ma WJ, Sobko T, Zhang YH, Xu YJ, Xu XJ, Yu DM, Nie SP, Cai QM, Xia L, Thomas GN, Griffiths SM. Overall obesity is leveling-off while abdominal obesity continues to rise in a Chinese population experiencing rapid economic development: analysis of serial cross-sectional health survey data 2002-2010. Int J Obes (Lond). 2015; 39(2): 288–94.

Lasserre AM, Chiolero A, Cachat F, Paccaud F, Bovet P. Overweight in Swiss children and associations with children's and parents' characteristics. Obesity (Silver Spring). 2007; 15(12): 2912-9.

Laurson KR, Welk GJ, Marton O, Kaj M, Csanyi T. Agreement and Diagnostic Performance of FITNESSGRAM, International Obesity Task Force, and Hungarian National BMI Standards. Res Q Exerc Sport. 2015; 86 Suppl 1: S21-28.

Lazarou C, Soteriades ES. Children's physical activity, TV watching and obesity in Cyprus: the CYKIDS study. Eur J Public Health. 2010; 20(1): 70-7.

Lazzeri G, Giacchi MV, Spinelli A, Pammolli A, Dalmasso P, Nardone P, Lamberti A, Cavallo F. Overweight among students aged 11-15 years and its relationship with breakfast, area of residence and parents' education: results from the Italian HBSC 2010 cross-sectional study. Nutr J. 2014; 13: 69.

Lazzeri G, Pammolli A, Pilato V, Giacchi MV. Relationship between 8/9-yr-old school children BMI, parents' BMI and educational level: a cross sectional survey. Open Nutr J. 2011; 76.

Lazzeri G, Pammolli A, Simi R, Pilato V, Giacchi MV. BMI from nutritional surveillance of 8-9 years old children in Tuscany (Italy). J Prev Med Hyg. 2011; 52(4): 181-5.

Le Pluart D, Sabate J-M, Bouchoucha M, Hercberg S, Benamouzig R, Julia C. Functional gastrointestinal disorders in 35,447 adults and their association with body mass index. Aliment Pharmacol Ther. 2015; 41(8): 758–67.

League of Arab States, Macro International, Inc, Ministry of Health (Morocco). Morocco Demographic and Health Survey 2003-2004. Calverton, United States: Macro International, Inc.

Leal DB, de Assis MAA, Gonzalez-Chica DA, da Costa FF. Trends in adiposity in Brazilian 7-10-year-old schoolchildren evidence for increasing overweight but not obesity between 2002 and 2007. Ann Hum Biol. 2014; 41(3): 255–62.

Lee K, Lee S, Kim SY, Kim SJ, Kim YJ. Percent body fat cutoff values for classifying overweight and obesity recommended by the International Obesity Task Force (IOTF) in Korean children. Asia Pac J Clin Nutr. 2007; 16(4): 649-55.

Lee K, Song Y-M. Parent-reported appetite of a child and the child's weight status over a 2-year period in Korean children. J Am Diet Assoc. 2007; 107(4): 678-80.

Lee S-A, Wen W, Xu WH, Zheng W, Li H, Yang G, Xiang Y-B, Shu X-O. Prevalence of obesity and correlations with lifestyle and dietary factors in Chinese men. Obesity (Silver Spring). 2008; 16(6): 1440-7.

Leech RM, McNaughton SA, Timperio A. Clustering of diet, physical activity and sedentary behaviour among Australian children: cross-sectional and longitudinal associations with overweight and obesity. Int J Obes. 2015; 39(7): 1079–85.

Léger D, Roscoat E du, Bayon V, Guignard R, Pâquereau J, Beck F. Short sleep in young adults: Insomnia or sleep debt? Prevalence and clinical description of short sleep in a representative sample of 1004 young adults from France. Sleep Med. 2011; 12(5): 454-62.

Leong SL, Madden C, Gray A, Horwath C. Self-determined, autonomous regulation of eating behavior is related to lower body mass index in a nationwide survey of middle-aged women. J Acad Nutr Diet. 2012; 112(9): 1337-46.

Leskosek B, Strel J, Kovac M. Overweight and obesity in Slovenian schoolgirls, 1991-2006. Coll Antropol. 2010; 34(4): 1303-8.

Li C-L, Chen S-Y, Lan C, Pan W-H, Chou H-C, Bai Y-B, Tzeng M-S, Lee M-S, Lai J-S. The effects of physical activity, body mass index (BMI) and waist circumference (WC) on glucose intolerance in older people: a nationwide study from Taiwan. Arch Gerontol Geriatr. 2011; 52(1): 54–9.

Li M, Dibley MJ, Sibbritt D, Yan H. An assessment of adolescent overweight and obesity in Xi'an City, China. Int J Pediatr Obes. 2006; 1(1): 50-8.

Li Y, Schouten EG, Hu X, Cui Z, Luan D, Ma G. Obesity prevalence and time trend among youngsters in China, 1982-2002. Asia Pac J Clin Nutr. 2008; 17(1): 131-7.

Liberia Institute for Statistics and Geo-information Services (LISGIS), Macro International, Inc. Liberia Demographic and Health Survey 2006-2007. Calverton, United States: Macro International, Inc.

Liese AD, Hirsch T, von Mutius E, Weiland SK. Burden of overweight in Germany: prevalence differences between former East and West German children. Eur J Public Health. 2006; 16(5): 526-31.

Lin Y, Bolca S, Vandevijvere S, De Vriese S, Mouratidou T, De Neve M, Polet A, Van Oyen H, Van Camp J, De Backer G, De Henauw S, Huybrechts I. Plant and animal protein intake and its association with overweight and obesity among the Belgian population. Br J Nutr. 2011; 105(7): 1106-16.

Lin Y, Wolk A, Hakansson N, Peñalvo JL, Lagergren J, Adlercreutz H, Lu Y. Validation of FFQ-based assessment of dietary lignans compared with serum enterolactone in Swedish women. Br J Nutr. 2013; 109(10): 1873-80.

Lintula T, Laitala V, Pesonen P, Sipilä K, Laitala M-L, Taanila A, Anttonen V. Self-reported oral health and associated factors in the North Finland 1966 birth cohort at the age of 31. BMC Oral Health. 2014; 14: 155.

Liou T-H, Huang Y-C, Chou P. Prevalence and secular trends in overweight and obese Taiwanese children and adolescents in 1991-2003. Ann Hum Biol. 2009; 36(2): 176-85.

Lissner L, Mehlig K, Sjöberg A, Chaplin J, Niklasson A, Albertsson-Wikland K. Secular trends in weight, height and BMI in young Swedes: the "Grow up Gothenburg" studies. Acta Paediatr. 2013; 102(3): 314-7.

Löfdahl HE, Lane A, Lu Y, Lagergren P, Harvey RF, Blazeby JM, Lagergren J. Increased population prevalence of reflux and obesity in the United Kingdom compared with Sweden: a potential explanation for the difference in incidence of esophageal adenocarcinoma. Eur J Gastroenterol Hepatol. 2011; 23(2): 128-32.

Lonnee-Hoffmann RAM, Salvesen O, Morkved S, Schei B. Self-reported pelvic organ prolapse surgery, prevalence, and nonobstetric risk factors: findings from the Nord Trøndelag Health Study. Int Urogynecol J. 2015; 26(3): 407–14.

Lopes VP, Stodden DF, Rodrigues LP. Weight status is associated with cross-sectional trajectories of motor co-ordination across childhood. Child Care Health Dev. 2014; 40(6): 891–9.

Lotfi MH, Saadati H, Afzali M. Prevalence of diabetes in people aged ≥ 30 years: the results of screen-ing program of Yazd Province, Iran, in 2012. J Res Health Sci. 2014; 14(1): 87–91.

Lundeen EA, Norris SA, Adair LS, Richter LM, Stein AD. Sex differences in obesity incidence: 20-year prospective cohort in South Africa. Pediatr Obes. 2016; 11(1): 75–80.

Lutomski JE, van den Broeck J, Harrington J, Shiely F, Perry IJ. Sociodemographic, lifestyle, mental health and dietary factors associated with direction of misreporting of energy intake. Public Health Nutr. 2011; 14(3): 532-41.

Ly KA, Ton TGN, Ngo QV, Vo TT, Fitzpatrick AL. Double burden: a cross-sectional survey assessing factors associated with underweight and overweight status in Danang, Vietnam. BMC Public Health. 2013; 35.

Ma Y-N, Chen T, Wang D, Liu M-M, He Q-C, Dong G-H. Prevalence of overweight and obesity among preschool children from six cities of northeast China. Arch Med Res. 2011; 42(7): 633-40.

Machado EC, Silveira MF da, Silveira VMF da. Prevalence of weight-loss strategies and use of substances for weight-loss among adults: a population study. Cad Saude Publica. 2012; 28(8): 1439-49.

Macro International, Inc, Ministry of Economy, Planning and Regional Development (Cameroon). Cameroon Demographic and Health Survey 1991. Calverton, United States: Macro International, Inc.

Macro International, Inc, Ministry of Health (Cambodia), National Institute of Statistics (Cambodia). Cambodia Demographic and Health Survey 2000. Calverton, United States: Macro International, Inc.

Macro International, Inc, Ministry of Health (Kyrgyzstan), Research Institute of Obstetrics and Pediatrics (Kyrgyzstan). Kyrgyzstan Demographic and Health Survey 1997. Calverton, United States: Macro International, Inc.

Macro International, Inc, Ministry of Health (Mali), National Directorate of Statistics and Informatics (DNSI) (Mali). Mali Demographic and Health Survey 2006. Calverton, United States: Macro International, Inc.

Macro International, Inc, Ministry of Health (Nicaragua), National Institute of Statistics and Censuses (Nicaragua). Nicaragua Demographic and Health Survey 1997-1998. Calverton, United States: Macro International, Inc.

Macro International, Inc, Ministry of Health (Nicaragua), National Institute of Statistics and Censuses (Nicaragua). Nicaragua Demographic and Health Survey 2001. Calverton, United States: Macro International, Inc.

Macro International, Inc, Ministry of Health and Population (Nepal), New ERA. Nepal Demographic and Health Survey 1996. Calverton, United States: Macro International, Inc.

Macro International, Inc, Ministry of Health and Population (Nepal), New ERA. Nepal Demographic and Health Survey 2001. Calverton, United States: Macro International, Inc.

Macro International, Inc, Ministry of Health and Population (Nepal), New ERA. Nepal Demographic and Health Survey 2006. Calverton, United States: Macro International, Inc.

Macro International, Inc, Ministry of Health and Social Services (Namibia), National Planning Commission (Namibia). Namibia Demographic and Health Survey 2000. Calverton, United States: Macro International, Inc.

Macro International, Inc, Ministry of Health and Social Services (Namibia). Namibia Demographic and Health Survey 2006-2007. Calverton, United States: Macro International, Inc.

Macro International, Inc, Ministry of Health and Sports (Bolivia), National Institute of Statistics (Bolivia). Bolivia Demographic and Health Survey 2003-2004. Calverton, United States: Macro International, Inc.

Macro International, Inc, Ministry of Health and Sports (Bolivia), National Institute of Statistics (Bolivia). Bolivia Demographic and Health Survey 2008. Calverton, United States: Macro International, Inc.

Macro International, Inc, Ministry of Planning (Congo, DR). Congo, DR Demographic and Health Survey 2007. Calverton, United States: Macro International, Inc.

Macro International, Inc, Ministry of Public Health (Morocco). Morocco Demographic and Health Survey 1992. Calverton, United States: Macro International, Inc.

Macro International, Inc, Mitra and Associates, National Institute of Population Research and Training (NIPORT). Bangladesh Demographic and Health Survey 1996-1997. Calverton, United States: Macro International, Inc.

Macro International, Inc, Mitra and Associates, National Institute of Population Research and Training (NIPORT). Bangladesh Demographic and Health Survey 2007. Calverton, United States: Macro International, Inc, 2009.

Macro International, Inc, National Bureau of Statistics (Tanzania). Tanzania Demographic and Health Survey 2004-2005. Calverton, United States: Macro International, Inc.

Macro International, Inc, National Center for Statistics and Economic Studies (Congo, Rep.). Congo, Rep. Demographic and Health Survey 2005. Calverton, United States: Macro International, Inc.

Macro International, Inc, National Centre of Documentation and Scientific Research (Comoros). Comoros Demographic and Health Survey 1996. Calverton, United States: Macro International, Inc.

Macro International, Inc, National Directorate of Statistics and Informatics (DNSI) (Mali), Planning and Statistics Unit, Ministry of Health (Mali). Mali Demographic and Health Survey 1995-1996. Calverton, United States: Macro International, Inc.

Macro International, Inc, National Directorate of Statistics and Informatics (DNSI) (Mali), Planning and Statistics Unit, Ministry of Health (Mali). Mali Demographic and Health Survey 2001. Calverton, United States: Macro International, Inc.

Macro International, Inc, National Institute of Nutrition (Kazakhstan). Kazakhstan Demographic and Health Survey 1995. Calverton, United States: Macro International, Inc.

Macro International, Inc, National Institute of Public Health (Cambodia), National Institute of Statistics (Cambodia). Cambodia Demographic and Health Survey 2005-2006. Calverton, United States: Macro International, Inc.

Macro International, Inc, National Institute of Statistical, Economic and Demographic Studies (Chad). Chad Demographic and Health Survey 2004. Calverton, United States: Macro International, Inc.

Macro International, Inc, National Institute of Statistics (Bolivia). Bolivia Demographic and Health Survey 1993-1994. Calverton, United States: Macro International, Inc.

Macro International, Inc, National Institute of Statistics (Bolivia). Bolivia Demographic and Health Survey 1998. Calverton, United States: Macro International, Inc.

Macro International, Inc, National Institute of Statistics (Cameroon). Cameroon Demographic and Health Survey 2004. Calverton, United States: Macro International, Inc.

Macro International, Inc, National Institute of Statistics (Côte d'Ivoire). Côte d'Ivoire Demographic and Health Survey 1994. Calverton, United States: Macro International, Inc.

Macro International, Inc, National Institute of Statistics (Côte d'Ivoire). Côte d'Ivoire Demographic and Health Survey 1998-1999. Calverton, United States: Macro International, Inc.

Macro International, Inc, National Institute of Statistics (Honduras), Secretary of Health (Honduras). Honduras Demographic and Health Survey 2005-2006. Calverton, United States: Macro International, Inc.

Macro International, Inc, National Institute of Statistics (Madagascar). Madagascar Demographic and Health Survey 1997. Calverton, United States: Macro International, Inc.

Macro International, Inc, National Institute of Statistics (Madagascar). Madagascar Demographic and Health Survey 2003-2004. Calverton, United States: Macro International, Inc.

Macro International, Inc, National Institute of Statistics (Peru), PRISMA (Peru). Peru Demographic and Health Survey 1991-1992. Calverton, United States: Macro International, Inc.

Macro International, Inc, National Institute of Statistics (Peru). Peru Demographic and Health Survey 1996. Calverton, United States: Macro International, Inc.

Macro International, Inc, National Institute of Statistics (Peru). Peru Demographic and Health Survey 2000. Calverton, United States: Macro International, Inc.

Macro International, Inc, National Institute of Statistics and Demography (Burkina Faso). Burkina Faso Demographic and Health Survey 1992-1993. Calverton, United States: Macro International, Inc.

Macro International, Inc, National Institute of Statistics and Demography (Burkina Faso). Burkina Faso Demographic and Health Survey 1998-1999. Calverton, United States: Macro International, Inc.

Macro International, Inc, National Institute of Statistics and Demography (Burkina Faso). Burkina Faso Demographic and Health Survey 2003. Calverton, United States: Macro International, Inc.

Macro International, Inc, National Institute of Statistics and Economic Analysis (INSAE) (Benin). Benin Demographic and Health Survey 1996. Calverton, United States: Macro International, Inc.

Macro International, Inc, National Institute of Statistics of Rwanda. Rwanda Demographic and Health Survey 2005. Calverton, United States: Macro International, Inc.

Macro International, Inc, National Office of Population (Rwanda). Rwanda Demographic and Health Survey 1992. Calverton, United States: Macro International, Inc.

Macro International, Inc, National Office of Population (Rwanda). Rwanda Demographic and Health Survey 2000. Calverton, United States: Macro International, Inc.

Macro International, Inc, National Office of Statistics (Mauritania). Mauritania Demographic and Health Survey 2000-2001. Calverton, United States: Macro International, Inc.

Macro International, Inc, National Population Commission of Nigeria. Nigeria Demographic and Health Survey 2008. Calverton, United States: Macro International, Inc, 2009.

Macro International, Inc, National Population Council (Egypt). Egypt Demographic and Health Survey 1992-1993. Calverton, United States: Macro International, Inc.

Macro International, Inc, National Population Council (Egypt). Egypt Demographic and Health Survey 1995-1996. Calverton, United States: Macro International, Inc.

Macro International, Inc, National Population Council (Egypt). Egypt Demographic and Health Survey 2000. Calverton, United States: Macro International, Inc.

Macro International, Inc, National Scientific and Applied Center for Preventive Medicine (Moldova). Moldova Demographic and Health Survey 2005. Calverton, United States: Macro International, Inc.

Macro International, Inc, National Statistical Office of Malawi. Malawi Demographic and Health Survey 1992. Calverton, United States: Macro International, Inc.

Macro International, Inc, National Statistical Office of Malawi. Malawi Demographic and Health Survey 2000. Calverton, United States: Macro International, Inc.

Macro International, Inc, National Statistical Office of Malawi. Malawi Demographic and Health Survey 2004-2005. Calverton, United States: Macro International, Inc.

Macro International, Inc, National Statistics and Evaluation Office (Eritrea). Eritrea Demographic and Health Survey 2002. Calverton, United States: Macro International, Inc.

Macro International, Inc, National Statistics Directorate (Guinea). Guinea Demographic and Health Survey 1999. Calverton, United States: Macro International, Inc.

Macro International, Inc, National Statistics Directorate (Guinea). Guinea Demographic and Health Survey 2005. Calverton, United States: Macro International, Inc.

Macro International, Inc, National Statistics Institute (Guatemala). Guatemala Demographic and Health Survey 1995. Calverton, United States: Macro International, Inc.

Macro International, Inc, National Statistics Institute (Guatemala). Guatemala Interim Demographic and Health Survey 1998-1999. Calverton, United States: Macro International, Inc.

Macro International, Inc, National Statistics Institute (Mozambique). Mozambique Demographic and Health Survey 1997. Calverton, United States: Macro International, Inc.

Macro International, Inc, National Statistics Institute (Mozambique). Mozambique Demographic and Health Survey 2003. Calverton, United States: Macro International, Inc.

Macro International, Inc, National Statistics Office (Eritrea). Eritrea Demographic and Health Survey 1995-1996. Calverton, United States: Macro International, Inc.

Macro International, Inc, National Statistics Office (Philippines). Philippines Demographic and Health Survey 1993. Calverton, United States: Macro International, Inc.

Macro International, Inc, Population and Housing Census Commissions Office (PHCCO). Ethiopia Demographic and Health Survey 2005. Calverton, United States: Macro International, Inc.

Macro International, Inc, Profamilia. Colombia Demographic and Health Survey 1995. Calverton, United States: Macro International, Inc.

Macro International, Inc, Profamilia. Colombia Demographic and Health Survey 2000. Calverton, United States: Macro International, Inc.

Macro International, Inc, Profamilia. Colombia Demographic and Health Survey 2004-2005. Calverton, United States: Macro International, Inc.

Macro International, Inc, RAND Corporation, University of California, Los Angeles (UCLA), University of Indonesia. Indonesia Family Life Survey 1997.

Macro International, Inc, State Statistical Committee of Azerbaijan. Azerbaijan Demographic and Health Survey 2006. Calverton, United States: Macro International, Inc.

Macro International, Inc, Statistics Department (Uganda). Uganda Demographic and Health Survey 1995. Calverton, United States: Macro International, Inc.

Macro International, Inc, Statistics Sierra Leone. Sierra Leone Demographic and Health Survey 2008. Calverton, United States: Macro International, Inc.

Macro International, Inc, Uganda Bureau of Statistics. Uganda Demographic and Health Survey 2000-2001. Calverton, United States: Macro International, Inc.

Macro International, Inc, Uganda Bureau of Statistics. Uganda Demographic and Health Survey 2006. Calverton, United States: Macro International, Inc.

Macro International, Inc.; Institute for Resource Development, National Institute of Population Studies (Pakistan). Pakistan Demographic and Health Survey 1990-1991. Calverton, United States: Macro International, Inc.

Macro International, Inc.; Institute for Resource Development, National Planning Office (Dominican Republic), Profamilia. Dominican Republic Demographic and Health Survey 1991. Calverton, United States: Macro International, Inc.

Macro Systems, Inc, Mitra and Associates, National Institute of Population Research and Training (NIPORT). Bangladesh Demographic and Health Survey 1999-2000. Calverton, United States: Macro International, Inc.

Macro Systems, Inc.; Institute for Resource Development, Makerere University, Ministry of Health (Uganda). Uganda Demographic and Health Survey 1988-1989. Columbia, United States: Macro Systems, Inc.

Macro Systems, Inc.; Institute for Resource Development, National Institute of Statistics (Bolivia). Bolivia Demographic and Health Survey 1989. Columbia, United States: Macro Systems, Inc.

Macro Systems, Inc.; Institute for Resource Development, National Office for Family and Population, Ministry of Public Health (Tunisia). Tunisia Demographic and Health Survey 1988. Columbia, United States: Macro Systems, Inc.

Macro Systems, Inc.; Institute for Resource Development, Paraguayan Center for Population Studies (CEPEP). Paraguay Demographic and Health Survey 1990. Columbia, United States: Macro Systems, Inc.

Maddah M, Nikooyeh B. Factors associated with overweight in children in Rasht, Iran: gender, maternal education, skipping breakfast and parental obesity. Public Health Nutr. 2010; 13(2): 196-200.

Maddah M, Nikooyeh B. Obesity among Iranian adolescent girls: location of residence and parental obesity. J Health Popul Nutr. 2010; 28(1): 61-6.

Maddah M. Overweight and obesity among Iranian female adolescents in Rasht: more overweight in the lower social group. Public Health Nutr. 2007; 10(5): 450-3.

Maffeis C, Consolaro A, Cavarzere P, Chini L, Banzato C, Grezzani A, Silvagni D, Salzano G, De Luca F, Tatò L. Prevalence of overweight and obesity in 2- to 6-year-old Italian children. Obesity (Silver Spring). 2006; 14(5): 765-9.

Magarey AM, Daniels LA, Boulton TJ. Prevalence of overweight and obesity in Australian children and adolescents: reassessment of 1985 and 1995 data against new standard international definitions. Med J Aust. 2001; 174(11): 561-4.

Magkos F, Manios Y, Christakis G, Kafatos AG. Secular trends in cardiovascular risk factors among school-aged boys from Crete, Greece, 1982-2002. Eur J Clin Nutr. 2005; 59(1): 1-7.

Malik M, Bakir A. Prevalence of overweight and obesity among children in the United Arab Emirates. Obes Rev. 2007; 8(1): 15-20.

Manios Y, Costarelli V, Kolotourou M, Kondakis K, Tzavara C, Moschonis G. Prevalence of obesity in preschool Greek children, in relation to parental characteristics and region of residence. BMC Public Health. 2007; 178.

Manios Y, Kolotourou M, Moschonis G, Sur H, Keskin Y, Kocaoglu B, Hayran O. Macronutrient intake, physical activity, serum lipids and increased body weight in primary schoolchildren in Istanbul. Pediatr Int. 2005; 47(2): 159-66.

Manios Y, Yiannakouris N, Papoutsakis C, Moschonis G, Magkos F, Skenderi K, Zampelas A. Behavioral and physiological indices related to BMI in a cohort of primary schoolchildren in Greece. Am J Hum Biol. 2004; 16(6): 639-47.

Marcellino C, Henn RL, Olinto MT, Bressan AW, Paniz VM, Pattussi MP. Physical inactivity and associated factors among women from a municipality in southern Brazil. J Phys Act Health. 2014; 11(4): 777–83.

Margarita Algeria, James S. Jackson, Ronald C. Kessler, and David Takeuchi. Collaborative Psychiatric Epidemiology Surveys (CPES), 2001-2003 [United States]. ICPSR20240-v7. Ann Arbor, MI: Inter-university Consortium for Political and Social Research [distributor], 2015-05-28. http://doi.org/10.3886/ICPSR20240.v7

Marild S, Bondestam M, Bergström R, Ehnberg S, Hollsing A, Albertsson-Wikland K. Prevalence trends of obesity and overweight among 10-year-old children in western Sweden and relationship with parental body mass index. Acta Paediatr. 2004; 93(12): 1588-95.

Markwick A, Vaughan L, Ansari Z. Opposing socioeconomic gradients in overweight and obese adults. Aust N Z J Public Health. 2013; 37(1): 32-8.

Marques A, Gaspar De Matos M. Trends and correlates of overweight and obesity among adolescents from 2002 to 2010: a three-cohort study based on a representative sample of Portuguese adolescents. Am J Hum Biol. 2014; 26(6): 844–9.

Marques-Vidal P, Paccaud F, Ravasco P. Ten-year trends in overweight and obesity in the adult Portuguese population, 1995 to 2005. BMC Public Health. 2011; 772.

Martinez-Gomez D, Moreno LA, Romeo J, Rey-López JP, Castillo R, Cabero MJ, Vicente-Rodriguez G, Gutiérrez A, Veiga OL, Marcos A,. Combined Influence of Lifestyle Risk Factors on Body Fat in Spanish Adolescents – the AVENA Study. Obes Facts. 2011; 2011(4): 105-111.

Martínez-Vizcaíno V, Sánchez López M, Moya Martínez P, Solera Martinez M, Notario Pacheco B, Salcedo Aguilar F, Rodríguez-Artalejo F. Trends in excess weight and thinness among Spanish schoolchildren in the period 1992-2004: the Cuenca study. Public Health Nutr. 2009; 12(7): 1015-8.

Martinez-Vizcaino V, Solera-Martinez M, Cavero-Redondo I, Garcia-Prieto JC, Arias-Palencia N, Notario-Pacheco B, Martinez-Andres M, Mota J, Sanchez-Lopez M, Cuenca Study Group. Association between parental socioeconomic status with underweight and obesity in children from two Spanish birth cohorts: a changing relationship. BMC Public Health. 2015; 15: 1276.

Martín-López R, Pérez-Farinós N, Hernández-Barrera V, de Andres AL, Carrasco-Garrido P, Jiménez-García R. The association between excess weight and self-rated health and psychological distress in women in Spain. Public Health Nutr. 2011; 14(7): 1259-65.

Martinovic M, Belojevic G, Evans GW, Lausevic D, Asanin B, Samardzic M, Terzic N, Pantovic S, Jaksic M, Boljevic J. Prevalence of and contributing factors for overweight and obesity among Montenegrin schoolchildren. Eur J Public Health. 2015; 25(5): 833–9.

Maruf FA, Udoji NV. Prevalence and Socio-Demographic Determinants of Overweight and Obesity in a Nigerian Population. J Epidemiol. 2015; 25(7): 475–81. and Maruf FA, Akinpelu AO, Udoji NV. Differential perceptions of body image and body weight among adults of different socioeconomic status in a sub-urban population. J Biosoc Sci. 2014; 46(3): 279–93.

Massey University (New Zealand), University of Auckland (New Zealand), University of Otago (New Zealand). New Zealand National Children's Nutrition Survey 2002.

Matsushita Y, Takahashi Y, Mizoue T, Inoue M, Noda M, Tsugane S, JPHC Study Group. Overweight and obesity trends among Japanese adults: a 10-year follow-up of the JPHC Study. Int J Obes (Lond). 2008; 32(12): 1861-7.

Matthiasdottir E, Jonsson SH, Kristjansson AL. Body weight dissatisfaction in the Icelandic adult population: a normative discontent?. Eur J Public Health. 2012; 22(1): 116-21.

Matthiessen J, Biltoft-Jensen A, Fagt S, Knudsen VK, Tetens I, Groth MV. Misperception of body weight among overweight Danish adults: trends from 1995 to 2008. Public Health Nutr. 2014; 17(7): 1439–46.

Matthiessen J, Stockmarr A, Biltoft-Jensen A, Fagt S, Zhang H, Groth MV. Trends in overweight and obesity in Danish children and adolescents: 2000-2008--exploring changes according to parental education. Scand J Public Health. 2014; 42(4): 385–92.

Matusik P, Malecka-Tendera E, Klimek K, Polish Childhood Obesity Study Group. Nutritional state of Polish prepubertal children assessed by population-specific and international standards. Acta Paediatr. 2007; 96(2): 276-80.

Mauny F, Viel JF, Roubaux F, Ratsimandresy R, Sellin B. Blood pressure, body mass index and socio-economic status in the urban population of Antananarivo (Madagascar). Ann Trop Med Parasitol. 2003; 97(6): 645-54.

MBRRACE Register, National Perinatal Epidemiology Unit, University of Oxford. United Kingdom Saving Lives, Improving Mothers' Care 2014. National Perinatal Epidemiology Unit, University of Oxford, 2014.

McCrory C, Layte R. Breastfeeding and risk of overweight and obesity at nine-years of age. Soc Sci Med. 2012; 75(2): 323-30.

McDonald CM, Baylin A, Arsenault JE, Mora-Plazas M, Villamor E. Overweight is more prevalent than stunting and is associated with socioeconomic status, maternal obesity, and a snacking dietary pattern in school children from Bogota, Colombia. J Nutr. 2009; 139(2): 370-6.

McMaster C, Cullen L, Raymond N. Overweight and obesity in Irish primary schools: retrospective cohort study. Child Care Health Dev. 2005; 31(5): 499-506.

Medical Research Institute (Sri Lanka), United Nations Children's Fund (UNICEF), World Food Programme (WFP). Sri Lanka Nutrition and Food Security Assessment 2009.

Meng N-H, Li C-I, Liu C-S, Lin C-H, Lin W-Y, Chang C-K, Li T-C, Lin C-C. Comparison of height- and weight-adjusted sarcopenia in a Taiwanese metropolitan older population. Geriatr Gerontol Int. 2015; 15(1): 45–53.

Mengistu MD. Pattern of blood pressure distribution and prevalence of hypertension and prehypertension among adults in Northern Ethiopia: disclosing the hidden burden. BMC Cardiovasc Disord. 2014; 14: 33.

Menzies Research Institute Tasmania, Ministry of Health (Vietnam). Vietnam STEPS Noncommunicable Disease Risk Factors Survey 2009.

Minghelli B, Oliveira R, Nunes C. Association of obesity with chronic disease and musculoskeletal factors. Rev Assoc Med Bras (1992). 2015; 61(4): 347–54.

Minghelli B, Oliveira R, Nunes C. Non-specific low back pain in adolescents from the south of Portugal: prevalence and associated factors. J Orthop Sci. 2014; 19(6): 883–92.

Ministry of Economy, Finance, and Planning in charge of Privatization (Djibouti), Ministry of Health (Djibouti), United Nations Children's Fund (UNICEF). Djibouti Multiple Indicator Cluster Survey 2006. New York, United States: United Nations Children's Fund (UNICEF).

Ministry of Education and Sports (Laos), Ministry of Health (Laos), Ministry of Planning and Investment (Laos). Laos Multiple Indicator Cluster Survey 2011-2012. New York, United States: United Nations Children's Fund (UNICEF), 2013.

Ministry of Health (Argentina), National Institute of Statistics and Censuses (Argentina). Argentina National Survey of Risk Factors 2009.

Ministry of Health (Armenia), National Statistical Service (NSS), ORC Macro. Armenia Demographic and Health Survey 2000. Calverton, United States: Macro International, Inc.

Ministry of Health (Bahrain), World Health Organization (WHO). Bahrain STEPS Noncommunicable Disease Risk Factors Survey 2007.

Ministry of Health (Bahrain). Bahrain National Nutrition Survey 1998-1999.

Ministry of Health (Barbados). Barbados STEPS Noncommunicable Disease Risk Factors Survey 2007.

Ministry of Health (Benin), World Health Organization (WHO). Benin - Littoral STEPS Noncommunicable Disease Risk Factors Survey 2007.

Ministry of Health (Bhutan), World Health Organization (WHO). Bhutan - Thimphu STEPS Noncommunicable Disease Risk Factors Survey 2007.

Ministry of Health (Bhutan), World Health Organization (WHO). Bhutan STEPS Noncommunicable Disease Risk Factors Survey 2014.

Ministry of Health (Botswana), Ministry of Local Government (Botswana), World Health Organization (WHO). Botswana STEPS Noncommunicable Disease Risk Factors Survey 2007.

Ministry of Health (Brazil), Secretariat of Health Surveillance (Brazil), Secretariat of Health Care (Brazil). Brazil Risk Factor Morbidity Noncommunicable Disease Survey 2002-2005. Rio de Janeiro, Brazil: National Cancer Institute (Brazil).

Ministry of Health (Brazil), Secretariat of Health Surveillance, Ministry of Health (Brazil), University of São Paulo. Brazil Surveillance System of Risk Factors for Chronic Diseases by Telephone Interviews 2006.

Ministry of Health (Brazil), Secretariat of Health Surveillance, Ministry of Health (Brazil). Brazil Surveillance System of Risk Factors for Chronic Diseases by Telephone Interviews 2007.

Ministry of Health (Brazil), Secretariat of Health Surveillance, Ministry of Health (Brazil). Brazil Surveillance System of Risk Factors for Chronic Diseases by Telephone Interviews 2008.

Ministry of Health (Brazil), Secretariat of Health Surveillance, Ministry of Health (Brazil). Brazil Surveillance System of Risk Factors for Chronic Diseases by Telephone Interviews 2009.

Ministry of Health (Brazil), Secretariat of Health Surveillance, Ministry of Health (Brazil). Brazil Surveillance System of Risk Factors for Chronic Diseases by Telephone Interviews 2010.

Ministry of Health (Brazil), Secretariat of Health Surveillance, Ministry of Health (Brazil). Brazil Surveillance System of Risk Factors for Chronic Diseases by Telephone Interviews 2011.

Ministry of Health (Brazil), Secretariat of Health Surveillance, Ministry of Health (Brazil). Brazil Surveillance System of Risk Factors for Chronic Diseases by Telephone Interviews 2012.

Ministry of Health (Cambodia), University of Health Sciences (Cambodia), World Health Organization (WHO). Cambodia STEPS Noncommunicable Disease Risk Factors Survey 2010.

Ministry of Health (Chile). Chile National Health Survey 2009-2010.

Ministry of Health (China), National Center for Chronic and Noncommunicable Disease Control and Prevention (China), World Health Organization (WHO). China WHO Study on Global AGEing and Adult Health 2007-2010.

Ministry of Health (Colombia), Regional Population Center (Colombia), Westinghouse; Institute for Resource Development. Colombia Demographic and Health Survey 1986. Columbia, United States: Westinghouse; Institute for Resource Development.

Ministry of Health (Congo, DR), World Health Organization (WHO). Congo, DR - Kinshasa STEPS Noncommunicable Disease Risk Factors Survey 2005.

Ministry of Health (Congo, Rep.), World Health Organization (WHO). Congo, Rep. - Brazzaville STEPS Noncommunicable Disease Risk Factors Survey 2004.

Ministry of Health (Côte d'Ivoire), World Health Organization (WHO). Côte d'Ivoire - Lagunes STEPS Noncommunicable Disease Risk Factors Survey 2005.

Ministry of Health (Eritrea), World Health Organization (WHO). Eritrea STEPS Noncommunicable Disease Risk Factors Survey 2004.

Ministry of Health (Indonesia), World Health Organization (WHO). Indonesia - Jawa Barat STEPS Noncommunicable Disease Risk Factors Survey 2006.

Ministry of Health (Indonesia), World Health Organization (WHO). Indonesia STEPS Noncommunicable Disease Risk Factors Survey 2001.

Ministry of Health (Italy), National Association of Hospital Cardiologists (Italy), National Center for Epidemiology, Surveillance, and Health Promotion (CNESPS) (Italy), National Institute of Health (Italy). Italy Health Examination Survey 2008-2012.

Ministry of Health (Italy), National Association of Hospital Cardiologists (Italy), National Institute of Health (Italy). Italy Cardiovascular Epidemiologic Observatory 1998-2002.

Ministry of Health (Italy), National Institute of Statistics (Italy). Italy Health Conditions, Risk Factors and Use of Health Services Survey 2004-2005.

Ministry of Health (Jordan), World Health Organization (WHO). Jordan STEPS Noncommunicable Disease Risk Factors Survey 2004.

Ministry of Health (Kuwait), World Health Organization (WHO). Kuwait STEPS Noncommunicable Disease Risk Factors Survey 2006.

Ministry of Health (Kuwait), World Health Organization (WHO). Kuwait STEPS Noncommunicable Disease Risk Factors Survey 2014.

Ministry of Health (Laos), National Institute of Public Health (NIOPH), National Statistical Center (Laos), United Nations Children's Fund (UNICEF). Laos Multiple Indicator Cluster Survey 2000. New York, United States: United Nations Children's Fund (UNICEF).

Ministry of Health (Laos), National Institute of Public Health (NIOPH), National Statistical Center (Laos). Laos National Health Survey 2000.

Ministry of Health (Laos), World Health Organization (WHO). Laos - Viangchan STEPS Noncommunicable Disease Risk Factors Survey 2008.

Ministry of Health (Malawi), World Health Organization (WHO). Malawi STEPS Noncommunicable Disease Risk Factors Survey 2009.

Ministry of Health (Malaysia), World Health Organization (WHO). Malaysia STEPS Noncommunicable Disease Risk Factors Survey 2005-2006.

Ministry of Health (Mauritania), World Health Organization (WHO). Mauritania - Nouakchott STEPS Noncommunicable Disease Risk Factors Survey 2006.

Ministry of Health (Mexico), Secretary of Public Education (Mexico). Mexico National School Health Survey 2008. Cuernavaca, Mexico: National Institute of Public Health (Mexico), 2010.

Ministry of Health (MOH) (Ghana), Ghana Statistical Service and United Nations Children's Fund (UNICEF). Ghana Multiple Indicator Cluster Survey 2006. New York, United States: United Nations Children's Fund (UNICEF).

Ministry of Health (Moldova), National Bureau of Statistics (Moldova), National Center of Public Health (Moldova), World Health Organization (WHO). Moldova STEPS Noncommunicable Disease Risk Factors Survey 2013.
Ministry of Health (Moldova), National Bureau of Statistics (Moldova), United Nations Children's Fund (UNICEF). Moldova Multiple Indicator Cluster Survey 2012. New York, United States: United Nations Children's Fund (UNICEF), 2015.
Ministry of Health (Mozambique), World Health Organization (WHO). Mozambique STEPS Noncommunicable Disease Risk Factors Survey 2005.
Ministry of Health (Myanmar), United Nations Children's Fund (UNICEF). Myanmar Multiple Indicator Cluster Survey 2000. New York, United States: United Nations Children's Fund (UNICEF).
Ministry of Health (Myanmar), World Health Organization (WHO). Myanmar - Yangon STEPS Noncommunicable Disease Risk Factors Survey 2003.
Ministry of Health (Myanmar), World Health Organization Regional Office for South-East Asia (SEARO). Myanmar STEPS Noncommunicable Disease Risk Factors Survey 2009.
Ministry of Health (New Zealand), National Research Bureau Ltd (New Zealand), Statistics New Zealand. New Zealand Health Survey 2002-2004.
Ministry of Health (New Zealand), National Research Bureau Ltd (New Zealand). New Zealand Health Survey 2006-2007.
Ministry of Health (New Zealand), University of Otago (New Zealand). New Zealand National Nutrition Survey 1996-1997.
Ministry of Health (New Zealand). New Zealand Tracking the Obesity Epidemic 1977-2003. Wellington, New Zealand: Ministry of Health (New Zealand), 2004.
Ministry of Health (Niger), World Health Organization (WHO). Niger STEPS Noncommunicable Disease Risk Factors Survey 2007.
Ministry of Health (Nigeria), Nigerian Heart Foundation, World Health Organization (WHO). Nigeria - Lagos STEPS Noncommunicable Disease Risk Factors Survey 2003.
Ministry of Health (Oman), World Health Organization (WHO). Oman - Ash Sharqīyah STEPS Noncommunicable Disease Risk Factors Survey 2006.
Ministry of Health (Oman), World Health Organization (WHO). Oman World Health Survey 2007-2008.
Ministry of Health (Palestine), Palestinian Central Bureau of Statistics, United Nations Children's Fund (UNICEF), United Nations Population Fund (UNFPA). Palestine - West Bank and Gaza Strip Multiple Indicator Cluster Survey 2000 - UNICEF. Ramallah, Palestine: Palestinian Central Bureau of Statistics.
Ministry of Health (Palestine), Palestinian Central Bureau of Statistics, United Nations Children's Fund (UNICEF), United Nations Population Fund (UNFPA). Palestine Multiple Indicator Cluster Survey 2010. New York, United States: United Nations Children's Fund (UNICEF), 2014.
Ministry of Health (Palestine), Palestinian Central Bureau of Statistics, United Nations Children's Fund (UNICEF). Palestine - West Bank and Gaza Strip Multiple Indicator Cluster Survey 1996. Ramallah, Palestine: Palestinian Central Bureau of Statistics.
Ministry of Health (Palestine), Palestinian Central Bureau of Statistics, United Nations Children's Fund (UNICEF). Palestine Multiple Indicator Cluster Survey 2014. New York, United States: United Nations Children's Fund (UNICEF), 2015.
Ministry of Health (Palestine), World Health Organization (WHO). Palestine STEPS Noncommunicable Disease Risk Factors Survey 2010-2011. Geneva, Switzerland: World Health Organization (WHO).
Ministry of Health (Panama). Panama Living Standards Measurement Survey Obesity Estimates, Children and Adults 1982, 1997, 2003.
Ministry of Health (Russian Federation). Russia - Moscow Behavioral Risk Factor Survey 2000-2001.

Ministry of Health (Saint Lucia), World Health Organization (WHO). Saint Lucia STEPS Noncommunicable Disease Risk Factors Survey 2012.
Ministry of Health (Samoa). Samoa STEPS Noncommunicable Disease Risk Factors Survey 2002.
Ministry of Health (Saudi Arabia), World Health Organization (WHO). Saudi Arabia STEPS Noncommunicable Disease Risk Factors Survey 2004-2005.
Ministry of Health (Seychelles), World Health Organization (WHO). Seychelles STEPS Noncommunicable Disease Risk Factors Survey 2013-2014.
Ministry of Health (Singapore). Singapore National Health Surveillance Survey 2007.
Ministry of Health (Singapore). Singapore National Health Survey 1998.
Ministry of Health (Singapore). Singapore National Health Survey 2004.
Ministry of Health (Singapore). Singapore National Health Survey 2010.
Ministry of Health (Sri Lanka), World Health Organization (WHO). Sri Lanka STEPS Noncommunicable Disease Risk Factors Survey 2006.
Ministry of Health (Swaziland), World Health Organization (WHO). Swaziland STEPS Noncommunicable Disease Risk Factors Survey 2007.
Ministry of Health (Swaziland), World Health Organization (WHO). Swaziland STEPS Noncommunicable Disease Risk Factors Survey 2014.
Ministry of Health (Syria), World Health Organization (WHO). Syria WHO Multi-country Survey Study on Health and Health System Responsiveness 2000-2001.
Ministry of Health (Tajikistan), United Nations Children's Fund (UNICEF). Tajikistan Micronutrient Status Survey 2009.
Ministry of Health (Timor-Leste), National University of East Timor, World Health Organization (WHO). Timor-Leste STEPS Noncommunicable Disease Risk Factors Survey 2014.
Ministry of Health (Togo), West African Health Organization, World Health Organization (WHO). Togo STEPS Noncommunicable Disease Risk Factors Survey 2010-2011.
Ministry of Health (Tonga), World Health Organization (WHO). Tonga STEPS Noncommunicable Disease Risk Factors Survey 2004.
Ministry of Health (Trinidad and Tobago). Trinidad and Tobago Adult Health Survey 2001.
Ministry of Health (Turkey). Turkey Chronic Diseases and Risk Factors Study 2011.
Ministry of Health (Uganda), World Health Organization (WHO). Uganda STEPS Noncommunicable Disease Risk Factors Survey 2014.
Ministry of Health (Uzbekistan), World Bank, World Health Organization (WHO). Uzbekistan STEPS Noncommunicable Disease Risk Factors Survey 2014.
Ministry of Health (Vanuatu), Secretariat of the Pacific Community (SPC), Vanuatu National Statistics Office. Vanuatu Demographic and Health Survey 2013.
Ministry of Health (Vanuatu), Secretariat of the Pacific Community (SPC). Vanuatu Noncommunicable Disease Survey 1998.
Ministry of Health (Vanuatu), United Nations Children's Fund (UNICEF). Vanuatu Multiple Indicator Cluster Survey 2007-2008. New York, United States: United Nations Children's Fund (UNICEF).
Ministry of Health (Vanuatu), World Health Organization (WHO). Vanuatu STEPS Noncommunicable Disease Risk Factors Survey 2011.
Ministry of Health (Zambia), World Health Organization (WHO). Zambia - Lusaka STEPS Noncommunicable Disease Risk Factors Survey 2008.
Ministry of Health (Zanzibar), World Health Organization (WHO). Tanzania - Zanzibar STEPS Noncommunicable Disease Risk Factors Survey 2011.
Ministry of Health and Child Welfare (Zimbabwe), University of Zimbabwe, World Health Organization (WHO). Zimbabwe STEPS Noncommunicable Disease Risk Factors Survey 2005.

Ministry of Health and Family Planning (Madagascar), World Health Organization (WHO). Madagascar - Antananarivo and Toliara STEPS Noncommunicable Disease Risk Factors Survey 2005.
Ministry of Health and Medical Education (Iran), World Health Organization (WHO). Iran STEPS Noncommunicable Disease Risk Factors Survey 2006.
Ministry of Health and Medical Education (Iran), World Health Organization (WHO). Iran STEPS Noncommunicable Disease Risk Factors Survey 2009.
Ministry of Health and Medical Education (Iran), World Health Organization (WHO). Iran WHO Multi-country Survey Study on Health and Health System Responsiveness 2000-2001. Geneva, Switzerland: World Health Organization (WHO).
Ministry of Health and Medical Services (Kiribati), World Health Organization (WHO). Kiribati STEPS Noncommunicable Disease Risk Factors Survey 2004-2006.
Ministry of Health and Population (Egypt), USAID, World Health Organization (WHO). Egypt STEPS Noncommunicable Disease Risk Factors Survey 2005.
Ministry of Health and Population (Egypt), USAID, World Health Organization (WHO). Egypt STEPS Noncommunicable Disease Risk Factors Survey 2011-2012.
Ministry of Health and Population (Nepal), Nepal Health Research Council (NHRC), World Health Organization (WHO). Nepal STEPS Noncommunicable Disease Risk Factors Survey 2012-2013.
Ministry of Health and Population (Nepal), Society for Local Integrated Development Nepal, World Health Organization (WHO). Nepal STEPS Noncommunicable Disease Risk Factors Survey 2007.
Ministry of Health and Prevention (Senegal), Research Center for Human Development (Senegal). Senegal Demographic and Health Survey 2005. Calverton, United States: Macro International, Inc.
Ministry of Health and Public Hygiene (Gabon), World Health Organization (WHO). Gabon - Estuaire STEPS Noncommunicable Disease Risk Factors Survey 2009.
Ministry of Health and Quality of Life (Mauritius), World Health Organization (WHO). Mauritius STEPS Noncommunicable Disease Risk Factors Survey 2004.
Ministry of Health and Sanitation (Sierra Leone), World Health Organization (WHO). Sierra Leone STEPS Noncommunicable Disease Risk Factors Survey 2009.
Ministry of Health and Social Welfare (Lesotho), World Health Organization (WHO). Lesotho STEPS Noncommunicable Disease Risk Factors Survey 2012 .
Ministry of Health and Welfare (Andorra). Andorra National Health Survey 1997.
Ministry of Health and Welfare (Andorra). Andorra National Health Survey 2002.
Ministry of Health and Welfare (Andorra). Andorra National Health Survey 2011.
Ministry of Health and Welfare (Andorra). Andorra National Survey of Children's Health 2004.
Ministry of Health and Welfare (Japan), National Institute of Health and Nutrition (Japan). Japan National Nutrition Survey 1999.
Ministry of Health and Welfare (Japan). Japan National Nutrition Survey 1980.
Ministry of Health and Welfare (Japan). Japan National Nutrition Survey 1981.
Ministry of Health and Welfare (Japan). Japan National Nutrition Survey 1982.
Ministry of Health and Welfare (Japan). Japan National Nutrition Survey 1983.
Ministry of Health and Welfare (Japan). Japan National Nutrition Survey 1984.
Ministry of Health and Welfare (Japan). Japan National Nutrition Survey 1985.
Ministry of Health and Welfare (Japan). Japan National Nutrition Survey 1986.
Ministry of Health and Welfare (Japan). Japan National Nutrition Survey 1987.
Ministry of Health and Welfare (Japan). Japan National Nutrition Survey 1988.
Ministry of Health and Welfare (Japan). Japan National Nutrition Survey 1989.
Ministry of Health and Welfare (Japan). Japan National Nutrition Survey 1990.
Ministry of Health and Welfare (Japan). Japan National Nutrition Survey 1991.

Ministry of Health and Welfare (Japan). Japan National Nutrition Survey 1992.
Ministry of Health and Welfare (Japan). Japan National Nutrition Survey 1993.
Ministry of Health and Welfare (Japan). Japan National Nutrition Survey 1994.
Ministry of Health and Welfare (Japan). Japan National Nutrition Survey 1995.
Ministry of Health and Welfare (Japan). Japan National Nutrition Survey 1996.
Ministry of Health and Welfare (Japan). Japan National Nutrition Survey 1997.
Ministry of Health and Welfare (Japan). Japan National Nutrition Survey 1998.
Ministry of Health and Welfare (Japan). Japan National Nutrition Survey 2000.
Ministry of Health and Welfare (Japan). Japan National Nutrition Survey 2001.
Ministry of Health and Welfare (Taiwan). Taiwan National Health Interview Survey and Drug Abuse Body Mass Index Estimates 2009.
Ministry of Health, Labour and Welfare (Japan). Japan National Nutrition Survey 2002.
Ministry of Health, Population and Hospital Reform (Algeria), World Health Organization (WHO). Algeria - Sétif and Mostaganem STEPS Noncommunicable Disease Risk Factors Survey 2003.
Ministry of Health, Social Services, and Equality (Spain), National Statistics Institute (Spain). Spain National Health Survey 2006-2007.
Ministry of Health, Social Services, and Equality (Spain), National Statistics Institute (Spain). Spain National Health Survey 2011-2012.
Ministry of Health, Social Services, and Equality (Spain). Spain National Health Survey 1987.
Ministry of Health, Social Services, and Equality (Spain). Spain National Health Survey 1993.
Ministry of Health, Social Services, and Equality (Spain). Spain National Health Survey 1995.
Ministry of Health, Social Services, and Equality (Spain). Spain National Health Survey 1997.
Ministry of Health, Social Services, and Equality (Spain). Spain National Health Survey 2001.
Ministry of Health, Welfare, and Sport (Netherlands), National Institute for Public Health and the Environment (Netherlands). Netherlands National Food Consumption Survey - Older Adults 2010-2012.
Ministry of Health, Welfare, and Sport (Netherlands), National Institute for Public Health and the Environment (Netherlands). Netherlands National Food Consumption Survey 2007-2010.
Ministry of Health, Welfare, and Sport (Netherlands). Netherlands National Food Consumption Survey Young Children 2005-2006.
Ministry of Planning and Reconstruction (Congo, DR), United Nations Children's Fund (UNICEF). Congo, DR Multiple Indicator Cluster Survey 2001. New York, United States: United Nations Children's Fund (UNICEF).
Ministry of Planning, Economic Development and Public Investment (Equatorial Guinea), United Nations Children's Fund (UNICEF). Equatorial Guinea Multiple Indicator Cluster Survey 2000. New York, United States: United Nations Children's Fund (UNICEF).
Ministry of Public Health (Chad), World Health Organization (WHO). Chad - Ville de N'Djaména STEPS Noncommunicable Disease Risk Factors Survey 2008.
Ministry of Public Health (Cuba), National Office of Statistics (Cuba), United Nations Children's Fund (UNICEF). Cuba Multiple Indicator Cluster Survey 2014. New York, United States: United Nations Children's Fund (UNICEF), 2005.
Ministry of Public Health (Ecuador), National Institute of Statistics and Censuses (Ecuador). Ecuador National Health and Nutrition Survey 2012.
Ministry of Public Health (Lebanon), World Health Organization (WHO). Lebanon WHO Multi-country Survey Study on Health and Health System Responsiveness 2000-2001.
Ministry of Public Health (Morocco), Westinghouse; Institute for Resource Development. Morocco Demographic and Health Survey 1987. Columbia, United States: Westinghouse; Institute for Resource Development.

Ministry of Public Health (North Korea), World Health Organization (WHO). Korea, North STEPS Noncommunicable Disease Risk Factors Survey 2007.

Ministry of Public Health (North Korea), World Health Organization (WHO). Korea, North STEPS Noncommunicable Disease Risk Factors Survey 2008.

Ministry of Public Health (Thailand). Thailand National Health and Examination Survey 2003-2004.

Ministry of Public Health (Thailand). Thailand Noncommunicable Disease and Injury Behavior Risk Surveillance Survey 2007.

Ministry of Public Health (Uruguay), World Health Organization (WHO). Uruguay STEPS Noncommunicable Disease Risk Factors Survey 2006.

Ministry of Public Health and Welfare (Paraguay), Pan American Health Organization (PAHO), Spanish Agency for International Development Cooperation (AECID). Paraguay STEPS Noncommunicable Disease Risk Factors Survey 2011.

Ministry of Statistics and Analysis of the Republic of Belarus, United Nations Children's Fund (UNICEF). Belarus Multiple Indicator Cluster Survey 2005. New York, United States: United Nations Children's Fund (UNICEF).

Mintem GC, Horta BL, Domingues MR, Gigante DP. Body size dissatisfaction among young adults from the 1982 Pelotas birth cohort. Eur J Clin Nutr. 2015; 69(1): 55–61.

Mitchell RT, McDougall CM, Crum JE. Decreasing prevalence of obesity in primary schoolchildren. Arch Dis Child. 2007; 92(2): 153-4.

Mitra and Associates, ORC Macro. Bangladesh Demographic and Health Survey 2004. Calverton, United States: ORC Macro.

Moges B, Amare B, Fantahun B, Kassu A. High prevalence of overweight, obesity, and hypertension with increased risk to cardiovascular disorders among adults in northwest Ethiopia: a cross sectional study. BMC Cardiovasc Disord. 2014; 14: 155. and Amare B, Moges B, Moges F, Fantahun B, Admassu M, Mulu A, Kassu A. Nutritional status and dietary intake of urban residents in Gondar, Northwest Ethiopia. BMC Public Health. 2012; 12: 752.

MOH Center for Health Information and Statistics, Macro International, Inc, National Statistical Service (NSS). Armenia Demographic and Health Survey 2005. Calverton, United States: Macro International, Inc.

Monyeki KD, Kemper HCG, Makgae PJ. Relationship between fat patterns, physical fitness and blood pressure of rural South African children: Ellisras Longitudinal Growth and Health Study. J Hum Hypertens. 2008; 22(5): 311-9.

Moraeus L, Lissner L, Sjoberg A. Stable prevalence of obesity in Swedish schoolchildren from 2008 to 2013 but widening socio-economic gap in girls. Acta Paediatr. 2014; 103(12): 1277–84.

Moreira P, Padez C, Mourão I, Rosado V. Dietary calcium and body mass index in Portuguese children. Eur J Clin Nutr. 2005; 59(7): 861-7.

Moreira P, Padrão P. Educational, economic and dietary determinants of obesity in Portuguese adults: a cross-sectional study. Eat Behav. 2006; 7(3): 220-8.

Morley BC, Scully ML, Niven PH, Okely AD, Baur LA, Pratt IS, Wakefield MA, NaSSDA Study Team. What factors are associated with excess body weight in Australian secondary school students. Med J Aust. 2012; 196(3): 189-92.

Mota J, Fidalgo F, Silva R, Ribeiro JC, Santos R, Carvalho J, Santos MP. Relationships between physical activity, obesity and meal frequency in adolescents. Ann Hum Biol. 2008; 35(1): 1-10.

Mourtakos SP, Tambalis KD, Panagiotakos DB, Antonogeorgos G, Arnaoutis G, Karteroliotis K, Sidossis LS. Maternal lifestyle characteristics during pregnancy, and the risk of obesity in the offspring: a study of 5,125 children. BMC Pregnancy Childbirth. 2015; 15: 66.

Muhammad NA, Omar K, Shah SA, Muthupalaniappen LAP, Arshad F. Parental perception of their children's weight status, and its association with their nutrition and obesity knowledge. Asia Pac J Clin Nutr. 2008; 17(4): 597-602.

Muniz J, Hervada J, Juane R, Lopez-Rodriguez I, Castro-Beiras A. Prevalence of diabetes mellitus in the population aged 40-69 years in Galicia, northwest Spain. Diabetes Res Clin Pract. 1995; 30(2): 137-42.

Musaiger AO, Al-Mannai MA. Weight, height, body mass index and prevalence of obesity among the adult population in Bahrain. Ann Hum Biol. 2001; 28(3): 346-50.

Mwaikambo SA, Leyna GH, Killewo J, Simba A, Puoane T. Why are primary school children overweight and obese? A cross sectional study undertaken in Kinondoni district, Dar-es-salaam. BMC Public Health. 2015; 15: 1269.

Nabipour I, Amiri M, Imami SR, Jahfari SM, Nosrati A, Iranpour D, Soltanian AR. Unhealthy lifestyles and ischaemic electrocardiographic abnormalities: the Persian Gulf Healthy Heart Study. East Mediterr Health J. 2008; 14(4): 858-68.

Nagel G, Wabitsch M, Galm C, Berg S, Brandstetter S, Fritz M, Klenk J, Peter R, Prokopchuk D, Steiner R, Stroth S, Wartha O, Weiland SK, Steinacker J. Determinants of obesity in the Ulm Research on Metabolism, Exercise and Lifestyle in Children (URMEL-ICE). Eur J Pediatr. 2009; 168(10): 1259-67.

Nakano T, Sei M, Ewis AA, Munakata H, Onishi C, Nakahori Y. Tracking overweight and obesity in Japanese children; a six years longitudinal study. J Med Invest. 2010; 57(1-2): 114-23.

Namibia Statistics Agency. Namibia Household Income and Expenditure Survey 2009-2010. Windhoek, Namibia: Namibia Statistics Agency.

NatCen Social Research and Royal Free and University College Medical School. Department of Epidemiology and Public Health, Health Survey for England, 2010 [computer file]. 2nd Edition. Colchester, Essex: UK Data Archive [distributor], July 2012. SN: 6986, http://dx.doi.org/10.5255/UKDA-SN-6986-2

NatCen Social Research and University College London. Department of Epidemiology and Public Health, Health Survey for England, 2011 [computer file]. Colchester, Essex: UK Data Archive [distributor], April 2013. SN: 7260, http://dx.doi.org/10.5255/UKDA-SN-7260-1

NatCen Social Research and University College London. Department of Epidemiology and Public Health, Health Survey for England, 2013 [computer file]. Colchester, Essex: UK Data Archive [distributor], January 2015. SN: 7649, http://dx.doi.org/10.5255/UKDA-SN-7649-1

NatCen Social Research et al. , Diet and Nutrition Survey of Infants and Young Children, 2011 [computer file]. 2nd Edition. Colchester, Essex: UK Data Archive [distributor], October 2013. SN: 7263 , http://dx.doi.org/10.5255/UKDA-SN-7263-2.

NatCen Social Research, Welsh Health Survey, 2012 [computer file]. Colchester, Essex: UK Data Archive [distributor], February 2014. SN: 7459, http://dx.doi.org/10.5255/UKDA-SN-7459-1.

NatCen Social Research, Welsh Health Survey, 2011 [computer file]. Colchester, Essex: UK Data Archive [distributor], January 2013. SN: 7188, http://dx.doi.org/10.5255/UKDA-SN-7188-1.

National Bureau of Statistics (Nigeria), United Nations Children's Fund (UNICEF). Nigeria Multiple Indicator Cluster Survey 2011. New York, United States: United Nations Children's Fund (UNICEF), 2013.

National Center for Disease Control (Georgia), State Department of Statistics of Georgia, United Nations Children's Fund (UNICEF). Georgia Multiple Indicator Cluster Survey 2005. New York, United States: United Nations Children's Fund (UNICEF).

National Center for Health Statistics (NCHS), Centers for Disease Control and Prevention (CDC), SRA International, Inc., United States Census Bureau. United States National Hospital Ambulatory Medical Care Survey 2010. Hyattsville, United States: National Center for Health Statistics (NCHS), Centers for Disease Control and Prevention (CDC).

National Center for Health Statistics (NCHS), Centers for Disease Control and Prevention (CDC), University of Michigan, Institute for Social Research, Eunice Shriver Kennedy National Institute of Child Health and Human Development (NICHD), Office of Population Affairs (OPA), Office of the Assistant Secretary for Planning and Evaluation (OASPE), Children's Bureau of the Administration of Children and Families (ACF). United States National Survey of Family Growth 2006-2010. Hyattsville, United States: National Center for Health Statistics (NCHS), Centers for Disease Control and Prevention (CDC), 2011.

National Center for Health Statistics (NCHS), Centers for Disease Control and Prevention (CDC), US Census Bureau. United States National Health Interview Survey 1980. Hyattville, United States: National Center for Health Statistics (NCHS), Centers for Disease Control and Prevention (CDC).

National Center for Health Statistics (NCHS), Centers for Disease Control and Prevention (CDC), US Census Bureau. United States National Health Interview Survey 1981. Hyattsville, United States: National Center for Health Statistics (NCHS), Centers for Disease Control and Prevention (CDC).

National Center for Health Statistics (NCHS), Centers for Disease Control and Prevention (CDC), US Census Bureau. United States National Health Interview Survey 1982. Hyattsville, United States: National Center for Health Statistics (NCHS), Centers for Disease Control and Prevention (CDC).

National Center for Health Statistics (NCHS), Centers for Disease Control and Prevention (CDC), US Census Bureau. United States National Health Interview Survey 1983. Hyattville, United States: National Center for Health Statistics (NCHS), Centers for Disease Control and Prevention (CDC).

National Center for Health Statistics (NCHS), Centers for Disease Control and Prevention (CDC), US Census Bureau. United States National Health Interview Survey 1984. Hyattsville, United States: National Center for Health Statistics (NCHS), Centers for Disease Control and Prevention (CDC).

National Center for Health Statistics (NCHS), Centers for Disease Control and Prevention (CDC), US Census Bureau. United States National Health Interview Survey 1985. Hyattsville, United States: National Center for Health Statistics (NCHS), Centers for Disease Control and Prevention (CDC).

National Center for Health Statistics (NCHS), Centers for Disease Control and Prevention (CDC), US Census Bureau. United States National Health Interview Survey 1986. Hyattsville, United States: National Center for Health Statistics (NCHS), Centers for Disease Control and Prevention (CDC).

National Center for Health Statistics (NCHS), Centers for Disease Control and Prevention (CDC), US Census Bureau. United States National Health Interview Survey 1987. Hyattsville, United States: National Center for Health Statistics (NCHS), Centers for Disease Control and Prevention (CDC).

National Center for Health Statistics (NCHS), Centers for Disease Control and Prevention (CDC), US Census Bureau. United States National Health Interview Survey 1988. Hyattsville, United States: National Center for Health Statistics (NCHS), Centers for Disease Control and Prevention (CDC).

National Center for Health Statistics (NCHS), Centers for Disease Control and Prevention (CDC), US Census Bureau. United States National Health Interview Survey 1989. Hyattsville, United States: National Center for Health Statistics (NCHS), Centers for Disease Control and Prevention (CDC).

National Center for Health Statistics (NCHS), Centers for Disease Control and Prevention (CDC), US Census Bureau. United States National Health Interview Survey 1990. Hyattsville, United States: National Center for Health Statistics (NCHS), Centers for Disease Control and Prevention (CDC).

National Center for Health Statistics (NCHS), Centers for Disease Control and Prevention (CDC), US Census Bureau. United States National Health Interview Survey 1991. Hyattsville, United States: National Center for Health Statistics (NCHS), Centers for Disease Control and Prevention (CDC).

National Center for Health Statistics (NCHS), Centers for Disease Control and Prevention (CDC), US Census Bureau. United States National Health Interview Survey 1992. Hyattsville, United States: National Center for Health Statistics (NCHS), Centers for Disease Control and Prevention (CDC).

National Center for Health Statistics (NCHS), Centers for Disease Control and Prevention (CDC), US Census Bureau. United States National Health Interview Survey 1993. Hyattville, United States: National Center for Health Statistics (NCHS), Centers for Disease Control and Prevention (CDC).

National Center for Health Statistics (NCHS), Centers for Disease Control and Prevention (CDC), US Census Bureau. United States National Health Interview Survey 1994. Hyattsville, United States: National Center for Health Statistics (NCHS), Centers for Disease Control and Prevention (CDC).

National Center for Health Statistics (NCHS), Centers for Disease Control and Prevention (CDC), US Census Bureau. United States National Health Interview Survey 1995. Hyattsville, United States: National Center for Health Statistics (NCHS), Centers for Disease Control and Prevention (CDC).

National Center for Health Statistics (NCHS), Centers for Disease Control and Prevention (CDC), US Census Bureau. United States National Health Interview Survey 1996. Hyattsville, United States: National Center for Health Statistics (NCHS), Centers for Disease Control and Prevention (CDC).

National Center for Health Statistics (NCHS), Centers for Disease Control and Prevention (CDC), US Census Bureau. United States National Health Interview Survey 1997. Hyattsville, United States: National Center for Health Statistics (NCHS), Centers for Disease Control and Prevention (CDC).

National Center for Health Statistics (NCHS), Centers for Disease Control and Prevention (CDC), US Census Bureau. United States National Health Interview Survey 1998. Hyattsville, United States: National Center for Health Statistics (NCHS), Centers for Disease Control and Prevention (CDC).

National Center for Health Statistics (NCHS), Centers for Disease Control and Prevention (CDC), US Census Bureau. United States National Health Interview Survey 1999. Hyattsville, United States: National Center for Health Statistics (NCHS), Centers for Disease Control and Prevention (CDC).

National Center for Health Statistics (NCHS), Centers for Disease Control and Prevention (CDC), US Census Bureau. United States National Health Interview Survey 2000. Hyattsville, United States: National Center for Health Statistics (NCHS), Centers for Disease Control and Prevention (CDC).

National Center for Health Statistics (NCHS), Centers for Disease Control and Prevention (CDC), US Census Bureau. United States National Health Interview Survey 2001. Hyattsville, United States: National Center for Health Statistics (NCHS), Centers for Disease Control and Prevention (CDC).

National Center for Health Statistics (NCHS), Centers for Disease Control and Prevention (CDC), US Census Bureau. United States National Health Interview Survey 2002. Hyattsville, United States: National Center for Health Statistics (NCHS), Centers for Disease Control and Prevention (CDC).

National Center for Health Statistics (NCHS), Centers for Disease Control and Prevention (CDC), US Census Bureau. United States National Health Interview Survey 2003. Hyattsville, United States: National Center for Health Statistics (NCHS), Centers for Disease Control and Prevention (CDC).

National Center for Health Statistics (NCHS), Centers for Disease Control and Prevention (CDC), US Census Bureau. United States National Health Interview Survey 2004. Hyattsville, United States: National Center for Health Statistics (NCHS), Centers for Disease Control and Prevention (CDC).

National Center for Health Statistics (NCHS), Centers for Disease Control and Prevention (CDC), US Census Bureau. United States National Health Interview Survey 2005. Hyattsville, United States: National Center for Health Statistics (NCHS), Centers for Disease Control and Prevention (CDC).

National Center for Health Statistics (NCHS), Centers for Disease Control and Prevention (CDC), US Census Bureau. United States National Health Interview Survey 2006. Hyattsville, United States: National Center for Health Statistics (NCHS), Centers for Disease Control and Prevention (CDC).

National Center for Health Statistics (NCHS), Centers for Disease Control and Prevention (CDC), US Census Bureau. United States National Health Interview Survey 2007. Hyattsville, United States: National Center for Health Statistics (NCHS), Centers for Disease Control and Prevention (CDC).

National Center for Health Statistics (NCHS), Centers for Disease Control and Prevention (CDC), US Census Bureau. United States National Health Interview Survey 2008. Hyattsville, United States: National Center for Health Statistics (NCHS), Centers for Disease Control and Prevention (CDC).

National Center for Health Statistics (NCHS), Centers for Disease Control and Prevention (CDC), US Census Bureau. United States National Health Interview Survey 2009. Hyattsville, United States: National Center for Health Statistics (NCHS), Centers for Disease Control and Prevention (CDC).

National Center for Health Statistics (NCHS), Centers for Disease Control and Prevention (CDC), US Census Bureau. United States National Health Interview Survey 2010. Hyattsville, United States: National Center for Health Statistics (NCHS), Centers for Disease Control and Prevention (CDC).

National Center for Health Statistics (NCHS), Centers for Disease Control and Prevention (CDC), US Census Bureau. United States National Health Interview Survey 2011. Hyattsville, United States: National Center for Health Statistics (NCHS), Centers for Disease Control and Prevention (CDC).

National Center for Health Statistics (NCHS), Centers for Disease Control and Prevention (CDC). United States National Health and Nutrition Examination Survey 1988-1994. Hyattsville, United States: National Center for Health Statistics (NCHS), Centers for Disease Control and Prevention (CDC).

National Center for Health Statistics (NCHS), Centers for Disease Control and Prevention (CDC). United States National Health and Nutrition Examination Survey 1999-2000. Hyattsville, United States: National Center for Health Statistics (NCHS), Centers for Disease Control and Prevention (CDC).

National Center for Health Statistics (NCHS), Centers for Disease Control and Prevention (CDC). United States National Health and Nutrition Examination Survey 2001-2002. Hyattsville, United States: National Center for Health Statistics (NCHS), Centers for Disease Control and Prevention (CDC).

National Center for Health Statistics (NCHS), Centers for Disease Control and Prevention (CDC). United States National Health and Nutrition Examination Survey 2003-2004. Hyattsville, United States: National Center for Health Statistics (NCHS), Centers for Disease Control and Prevention (CDC).

National Center for Health Statistics (NCHS), Centers for Disease Control and Prevention (CDC). United States National Health and Nutrition Examination Survey 2005-2006. Hyattsville, United States: National Center for Health Statistics (NCHS), Centers for Disease Control and Prevention (CDC), 2007.

National Center for Health Statistics (NCHS), Centers for Disease Control and Prevention (CDC). United States National Health and Nutrition Examination Survey 2007-2008. Hyattsville, United States: National Center for Health Statistics (NCHS), Centers for Disease Control and Prevention (CDC), 2009.

National Center for Health Statistics (NCHS), Centers for Disease Control and Prevention (CDC). United States National Health and Nutrition Examination Survey 2009-2010. Hyattsville, United States: National Center for Health Statistics (NCHS), Centers for Disease Control and Prevention (CDC), 2011.

National Center for Health Statistics (NCHS), Centers for Disease Control and Prevention (CDC). United States National Health and Nutrition Examination Survey 2011-2012. Hyattsville, United States: National Center for Health Statistics (NCHS), Centers for Disease Control and Prevention (CDC), 2013.

National Center for Health Statistics (NCHS), Centers for Disease Control and Prevention (CDC). United States National Health and Nutrition Examination Survey 2013-2014. Hyattsville, United States: National Center for Health Statistics (NCHS), Centers for Disease Control and Prevention (CDC).

National Centre for Social Research and University College London. Department of Epidemiology and Public Health, Health Survey for England, 2001 [computer file]. 2nd Edition. Colchester, Essex: UK Data Archive [distributor], June 2004. SN: 4628.

National Centre for Social Research and University College London. Department of Epidemiology and Public Health, Health Survey for England, 2002 [computer file]. Colchester, Essex: UK Data Archive [distributor], May 2004. SN: 4912.

National Centre for Social Research and University College London. Department of Epidemiology and Public Health, Health Survey for England, 2003 [computer file]. Colchester, Essex: UK Data Archive [distributor], March 2005. SN: 5098.

National Centre for Social Research and University College London. Department of Epidemiology and Public Health, Health Survey for England, 2004 [computer file]. Colchester, Essex: UK Data Archive [distributor], July 2006. SN: 5439.

National Centre for Social Research and University College London. Department of Epidemiology and Public Health, Health Survey for England, 2006 [computer file]. 4th Edition. Colchester, Essex: UK Data Archive [distributor], July 2011. SN: 5809, http://dx.doi.org/10.5255/UKDA-SN-5809-1

National Centre for Social Research and University College London. Department of Epidemiology and Public Health, Health Survey for England, 2007 [computer file]. 2nd Edition. Colchester, Essex: UK Data Archive [distributor], April 2010. SN: 6112, http://dx.doi.org/10.5255/UKDA-SN-6112-1

National Centre for Social Research and University College London. Department of Epidemiology and Public Health, Health Survey for England, 2008 [computer file]. 3rd Edition. Colchester, Essex: UK Data Archive [distributor], July 2011. SN: 6397, http://dx.doi.org/10.5255/UKDA-SN-6397-1

National Centre for Social Research and University College London. Department of Epidemiology and Public Health, Health Survey for England, 2009 [computer file]. 2nd Edition. Colchester, Essex: UK Data Archive [distributor], July 2011. SN: 6732, http://dx.doi.org/10.5255/UKDA-SN-6732-1

National Centre for Social Research and University College London. Department of Epidemiology and Public Health, Health Survey for England, 1999 [computer file]. 3rd Edition. Colchester, Essex: UK Data Archive [distributor], February 2002. SN: 4365.

National Centre for Social Research and University College London. Department of Epidemiology and Public Health, Health Survey for England, 2005 [computer file]. Colchester, Essex: UK Data Archive [distributor], July 2007. SN: 5675.

National Centre for Social Research and University of Leicester, Adult Psychiatric Morbidity Survey, 2007 [computer file]. 3rd Edition. Colchester, Essex: UK Data Archive [distributor], January 2011. SN: 6379, http://dx.doi.org/10.5255/UKDA-SN-6379-1

National Centre for Social Research et al. , National Survey of Sexual Attitudes and Lifestyles II, 2000-2001 [computer file]. Colchester, Essex: UK Data Archive [distributor], August 2005. SN: 5223, http://dx.doi.org/10.5255/UKDA-SN-5223-1

National Centre for Social Research et al., Low Income Diet and Nutrition Survey, 2003-2005 [computer file]. Colchester, Essex: UK Data Archive [distributor], March 2008. SN: 5808, http://dx.doi.org/10.5255/UKDA-SN-5808-1.

National Centre for Social Research, Welsh Health Survey, 2005-2006 [computer file]. 2nd Edition. Colchester, Essex: UK Data Archive [distributor], February 2011. SN: 5750 , http://dx.doi.org/10.5255/UKDA-SN-5750-1

National Centre for Social Research, Welsh Health Survey, 2007 [computer file]. 2nd Edition. Colchester, Essex: UK Data Archive [distributor], February 2011. SN: 6052, http://dx.doi.org/10.5255/UKDA-SN-6052-1.

National Centre for Social Research, Welsh Health Survey, 2008 [computer file]. 2nd Edition. Colchester, Essex: UK Data Archive [distributor], February 2011. SN: 6372.

National Centre for Social Research, Welsh Health Survey, 2009 [computer file]. 2nd Edition. Colchester, Essex: UK Data Archive [distributor], February 2011. SN: 6589, http://dx.doi.org/10.5255/UKDA-SN-6589-1.

National Centre for Social Research, Welsh Health Survey, 2010 [computer file]. Colchester, Essex: UK Data Archive [distributor], November 2011. SN: 6895, http://dx.doi.org/10.5255/UKDA-SN-6895-1.

National Centre for Social Research, Beaufort Research Limited and University College London. Department of Epidemiology and Public Health, Welsh Health Survey, 2003-2004 [computer file]. 2nd Edition. Colchester, Essex: UK Data Archive [distributor], February 2011. SN: 5692 , http://dx.doi.org/10.5255/UKDA-SN-5692-1

National Centre for Social Research, Beaufort Research Limited and University College London. Department of Epidemiology and Public Health, Welsh Health Survey, 2004-2005 [computer file]. 2nd Edition. Colchester, Essex: UK Data Archive [distributor], February 2011. SN: 5693, http://dx.doi.org/10.5255/UKDA-SN-5693-1.

National Centre for Social Research, University College London Department of Epidemiology and Public Health, Health Survey for England, 1998 [computer file]. 4th ed. Colchester, Essex: UK Data Archive [distributor], 30 November 2002. SN: 4150.

National Centre for Social Research, University College London Department of Epidemiology and Public Health, Health Survey for England, 2000 [computer file]. Colchester, Essex: UK Data Archive [distributor], 23 April 2002. SN: 4487.

National Council for Population and Family (Dominican Republic), Westinghouse; Institute for Resource Development. Dominican Republic Demographic and Health Survey 1986. Columbia, United States: Westinghouse; Institute for Resource Development.

National Epidemiology Center (Hungary), National Institute for Food and Nutrition Science (Hungary). Hungary National Population Health Survey 2003. Budapest, Hungary: National Epidemiology Center (Hungary).

National Food Agency (Sweden), Statistics Sweden. Sweden National Food Consumption Survey 2010-2011 [Unpublished].

National Food and Nutrition Centre (Fiji). Fiji National Nutrition Survey 2004.

National Health Examination Survey Office (Thailand). Thailand National Health and Examination Survey 2008-2009.

National Institute for Health and Welfare (Finland). Finland Children and Young People's Health Follow up Study (LATE) 2007-2009.

National Institute for Health and Welfare (Finland). Finland Health Behavior and Health Among the Adult Population 2008.

National Institute for Health and Welfare (Finland). Finland Health Behavior and Health Among the Adult Population 2009.

National Institute for Health and Welfare (Finland). Finland Health Behavior and Health Among the Adult Population 2010.

National Institute for Health Development (Estonia). Estonia Health Behavior Among the Adult Population 2004.

National Institute for Health Development (Estonia). Estonia Health Behavior Among the Adult Population 2006.

National Institute for Health Development (Estonia). Estonia Health Behavior Among the Adult Population 2008.

National Institute for Health Development (Estonia). Estonia Health Behavior Among the Adult Population 2010.

National Institute for Health Development (Estonia). Estonia Health Behavior Among the Adult Population 2012.

National Institute for Health Development (Estonia). Estonia Health Interview Survey 2006-2007.

National Institute for Medical Research (Tanzania), World Health Organization (WHO). Tanzania STEPS Noncommunicable Disease Risk Factors Survey 2012.

National Institute for Public Health and the Environment (Netherlands), Statistics Netherlands. Netherlands Risk Factors and Health Survey 1998-2001.

National Institute for Public Health and the Environment (Netherlands). Netherlands Measurement Survey 2009-2010.

National Institute of Health (Italy). Italy Behavioral Risk Factor Surveillance System 2010.

National Institute of Health (Italy). Italy Behavioral Risk Factor Surveillance System 2011.
National Institute of Health (Italy). Italy Behavioral Risk Factor Surveillance System 2012.
National Institute of Health (Italy). Italy Behavioral Risk Factor Surveillance System 2013.
National Institute of Health (Portugal), Statistics Portugal. Portugal National Health Survey 2005-2006.
National Institute of Health Research and Development (NIHRD), Ministry of Health (Indonesia), World Health Organization (WHO). Indonesia WHO Multi-country Survey Study on Health and Health System Responsiveness 2001.
National Institute of Health Research and Development (NIHRD), Ministry of Health (Indonesia). Indonesia Basic Health Research 2010.
National Institute of Public Health (Mexico), National Institute of Statistics, Geography, and Informatics (Mexico). Mexico National Nutrition Survey 1998-1999.
National Institute of Public Health (Mexico), World Health Organization (WHO). Mexico WHO Multi-country Survey Study on Health and Health System Responsiveness 2000-2001. Geneva, Switzerland: World Health Organization (WHO).
National Institute of Public Health (Mexico), World Health Organization (WHO). Mexico WHO Study on Global AGEing and Adult Health 2009-2010. Geneva, Switzerland: World Health Organization (WHO), 2011.
National Institute of Public Health (Mexico). Mexico National Health Survey 1999-2000.
National Institute of Public Health (Mexico). Mexico National Nutrition Survey 1988. Cuernavaca, Mexico: National Institute of Public Health (Mexico).
National Institute of Public Health (Mexico). Mexico National Survey of Health and Nutrition 2005-2006. Cuernavaca, Mexico: National Institute of Public Health (Mexico).
National Institute of Public Health (Mexico). Mexico National Survey of Health and Nutrition 2011-2012. Cuernavaca, Mexico: National Institute of Public Health (Mexico).
National Institute of Statistics (Albania), United Nations Children's Fund (UNICEF). Albania Multiple Indicator Cluster Survey 2000. New York, United States: United Nations Children's Fund (UNICEF).
National Institute of Statistics (Albania), United Nations Children's Fund (UNICEF). Albania Multiple Indicator Cluster Survey 2005. New York, United States: United Nations Children's Fund (UNICEF).
National Institute of Statistics (Albania), World Bank (WB). Albania Living Standards Measurement Survey 2002. Washington DC, United States: World Bank (WB).
National Institute of Statistics (Angola), United Nations Children's Fund (UNICEF). Angola Multiple Indicator Cluster Survey 2001. New York, United States: United Nations Children's Fund (UNICEF).
National Institute of Statistics (Cambodia). Cambodia Anthropometrics Survey 2008 - CamNut. Phnom Penh, Cambodia: National Institute of Statistics (Cambodia).
National Institute of Statistics (Italy). Italy Aspects of Daily Life 2002.
National Institute of Statistics (Italy). Italy Aspects of Daily Life 2003.
National Institute of Statistics (Italy). Italy Aspects of Daily Life 2005.
National Institute of Statistics (Italy). Italy Aspects of Daily Life 2006.
National Institute of Statistics (Italy). Italy Aspects of Daily Life 2007.
National Institute of Statistics (Italy). Italy Aspects of Daily Life 2008.
National Institute of Statistics (Italy). Italy Aspects of Daily Life 2009.
National Institute of Statistics (Italy). Italy Aspects of Daily Life 2010.
National Institute of Statistics (Italy). Italy Aspects of Daily Life 2011.
National Institute of Statistics (Italy). Italy Aspects of Daily Life 2012.
National Institute of Statistics (Italy). Italy Health Conditions and Use of Health Services Survey 1999-2000.
National Institute of Statistics and Censuses (Nicaragua), World Bank. Nicaragua Living Standards Measurement Survey 2005.

National Institute of Statistics and Demography (Burkina Faso), World Bank. Burkina Faso Core Welfare Indicators Questionnaire Survey 2003. Ouagadougou, Burkina Faso: National Institute of Statistics and Demography (Burkina Faso).
National Institute of Statistics and Demography (Burkina Faso), World Bank. Burkina Faso Core Welfare Indicators Questionnaire Survey 2005. Ouagadougou, Burkina Faso: National Institute of Statistics and Demography (Burkina Faso).
National Institute of Statistics and Economic Analysis (INSAE) (Benin), National Program Against AIDS (PNLS) (Benin), Macro International, Inc. Benin Demographic and Health Survey 2006. Calverton, United States: Macro International, Inc.
National Institute of Statistics and Economic Analysis (INSAE) (Benin), ORC Macro. Benin Demographic and Health Survey 2001. Calverton, United States: ORC Macro.
National Institute of Statistics, Geography, and Informatics (Mexico), Population Studies Center, University of Pennsylvania, University of Maryland, University of Wisconsin. Mexico Health and Aging Study 2001.
National Institute of Statistics, Geography, and Informatics (Mexico), Population Studies Center, University of Pennsylvania, University of Maryland, University of Wisconsin. Mexico Health and Aging Study 2003.
National Institute of Statistics, Geography, and Informatics (Mexico), Population Studies Center, University of Pennsylvania, University of Maryland, University of Wisconsin. Mexico Health and Aging Study 2012. Mexico City, México: National Institute of Statistics, Geography, and Informatics (Mexico).
National Institute on Alcohol Abuse and Alcoholism (NIAAA), National Institutes of Health (NIH), US Department of Health and Human Services. United States National Epidemiologic Survey on Alcohol and Related Conditions 2001-2002 [Unpublished].
National Institute on Alcohol Abuse and Alcoholism (NIAAA), National Institutes of Health (NIH), US Department of Health and Human Services. United States National Epidemiologic Survey on Alcohol and Related Conditions 2004-2005 [Unpublished].
National Nutrition Monitoring Bureau (India). India Rural Second Repeat Survey of Diet and Nutritional Status 1996-1997.
National Nutrition Monitoring Bureau (India). India Rural Survey of Diet and Nutritional Status 1994.
National Nutrition Monitoring Bureau (India). India Rural Survey of Diet and Nutritional Status 2000-2001.
National Nutrition Monitoring Bureau (India). India Rural Survey of Diet and Nutritional Status 2004-2005.
National Nutrition Monitoring Bureau (India). India Rural Third Repeat Survey of Diet and Nutritional Status 2011-2012.
National Nutrition Monitoring Bureau (India). India Tribal First Repeat Survey of Diet and Nutritional Status 1998-1999.
National Nutrition Monitoring Bureau (India). India Urban Slums Survey of Diet and Nutritional Status 1993-1994.
National Office of Statistics (Mauritania), United Nations Children's Fund (UNICEF). Mauritania Multiple Indicator Cluster Survey 2007. New York, United States: United Nations Children's Fund (UNICEF).
National Office of Statistics (Mauritania), United Nations Children's Fund (UNICEF). Mauritania Multiple Indicator Cluster Survey 2011. New York, United States: United Nations Children's Fund (UNICEF), 2015.
National Population Commission of Nigeria, ORC Macro, UK Department for International Development (DFID), United Nations Children's Fund (UNICEF), United Nations Population Fund (UNFPA). Nigeria Demographic and Health Survey 2003. Calverton, United States: ORC Macro.
National Public Health Institute (Finland). Finland Health Behavior and Health Among the Adult Population 2001.
National Public Health Institute (Finland). Finland Health Behavior and Health Among the Adult Population 2002.

National Public Health Institute (Finland). Finland Health Behavior and Health Among the Adult Population 2003.

National Public Health Institute (Finland). Finland Health Behavior and Health Among the Adult Population 2004.

National Public Health Institute (Finland). Finland Health Behavior and Health Among the Adult Population 2005.

National Public Health Institute (Finland). Finland Health Behavior and Health Among the Adult Population 2006.

National Public Health Institute (Finland). Finland Health Behavior and Health Among the Adult Population 2007.

National Public Health Institute (Finland). Latvia Health Behavior Among the Adult Population 2006.

National Statistical Committee of the Kyrgyz Republic, United Nations Children's Fund (UNICEF). Kyrgyzstan Multiple Indicator Cluster Survey 2014. New York, United States: United Nations Children's Fund (UNICEF), 2015.

National Statistical Institute (Congo, DR), Ministry of Planning (Congo, DR), United Nations Children's Fund (UNICEF). Congo, DR Multiple Indicator Cluster Survey 2010. New York, United States: United Nations Children's Fund (UNICEF).

National Statistical Office (Thailand), United Nations Children's Fund (UNICEF). Thailand Multiple Indicator Cluster Survey 2005-2006. New York, United States: United Nations Children's Fund (UNICEF).

National Statistical Office of Malawi, United Nations Children's Fund (UNICEF). Malawi Multiple Indicator Cluster Survey 2013-2014. New York, United States: United Nations Children's Fund (UNICEF), 2015.

National Statistical Office of Malawi, World Bank. Malawi Living Standards Measurement Survey 2010-2011. Washington DC, United States: World Bank.

National Statistical Office of Mongolia, United Nations Children's Fund (UNICEF). Mongolia Multiple Indicator Cluster Survey 2005. New York, United States: United Nations Children's Fund (UNICEF).

National Statistical Office of Mongolia, United Nations Children's Fund (UNICEF). Mongolia Multiple Indicator Cluster Survey 2010. New York, United States: United Nations Children's Fund (UNICEF), 2013.

National Statistical Office of Mongolia. Mongolia Multiple Indicator Cluster Survey 2000. New York, United States: United Nations Children's Fund (UNICEF).

National Statistics Bureau (Bhutan), United Nations Children's Fund (UNICEF), United Nations Population Fund (UNFPA). Bhutan Multiple Indicator Cluster Survey 2010. New York, United States: United Nations Children's Fund (UNICEF).

National Statistics Institute (Spain). Spain National Health Survey 2003.

National Statistics Office (Malta). Malta Lifestyle Survey 2003.

National Statistics Office (Malta). Malta Lifestyle Survey 2006-2007.

National Team for the Acceleration of Poverty Reduction (TNP2K) (Indonesia), SurveyMETER, University of Southern California, World Bank. Indonesia Family Life Survey East 2012.

Navia B, Aparicio A, Perea JM, Perez-Farinos N, Villar-Villalba C, Labrado E, Ortega RM. Sodium intake may promote weight gain; results of the FANPE study in a representative sample of the adult Spanish population. Nutr Hosp. 2014; 29(6): 1283–9.

Nemesure B, Wu S-Y, Hennis A, Leske MC. Nine-year incidence of obesity and overweight in an African-origin population. Int J Obes (Lond). 2008; 32(2): 329-35.

Ng N. Preventable risk factors for noncommunicable diseases in rural Indonesia:prevalence study using WHO STEPS approach. Bull World Health Organ. 2006; 2006(4): 305-13.

Nielsen TL, Wraae K, Brixen K, Hermann AP, Andersen M, Hagen C. Prevalence of overweight, obesity and physical inactivity in 20- to 29-year-old, Danish men. Relation to sociodemography, physical dysfunction and low socioeconomic status: the Odense Androgen Study. Int J Obes (Lond). 2006; 30(5): 805-15.

Niizeki T, Takeishi Y, Takabatake N, Shibata Y, Konta T, Kato T, Kawata S, Kubota I. Circulating levels of heart-type fatty acid-binding protein in a general Japanese population: effects of age, gender, and physiologic characteristics. Circ J. 2007; 71(9): 1452-7.

Niven P, Scully M, Morley B, Baur L, Crawford D, Pratt IS, Wakefield M, NaSSDA Study Team. What factors are associated with frequent unhealthy snack-food consumption among Australian secondary-school students?. Public Health Nutr. 2015; 18(12): 2153–60.

Niwa Y, Yatsuya H, Tamakoshi K, Nishio K, Kondo T, Lin Y, Suzuki S, Wakai K, Tokudome S, Yamamoto A, Hamajima N, Toyoshima H, Tamakoshi A, JACC Study Group. Relationship between body mass index and the risk of ovarian cancer in the Japanese population: findings from the Japanese Collaborate Cohort (JACC) study. J Obstet Gynaecol Res. 2005; 31(5): 452-8.

Norboo T, Stobdan T, Tsering N, Angchuk N, Tsering P, Ahmed I, Chorol T, Kumar Sharma V, Reddy P, Singh SB, Kimura Y, Sakamoto R, Fukutomi E, Ishikawa M, Suwa K, Kosaka Y, Nose M, Yamaguchi T, Tsukihara T, Matsubayashi K, Otsuka K, Okumiya K. Prevalence of hypertension at high altitude: cross-sectional survey in Ladakh, Northern India 2007-2011. BMJ Open. 2015; 5(4): e007026.

Northern Ireland Statistics and Research Agency (NISRA). United Kingdom - Northern Ireland Health and Social Wellbeing Survey 2005-2006 - NISRA.

Northern Ireland Statistics and Research Agency. Central Survey Unit, Northern Ireland Health and Social Wellbeing Survey, 1997 [computer file]. Colchester, Essex: UK Data Archive [distributor], October 2002. SN: 4589, http://dx.doi.org/10.5255/UKDA-SN-4589-1.

Northern Ireland Statistics and Research Agency. Central Survey Unit, Northern Ireland Health and Social Wellbeing Survey, 2005-2006 [computer file]. Colchester, Essex: UK Data Archive [distributor], October 2007. SN: 5710, http://dx.doi.org/10.5255/UKDA-SN-5710-1.

Nova E, Martinez-Gomez D, Gomez-Martinez S, Veses AM, Calle ME, Veiga OL, Marcos A. Influence of health behaviours on the incidence of infection and allergy in adolescents: the AFINOS cross-sectional study. BMC Public Health. 2014; 14: 19.

O’Dea JA, Chiang H, Peralta LR. Socioeconomic patterns of overweight, obesity but not thinness persist from childhood to adolescence in a 6-year longitudinal cohort of Australian schoolchildren from 2007 to 2012. BMC Public Health. 2014; 14: 222.

O’Dea JA, Dibley MJ. Prevalence of obesity, overweight and thinness in Australian children and adolescents by socioeconomic status and ethnic cultural group in 2006 and 2012. Int J Public Health. 2014; 59(5): 819–28.

O'Dea JA, Amy NK. Perceived and desired weight, weight related eating and exercising behaviours, and advice received from parents among thin, overweight, obese or normal weight Australian children and adolescents. Int J Behav Nutr Phys Act. 2011; 68.

Oellingrath IM, Svendsen MV, Brantsaeter AL. Eating patterns and overweight in 9- to 10-year-old children in Telemark County, Norway: a cross-sectional study. Eur J Clin Nutr. 2010; 64(11): 1272-9.

Office for National Statistics. Social and Vital Statistics Division and Food Standards Agency, National Diet and Nutrition Survey : Adults Aged 19 to 64 Years, 2000-2001 [computer file]. Colchester, Essex: UK Data Archive [distributor], May 2005. SN: 5140 , http://dx.doi.org/10.5255/UKDA-SN-5140-1.

Office of Population Censuses and Surveys. Social Survey Division, Dietary and Nutritional Survey of British Adults, 1986-1987 [computer file]. Colchester, Essex: UK Data Archive [distributor], September 1991. SN: 2836, http://dx.doi.org/10.5255/UKDA-SN-2836-1.

Office of Population Censuses and Surveys. Social Survey Division, Health Survey for England, 1991-1992: Combined Data File. Colchester, Essex: UK Data Archive [distributor], October 1997. SN: 3238, http://dx.doi.org/10.5255/UKDA-SN-3238-1

Office of Population Censuses and Surveys. Social Survey Division, Health Survey for England, 1993 [Computer file]. Colchester, Essex: UK Data Archive [distributor], April 1995. SN: 3316, http://dx.doi.org/10.5255/UKDA-SN-3316-1

Oguz A, Temizhan A, Abaci A, Kozan O, Erol C, Ongen Z, Celik S. Obesity and abdominal obesity; an alarming challenge for cardio-metabolic risk in Turkish adults. Anatol J Cardiol. 2008; 8(6): 401-6.

Olafsdottir LB, Gudjonsson H, Jonsdottir HH, Thjodleifsson B. Natural history of heartburn: a 10-year population-based study. World J Gastroenterol. 2011; 17(5): 639-45.

Oliveira AM, Oliveira AC, Almeida MS, Oliveira N, Adan L. Influence of the family nucleus on obesity in children from northeastern Brazil: a cross-sectional study. BMC Public Health. 2007; 235.

Omega Contract Research Organization, Turkish Society of Hypertension and Renal Diseases. Turkey Prevalence, Awareness, Treatment and Control of Hypertension Study 2003. Ankara, Turkey: Turkish Society of Hypertension and Renal Diseases.

Omega Contract Research Organization, Turkish Society of Hypertension and Renal Diseases. Turkey Salt Consumption and Blood Pressure Study 2007.

Önal AE, Erbil S, Özel S, Aciksari K, Tumerdem Y. The prevalence of and risk factors for hypertension in adults living in Istanbul. Blood Press. 2004; 13(1): 31-6.

Oner N, Vatansever U, Sari A, Ekuklu E, Güzel A, Karasalihoglu S, Boris NW. Prevalence of underweight, overweight and obesity in Turkish adolescents. Swiss Med Wkly. 2004; 134(35-36): 529-33.

Ostrowska-Nawarycz L, Nawarycz T. Prevalence of excessive body weight and high blood pressure in children and adolescents in the city of Łódź. Kardiol Pol. 2007; 65(9): 1079-1087.

Otani T, Iwasaki M, Inoue M, Shoichiro Tsugane for the Japan Public Health Center-based Prospective Study Group. Body mass index, body height, and subsequent risk of colorectal cancer in middle-aged and elderly Japanese men and women: Japan public health center-based prospective study. Cancer Causes Control. 2005; 16(7): 839–50.

Ozer BK. Growth reference centiles and secular changes in Turkish children and adolescents. Econ Hum Biol. 2007; 5(2): 280-301.

Ozmen D, Ozmen E, Ergin D, Cetinkaya AC, Sen N, Dundar PE, Taskin EO. The association of self-esteem, depression and body satisfaction with obesity among Turkish adolescents. BMC Public Health. 2007; 80.

Pagano R, La Vecchia C. Overweight and obesity in Italy, 1990-91. Int J Obes Relat Metab Disord. 1994; 18(10): 665-9.

Pagano R, Negri E, Decarli A, La Vecchia C. Smoking and Weight in the 1983 Italian National Health Survey. Int J Obes (Lond). 1987; 11(4): 333-8.

Palestinian Central Bureau of Statistics, United Nations Children's Fund (UNICEF). Palestinians in Lebanon Multiple Indicator Cluster Survey 2011. New York, United States: United Nations Children's Fund (UNICEF), 2013.

Palestinian Central Bureau of Statistics. Palestine Demographic and Health Survey 2004.

Palomo G I, Icaza N G, Mujica E V, Núñez F L, Leiva M E, Vásquez R M, Alarcón L M, Moyano D E. Prevalencia de factores de riesgo cardiovascular clásicos en población adulta de Talca, Chile, 2005. Rev Med Chil. 2007; 135(7): 904-12.

Pan American Health Organization (PAHO), Center for Demography and Ecology, University of Wisconsin-Madison, Inter-University Consortium for Political and Social Research (ICPSR), Centre for Population and Development Studies, National Statistics Office (Cuba), Iberoamerican Center for the Third Age. Cuba - Havana Survey on Health, Well-Being, and Aging in Latin America and the Caribbean 1999-2000. Ann Arbor, United States: Inter-University Consortium for Political and Social Research (ICPSR).

Pan American Health Organization (PAHO), Center for Demography and Ecology, University of Wisconsin-Madison, Inter-University Consortium for Political and Social Research (ICPSR), College of the Northern Border (COLEF), Research in Health and Demographics (INSAD), National Institute of Medical Sciences and Nutrition Salvador Zubirán. Mexico - Mexico City Survey on Health, Well-Being, and Aging in Latin America and the Caribbean 1999-2000. Ann Arbor, United States: Inter-University Consortium for Political and Social Research (ICPSR).

Pan American Health Organization (PAHO), Center for Demography and Ecology, University of Wisconsin-Madison, Inter-University Consortium for Political and Social Research (ICPSR), Institute of Nutrition and Food Technology (INTA), University of Chile, Center for Geriatrics and Gerontology, Pontifical Catholic University of Chile. Chile - Santiago Survey on Health, Well-Being, and Aging in Latin America and the Caribbean 1999-2000. Ann Arbor, United States: Inter-University Consortium for Political and Social Research (ICPSR).

Pan American Health Organization (PAHO), Center for Demography and Ecology, University of Wisconsin-Madison, Inter-University Consortium for Political and Social Research (ICPSR), University of São Paulo. Brazil - São Paulo Survey on Health, Well-Being, and Aging in Latin America and the Caribbean 1999-2000. Ann Arbor, United States: Inter-University Consortium for Political and Social Research (ICPSR).

Pan American Health Organization (PAHO), World Health Organization (WHO). Grenada STEPS Noncommunicable Disease Risk Factors Survey 2010-2011.

Pan American Health Organization (PAHO). Costa Rica - San Jose Diabetes, Hypertension, and Noncommunicable Disease Risk Factors Survey 2004.

Pan American Health Organization (PAHO). Honduras - Tegucigalpa Diabetes, Hypertension, and Non-Communicable Disease Risk Factors Survey 2003-2004.

Pan Arab Project for Family Health (PAPFAM), United Nations Children's Fund (UNICEF). Somalia Multiple Indicator Cluster Survey 2006. New York, United States: United Nations Children's Fund (UNICEF).

Pandey RM, Gupta R, Misra A, Misra P, Singh V, Agrawal A, Dey S, Rao S, Menon VU, Kamalamma N, Devi KPV, Revathi K, Sharma V. Determinants of urban-rural differences in cardiovascular risk factors in middle-aged women in India: a cross-sectional study. Int J Cardiol. 2013; 163(2): 157-62.

Panel Study of Income Dynamics, 1999 public use dataset. Produced and distributed by the University of Michigan with primary funding from the National Science Foundation, the National Institute of Aging, and the National Institute of Child Health and Human Development. Ann Arbor, MI, (2011).

Panel Study of Income Dynamics, 2001 public use dataset. Produced and distributed by the University of Michigan with primary funding from the National Science Foundation, the National Institute of Aging, and the National Institute of Child Health and Human Development. Ann Arbor, MI, (2011).

Panel Study of Income Dynamics, 2003 public use dataset. Produced and distributed by the University of Michigan with primary funding from the National Science Foundation, the National Institute of Aging, and the National Institute of Child Health and Human Development. Ann Arbor, MI, (2011).

Panel Study of Income Dynamics, 2005 public use dataset. Produced and distributed by the University of Michigan with primary funding from the National Science Foundation, the National Institute of Aging, and the National Institute of Child Health and Human Development. Ann Arbor, MI, (2011).

Panel Study of Income Dynamics, 2007 public use dataset. Produced and distributed by the University of Michigan with primary funding from the National Science Foundation, the National Institute of Aging, and the National Institute of Child Health and Human Development. Ann Arbor, MI, (2011).

Pang W, Sun Z, Zhang X, Liu S, Xu C, Li J, Sun Y, Zheng L, Li J, Hu D. Body mass index and the prevalence of prehypertension and hypertension in a Chinese rural population. Intern Med. 2008; 47(10): 893-7.

Papadimitriou A, Kounadi D, Konstantinidou M, Xepapadaki P, Nicolaidou P. Prevalence of obesity in elementary schoolchildren living in Northeast Attica, Greece. Obesity (Silver Spring). 2006; 14(7): 1113-7.

Pasquet P, Temgoua LS, Melaman-Sego F, Froment A, Rikong-Adié H. Prevalence of overweight and obesity for urban adults in Cameroon. Ann Hum Biol. 2003; 30(5): 551-62.

Patil YP, Shinde RL. Undernutrition among Indian men: a study based on NFHS-3. Am J Men Health. 2014; 8(6): 492–502.

Patterson KAE, Cleland V, Venn A, Blizzard L, Gall S. A cross-sectional study of geographic differences in health risk factors among young Australian adults: the role of socioeconomic position. BMC Public Health. 2014; 14: 1278.

Patton GC, Coffey C, Carlin JB, Sawyer SM, Williams J, Olsson CA, Wake M. Overweight and obesity between adolescence and young adulthood: a 10-year prospective cohort study. J Adolesc Health. 2011; 48(3): 275-80.

Pavlica T, Rakic R, Sakac D. Anthropometric indices of obesity and potential health risk in adult rural population from Backa and Banat--the Republic of Serbia. Coll Antropol. 2014; 38(1): 227–33.

Péneau S, Rouchaud A, Rolland-Cachera M-F, Arnault N, Hercberg S, Castetbon K. Body size and growth from birth to 2 years and risk of overweight at 7-9 years. Int J Pediatr Obes. 2011; 6(2-2): e162-169.

Péneau S, Salanave B, Rolland-Cachera M-F, Hercberg S, Castetbon K. Correlates of sedentary behavior in 7 to 9-year-old French children are dependent on maternal weight status. Int J Obes (Lond). 2011; 35(7): 907-15.

Pereira M, Lunet N, Paulo C, Severo M, Azevedo A, Barros H. Incidence of hypertension in a prospective cohort study of adults from Porto, Portugal. BMC Cardiovasc Disord. 2012; 114.

Pérez CM, Guzmán M, Ortiz AP, Estrella M, Valle Y, Pérez N, Haddock L, Suárez E. Prevalence of the metabolic syndrome in San Juan, Puerto Rico. Ethn Dis. 2008; 18(4): 434-41.

Perry CP, Keane E, Layte R, Fitzgerald AP, Perry IJ, Harrington JM. The use of a dietary quality score as a predictor of childhood overweight and obesity. BMC Public Health. 2015; 15: 581.

Pimenta AM, Kac G, Gazzinelli A, Corrêa-Oliveira R, Velásquez-Meléndez G. Association between central obesity, triglycerides and hypertension in a rural area in Brazil. Arq Bras Cardiol. 2008; 90(6): 386-92.

Pirkola J, Tammelin T, Bloigu A, Pouta A, Laitinen J, Ruokonen A, Tapanainen P, Järvelin M-R, Vääräsmäki M. Prevalence of metabolic syndrome at age 16 using the International Diabetes Federation paediatric definition. Arch Dis Child. 2008; 93(11): 945-51.

Pliatskidou S, Samakouri M, Kalamara E, Papageorgiou E, Koutrouvi K, Goulemtzakis C, Nikolaou E, Livaditis M. Validity of the Greek Eating Disorder Examination Questionnaire 6.0 (EDE-Q-6.0) among Greek adolescents. Psychiatriki. 2015; 26(3): 204–16.

Pomerleau J, Pudule I, Grinberga D, Kadziauskiene K, Abaravicius A, Bartkeviciute R, Vaask S, Robertson A, McKee M. Patterns of body weight in the Baltic Republics. Public Health Nutr. 2000; 3(1): 3-10.

Pomeroy E, Stock JT, Stanojevic S, Miranda JJ, Cole TJ, Wells JCK. Stunting, adiposity, and the individual-level dual burden among urban lowland and rural highland Peruvian children. Am J Hum Biol. 2014; 26(4): 481–90.

Pontificia Universidad Javeriana (Colombia), World Health Organization (WHO). Colombia WHO Multi-country Survey Study on Health and Health System Responsiveness 2000-2001. Geneva, Switzerland: World Health Organization (WHO).

Population Department, Ministry of the Interior (Burundi), Westinghouse; Institute for Resource Development. Burundi Demographic and Health Survey 1987. Columbia, United States: Westinghouse; Institute for Resource Development.

Pu C, Chou Y-J. Health ratings for underweight, overweight and obese adolescents: disparities between adolescent's own report and the parent's report. Asia Pac J Clin Nutr. 2010; 19(2): 180-7.

Public Health and Reforms Center, Ministry of Health (PHRC) (Azerbaijan). Azerbaijan Demographic and Health Survey 2011.

Public Health Authority of the Slovak Republic, World Health Organization (WHO). Slovakia WHO Multi-country Survey Study on Health and Health System Responsiveness 2000.

Public Health Authority of the Slovak Republic. Slovakia National Anthropometric Survey 2001.

Public Health Institute of Federation of Bosnia and Herzegovina. Bosnia and Herzegovina, Federation of Noncommunicable Disease Risk Factor Survey 2002.

Public Health Institute of Iceland. Iceland Dietary Survey 2010-2011.

Qatar Statistics Authority, Supreme Council of Health (Qatar), World Health Organization (WHO). Qatar STEPS Noncommunicable Disease Risk Factors Survey 2012.

Ragoobirsingh D, Morrison EYSA, Johnson P, Lewis-Fuller E. Obesity in the Caribbean: the Jamaican experience. Diabet Obes Metab. 2004; 6(1): 23-7.

Rajput N, Tuohy P, Mishra S, Smith A, Taylor B. Overweight and obesity in 4-5-year-old children in New Zealand: results from the first 4 years 2009-2012 of the B4School Check programme. J Paediatr Child Health. 2015; 51(3): 334–43.

Ramachandran A, Mary S, Yamuna A, Murugesan N, Snehalatha C. High prevalence of diabetes and cardiovascular risk factors associated with urbanization in India. Diabetes Care. 2008; 31(5): 893-8.

Ramachandran A, Snehalatha C, Vinitha R, Thayyil M, Kumar CKS, Sheeba L, Joseph S, Vijay V. Prevalence of overweight in urban Indian adolescent school children. Diabetes Res Clin Pract. 2002; 57(3): 185-90.

Ramke J, Brian G. BMI among Timorese aged ≥ 40 years. Public Health Nutr. 2012; 15(11): 2118-23.

Ramos de Marins VM, Varnier Almeida RM, Pereira RA, Barros MB. Factors associated with overweight and central body fat in the city of Rio de Janeiro: results of a two-stage random sampling survey. Public Health. 2001; 115(3): 236-42.

Ramos E, Barros H. Family and school determinants of overweight in 13-year-old Portuguese adolescents. Acta Paediatr. 2007; 96(2): 281-6.

RAND Corporation, University of Indonesia. Indonesia Family Life Survey 1993-1994. Santa Monica, United States: RAND Corporation.

Ranilović J, Markovina J, Znidar K, Colić Barić I. Attitudes to healthy eating among a representative sampling of Croatian adults: a comparison with Mediterranean countries. Int J Food Sci Nutr. 2009; 11-29.

Rapp K, Schroeder J, Klenk J, Stoehr S, Ulmer H, Concin H, Diem G, Oberaigner W, Weiland SK. Obesity and incidence of cancer: a large cohort study of over 145,000 adults in Austria. Br J Cancer. 2005; 93(9): 1062–7.

Rashidy-Pour A, Malek M, Eskandarian R, Ghorbani R. Obesity in the Iranian population. Obes Rev. 2009; 10(1): 2-6.

Rasmussen F, Eriksson M, Nordquist T. Bias in height and weight reported by Swedish adolescents and relations to body dissatisfaction: the COMPASS study. Eur J Clin Nutr. 2007; 61(7): 870-6.

Rasmussen M, Holstein BE, Due P. Tracking of overweight from mid-adolescence into adulthood: consistent patterns across socio-economic groups. Eur J Public Health. 2012; 22(6): 885-7.

Reddy KS, Prabhakaran D, Shah P, Shah B. Differences in body mass index and waist: hip ratios in North Indian rural and urban populations. Obes Rev. 2002; 3(3): 197-202.

Redon J, Cea-Calvo L, Lozano JV, Martı-Canales JC, Llisterri JL, Aznar J, Gonzalez-Esteban J, et al. Spain Prevention of Stroke Risk (PREV-ICTUS) 2005.

Redón J, Cea-Calvo L, Lozano JV, Martí-Canales JC, Llisterri JL, Aznar J, González-Esteban J, Investigators of the PREV-ICTUS study. Blood pressure and estimated risk of stroke in the elderly population of Spain: the PREV-ICTUS study. Stroke. 2007; 38(4): 1167-73.

Regidor E, Banegas JR, Gutiérrez-Fisac JL, Domínguez V, Rodríguez-Artalejo F. Influence of childhood socioeconomic circumstances, height, and obesity on pulse pressure and systolic and diastolic blood pressure in older people. J Hum Hypertens. 2006; 20(1): 73-82.

Reichert FF, Azevedo MR, Breier A, Gerage AM. Physical activity and prevalence of hypertension in a population-based sample of Brazilian adults and elderly. Prev Med. 2009; 49(2-3): 200-4.

Renzaho A, Wooden M, Houng B. Associations between body mass index and health-related quality of life among Australian adults. Qual Life Res. 2010; 19(4): 515-20.

Rerksuppaphol S, Rerksuppaphol L. Prevalence of overweight and obesity among school children in suburb Thailand defined by the International Obesity Task Force Standard. [corrected]. J Med Assoc Thai. 2010; S27-31.

Rex KF, Larsen NH, Rex H, Niclasen B, Pedersen ML. A national study on weight classes among children in Greenland at school entry. Int J Circumpolar Health. 2014; 73..

Reynolds K, Gu D, Whelton PK, Wu X, Duan X, Mo J, He J, InterASIA Collaborative Group. Prevalence and risk factors of overweight and obesity in China. Obesity (Silver Spring). 2007; 15(1): 10-8.

Ribeiro J, Santos P, Duarte J, Mota J. Association between overweight and early sexual maturation in Portuguese boys and girls. Ann Hum Biol. 2006; 33(1): 55-63.

Robert Koch Institute. Germany Health Interview and Examination Survey 2008-2011.

Robert Koch Institute. Germany Telephone Health Survey 2002-2003.

Roche (France). France National Survey of Overweight and Obesity 2003.

Rodenburg G, Kremers SPJ, Oenema A, van de Mheen D. Associations of parental feeding styles with child snacking behaviour and weight in the context of general parenting. Public Health Nutr. 2014; 17(5): 960–9.

Rodrigues Junior W, Gaban SCN, Pontes ERJC, Souza CC, Gimenes LP, Lacerda PF, Cunha MLMN, Stefanello II JVL, Brum LM, Oliveira LA, Silva CR, Ribeiro ALD. Diabetes mellitus and impaired glucose tolerance in urban adult population. Rev Assoc Med Bras. 2014; 60(2): 118-24.

Rodríguez-Artalejo F, Garcés C, Gorgojo L, López García E, Martín-Moreno JM, Benavente M, del Barrio JL, Rubio R, Ortega H, Fernández O, de Oya M, Investigators of the Four Provinces Study. Dietary patterns among children aged 6-7 y in four Spanish cities with widely differing cardiovascular mortality. Eur J Clin Nutr. 2002; 56(2): 141-8.

Rodríguez-Martín A, Novalbos Ruiz JP, Martínez Nieto JM, Escobar Jiménez L. Life-style factors associated with overweight and obesity among Spanish adults. Nutr Hosp. 2009; 24(2): 144-51.

Rolland-Cachera M-F, Castetbon K, Arnault N, Bellisle F, Romano M-C, Lehingue Y, Frelut M-L, Hercberg S. Body mass index in 7-9-y-old French children: frequency of obesity, overweight and thinness. Int J Obes Relat Metab Disord. 2002; 26(12): 1610-6.

Rosaneli CF, Baena CP, Auler F, Nakashima ATA, Netto-Oliveira ER, Oliveira AB, Guarita-Souza LC, Olandoski M, Faria-Neto JR. Elevated blood pressure and obesity in childhood: a cross-sectional evaluation of 4,609 schoolchildren. Arq Bras Cardiol. 2014; 103(3): 238–44.

Rosero-Bixby, Luis , Xinia Fernández, and William H. Dow. CRELES: Costa Rican Longevity and Healthy Aging Study, 2005 (Costa Rica Estudio de Longevidad y Envejecimiento Saludable) [Computer file]. ICPSR26681-v1. Ann Arbor, MI: Inter-university Consortium for Political and Social Research [distributor], 2010-07-21. doi:10.3886/ICPSR26681

Rosero-Bixby, Luis, Xinia Fernández, and William H. Dow. CRELES-2: Costa Rican Longevity and Healthy Aging Study - Wave 2, 2006-2008 (Costa Rica Estudio de Longevidad y Envejecimiento Saludable, Ronda 2). ICPSR31263-v1. Ann Arbor, MI: Inter-university Consortium for Political and Social Research [distributor], 2013-10-23. doi:10.3886/ICPSR31263.v1

Roskam A-JR, Kunst AE. The predictive value of different socio-economic indicators for overweight in nine European countries. Public Health Nutr. 2008; 11(12): 1256-66.

Rossiter MD, Colapinto CK, Khan MKA, McIsaac J-LD, Williams PL, Kirk SFL, Veugelers PJ. Breast, Formula and Combination Feeding in Relation to Childhood Obesity in Nova Scotia, Canada. Matern Child Health J. 2015; 19(9): 2048–56.

Rush E, Reed P, McLennan S, Coppinger T, Simmons D, Graham D. A school-based obesity control programme: Project Energize. Two-year outcomes. Br J Nutr. 2012; 107(4): 581-7.

Rush E, Reed PW, Simmons D, Coppinger T, McLennan S, Graham D. Baseline measures for a school-based obesity control programme: Project Energize: differences by ethnicity, rurality, age and school socio-economic status. J Paediatr Child Health. 2013; 49(4): e324-331.

Russell J, Flood V, Yeatman H, Mitchell P. Prevalence and risk factors of food insecurity among a cohort of older Australians. J Nutr Health Aging. 2014; 18(1): 3–8.

Russia Longitudinal Monitoring Survey (RLMS-HSE), Round IX 2000. National Research University Higher School of Economics, ZAO Demoscope, Carolina Population Center, Univeristy of North Carolina at Chapel Hill, Institute of Sociology, Russian Academy of Sciences.

Russia Longitudinal Monitoring Survey (RLMS-HSE), Round V 1994. National Research University Higher School of Economics, ZAO Demoscope, Carolina Population Center, Univeristy of North Carolina at Chapel Hill, Institute of Sociology, Russian Academy of Sciences.

Russia Longitudinal Monitoring Survey (RLMS-HSE), Round VI 1995. National Research University Higher School of Economics, ZAO Demoscope, Carolina Population Center, Univeristy of North Carolina at Chapel Hill, Institute of Sociology, Russian Academy of Sciences.

Russia Longitudinal Monitoring Survey (RLMS-HSE), Round VII 1996. National Research University Higher School of Economics, ZAO Demoscope, Carolina Population Center, Univeristy of North Carolina at Chapel Hill, Institute of Sociology, Russian Academy of Sciences.

Russia Longitudinal Monitoring Survey (RLMS-HSE), Round VIII 1998-1999. National Research University Higher School of Economics, ZAO Demoscope, Carolina Population Center, Univeristy of North Carolina at Chapel Hill, Institute of Sociology, Russian Academy of Sciences.

Russia Longitudinal Monitoring Survey (RLMS-HSE), Round X 2001. National Research University Higher School of Economics, ZAO Demoscope, Carolina Population Center, Univeristy of North Carolina at Chapel Hill, Institute of Sociology, Russian Academy of Sciences.

Russia Longitudinal Monitoring Survey (RLMS-HSE), Round XI 2002. National Research University Higher School of Economics, ZAO Demoscope, Carolina Population Center, Univeristy of North Carolina at Chapel Hill, Institute of Sociology, Russian Academy of Sciences.

Russia Longitudinal Monitoring Survey (RLMS-HSE), Round XII 2003. National Research University Higher School of Economics, ZAO Demoscope, Carolina Population Center, Univeristy of North Carolina at Chapel Hill, Institute of Sociology, Russian Academy of Sciences.

Russian Academy of Medical Science, World Health Organization (WHO). Russia WHO Study on Global AGEing and Adult Health 2007-2010.

Saaristo TE, Barengo NC, Korpi-Hyövälti E, Oksa H, Puolijoki H, Saltevo JT, Vanhala M, Sundvall J, Saarikoski L, Peltonen M, Tuomilehto J. High prevalence of obesity, central obesity and abnormal glucose tolerance in the middle-aged Finnish population. BMC Public Health. 2008; 8(1): 423.

Sachdev HPS, Osmond C, Fall CHD, Lakshmy R, Ramji S, Dey Biswas SK, Prabhakaran D, Tandon N, Reddy KS, Barker DJP, Bhargava SK. Predicting adult metabolic syndrome from childhood body mass index: follow-up of the New Delhi birth cohort. Arch Dis Child. 2009; 94(10): 768-74.

Saely CH, Risch L, Frey F, Lupi GA, Leuppi JD, Drexel H, Huber AR. Body mass index, blood pressure, and serum cholesterol in young Swiss men: an analysis on 56784 army conscripts. Swiss Med Wkly. 2009; 139(35-36): 518-24.

Sahel Institute, Westinghouse; Institute for Resource Development. Mali Demographic and Health Survey 1987. Columbia, United States: Westinghouse; Institute for Resource Development.

Saito I, Mori M, Shibata H, Hirose H, Tsujioka M, Kawabe H. Prevalence of metabolic syndrome in young men in Japan. J Atheroscler Thromb. 2007; 14(1): 27-30.

Sakai R. Relationship between prevalence of childhood obesity in 17-year-olds and socioeconomic and environmental factors: prefecture-level analysis in Japan. Asia Pac J Public Health. 2013; 25(2): 159-69.

Sakamoto N, Yang L. BMI centile curves for Japanese children aged 5-17 years in 2000-2005. Public Health Nutr. 2009; 12(10): 1688-92.

Salanave B, Peneau S, Rolland-Cachera M-F, Hercberg S, Castetbon K. Stabilization of overweight prevalence in French children between 2000 and 2007. Int J Pediatr Obes. 2009; 4(2): 66-72.

Salcedo V, Gutiérrez-Fisac JL, Guallar-Castillón P, Rodríguez-Artalejo F. Trends in overweight and misperceived overweight in Spain from 1987 to 2007. Int J Obes (Lond). 2010; 34(12): 1759-65.

Sánchez-Castillo CP, Lara JJ, Villa AR, Aguirre J, Escobar M, Gutiérrez H, Chávez A, James WP. Unusually high prevalence rates of obesity in four Mexican rural communities. Eur J Clin Nutr. 2001; 55(10): 833-40.

Sandercock GRH, Ogunleye A, Voss C. Comparison of cardiorespiratory fitness and body mass index between rural and urban youth: findings from the East of England Healthy Hearts Study. Pediatr Int. 2011; 53(5): 718-24.
Sanigorski AM, Bell AC, Kremer PJ, Swinburn BA. High childhood obesity in an Australian population. Obesity (Silver Spring). 2007; 15(8): 1908-12.
Santos R, Santos MP, Ribeiro JC, Mota J. Physical activity and other lifestyle behaviors in a Portuguese sample of adults: results from the Azorean Physical Activity and Health Study. J Phys Act Health. 2009; 6(6): 750-9.
Sardinha LB, Santos R, Vale S, Silva AM, Ferreira JP, Raimundo AM, Moreira H, Baptista F, Mota J. Prevalence of overweight and obesity among Portuguese youth: a study in a representative sample of 10-18-year-old children and adolescents. Int J Pediatr Obes. 2011; 6(2-2): e124-128.
Savva SC, Kourides Y, Tornaritis M, Epiphaniou-Savva M, Chadjigeorgiou C, Kafatos A. Obesity in children and adolescents in Cyprus. Prevalence and predisposing factors. Int J Obes Relat Metab Disord. 2002; 26(8): 1036-45.
Savva SC, Kourides YA, Hadjigeorgiou C, Tornaritis MJ. Overweight and obesity prevalence and trends in children and adolescents in Cyprus 2000-2010. Obes Res Clin Pract. 2014; 8(5): e426-434.
Savva SC, Tornaritis M, Chadjigeorgiou C, Kourides YA, Savva ME, Panagi A, Chrictodoulou E, Kafatos A. Prevalence and socio-demographic associations of undernutrition and obesity among preschool children in Cyprus. Eur J Clin Nutr. 2005; 59(11): 1259-65.
Schwarz B, Bischof HP, Kunze M. Overweight and coronary risk factors results from a western Austrian survey. Soz Praventivmed. 1991; 36(6): 322-6.
Scientific Institute of Public Health - Louis Pasteur (Belgium), Statistics Belgium. Belgium Health Interview Survey 1997.
Scientific Institute of Public Health (IPH) (Belgium), Statistics Belgium. Belgium Health Interview Survey 2001.
Scientific Institute of Public Health (IPH) (Belgium), Statistics Belgium. Belgium Health Interview Survey 2004.
Scientific Institute of Public Health (WIV-ISP) (Belgium), Statistics Belgium. Belgium Health Interview Survey 2008.
ScotCen Social Research and University College London. Department of Epidemiology and Public Health, Scottish Health Survey, 2010 [computer file]. Colchester, Essex: UK Data Archive [distributor], April 2012. SN: 6987, http://dx.doi.org/10.5255/UKDA-SN-6987-1
ScotCen Social Research, Scottish Health Survey, 2013 [computer file]. Colchester, Essex: UK Data Archive [distributor], December 2014. SN: 7594, http://dx.doi.org/10.5255/UKDA-SN-7594-1.
ScotCen Social Research, University College London. Department of Epidemiology and Public Health and University of Glasgow. MRC/CSO Social and Public Health Sciences Unit, Scottish Health Survey, 2011 [computer file]. 2nd Edition. Colchester, Essex: UK Data Archive [distributor], August 2013. SN: 7247, http://dx.doi.org/10.5255/UKDA-SN-7247-2
ScotCen Social Research, University College London. Department of Epidemiology and Public Health and University of Glasgow. MRC/CSO Social and Public Health Sciences Unit, Scottish Health Survey, 2012 [computer file]. 2nd Edition. Colchester, Essex: UK Data Archive [distributor], January 2014. SN: 7417, http://dx.doi.org/10.5255/UKDA-SN-7417-2
Scottish Centre for Social Research and University College London. Department of Epidemiology and Public Health, Scottish Health Survey, 2008 [computer file]. 2nd Edition. Colchester, Essex: UK Data Archive [distributor], April 2013. SN: 6383, http://dx.doi.org/10.5255/UKDA-SN-6383-2
Scottish Centre for Social Research and University College London. Department of Epidemiology and Public Health, Scottish Health Survey, 2009 [computer file]. 4th Edition. Colchester, Essex: UK Data Archive [distributor], November 2011. SN: 6713, http://dx.doi.org/10.5255/UKDA-SN-6713-2
Secretary State of Planning, National Institute of Statistics and Census (INEC), United Nations Children's Fund (UNICEF). Guinea-Bissau Multiple Indicator Cluster Survey 2000. New York, United States: United Nations Children's Fund (UNICEF).

Sekine M, Yamagami T, Hamanishi S, Handa K, Saito T, Nanri S, Kawaminami K, Tokui N, Yoshida K, Kagamimori S. Parental obesity, lifestyle factors and obesity in preschool children: results of the Toyama Birth Cohort study. J Epidemiol. 2002; 12(1): 33-9.

Senol V, Unalan D, Bayat M, Mazicioglu MM, Ozturk A, Kurtoglu S. Change in reference body mass index percentiles and deviation in overweight and obesity over 3 years in Turkish children and adolescents. J Pediatr Endocrinol Metab. 2014; 27(11–12): 1121–9.

Serra-Majem L, Aranceta Bartrina J, Pérez-Rodrigo C, Ribas-Barba L, Delgado-Rubio A. Prevalence and deteminants of obesity in Spanish children and young people. Br J Nutr. 2006; S67-72.

Serrano-Sanchez JA, Lera-Navarro A, Dorado-García C, González-Henriquez JJ, Sanchis-Moysi J. Contribution of individual and environmental factors to physical activity level among Spanish adults. PLoS One. 2012; 7(6): e38693.

Shafique S, Akhter N, Stallkamp G, de Pee S, Panagides D, Bloem MW. Trends of under- and overweight among rural and urban poor women indicate the double burden of malnutrition in Bangladesh. Int J Epidemiol. 2007; 36(2): 449-57.

Shah SM, Nanan D, Rahbar MH, Rahim M, Nowshad G. Assessing obesity and overweight in a high mountain Pakistani population. Trop Med Int Health. 2004; 9(4): 526-32.

Shan X-Y, Xi B, Cheng H, Hou D-Q, Wang Y, Mi J. Prevalence and behavioral risk factors of overweight and obesity among children aged 2-18 in Beijing, China. Int J Pediatr Obes. 2010; 5(5): 383–9.

Shapo L, Pomerleau J, McKee M, Coker R, Ylli A. Body weight patterns in a country in transition: a population-based survey in Tirana City, Albania. Public Health Nutr. 2003; 6(5): 471-7.

Shelley E, Daly L, Kilcoyne D, Graham I. Obesity: a public health problem in Ireland?. Ir J Med Sci. 1991; 29-34.

Shinozaki K, Okuda M, Sasaki S, Kunitsugu I, Shigeta M. Dietary Fiber Consumption Decreases the Risks of Overweight and Hypercholesterolemia in Japanese Children. Ann Nutr Metab. 2015; 67(1): 58–64.

Shirasawa T, Ochiai H, Nanri H, Nishimura R, Ohtsu T, Hoshino H, Tajima N, Kokaze A. Trends of Underweight and Overweight Obesity Among Japanese Schoolchildren From 2003 to 2012, Defined by Body Mass Index and Percentage Overweight Cutoffs. J Epidemiol. 2015; 25(7): 482–8.

Shiri R, Solovieva S, Husgafvel-Pursiainen K, Taimela S, Saarikoski LA, Huupponen R, Viikari J, Raitakari OT, Viikari-Juntura E. The association between obesity and the prevalence of low back pain in young adults: the Cardiovascular Risk in Young Finns Study. Am J Epidemiol. 2008; 167(9): 1110-9.

Shiue I. Associated social factors of body mass index in adults and the very old in the UK. Int J Cardiol. 2013; 168(1): 543-5.

Siervo M, Grey P, Nyan OA, Prentice AM. Urbanization and obesity in The Gambia: a country in the early stages of the demographic transition. Eur J Clin Nutr. 2006; 60(4): 455-63.

Simen-Kapeu A, Kuhle S, Veugelers PJ. Geographic differences in childhood overweight, physical activity, nutrition and neighbourhood facilities: implications for prevention. Can J Public Health. 2010; 101(2): 128-32.

Simsek E, Akpinar S, Bahcebasi T, Senses DA, Kocabay K. The prevalence of overweight and obese children aged 6-17 years in the West Black Sea region of Turkey. Int J Clin Pract. 2008; 62(7): 1033-8.

Singh RB, Bajaj S, Niaz MA, Rastogi SS, Moshiri M. Prevalence of type 2 diabetes mellitus and risk of hypertension and coronary artery disease in rural and urban population with low rates of obesity. Int J Cardiol. 1998; 66(1): 65-72.

Siu J, Giskes K, Shaw J, Turrell G. Perceived weight status may contribute to education inequalities in five-year weight change among mid-aged women. Aust N Z J Public Health. 2011; 35(3): 284-91.

Sjöberg A, Moraeus L, Yngve A, Poortvliet E, Al-Ansari U, Lissner L. Overweight and obesity in a representative sample of schoolchildren - exploring the urban-rural gradient in Sweden. Obes Rev. 2011; 12(5): 305-14.

Smetanina N, Albaviciute E, Babinska V, Karinauskiene L, Albertsson-Wikland K, Petrauskiene A, Verkauskiene R. Prevalence of overweight/obesity in relation to dietary habits and lifestyle among 7-17 years old children and adolescents in Lithuania. BMC Public Health. 2015; 15: 1001.

Smith BJ, Phongsavan P, Havea D, Halavatau V, Chey T. Body mass index, physical activity and dietary behaviours among adolescents in the Kingdom of Tonga. Public Health Nutr. 2007; 10(2): 137-44.
Smpokos EA, Linardakis M, Papadaki A, Kafatos A. Secular changes in anthropometric measurements and blood pressure in children of Crete, Greece, during 1992/93 and 2006/07. Prev Med. 2011; 52(3-4): 213-7.
Snoek HM, van Strien T, Janssens JMAM, Engels RCME. Emotional, external, restrained eating and overweight in Dutch adolescents. Scand J Psychol. 2007; 48(1): 23-32.
So H-K, Nelson EAS, Li AM, Wong EMC, Lau JTF, Guldan GS, Mak K-H, Wang Y, Fok T-F, Sung RYT. Secular changes in height, weight and body mass index in Hong Kong Children. BMC Public Health. 2008; 8: 320.
Sobngwi E, Mbanya JC, Unwin NC, Kengne AP, Fezeu L, Minkoulou EM, Aspray TJ, Alberti KG. Physical activity and its relationship with obesity, hypertension and diabetes in urban and rural Cameroon. Int J Obes Relat Metab Disord. 2002; 26(7): 1009-16.
Solbraa AK, Holme IM, Graff-Iversen S, Resaland GK, Aadland E, Anderssen SA. Physical activity and cardiovascular risk factors in a 40- to 42-year-old rural Norwegian population from 1975-2010: repeated cross-sectional surveys. BMC Public Health. 2014; 14: 569.
Soori H. Pattern of dietary behaviour and obesity in Ahwaz, Islamic Republic of Iran. East Mediterr Health J. 2001; 7(1-2): 163-70.
Southern Africa Labour and Development Research Unit. National Income Dynamics Study 2010-2011, Wave 2. Version 1.0. Cape Town: Southern Africa Labour and Development Research Unit [producer], 2012. Cape Town: DataFirst [distributor], 2013.
Southern Africa Labour and Development Research Unit. National Income Dynamics Study 2012, Wave 3 [dataset]. Version 1.2. Cape Town: Southern Africa Labour and Development Research Unit [producer], 2013. Cape Town: DataFirst [distributor], 2013
Southern Africa Labour Development Research Unit (SALDRU), University of Cape Town. South Africa Integrated Family Survey 1999.
Southern Sudan Centre for Census, Statistics and Evaluation (SSCCSE), Food and Agriculture Organization of the United Nations (FAO), Statistics Norway. South Sudan National Baseline Household Survey (NBHS) 2009. Juba, Sudan: Southern Sudan Centre for Census, Statistics and Evaluation (SSCCSE).
Spahija B, Qirjako G, Toçi E, Roshi E, Burazeri G. Socioeconomic and lifestyle determinants of obesity in a transitional southeast European population. Med Arh. 2012; 66(3 Suppl 1): 16-20.
Spanish Agency for Food Safety and Nutrition. Spain ALADINO Study 2010-2011.
Stamm H, Gebert A, Guggenbühl L, Lamprecht M. Excess weight among children and adolescents in Switzerland--prevalence and correlates for the early 2010s. Swiss Med Wkly. 2014; 144: w13956.
Starc G, Strel J. Tracking excess weight and obesity from childhood to young adulthood: a 12-year prospective cohort study in Slovenia. Public Health Nutr. 2011; 14(1): 49-55.
State Statistical Office (Macedonia) and United Nations Children's Fund (UNICEF). Macedonia Multiple Indicator Cluster Survey 2005. New York, United States: United Nations Children's Fund (UNICEF).
State Statistics Committee of Azerbaijan, United Nations Children's Fund (UNICEF). Azerbaijan Multiple Indicator Cluster Survey 2000. New York, United States: United Nations Children's Fund (UNICEF).
Statistical Institute of Belize, United Nations Children's Fund (UNICEF). Belize Multiple Indicator Cluster Survey 2006. New York, United States: United Nations Children's Fund (UNICEF).
Statistical Institute of Belize, United Nations Children's Fund (UNICEF). Belize Multiple Indicator Cluster Survey 2011. New York, United States: United Nations Children's Fund (UNICEF), 2013.
Statistical Office of Montenegro. Montenegro Multiple Indicator Cluster Survey 2013. New York, United States: United Nations Children's Fund (UNICEF), 2015.
Statistical Office of the Republic of Serbia, United Nations Children's Fund (UNICEF). Serbia Multiple Indicator Cluster Survey 2010. New York, United States: United Nations Children's Fund (UNICEF).

Statistical Office of the Republic of Serbia. Serbia Multiple Indicator Cluster Survey 2014. New York, United States: United Nations Children's Fund (UNICEF), 2015.
Statistical Service of Cyprus (CYSTAT). Cyprus European Health Interview Survey 2008.
Statistics Austria. Austria Health Survey 2006-2007.
Statistics Canada. Canada CANSIM Database - Body Mass Index, by Age Group and Sex, Household Population Aged 18 and Over Excluding Pregnant Women, Canada and Provinces, Every 2 Years. Ottawa, Canada: Statistics Canada.
Statistics Canada. Canada CANSIM Database - Health Indicator Profile, Annual Estimates, by Age Group and Sex, Canada, Provinces, Territories, Health Regions, and Peer Groups. Ottawa, Canada: Statistics Canada.
Statistics Canada. Canada Community Health Survey - Healthy Aging 2008-2009. Ottawa, Canada: Statistics Canada.
Statistics Canada. Canada Community Health Survey 2000-2001. Ottawa, Canada: Statistics Canada, 2003.
Statistics Canada. Canada Community Health Survey 2003. Ottawa, Canada: Statistics Canada.
Statistics Canada. Canada Community Health Survey 2009-2010. Ottawa, Canada: Statistics Canada, 2011.
Statistics Canada. Canada Community Health Survey 2010. Ottawa, Canada: Statistics Canada.
Statistics Canada. Canada National Population Health Survey 1998-1999. Ottawa, Canada: Statistics Canada.
Statistics Netherlands. Netherlands Permanent Quality of Life Survey 2000.
Statistics Netherlands. Netherlands Permanent Quality of Life Survey 2001.
Statistics Netherlands. Netherlands Permanent Quality of Life Survey 2002.
Statistics Netherlands. Netherlands Permanent Quality of Life Survey 2003.
Statistics Netherlands. Netherlands Permanent Quality of Life Survey 2004.
Statistics Netherlands. Netherlands Permanent Quality of Life Survey 2005.
Statistics Netherlands. Netherlands Permanent Quality of Life Survey 2006.
Statistics Netherlands. Netherlands Permanent Quality of Life Survey 2007.
Statistics Netherlands. Netherlands Permanent Quality of Life Survey 2008.
Statistics Netherlands. Netherlands Permanent Quality of Life Survey 2009.
Statistics Norway. Norway Lifestyle Habits by Gender and Age. Oslo, Norway: Statistics Norway.
Statistics Norway. Norway Survey of Living Conditions 2002. Oslo, Norway: Statistics Norway.
Statistics Norway. Norway Survey of Living Conditions 2005-2006. Oslo, Norway: Statistics Norway.
Statistics Norway. Norway Survey of Living Conditions 2008-2009. Oslo, Norway: Statistics Norway.
Statistics Sierra Leone, United Nations Children's Fund (UNICEF). Sierra Leone Multiple Indicator Cluster Survey 2010. New York, United States: United Nations Children's Fund (UNICEF).
Statistics Sweden, Swedish National Institute of Public Health. Sweden National Survey of Public Health 2004.
Statistics Sweden, Swedish National Institute of Public Health. Sweden National Survey of Public Health 2005.
Statistics Sweden, Swedish National Institute of Public Health. Sweden National Survey of Public Health 2006.
Statistics Sweden, Swedish National Institute of Public Health. Sweden National Survey of Public Health 2007.
Statistics Sweden, Swedish National Institute of Public Health. Sweden National Survey of Public Health 2008.
Statistics Sweden, Swedish National Institute of Public Health. Sweden National Survey of Public Health 2009.
Statistics Sweden, Swedish National Institute of Public Health. Sweden National Survey of Public Health 2010.
Statistics Sweden, Swedish National Institute of Public Health. Sweden National Survey of Public Health 2011.
Statistics Sweden, Swedish National Institute of Public Health. Sweden National Survey of Public Health 2012.
Steele RM, van Sluijs EMF, Cassidy A, Griffin SJ, Ekelund U. Targeting sedentary time or moderate- and vigorous-intensity activity: independent relations with adiposity in a population-based sample of 10-y-old British children. Am J Clin Nutr. 2009; 90(5): 1185-92.
Steenhuis IHM, Bos AER, Mayer B. (Mis)interpretation of body weight in adult women and men. J Hum Nutr Diet. 2006; 19(3): 219-28.

Stene LC, Giacaman R, Abdul-Rahim H, Husseini A, Norum KR, Holmboe-Ottesen G. Obesity and associated factors in a Palestinian West Bank village population. Eur J Clin Nutr. 2001; 55(9): 805-11.

Steyn K, Bourne L, Jooste P, Fourie JM, Rossouw K, Lombard C. Anthropometric profile of a black population of the Cape Peninsula in South Africa. East Afr Med J. 1998; 75(1): 35-40.

Stoupa A, Goischke A, Garcin C, Elie C, Viaud M, Thery A, Richard G, Polak M. Can school health check-ups serve as screening tool for growth anomalies and obesity in children?. J Pediatr Endocrinol Metab. 2015; 28(9–10): 1115–21.

Su TT, Sim PY, Nahar AM, Majid HA, Murray LJ, Cantwell MM, Al-Sadat N, Jalaludin MY. Association between self-reported physical activity and indicators of body composition in Malaysian adolescents. Prev Med. 2014; 67: 100–5.

Subramaniam M, Picco L, He V, Vaingankar JA, Abdin E, Verma S, Rekhi G, Yap M, Lee J, Chong SA. Body mass index and risk of mental disorders in the general population: results from the Singapore Mental Health Study. J Psychosom Res. 2013; 74(2): 135-41.

Subramanian SV, Smith GD. Patterns, distribution, and determinants of under- and overnutrition: a population-based study of women in India. Am J Clin Nutr. 2006; 84(3): 633-40.

Sumarni Mohd G, Muhammad Amir K, Ibrahim Md S, Mohd Rodi I, Izzuna Mudla MG, Nurziyana I. Obesity among schoolchildren in Kuala Selangor: a cross-sectional study. Trop Biomed. 2006; 23(2): 148-54.

Sun Y, Sekine M, Kagamimori S. Lifestyle and overweight among Japanese adolescents: the Toyama Birth Cohort Study. J Epidemiol. 2009; 19(6): 303-10.

Sundblom E, Petzold M, Rasmussen F, Callmer E, Lissner L. Childhood overweight and obesity prevalences levelling off in Stockholm but socioeconomic differences persist. Int J Obes (Lond). 2008; 32(10): 1525-30.

Sundquist J, Johansson S-E, Sundquist K. Levelling off of prevalence of obesity in the adult population of Sweden between 2000/01 and 2004/05. BMC Public Health. 2010; 119.

Suraj Eye Institute. India - Mahārāshtra Central India Eye and Medical Study 2006-2008.

Sutton-Tyrell, Kim, Faith Selzer, MaryFran Sowers, Joel Finkelstein, Lynda Powell, Ellen Gold, Gail Greendale, Gerson Weiss, and Karen Matthews. Study of Women's Health Across the Nation (SWAN), 2002-2004: Visit 06 Dataset. ICPSR31181-v1. Ann Arbor, MI: Inter-university Consortium for Political and Social Research [distributor], 2014-09-24. http://doi.org/10.3886/ICPSR31181.v1

Sutton-Tyrell, Kim, Faith Selzer, MaryFran Sowers, Joel Finkelstein, Lynda Powell, Ellen Gold, Gail Greendale, Gerson Weiss, and Karen Matthews. Study of Women's Health Across the Nation (SWAN), 2003-2005: Visit 07 Dataset. ICPSR31901-v1. Ann Arbor, MI: Inter-university Consortium for Political and Social Research [distributor], 2014-09-30. http://doi.org/10.3886/ICPSR31901.v1

Sutton-Tyrell, Kim, Faith Selzer, MaryFran Sowers, Joel Finkelstein, Lynda Powell, Ellen Gold, Gail Greendale, Gerson Weiss, and Karen Matthews. Study of Women's Health Across the Nation (SWAN), 2004-2006: Visit 08 Dataset. ICPSR32122-v1. Ann Arbor, MI: Inter-university Consortium for Political and Social Research [distributor], 2014-09-30. http://doi.org/10.3886/ICPSR32122.v1

Sutton-Tyrell, Kim, Faith Selzer, MaryFran Sowers, Joel Finkelstein, Lynda Powell, Ellen Gold, Gail Greendale, Gerson Weiss, and Karen Matthews. Study of Women's Health Across the Nation (SWAN), 2005-2007: Visit 09 Dataset. ICPSR32721-v1. Ann Arbor, MI: Inter-university Consortium for Political and Social Research [distributor], 2014-09-30. http://doi.org/10.3886/ICPSR32721.v1

Sutton-Tyrell, Kim, Faith Selzer, MaryFran Sowers, Robert Neer, Lynda Powell, Ellen Gold, Gail Greendale, Gerson Weiss, Karen Matthews, and Sonja McKinlay. Study of Women's Health Across the Nation (SWAN), 2000-2002: Visit 04 Dataset. ICPSR30142-v1. Ann Arbor, MI: Inter-university Consortium for Political and Social Research[distributor], 2014-02-13. http://doi.org/10.3886/ICPSR30142.v1

Sutton-Tyrell, Kim, Faith Selzer, MaryFran Sowers, Robert Neer, Lynda Powell, Ellen Gold, Gail Greendale, Gerson Weiss, Karen Matthews, and Sonja McKinlay. Study of Women's Health Across the Nation (SWAN), 2001-2003: Visit 05 Dataset. ICPSR30501-v1. Ann Arbor, MI: Inter-university Consortium for Political and Social Research [distributor], 2014-09-02. http://doi.org/10.3886/ICPSR30501.v1

Sutton-Tyrrell, Kim, Faith Selzer, MaryFran Sowers, Joel Finkelstein, Lynda Powell, Ellen Gold, Gail David, Gerson Weiss, and Karen Matthews. Study of Women Across the Nation (SWAN), 2006-2008: Visit 10 Dataset. ICPSR32961-v1. Ann Arbor, MI: Inter-university Consortium for Political and Social Research [distributor], 2014-10-08. http://doi.org/10.3886/ICPSR32961.v1

Sutton-Tyrrell, Kim, Faith Selzer, MaryFran Sowers, Robert Neer, Lynda Powell, Ellen Gold, Gail Greendale, Gerson Weiss, Karen Matthews, and Sonja McKinlay. Study of Women's Health Across the Nation (SWAN), 1996-1997: Baseline Dataset. ICPSR28762-v2. Ann Arbor, MI: Inter-university Consortium for Political and Social Research[distributor], 2014-02-04. http://doi.org/10.3886/ICPSR28762.v2

Sutton-Tyrrell, Kim, Faith Selzer, MaryFran Sowers, Robert Neer, Lynda Powell, Ellen Gold, Gail Greendale, Gerson Weiss, Karen Matthews, and Sonja McKinlay. Study of Women's Health Across the Nation (SWAN), 1997-1999: Visit 01 Dataset. ICPSR29221-v1. Ann Arbor, MI: Inter-university Consortium for Political and Social Research[distributor], 2014-02-05. http://doi.org/10.3886/ICPSR29221.v1

Sutton-Tyrrell, Kim, Faith Selzer, MaryFran Sowers, Robert Neer, Lynda Powell, Ellen Gold, Gail Greendale, Gerson Weiss, Karen Matthews, and Sonja McKinlay. Study of Women's Health Across the Nation (SWAN), 1998-2000: Visit 02 Dataset. ICPSR29401-v1. Ann Arbor, MI: Inter-university Consortium for Political and Social Research[distributor], 2014-02-06. http://doi.org/10.3886/ICPSR29401.v1

Sutton-Tyrrell, Kim, Faith Selzer, MaryFran Sowers, Robert Neer, Lynda Powell, Ellen Gold, Gail Greendale, Gerson Weiss, Karen Matthews, and Sonja McKinlay. Study of Women's Health Across the Nation (SWAN), 1999-2001: Visit 03 Dataset. ICPSR29701-v1. Ann Arbor, MI: Inter-university Consortium for Political and Social Research [distributor], 2014-02-12. http://doi.org/10.3886/ICPSR29701.v1

Tai A, Volkmer R, Burton A. Association between asthma symptoms and obesity in preschool (4-5 year old) children. J Asthma. 2009; 46(4): 362-5.

Tai TY, Chuang LM, Wu HP, Chen CJ. Association of body build with non-insulin-dependent diabetes mellitus and hypertension among Chinese adults: a 4-year follow-up study. Int J Epidemiol. 1992; 21(3): 511-7.

Takahashi Y, Sakai M, Tokuda Y, Takahashi O, Ohde S, Nakayama T, Fukuhara S, Fukui T, Shimbo T. The relation between self-reported body weight and health-related quality of life: a cross-sectional study in Japan. J Public Health (Oxf). 2011; 33(4): 518-26.

Tanaka K, Kiyohara Y, Kubo M, Matsumoto T, Tanizaki Y, Okubo K, Ninomiya T, Oishi Y, Shikata K, Iida M. Secular trends in the incidence, mortality, and survival rate of gastric cancer in a general Japanese population: the Hisayama study. Cancer Causes Control. 2005; 16(5): 573-8.

Tanchoco CC, Cruz AJ, Duante CA, Litonjua AD. Prevalence of metabolic syndrome among Filipino adults aged 20 years and over. Asia Pac J Clin Nutr. 2003; 12(3): 271-6.

Tang HK, Dibley MJ, Sibbritt D, Tran HM. Gender and socio-economic differences in BMI of secondary high school students in Ho Chi Minh city. Asia Pac J Clin Nutr. 2007; 16(1): 74-83.

Tangtrakulwanich B, Suwanno P. Epidemiology and risk factors of patellofemoral osteoarthritis in adults: a population-based study in southern Thailand. J Med Assoc Thai. 2012; 95(8): 1048-52.

Taylor R, Jalaludin B, Levy S, Montaville B, Gee K, Sladden T. Prevalence of diabetes, hypertension and obesity at different levels of urbanisation in Vanuatu. Med J Aust. 1991; 155(2): 86-90.

Tazi MA, Abir-Khalil S, Lahmouz F, Arrach ML, Chaouki N. Risk factors for hypertension among the adult Moroccan population. East Mediterr Health J. 2009; 15(4): 827-41.

Tchicaya A, Lorentz N. Socioeconomic inequality and obesity prevalence trends in Luxembourg, 1995-2007. BMC Res Notes. 2012; 467.

Telepanel Foundation (STP), CentERdata. Netherlands Dutch National Bank Household Survey 1993-1994. Tillburg, Netherlands: CentERdata.

Temple NJ, Steyn K, Hoffman M, Levitt NS, Lombard CJ. The epidemic of obesity in South Africa: a study in a disadvantaged community. Ethn Dis. 2001; 11(3): 431-7.

The InterASIA Collaborative Group. Cardiovascular risk factor levels in urban and rural Thailand - The International Collaborative Study of Cardiovascular Disease in Asia (InterASIA). Eur J Cardiovasc Prev Rehabil. 2003; 10(4): 249-57.

Theorell-Haglöw J, Berglund L, Janson C, Lindberg E. Sleep duration and central obesity in women - differences between short sleepers and long sleepers. Sleep Med. 2012; 13(8): 1079-85.

Thibault H, Carriere C, Langevin C, Kossi Déti E, Barberger-Gateau P, Maurice S. Prevalence and factors associated with overweight and obesity in French primary-school children. Public Health Nutr. 2013; 16(2): 193-201.

Thibault H, Contrand B, Saubusse E, Baine M, Maurice-Tison S. Risk factors for overweight and obesity in French adolescents: physical activity, sedentary behavior and parental characteristics. Nutrition. 2010; 26(2): 192-200.

Thuesen BH, Cerqueira C, Aadahl M, Ebstrup JF, Toft U, Thyssen JP, Fenger RV, Hersoug L-G, Elberling J, Pedersen O, Hansen T, Johansen JD, Jørgensen T, Linneberg A. Cohort Profile: the Health2006 cohort, research centre for prevention and health. Int J Epidemiol. 2014; 43(2): 568–75.

Tian H, Xie H, Song G, Zhang H, Hu G. Prevalence of overweight and obesity among 2.6 million rural Chinese adults. Prev Med. 2009; 48(1): 59-63.

Tian X, Zhao G, Li Y, Wang L, Shi Y. Overweight and obesity difference of Chinese population between different urbanization levels. J Rural Health. 2014; 30(1): 101–12.

Tikkinen KAO, Auvinen A, Johnson TM 2nd, Weiss JP, Keränen T, Tiitinen A, Polo O, Partinen M, Tammela TLJ. A systematic evaluation of factors associated with nocturia--the population-based FINNO study. Am J Epidemiol. 2009; 170(3): 361-8.

Tjepkema, Michael, Statistics Canada. Canada Community Health Survey Findings – Adult Obesity in Canada: Measured Height and Weight 2004. Ottawa, Canada: Statistics Canada.

Toft U, Vinding AL, Larsen FB, Hvidberg MF, Robinson KM, Glumer C. The development in body mass index, overweight and obesity in three regions in Denmark. Eur J Public Health. 2015; 25(2): 273–8.

Tokmakidis SP, Christodoulos AD, Mantzouranis NI. Validity of self-reported anthropometric values used to assess body mass index and estimate obesity in Greek school children. J Adolesc Health. 2007; 40(4): 305-10.

Tokmakidis SP, Kasambalis A, Christodoulos AD. Fitness levels of Greek primary schoolchildren in relationship to overweight and obesity. Eur J Pediatr. 2006; 165(12): 867-74.

Torheim LE, Granli GI, Sidibé CS, Traoré AK, Oshaug A. Women's iodine status and its determinants in an iodine-deficient area in the Kayes region, Mali. Public Health Nutr. 2005; 8(4): 387-94.

Torheim LE, Ouattara F, Diarra MM, Thiam FD, Barikmo I, Hatløy A, Oshaug A. Nutrient adequacy and dietary diversity in rural Mali: association and determinants. Eur J Clin Nutr. 2004; 58(4): 594-604.

Toselli S, Brasili P, Iuliano T, Spiga F. Anthropometric variables, lifestyle and sports in school-age children: comparison between the cities of Bologna and Crotone. Homo. 2014; 65(6): 499–508.

Tringler M, Rodriguez EM, Aguera D, Molina JD, Canziani GA, Diaz A. High blood pressure, overweight and obesity among rural scholars from the Vela Project: a population-based study from South America. High Blood Press Cardiovasc Prev. 2012; 19(1): 41–6.

Trinity College Dublin. Ireland Longitudinal Study on Ageing 2009-2011. Dublin, Ireland: Irish Social Science Data Archive, University College Dublin.

Tuan NT, Tuong PD, Popkin BM. Body mass index (BMI) dynamics in Vietnam. Eur J Clin Nutr. 2008; 62(1): 78-86.

Turconi G, Maccarini L, Bazzano R, Roggi C. Overweight and blood pressure: results from the examination of a selected group of adolescents in northern Italy. Public Health Nutr. 2008; 11(9): 905-13.

Turconi G, Rossi M, Roggi C, Maccarini L. Nutritional status, dietary habits, nutritional knowledge and self-care assessment in a group of older adults attending community centres in Pavia, Northern Italy. J Hum Nutr Diet. 2013; 26(1): 48-55.

Turkish Society of Hypertension and Renal Diseases. Turkey Hypertension Prevalence Study 2012.

Turkish Society of Nephrology. Turkey Chronic Renal Disease In Turkey Study 2007.

Tutkuviene J. Body mass index, prevalence of overweight and obesity in Lithuanian children and adolescents, 1985-2002. Coll Antropol. 2007; 31(1): 109-21.

Tzotzas T, Kapantais E, Tziomalos K, Ioannidis I, Mortoglou A, Bakatselos S, Kaklamanou M, Lanaras L, Kaklamanos I. Epidemiological survey for the prevalence of overweight and abdominal obesity in Greek adolescents. Obesity (Silver Spring). 2008; 16(7): 1718-22.

Ul-Haq Z, Mackay DF, Fenwick E, Pell JP. Impact of metabolic comorbidity on the association between body mass index and health-related quality of life: a Scotland-wide cross-sectional study of 5,608 participants. BMC Public Health. 2012; 143.

United Arab Emirates University (UAEU). United Arab Emirates Health and Lifestyle Survey 2000.

United Nations Children's Fund (UNICEF), Bureau of Statistics (Guyana). Guyana Multiple Indicator Cluster Survey 2006. New York, United States: United Nations Children's Fund (UNICEF).

United Nations Children's Fund (UNICEF), Census Bureau (Chad), National Institute of Statistical, Economic and Demographic Studies (Chad). Chad Multiple Indicator Cluster Survey 2000. New York, United States: United Nations Children's Fund (UNICEF).

United Nations Children's Fund (UNICEF), Central Bureau of Statistics (Syria), Ministry of Health (Syria), Pan Arab Project for Family Health (PAPFAM). Syria Multiple Indicator Cluster Survey 2006. New York, United States: United Nations Children's Fund (UNICEF).

United Nations Children's Fund (UNICEF), Central Organization for Statistics and Information Technology (Iraq), Kurdistan Regional Statistics Office. Iraq Multiple Indicator Cluster Survey 2006. New York, United States: United Nations Children's Fund (UNICEF).

United Nations Children's Fund (UNICEF), Department of Statistics (Laos), Ministry of Health (Laos). Laos Multiple Indicator Cluster Survey 2006. New York, United States: United Nations Children's Fund (UNICEF).

United Nations Children's Fund (UNICEF), Government of Guinea-Bissau. Guinea-Bissau Multiple Indicator Cluster Survey 2006. New York, United States: United Nations Children's Fund (UNICEF).

United Nations Children's Fund (UNICEF), National Bureau of Statistics (Nigeria). Nigeria Multiple Indicator Cluster Survey 2007. New York, United States: United Nations Children's Fund (UNICEF).

United Nations Children's Fund (UNICEF), National Institute of Statistics (Cameroon). Cameroon Multiple Indicator Cluster Survey 2006. New York, United States: United Nations Children's Fund (UNICEF).

United Nations Children's Fund (UNICEF), National Institute of Statistics (Côte d'Ivoire). Côte d'Ivoire Multiple Indicator Cluster Survey 2006. New York, United States: United Nations Children's Fund (UNICEF).

United Nations Children's Fund (UNICEF), National Institute of Statistics and Demography (INSD) (Burkina Faso). Burkina Faso Multiple Indicator Cluster Survey 2006. New York, United States: United Nations Children's Fund (UNICEF).

United Nations Children's Fund (UNICEF), National Statistical Committee of the Kyrgyz Republic. Kyrgyzstan Multiple Indicator Cluster Survey 2005-2006. New York, United States: United Nations Children's Fund (UNICEF).

United Nations Children's Fund (UNICEF), National Statistics Institute (Mozambique). Mozambique Multiple Indicator Cluster Survey 2008-2009. New York, United States: United Nations Children's Fund (UNICEF).

United Nations Children's Fund (UNICEF), National Statistics Office (Malawi). Malawi Multiple Indicator Cluster Survey 2006. New York, United States: United Nations Children's Fund (UNICEF).

United Nations Children's Fund (UNICEF), State Committee of the Republic of Uzbekistan on Statistics. Uzbekistan Multiple Indicator Cluster Survey 2006. New York, United States: United Nations Children's Fund (UNICEF).

United Nations Children's Fund (UNICEF), State Committee on Statistics of the Republic of Tajikistan. Tajikistan Multiple Indicator Cluster Survey 2005. New York, United States: United Nations Children's Fund (UNICEF).

United Nations Children's Fund (UNICEF), Statistical Office of Montenegro. Montenegro Multiple Indicator Cluster Survey 2005. New York, United States: United Nations Children's Fund (UNICEF).

United Nations Children's Fund (UNICEF), Statistical Office of the Republic of Serbia (SORS), Strategic Marketing Research Agency (SMMRI). Serbia Multiple Indicator Cluster Survey 2005-2006. New York, United States: United Nations Children's Fund (UNICEF).

United Nations Children's Fund (UNICEF), Statistics Sierra Leone. Sierra Leone Multiple Indicator Cluster Survey 2005. New York, United States: United Nations Children's Fund (UNICEF).

United Nations Children's Fund (UNICEF), Zimbabwe National Statistics Agency. Zimbabwe Multiple Indicator Cluster Survey 2014. New York, United States: United Nations Children's Fund (UNICEF), 2015.

United Nations Children's Fund (UNICEF). Central African Republic Multiple Indicator Cluster Survey 2006. New York, United States: United Nations Children's Fund (UNICEF).

United Nations Children's Fund (UNICEF). South Sudan Multiple Indicator Cluster Survey 1999. New York, United States: United Nations Children's Fund (UNICEF).

United Nations Children's Fund (UNICEF). Zaire Multiple Indicator Cluster Survey 1995. New York, United States: United Nations Children's Fund (UNICEF).

United Nations Development Programme (UNDP), United Nations Children's Fund (UNICEF). Comoros Multiple Indicator Cluster Survey 2000. New York, United States: United Nations Children's Fund (UNICEF).

United States Department of Health and Human Services. Substance Abuse and Mental Health Services Administration. Office of Applied Studies. National Household Survey on Drug Abuse, 1999. ICPSR03239-v4. Ann Arbor, MI: Inter-university Consortium for Political and Social Research [distributor], 2006-12-07. doi:10.3886/ICPSR03239.v4

University of Cape Town, Southern Africa Labour and Development Research Unit. National Income Dynamics Study (NIDS) Wave 1 [computer files]. Cape Town: Southern Africa Labour and Development Research Unit [producer], 2009. Cape Town: DataFirst [distributor], 2009

University of Kwazulu-Natal, University of Wisconsin, London School of Hygiene and Tropical Medicine, International Food Policy Research Institute (IFPRI), Department of Social Development (South Africa), Norwegian Institute for Urban and Regional Research (NIBR). South Africa KwaZulu-Natal Income Dynamics Study 2004. Durban, South Africa: University of Kwazulu-Natal.

University of the West Indies. Jamaica Health and Lifestyle Survey 2007-2008.

Utter J, Denny S, Crengle S, Ameratunga S, Robinson E, Clark T, Percival T, Maddison R. Overweight among New Zealand adolescents: associations with ethnicity and deprivation. Int J Pediatr Obes. 2010; 5(6): 461-6.

Utter J, Scragg R, Schaaf D, Fitzgerald E, Wilson N. Correlates of body mass index among a nationally representative sample of New Zealand children. Int J Pediatr Obes. 2007; 2(2): 104-13.

Van Den Ende C, Twisk JWR, Monyeki KD. The relationship between BMI and dietary intake of primary school children from a rural area of South Africa: The Ellisras longitudinal study. Am J Hum Biol. 2014; 26(5): 701–6.

Van der Sluis ME, Lien N, Twisk JWR, Steenhuis IHM, Bere E, Klepp K-I, Wind M. Longitudinal associations of energy balance-related behaviours and cross-sectional associations of clusters and body mass index in Norwegian adolescents. Public Health Nutr. 2010; 13(10A): 1716-21.

van Nassau F, Singh AS, Cerin E, Salmon J, van Mechelen W, Brug J, Chinapaw MJ. The Dutch Obesity Intervention in Teenagers DOiT cluster controlled implementation trial: intervention effects and mediators and moderators of adiposity and energy balance-related behaviours. Int J Behav Nutr Phys Act. 2014; 11: 158.

van Nassau F, Singh AS, van Mechelen W, Brug J, Chinapaw MJM. Body mass index, waist circumference and skinfold thickness in 12- to 14-year-old Dutch adolescents: differences between 2003 and 2011. Pediatr Obes. 2014; 9(6): e137-140.

van Strien T, Herman CP, Verheijden MW. Dietary restraint and body mass change. A 3-year follow up study in a representative Dutch sample. Appetite. 2014; 76: 44–9.

Van Strien T, Herman CP, Verheijden MW. Eating style, overeating and weight gain. A prospective 2-year follow-up study in a representative Dutch sample. Appetite. 2012; 59(3): 782-9.

Van Zutphen M, Bell AC, Kremer PJ, Swinburn BA. Association between the family environment and television viewing in Australian children. J Paediatr Child Health. 2007; 43(6): 458-63.

Vanaelst B, Michels N, Clays E, Herrmann D, Huybrechts I, Sioen I, Vyncke K, De Henauw S. The association between childhood stress and body composition, and the role of stress-related lifestyle factors--cross-sectional findings from the baseline ChiBSD survey. Int J Behav Med. 2014; 21(2): 292–301.

Vaska V, Volkmer R. Increasing prevalence of obesity in South Australian 4-year-olds: 1995-2002. J Paediatr Child Health. 2004; 40(7): 353-5.

Vázquez FL, Díaz O, Pomar C. Prevalence of overweight and obesity among preadolescent schoolchildren in Galicia, Spain. Child Care Health Dev. 2010; 36(3): 392-5.

Vedamurthy I, Nahum M, Bavelier D, Levi DM. Mechanisms of recovery of visual function in adult amblyopia through a tailored action video game. Sci Rep. 2015; 5: 8482.

Veltsista A, Laitinen J, Sovio U, Roma E, Järvelin M-R, Bakoula C. Relationship between eating behavior, breakfast consumption, and obesity among Finnish and Greek adolescents. J Nutr Educ Behav. 2010; 42(6): 417-21.

Veugelers P, Sithole F, Zhang S, Muhajarine N. Neighborhood characteristics in relation to diet, physical activity and overweight of Canadian children. Int J Pediatr Obes. 2008; 3(3): 152-9.

Vidal E, Carlin E, Driul D, Tomat M, Tenore A. A comparison study of the prevalence of overweight and obese Italian preschool children using different reference standards. Eur J Pediatr. 2006; 165(10): 696-700.

Visscher TLS, Viet AL, Kroesbergen IHT, Seidell JC. Underreporting of BMI in adults and its effect on obesity prevalence estimations in the period 1998 to 2001. Obesity (Silver Spring). 2006; 14(11): 2054-63.

Wake M, Hardy P, Canterford L, Sawyer M, Carlin JB. Overweight, obesity and girth of Australian preschoolers: prevalence and socio-economic correlates. Int J Obes (Lond). 2007; 31(7): 1044-51.

Wake M, Hardy P, Sawyer MG, Carlin JB. Comorbidities of overweight/obesity in Australian preschoolers: a cross-sectional population study. Arch Dis Child. 2008; 93(6): 502-7.

Wallmann-Sperlich B, Froboese I. Physical activity during work, transport and leisure in Germany--prevalence and socio-demographic correlates. PLoS One. 2014; 9(11): e112333.

Walls HL, Peeters A, Son PT, Quang NN, Hoai NTT, Loi DD, Viet NL, Khai PG, Reid CM. Prevalence of underweight, overweight and obesity in urban Hanoi, Vietnam. Asia Pac J Clin Nutr. 2009; 18(2): 234-9.

Wang L, Hui SS. Diagnostic accuracy of different body weight and height-based definitions of childhood obesity in identifying overfat among Chinese children and adolescents: a cross-sectional study. BMC Public Health. 2015; 15: 802.

Wardle J, Williamson S, Johnson F, Edwards C. Depression in adolescent obesity: cultural moderators of the association between obesity and depressive symptoms. Int J Obes (Lond). 2006; 30(4): 634-43.

Warsy AS, el-Hazmi MA. Diabetes mellitus, hypertension and obesity--common multifactorial disorders in Saudis. East Mediterr Health J. 1999; 5(6): 1236-42.

Watkins DC, Murray LJ, McCarron P, Boreham CAG, Cran GW, Young IS, McGartland C, Robson PJ, Savage JM. Ten-year trends for fatness in Northern Irish adolescents: the Young Hearts Projects--repeat cross-sectional study. Int J Obes (Lond). 2005; 29(6): 579-85.

Wen JG, Li JS, Wang ZM, Huang CX, Shang XP, Su ZQ, Lu YT, Suo ZH, Wang Y, Qin GJ, Zhang WX, Heesakkers JP. The prevalence and risk factors of OAB in middle-aged and old people in China. Neurourol Urodyn. 2014; 33(4): 387–91.

Werner B, Bodin L. Obesity in Swedish schoolchildren is increasing in both prevalence and severity. J Adolesc Health. 2007; 41(6): 536-43.

Whelton H, Harrington J, Crowley E, Kelleher V, Cronin M, Perry IJ. Prevalence of overweight and obesity on the island of Ireland: results from the North South Survey of Children's Height, Weight and Body Mass Index, 2002. BMC Public Health. 2007; 187.

Wickens K, Barry D, Friezema A, Rhodius R, Bone N, Purdie G, Crane J. Obesity and asthma in 11-12 year old New Zealand children in 1989 and 2000. Thorax. 2005; 60(1): 7-12.

Wickramasinghe VP, Arambepola C, Bandara P, Abeysekera M, Kuruppu S, Dilshan P, Dissanayake BS. Distribution of obesity-related metabolic markers among 5-15 year old children from an urban area of Sri Lanka. Ann Hum Biol. 2013; 40(2): 168-74.

Wickramasinghe VP, Lamabadusuriya SP, Atapattu N, Sathyadas G, Kuruparanantha S, Karunarathne P. Nutritional status of schoolchildren in an urban area of Sri Lanka. Ceylon Med J. 2004; 49(4): 114-8.

Wijewardene K, Mohideen MR, Mendis S, Fernando DS, Kulathilaka T, Weerasekara D, Uluwitta P. Prevalence of hypertension, diabetes and obesity: baseline findings of a population based survey in four provinces in Sri Lanka. Ceylon Med J. 2005; 50(2): 62-70.

Wijnhoven TMA, van Raaij JMA, Spinelli A, Rito AI, Hovengen R, Kunesova M, Starc G, Rutter H, Sjöberg A, Petrauskiene A, O'Dwyer U, Petrova S, Farrugia Sant'angelo V, Wauters M, Yngve A, Rubana I-M, Breda J. WHO European Childhood Obesity Surveillance Initiative 2008: weight, height and body mass index in 6-9-year-old children. Pediatr Obes. 2013; 8(2): 79-97.

Wijnhoven TMA, van Raaij JMA, Spinelli A, Starc G, Hassapidou M, Spiroski I, Rutter H, Martos É, Rito AI, Hovengen R, Pérez-Farinós N, Petrauskiene A, Eldin N, Braeckevelt L, Pudule I, Kunešová M, Breda J. WHO European Childhood Obesity Surveillance Initiative: body mass index and level of overweight among 6-9-year-old children from school year 2007/2008 to school year 2009/2010. BMC Public Health. 2014; 14: 806.

Wildman RP, Gu D, Muntner P, Wu X, Reynolds K, Duan X, Chen C-S, Huang G, Bazzano LA, He J. Trends in overweight and obesity in Chinese adults: between 1991 and 1999-2000. Obesity (Silver Spring). 2008; 16(6): 1448-53.

Wildman RP, Gu D, Muntner P, Wu X, Reynolds K, Duan X, Chen C-S, Huang G, Bazzano LA, He J. Trends in overweight and obesity in Chinese adults: between 1991 and 1999-2000. Obesity (Silver Spring). 2008; 16(6): 1448–53.

Wirt T, Schreiber A, Kesztyus D, Steinacker JM. Early life cognitive abilities and body weight: cross-sectional study of the association of inhibitory control, cognitive flexibility, and sustained attention with BMI percentiles in primary school children. J Obes. 2015; 2015: 534651.

Wong JPS, Ho SY, Lai MK, Leung GM, Stewart SM, Lam TH. Overweight, obesity, weight-related concerns and behaviours in Hong Kong Chinese children and adolescents. Acta Paediatr. 2005; 94(5): 595-601.

World Health Organization (2005). Study on Global Ageing and Adult Health (SAGE), Pilot Study, 2005 (Data Set 27-28, Cunningham, Shayna.) [machine-readable data file and documentation]. Geneva, Switzerland: World Health Organization (Producer). Los Altos, CA: Sociometrics Corporation, Data Archive of Social Research on Aging (Producer & Distributor).

World Health Organization (WHO), Ministry of Health (Mongolia), National Medical Research Institute (Mongolia), Health Sciences University (Mongolia), National Oncology Center of Mongolia (Mongolia). Mongolia STEPS Noncommunicable Disease Risk Factors Survey 2005.

World Health Organization (WHO), Ministry of Health (Mongolia), National Medical Research Institute (Mongolia), Health Sciences University (Mongolia), National Oncology Center of Mongolia (Mongolia). Mongolia STEPS Noncommunicable Disease Risk Factors Survey 2009.

World Health Organization (WHO), Ministry of Health (Seychelles), Institute of Social and Preventive Medicine, University of Lausanne (Switzerland), University Hospital Center (Switzerland). Seychelles STEPS Noncommunicable Disease Risk Factors Survey 2004.

World Health Organization (WHO), Ministry of Health and Medical Education (Iran), Center for Non-Communicable Diseases Control (Iran). Iran STEPS Noncommunicable Disease Risk Factors Survey 2005.

World Health Organization (WHO), Ministry of Health and Medical Education (Iran), Center for Non-Communicable Diseases Control (Iran). Iran STEPS Noncommunicable Disease Risk Factors Survey 2007.

World Health Organization (WHO), Society for Local Integrated Development Nepal (SOLID Nepal), Central Bureau of Statistics (Nepal), Government of Nepal. Nepal STEPS Noncommunicable Disease Risk Factors Survey 2005.

World Health Organization (WHO), Society for Local Integrated Development Nepal (SOLID Nepal), Ministry of Health and Population (Nepal). Nepal - Bāgmatī STEPS Noncommunicable Disease Risk Factors Survey 2003.

World Health Organization (WHO). Austria World Health Survey 2003. Geneva, Switzerland: World Health Organization (WHO), 2005.

World Health Organization (WHO). Bosnia and Herzegovina World Health Survey 2003. Geneva, Switzerland: World Health Organization (WHO), 2005.

World Health Organization (WHO). Burkina Faso World Health Survey 2002-2003. Geneva, Switzerland: World Health Organization (WHO), 2005.

World Health Organization (WHO). Cape Verde STEPS Noncommunicable Disease Risk Factors Survey 2007.

World Health Organization (WHO). Central African Republic - Bangui STEPS Noncommunicable Disease Risk Factors Survey 2010.

World Health Organization (WHO). Chad World Health Survey 2003. Geneva, Switzerland: World Health Organization (WHO), 2005.

World Health Organization (WHO). China World Health Survey 2002. Geneva, Switzerland: World Health Organization (WHO), 2005.

World Health Organization (WHO). Comoros STEPS Noncommunicable Disease Risk Factors Survey 2011.

World Health Organization (WHO). Comoros World Health Survey 2003. Geneva, Switzerland: World Health Organization (WHO), 2005.

World Health Organization (WHO). Congo, Rep. World Health Survey 2003. Geneva, Switzerland: World Health Organization (WHO), 2005.

World Health Organization (WHO). Côte d'Ivoire World Health Survey 2003. Geneva, Switzerland: World Health Organization (WHO), 2005.

World Health Organization (WHO). Croatia World Health Survey 2003. Geneva, Switzerland: World Health Organization (WHO), 2005.

World Health Organization (WHO). Czech Republic World Health Survey 2002-2003. Geneva, Switzerland: World Health Organization (WHO), 2005.

World Health Organization (WHO). Denmark World Health Survey 2003. Geneva, Switzerland: World Health Organization (WHO), 2005.

World Health Organization (WHO). Dominica STEPS Noncommunicable Disease Risk Factors Survey 2007-2008.

World Health Organization (WHO). Ecuador World Health Survey 2003. Geneva, Switzerland: World Health Organization (WHO), 2005.

World Health Organization (WHO). Estonia World Health Survey 2003. Geneva, Switzerland: World Health Organization (WHO), 2005.

World Health Organization (WHO). Ethiopia - Addis Ababa STEPS Noncommunicable Disease Risk Factors Survey 2006.
World Health Organization (WHO). Ethiopia - Butajira STEPS Noncommunicable Disease Risk Factors Survey 2003.
World Health Organization (WHO). Ethiopia World Health Survey 2003. Geneva, Switzerland: World Health Organization (WHO), 2005.
World Health Organization (WHO). Finland World Health Survey 2004. Geneva, Switzerland: World Health Organization (WHO), 2005.
World Health Organization (WHO). France World Health Survey 2003. Geneva, Switzerland: World Health Organization (WHO), 2005.
World Health Organization (WHO). Gambia STEPS Noncommunicable Disease Risk Factors Survey 2010.
World Health Organization (WHO). Georgia STEPS Noncommunicable Disease Risk Factors Survey 2010.
World Health Organization (WHO). Georgia World Health Survey 2003. Geneva, Switzerland: World Health Organization (WHO), 2005.
World Health Organization (WHO). Germany World Health Survey 2004. Geneva, Switzerland: World Health Organization (WHO), 2005.
World Health Organization (WHO). Ghana World Health Survey 2003. Geneva, Switzerland: World Health Organization (WHO), 2005.
World Health Organization (WHO). Greece World Health Survey 2003. Geneva, Switzerland: World Health Organization (WHO), 2005.
World Health Organization (WHO). Guinea - Conakry STEPS Noncommunicable Disease Risk Factors Survey 2009.
World Health Organization (WHO). Hungary World Health Survey 2003. Geneva, Switzerland: World Health Organization (WHO), 2005.
World Health Organization (WHO). Ireland World Health Survey 2003. Geneva, Switzerland: World Health Organization (WHO), 2005.
World Health Organization (WHO). Israel World Health Survey 2003. Geneva, Switzerland: World Health Organization (WHO), 2005.
World Health Organization (WHO). Italy World Health Survey 2003. Geneva, Switzerland: World Health Organization (WHO), 2005.
World Health Organization (WHO). Kazakhstan World Health Survey 2002-2003. Geneva, Switzerland: World Health Organization (WHO), 2005.
World Health Organization (WHO). Kenya World Health Survey 2004. Geneva, Switzerland: World Health Organization (WHO), 2005.
World Health Organization (WHO). Laos World Health Survey 2003.
World Health Organization (WHO). Latvia World Health Survey 2003. Geneva, Switzerland: World Health Organization (WHO), 2005.
World Health Organization (WHO). Liberia STEPS Noncommunicable Disease Risk Factors Survey 2011.
World Health Organization (WHO). Luxembourg World Health Survey 2003. Geneva, Switzerland: World Health Organization (WHO), 2005.
World Health Organization (WHO). Malawi World Health Survey 2003. Geneva, Switzerland: World Health Organization (WHO), 2005.
World Health Organization (WHO). Malaysia World Health Survey 2003. Geneva, Switzerland: World Health Organization (WHO), 2005.
World Health Organization (WHO). Mali STEPS Noncommunicable Disease Risk Factors Survey 2007.
World Health Organization (WHO). Mali World Health Survey 2003. Geneva, Switzerland: World Health Organization (WHO), 2005.

World Health Organization (WHO). Mauritania World Health Survey 2003. Geneva, Switzerland: World Health Organization (WHO), 2005.
World Health Organization (WHO). Mauritius World Health Survey 2003. Geneva, Switzerland: World Health Organization (WHO), 2005.
World Health Organization (WHO). Mexico World Health Survey 2002-2003. Geneva, Switzerland: World Health Organization (WHO), 2005.
World Health Organization (WHO). Morocco World Health Survey 2003. Geneva, Switzerland: World Health Organization (WHO), 2005.
World Health Organization (WHO). Namibia World Health Survey 2003. Geneva, Switzerland: World Health Organization (WHO), 2005.
World Health Organization (WHO). Netherlands World Health Survey 2004. Geneva, Switzerland: World Health Organization (WHO), 2005.
World Health Organization (WHO). Norway World Health Survey 2003. Geneva, Switzerland: World Health Organization (WHO), 2005.
World Health Organization (WHO). Pakistan World Health Survey 2003-2004. Geneva, Switzerland: World Health Organization (WHO), 2005.
World Health Organization (WHO). Papua New Guinea STEPS Noncommunicable Disease Risk Factors Survey 2007-2008.
World Health Organization (WHO). Paraguay World Health Survey 2002-2003. Geneva, Switzerland: World Health Organization (WHO), 2005.
World Health Organization (WHO). Philippines World Health Survey 2003. Geneva, Switzerland: World Health Organization (WHO), 2005.
World Health Organization (WHO). Portugal World Health Survey 2003. Geneva, Switzerland: World Health Organization (WHO), 2006.
World Health Organization (WHO). Russia World Health Survey 2003. Geneva, Switzerland: World Health Organization (WHO), 2005.
World Health Organization (WHO). Sao Tome and Principe STEPS Noncommunicable Disease Risk Factors Survey 2008.
World Health Organization (WHO). Senegal World Health Survey 2003. Geneva, Switzerland: World Health Organization (WHO), 2005.
World Health Organization (WHO). Slovakia World Health Survey 2003. Geneva, Switzerland: World Health Organization (WHO), 2005.
World Health Organization (WHO). Slovenia World Health Survey 2003. Geneva, Switzerland: World Health Organization (WHO), 2005.
World Health Organization (WHO). South Africa World Health Survey 2002-2003. Geneva, Switzerland: World Health Organization (WHO), 2005.
World Health Organization (WHO). Spain World Health Survey 2002-2003. Geneva, Switzerland: World Health Organization (WHO), 2005.
World Health Organization (WHO). Sri Lanka World Health Survey 2003. Geneva, Switzerland: World Health Organization (WHO), 2005.
World Health Organization (WHO). Swaziland World Health Survey 2003. Geneva, Switzerland: World Health Organization (WHO), 2005.
World Health Organization (WHO). Sweden World Health Survey 2003. Geneva, Switzerland: World Health Organization (WHO), 2005.
World Health Organization (WHO). Tunisia World Health Survey 2003. Geneva, Switzerland: World Health Organization (WHO), 2005.

World Health Organization (WHO). Turkey World Health Survey 2003. Geneva, Switzerland: World Health Organization (WHO), 2005.
World Health Organization (WHO). Ukraine World Health Survey 2002-2003. Geneva, Switzerland: World Health Organization (WHO), 2005.
World Health Organization (WHO). United Arab Emirates World Health Survey 2003. Geneva, Switzerland: World Health Organization (WHO), 2005.
World Health Organization (WHO). United Kingdom World Health Survey 2004. Geneva, Switzerland: World Health Organization (WHO), 2005.
World Health Organization (WHO). Uruguay World Health Survey 2002-2003. Geneva, Switzerland: World Health Organization (WHO), 2005.
World Health Organization (WHO). Vietnam - Hà Tây STEPS Noncommunicable Disease Risk Factors Survey 2005.
World Health Organization (WHO). Vietnam World Health Survey 2002-2003. Geneva, Switzerland: World Health Organization (WHO), 2005.
World Health Organization (WHO). Zambia World Health Survey 2003. Geneva, Switzerland: World Health Organization (WHO), 2005.
World Health Organization (WHO). Zimbabwe World Health Survey 2003. Geneva, Switzerland: World Health Organization (WHO), 2005.
World Health Organization Regional Office for Europe (WHO/Europe). Inequalities in Young People's Health: HBSC International Report from the 2005-2006 Survey. Copenhagen, Denmark: World Health Organization Regional Office for Europe (WHO/Europe), 2008.
World Health Organization Regional Office for Europe (WHO/Europe). Social Determinants of Health and Well-being Among Young People: Health Behaviour in School-aged Children (HBSC) Study: International Report from the 2009/2010 Survey. Copenhagen, Denmark: World Health Organization Regional Office for Europe (WHO/Europe), 2012.
World Health Organization Regional Office for Europe (WHO/Europe). Young People's Health in Context. Health Behaviour in School-aged Children (HBSC) Study: International Report from the 2001-2002 Survey. Copenhagen, Denmark: World Health Organization Regional Office for Europe (WHO/Europe), 2004.
Wu N, Tang X, Wu Y, Qin X, He L, Wang J, Li N, Li J, Zhang Z, Dou H, Liu J, Yu L, Xu H, Zhang J, Hu Y, Iso H. Cohort profile: the Fangshan Cohort Study of cardiovascular epidemiology in Beijing, China. J Epidemiol. 2014; 24(1): 84–93.
Wu XY, Ohinmaa A, Veugelers PJ. Diet quality, physical activity, body weight and health-related quality of life among grade 5 students in Canada. Public Health Nutr. 2012; 15(1): 75-81.
Xie B, Chou C-P, Spruijt-Metz D, Reynolds K, Clark F, Palmer PH, Gallaher P, Sun P, Guo Q, Johnson CA. Socio-demographic and economic correlates of overweight status in Chinese adolescents. Am J Health Behav. 2007; 31(4): 339-52.
Xie YJ, Stewart SM, Lam TH, Viswanath K, Chan SS. Television viewing time in Hong Kong adult population: associations with body mass index and obesity. PLoS One. 2014; 9(1): e85440.
Xiong F, Garnett SP, Cowell CT, Biesheuvel C, Zeng Y, Long C-L, Wang Q, Wang D-G, Luo Y-H, Luo S-Q. Waist circumference and waist-to-height ratio in Han Chinese children living in Chongqing, south-west China. Public Health Nutr. 2011; 14(1): 20-6.
Xu Y-Q, Ji C-Y. Prevalence of the metabolic syndrome in secondary school adolescents in Beijing, China. Acta Paediatr. 2008; 97(3): 348-53.
Yan J, Liu L, Roebothan B, Ryan A, Chen Z, Yi Y, Wang P. A preliminary investigation into diet adequacy in senior residents of Newfoundland and Labrador, Canada: a cross-sectional study. BMC Public Health. 2014; 14: 302.

Yang J, Yu W, Zhou Q, Mahapatra T, Li Y, Zhang X, Chen L, Mahapatra S, Yan Y, Tang W. Burden and correlates of non-communicable-diseases among rural residents: a cross-sectional study in Hebei, China. BMC Public Health. 2015; 15: 571.

Yang Z-J, Liu J, Ge J-P, Chen L, Zhao Z-G, Yang W-Y, China National Diabetes and Metabolic Disorders Study Group. Prevalence of cardiovascular disease risk factor in the Chinese population: the 2007-2008 China National Diabetes and Metabolic Disorders Study. Eur Heart J. 2012; 33(2): 213–20.

Yannakoulia M, Panagiotakos DB, Pitsavos C, Stefanadis C. Correlates of BMI misreporting among apparently healthy individuals: the ATTICA study. Obesity (Silver Spring). 2006; 14(5): 894-901.

Yao Y, Ren X, Song X, He L, Jin Y, Chen Y, Lu W, Guo D, Ding L, Tang H, Wei N, Qiu S, Li C. The relationship between dental caries and obesity among primary school children aged 5 to 14 years. Nutr Hosp. 2014; 30(1): 60–5.

Yarnell JW, McCrum EE, Patterson CC, Skidmore P, Shields MD, McMahon J, Evans AE. Prevalence and awareness of excess weight in 13 and 14 year olds in Northern Ireland using recent international guidelines. Acta Paediatr. 2001; 90(12): 1435-9.

Yngve A, De Bourdeaudhuij I, Wolf A, Grjibovski A, Brug J, Due P, Ehrenblad B, Elmadfa I, Franchini B, Klepp K-I, Poortvliet E, Rasmussen M, Thorsdottir I, Perez Rodrigo C. Differences in prevalence of overweight and stunting in 11-year olds across Europe: The Pro Children Study. Eur J Public Health. 2008; 18(2): 126-30.

Yoo S, Kim H-B, Lee S-Y, Kim B-S, Kim J-H, Yu J-H, Kim B-J, Hong S-J. Association between obesity and the prevalence of allergic diseases, atopy, and bronchial hyperresponsiveness in Korean adolescents. Int Arch Allergy Immunol. 2011; 154(1): 42-8.

Yoo S, Lee S-Y, Kim K-N, Sung E. Obesity in Korean pre-adolescent school children: comparison of various anthropometric measurements based on bioelectrical impedance analysis. Int J Obes (Lond). 2006; 30(7): 1086-90.

Yoon K, Jang S-N, Chun H, Cho S-I. Self-reported anthropometric information cannot vouch for the accurate assessment of obesity prevalence in populations of middle-aged and older Korean individuals. Arch Gerontol Geriatr. 2014; 59(3): 584–92.

Yoshiike N, Seino F, Tajima S, Arai Y, Kawano M, Furuhata T, Inoue S. Twenty-year changes in the prevalence of overweight in Japanese adults: The National Nutrition Survey 1976-95. Obes Rev. 2002; 3(3): 183-90.

Yuca SA, Yilmaz C, Cesur Y, Dogan M, Kaya A, Basarangolu M. Prevalence of overweight and obesity in children and adolescents in eastern Turkey. J Clin Res Pediatr Endocrinol. 2010; 2(4): 159-63.

Yumuk VD, Hatemi H, Tarakci T, Uyar N, Turan N, Bagriacik N, Ipbuker A. High prevalence of obesity and diabetes mellitus in Konya, a central Anatolian city in Turkey. Diabetes Res Clin Pract. 2005; 70(2): 151-8.

Zaal AAB, Brebner J, Musaiger AO, Souza RD. Anthropometric characteristics and obesity among adolescents in the United Arab Emirates. East Mediterr Health J. 2011; 17(5): 382-6.

Zaghloul S, Al-Hooti SN, Al-Hamad N, Al-Zenki S, Alomirah H, Alayan I, Al-Attar H, Al-Othman A, Al-Shami E, Al-Somaie M, Jackson RT. Evidence for nutrition transition in Kuwait: over-consumption of macronutrients and obesity. Public Health Nutr. 2013; 16(4): 596-607.

Zaghloul S, Waslien C, Al Somaie M, Prakash P. Low Adherence of Kuwaiti Adults to Fruit and Vegetable Dietary Guidelines. East Mediterr Health J. 2012; 18(5): 461-7.

Zaman MM, Yoshiike N. Prevalence of overweight defined by body mass index in a rural adult population of Bangladesh. J Health Popul Nutr. 2003; 21(2): 162-3.

Zhang W, Li Y, Wang TD, Meng H-X, Min G-W, Fang Y-L, Niu X-Y, Ma L-S, Guo J-H, Zhang J, Sun M-Z, Li C-X. Nutritional status of the elderly in rural North China: a cross-sectional study. J Nutr Health Aging. 2014; 18(8): 730–6.

Zhang X, Sun Z, Zhang X, Zheng L, Liu S, Xu C, Li J, Zhao F, Li J, Hu D, Sun Y. Prevalence and associated factors of overweight and obesity in a Chinese rural population. Obesity (Silver Spring). 2008; 16(1): 168-71.

Zhang Y-X, Wang S-R. Differences in development and the prevalence of obesity among children and adolescents in different socioeconomic status districts in Shandong, China. Ann Hum Biol. 2012; 39(4): 290-6.

Zheng W, Chow W-H, Yang G, Jin F, Rothman N, Blair A, Li H-L, Wen W, Ji B-T, Li Q, Shu X-O, Gao Y-T. The Shanghai Women's Health Study: rationale, study design, and baseline characteristics. Am J Epidemiol. 2005; 162(11): 1123-31.

Zunzunegui MV, Sanchez MT, Garcia A, Ribera Casado JM, Otero A. Body Mass Index and Long-Term Mortality in an Elderly Mediterranean Population. J Aging Health. 2012; 24(1): 29-47.

References

1. Ng M, Liu P, Thomson B, Murray CJL. A novel method for estimating distributions of body mass index. Population Health Metrics 2016;14:6.

2. Cole TJ, Lobstein T. Extended international (IOTF) body mass index cut-offs for thinness, overweight and obesity. Pediatr Obes 2012;7(4):284–94.

3. GBD 2015 Risk Factors Collaborators. Global, regional, and national comparative risk assessment of 79 behavioural, environmental and occupational, and metabolic risks or clusters of risks, 1990–2015: a systematic analysis for the Global Burden of Disease Study 2015. Lancet 388:1659–724.

4. Ng M, Fleming T, Robinson M, et al. Global, regional, and national prevalence of overweight and obesity in children and adults during 1980–2013: a systematic analysis for the Global Burden of Disease Study 2013. The Lancet 2014;384(9945):766–81.

5. Ng M, Freeman MK, Fleming TD, et al. Smoking prevalence and cigarette consumption in 187 countries, 1980-2012. JAMA 2014;311(2):183–92.

6. Vander Hoorn S, EzzATI Ma, Rodgers Anthon, Lopez AD, Murray CJ. Estimating attributable burden of disease from exposure and hazard data. Comparative quantification of health risks: global and regional burden of disease attributable to selected major risk factors Geneva: World Health Organization 2004;2129–40.
